# Changing Prevalence of Immunocompromising Conditions in Patients Hospitalized with Severe Acute Respiratory Infection in Europe: Insights from the COVIDRIVE Study

**DOI:** 10.1007/s44197-025-00503-w

**Published:** 2026-03-09

**Authors:** Wilhelmine Meeraus, Ivana Prokić, J. Gabrielle Breugelmans, Konstantina Chatzikonstantinidou, Eduardo Conde-Sousa, Irma Casas, Wendy Hartig-Merkel, Gerrit Luit ten Kate, Sandra Manzanares-Laya, Charlotte Martin, Andrea Orsi, Susana Otero Romero, Sudhir Venkatesan, Marc-Alain Widdowson, Kaatje Bollaerts, Sylvia Taylor

**Affiliations:** 1 Medical Evidence, Vaccines & Immune Therapies, AstraZeneca, Cambridge, UK; 2 P95 Clinical and Epidemiology Services, Leuven, Belgium; 3 Coalition for Epidemic Preparedness Innovations (CEPI), Oslo, Norway; 4 Servicio de Medicina Preventiva, German Trias i Pujol University Hospital, Barcelona, Spain; 5 Department of infectious diseases and internal medicine, University Hospital Antwerp, Edegem, Belgium; 6 Infectious Diseases Department, Université Libre de Bruxelles, Centre Hospitalier Universitaire Saint-Pierre, Brussels, Belgium; 7 Interuniversity Research Center on Influenza and Other Transmissible Infections (CIRI- IT), Genoa, Italy; 8 Servicio de Medicina Preventiva y Epidemiología, Hospital Universitari Vall d’Hebron, Vall d’Hebron Barcelona Campus Hospitalari, Barcelona, Spain; 9 BPM Evidence Statistics, Biopharmaceuticals Medical, AstraZeneca, Cambridge, UK; 10 Institute Of Tropical Medicine, Antwerp, Belgium; 11 Present Address: World Health Organisation Europe, Copenhagen, Denmark; 12 Medical Evidence, Vaccines & Immune Therapies, BioPharmaceuticals Medical, AstraZeneca, Eastbrook House, Shaftesbury Road, Cambridge, CB2 8DU UK

**Keywords:** Severe acute respiratory infection, COVID-19, SARS-CoV-2, Immunocompromised host, Prevalence, Hospitalization, Comorbidity

## Abstract

**Background:**

Immunocompromised individuals are at substantially increased risk of severe acute respiratory infection (SARI), often compounded by inadequate response to vaccination. We assessed the prevalence of immunocompromising conditions (ICs) among hospitalized SARI patients, overall and by severe acute respiratory syndrome coronavirus 2 (SARS-CoV-2) status, focusing on temporal trends during the coronavirus disease 2019 (COVID-19) pandemic. We hypothesized that IC prevalence among SARS-CoV-2-positive SARI patients may have increased over time due to increasing background rates of SARS-CoV-2 exposure and differential vaccination coverage and efficacy among those with IC.

**Methods:**

We conducted a secondary data analysis of 5280 adults (≥ 18 years) hospitalized with SARI, enrolled in the COVIDRIVE study from five hospitals in Belgium, Italy, and Spain (June 2021-May 2023) and tested for SARS-CoV-2 by reverse transcription-polymerase chain reaction (RT-PCR). IC prevalence (including 95% confidence intervals [CIs)] was assessed using two definitions (main and alternative), excluding and including cancer patients. Changes in IC prevalence over time were assessed using linear trend analysis.

**Results:**

Among the 5280 SARI cases, IC prevalence by main definition was 13.9% (95% CI: 12.9–14.8) overall, increasing from 8.3% (95% CI: 3.7–15.8) to a peak of 22.0% (95% CI: 17.9–26.4) in Q3 2022; by alternative definition, IC prevalence was 28.8% (95% CI: 27.6–30.0) overall, increasing from 18.8% (95% CI: 11.5–28.0) to a peak of 38.5% (95% CI: 32.7–44.5) in Q2 2023. Among the 1924 SARS-CoV-2-positive cases, IC prevalence by main definition was 14.2% (95% CI: 12.7–15.9) overall, increasing from 3.4% (95% CI: 0.1–17.8) to a peak of 23.8% (95% CI: 17.4–31.1) in Q3 2022; by alternative definition, IC prevalence was 29.6% (95% CI: 27.6–31.7) overall, increasing from 3.4% (95% CI: 0.1–17.8) to a peak of 46.8% (95% CI: 32.1–61.9) in Q2 2023. The larger observed increase in IC prevalence (by both definitions) over time among SARS-CoV-2-positives, relative to SARI patients without SARS-CoV-2, was statistically significant.

**Conclusions:**

Immunocompromised individuals represent a high proportion of hospitalized SARI cases, with results suggesting IC prevalence was higher during later periods of the COVID-19 pandemic. As immunocompromised individuals often respond inadequately to vaccination, alternative strategies are needed to better protect this vulnerable group.

**Supplementary Information:**

The online version contains supplementary material available at 10.1007/s44197-025-00503-w.

## Introduction

Immunocompromised individuals represent a diverse yet vulnerable population that face an elevated risk of severe disease from respiratory viruses, which is often compounded by an inadequate vaccine response and limited options for disease prevention [1, 2]. As immunocompromising conditions (ICs) are not well defined, studies have estimated that prevalence among adolescents/adults may range between about 1–7%, with prevalence potentially increasing in the future as populations age, further conditions are identified, and more immune-modulating therapies become available [3–5]. One study of nationwide US claims data, conducted before the coronavirus disease 2019 (COVID-19) pandemic, has suggested that individuals with ICs may represent up to a third of severe acute respiratory infection (SARI) cases [5].

ICs are classified as either primary (generally inherited absence or deficiency of humoral and/or cellular immunity) or secondary (acquired deficiency of humoral and/or cellular immunity as a result of a disease, such as cancer, or its therapy) [6]. Cancer patients were more likely to die throughout the COVID-19 pandemic, and as the pandemic progressed, in-hospital mortality reduced more slowly for patients with ICs than for those without [7]. More broadly, it is recognized that community-acquired viral respiratory tract infections in cancer patients will progress to pneumonia in 30% of cases, and to death in approximately 25% [8].

The COVID-19 public health emergency was declared over on May 5th 2023 by the World Health Organization (WHO) [9], but Severe Acute Respiratory Syndrome Coronavirus 2 (SARS-CoV-2) that causes COVID-19 is now endemic globally. Although COVID-19 is mild for most individuals, the risk of SARI and COVID-19 is elevated in individuals with multiple comorbidities [10, 11] and the risk of progression to severe COVID-19 is increased by the presence of underlying conditions (including ICs), irrespective of age [12].

In immunocompromised individuals, COVID-19 vaccines elicit shorter duration of protection, lower seroconversion rates post-vaccination, and reduced neutralization activity [13–15]. Such individuals have been shown to have higher rates of SARI due to (SARS-CoV-2) breakthrough infection and worse outcomes despite vaccination [16], including hospitalization [17], than individuals without ICs. Furthermore, during the COVID-19 pandemic, patients with ICs and COVID-19-related SARI had more severe in-hospital disease progression compared to patients without ICs.

Our study aimed to evaluate the prevalence of ICs and other comorbid conditions among hospitalized SARI patients, overall and stratified by SARS-CoV-2 test status during the COVID-19 pandemic, from the initial phases of vaccination roll-out to the end of the pandemic declaration and transition into an endemic state. We hypothesized that over time, IC prevalence among SARS-CoV-2 positives may have increased with increasing background rates of SARS-CoV-2 exposure and differential vaccination coverage and efficacy among those with ICs. We conducted a secondary data use analysis, using data from the COVIDRIVE study, which since 2021 monitors the effectiveness of COVID-19 vaccines in Europe and collects information on ICs and other comorbidities in individuals hospitalized with SARI (EUPAS42328). The study was conducted by the COVIDRIVE European public-private partnership, and the COVIDRIVE Consortium was established in March 2021. It expanded its scope in 2024, evolving into id.DRIVE (https://iddrive.eu/), to facilitate the conduct of observational studies on infectious diseases, vaccines, related preventive measures, therapeutics, and diagnostics in Europe.

## Methods

### Study Objectives

The primary objective of this study was to estimate the prevalence of ICs among hospitalized SARI patients with and without SARS-CoV-2, over the course of the COVID-19 pandemic. Secondary objectives were to describe the demographic and clinical characteristics of SARI patients (overall, and by SARS-CoV-2 test status), and to estimate the prevalence of other chronic conditions among SARI patients (also with stratification by SARS-CoV-2 test status). An exploratory objective was to estimate the prevalence of co-occurrence of chronic conditions among SARI patients. Sensitivity analyses were conducted repeating all analyses using an alternative SARI definition.

### Study Design and Data Sources

This study is a secondary data use study of real-world data collected by COVIDRIVE from a large sample of hospitalized SARI patients during the majority of the COVID-19 pandemic period. The COVIDRIVE study, which is still ongoing as part of id.DRIVE, is a multi-center, multi-country, hospital-based, test-negative case-control study (www.iddrive.eu). The primary COVIDRIVE data collection study was designed to estimate the effectiveness of COVID-19 vaccines against SARI hospitalization due to SARS-CoV-2. During the study period, eight study sites (i.e., hospitals or hospital networks) in four countries (Austria, Belgium, Italy and Spain) recruited SARI patients. However, only data from five study sites (in three countries) were considered valid for our secondary data use study: Belgium (Centre Hospitalier Universitaire Saint-Pierre [CHU Saint-Pierre] and Universitair Ziekenhuis Antwerpen [UZA]), Italy (Centro Interuniversitario di Ricerca sull’Influenza e le altre Infezioni Trasmissibili [CIRI-IT]), and Spain (Hospital Universitario Germans Trias i Pujol [GTPUH] and Hospital Universitari Vall d’Hebron [HUVH]). We excluded data from one Spanish study site due to a different IC definition than that used by the other study sites, and we excluded one Austrian and one Belgian study site due to a low number of SARS-CoV-2-negative study participants with few or no controls having ICs. Details of the included study sites are presented in Table S1.

### Study Population, Sample Selection

#### SARI Definition

Included in the analysis were adults (≥ 18 years old) presenting with SARI symptoms at one of the participating study sites during the study period (01 June 2021-31 May 2023). According to the main COVIDRIVE definition, a SARI patient was defined as a hospitalized patient with clinical suspicion of a respiratory infection, symptom onset within 14 days prior to hospital admission, and at least one of the following symptoms: fever, cough, shortness of breath or sudden onset of anosmia, ageusia or dysgeusia, according to the European Centre of Disease Prevention and Control (ECDC) definition of SARI [18]. This definition was considered as the *main SARI definition* in the present study (Table 1), while an *alternative SARI definition* by WHO [19] was explored via sensitivity analysis. WHO considered SARI patients as those hospitalized with symptom onset within the 10 days prior to hospital admission and the following symptoms: a history of fever or measured fever of ≥ 38 °C AND cough.

**Table 1:** SARI, IC status, and SARS-CoV-2 test status definitions as defined in COVIDRIVE and used in this study

SARI patient	
Main definition (ECDC definition ^1^)	Alternative definition (WHO definition ^2^) used in sensitivity analysis
Hospitalized patient with a suspicion of a respiratory infection with symptom onset within the 14 days prior to hospital admission and at least one of the following symptoms: • Fever, • Cough, • Shortness of breath, • Sudden onset of anosmia, ageusia or dysgeusia	Hospitalized patient with a severe acute respiratory infection, with symptom onset within the 10 days prior to hospital admission and the following symptoms: • A history of fever or measured fever of ≥ 38 °C AND • Cough
IC status	
***Main IC definition*** ^***3***^	***Alternative IC definition*** ^*4*^
Having an IC defined by any of the following ICD-10 codes: • B20-B24, D80–84, D89, Z94 INCLUDING: HIV infections, immunodeficiencies & organ transplants. Or iatrogenic: ≥2 week systemic treatment, in the 3 months preceding symptom onset, with any of the following: corticosteroid (≥ 20 mg prednisolone daily or equivalent), ciclosporin, tacrolimus, mycophenolate, methotrexate, azathioprine, TNF-α blockers and other biological or cytostatic drugs with immunosuppressive effect. EXCLUDING: disorders of the immune system which do not lead to immunosuppression (e.g., some autoimmune conditions).	Having at least one of the following two chronic conditions: • IC (as per the main definition) AND/OR • cancer as defined by any of the following ICD-10 codes: C00-97, D37-48, Z85, Z92.3, Z92.6. INCLUDING: all malignant neoplasms (both solid and hematologic) with potential to metastasize, either in treatment, active follow-up, or < 5 years post curative treatment. EXCLUDING: Benign and in situ neoplasms. Basal cell carcinomas. Any cancer previously treated with curative intent and in complete remission for ≥ 5 years.
**SARS-CoV-2 test status**	
***Main definition*** ^***3***^	***Alternative definition*** ^4^ ***used in sensitivity analysis***
The specimen(s) collected between 14 days prior and up to *72* hours after hospital admission.	The specimen(s) collected between 14 days prior and up to *24* hours after hospital admission.

^1^ https://www.ecdc.europa.eu/en/publications-data/interim-public-health-considerations-covid-19-vaccination-roll-out-during-2023; ^2^ https://www.who.int/teams/global-influenza-programme/surveillance-and-monitoring/case-definitions-for-ili-and-sari; ^3^ as per COVIDRIVE Master Protocol v4.0; ^4^ specific for this secondary data analysis. Abbreviations: ECDC, European Centre for Disease Prevention and Control; HIV, human immunodeficiency virus; IC, immunocompromising condition; ICD, international classification of diseases, 10th revision; SARI, severe acute respiratory infection; SARS-CoV-2, severe acute respiratory syndrome coronavirus 2; TNF, tumor necrosis factor; WHO, World Health Organization

#### Inclusion and Exclusion Criteria

The COVIDRIVE inclusion criteria required the study participants to meet the SARI definition and:

- to be eligible for COVID-19 vaccination per their national or regional immunization recommendations prior to hospital admission and.
- to be hospitalized for at least one day (defined as at least one overnight stay).

Per COVIDRIVE study protocol, the following patients were excluded:

- patients who had been hospitalized due to COVID-19 within 3 months prior to their current hospital admission (hospital transfers were not considered a prior hospitalization),
- patients who could not be swabbed for test specimen collection (due to severe nasal septum deviation, nasal obstruction or other conditions that contraindicated nasopharyngeal swabbing), and.
- patients with incomplete data on test status, sex, age, study site, symptom onset date and vaccination variables.

For this secondary analysis, we furthermore excluded 66 (1.2%) patients with:

- incomplete data on IC status and/or other comorbidities (cancer, asthma, lung disease, cardiovascular disease, hypertension, type 2 diabetes, liver disease, and renal disease).

### Definitions of ICs (IC Status)

We used the main COVIDRIVE definition of ICs as the *main IC definition* (Table 1), including International Statistical Classification of Diseases and Related Health Problems, Tenth Revision [ICD-10] codes B20-B24, D80–84, D89, Z94 and conditions such as human immunodeficiency virus (HIV) infections, immunodeficiencies, organ transplants, and iatrogenic conditions, as recorded in the hospital medical records. This definition excluded disorders of the immune system which do not lead to being immunocompromised (e.g., some autoimmune conditions). As this stringent main definition may not have captured patients who had ICs as a result of cancer treatment, we also used a broader *alternative IC definition* (having at least one of the following two chronic conditions: IC as per the main definition and/or cancer as defined in COVIDRIVE by any of the ICD-10 codes C00-97, D37-48, Z85, Z92.3, Z92.6, Table 1).

### SARS-CoV-2 Testing (SARS-CoV-2 Test Status)

All study participants in COVIDRIVE were tested for SARS-CoV-2 using reverse transcription polymerase chain reaction (RT-PCR) or similar molecular assays. An individual was considered SARS-CoV-2 ‘positive’ if they had at least one positive test result on a sample taken from 14 days prior and up to 72 h after hospital admission. Whereas an individual was considered SARS-CoV-2 ‘negative’ if all samples taken during that same time period were negative. This outcome definition was referred to as the *main SARS-CoV-2 test status definition* (Table 1). Furthermore, a more stringent *alternative SARS-CoV-2 test status definition* was defined based on sampling from 14 days prior up to 24 h after hospital admission, and was explored by means of sensitivity analysis in order to fully exclude hospital-acquired SARS-CoV-2 infections.

### Other Variables

Additional data on SARI severity, length of hospital stay, chronic conditions (comorbidities), smoking history, long-term care facility (LTCF) residence, sex, age, symptoms (and onset date) and COVID-19 vaccination history and date were also collected for COVIDRIVE study participants, and we included these variables in the secondary data analysis (Table 2**)**.

**Table 2:** Other variables collected as defined in COVIDRIVE and used in this study

Variable	Explanation	Categories (if applicable)
**Age**	Years of age at hospital admission	NA
**Sex**	*Sex assigned at birth*	‘Male’ ‘Female’
**Country**	Country of the participating hospital	‘Belgium’ ‘Italy’ ‘Spain’
**Study site**	Participating hospital	‘CHU Saint-Pierre’ ‘CIRI-IT’ ‘GTPUH’ ‘HUVH’ ‘UZA’
**COVID-19 vaccination history at recruitment**	Vaccination status^1^	‘Unvaccinated’ ‘Incomplete primary series’ ‘Primary series completed but no boosters’ ‘At least one booster dose’
	Number of booster doses, for patients with complete primary series	‘0’ ‘1’ ‘2’ ‘3+’
	Time in days since last vaccine dose	NA
	Categories of time since last vaccine dose	< 2 months 2–4 months 4–6 months 6–8 months ≥ 8 months
**SARI severity level**	Severity of SARI defined using presence/absence of ICU admission and in-hospital death	‘Hospital admission without ICU admission and without in-hospital death’ ‘ICU admission with or without in-hospital death’ ‘ICU admission without in-hospital death’ ‘In-hospital death’
**Length of hospital stay**	The number of overnights spent at the hospital from hospital admission until hospital discharge or death. COVID-19 hospitalization that took place within 3 months of the first COVID-19 hospitalization was considered part of the same episode; in this case, the lengths of stay of the two admissions were summed.	NA
**Chronic comorbidities**	Number of chronic comorbidities other than ICs	‘0’ ‘1’ ‘2’ ‘3+’
	Comorbidity categories as defined by ICD-10 codes (Table S2)	‘Asthma’ ‘Lung disease’ ‘Cardiovascular disease’ ‘Hypertension’ ‘Chronic liver disease’ ‘Chronic kidney disease’ ‘Type 2 diabetes’ ‘Cancer’
**Smoking history**	Categories of smoking behavior at hospital admission	‘Never smoker’ ‘Former smoker i.e., smoke-free for at least 28 days’ ‘Current smoker’
**Long-term care facility (LTCF) residence**	Residence in LTCF prior to hospitalization	‘Yes’ ‘No’
**Date of SARI symptom onset**	Fiscal quarter (3-month period)	‘Q2 2021 (01 June 2021-30 Jun 2021)’ ‘Q3 2021 (01 Jul 2021-30 Sep 2021)’ ‘Q4 2021 (01 Oct 2021-31 Dec 2021)’ ‘Q1 2022 (01 Jan 2022-31 Mar 2022)’ ‘Q2 2022 (01 Apr 2022-30 Jun 2022)’ ‘Q3 2022 (01 Jul 2022-30 Sep 2022)’ ‘Q4 2022 (01 Oct 2022-31 Dec 2022)’ ‘Q1 2023 (01 Jan 2023-31 Mar 2023)’ ‘Q2 2023 (01 Apr 2023-31 May 2023)’
**SARI symptoms**	Symptoms	‘Cough’ ‘Fever’ ‘Shortness of breath’ ‘Sudden onset of anosmia, ageusia, or dysgeusia’
	Number of symptoms	‘1’ ‘2’ ‘3+’
**Date of hospitalization**	Date of admission to hospital	NA

^1^ Primary series for all participants was defined as having received one or two COVID-19 vaccine doses (i.e., one dose for the JCOVDEN COVID-19 vaccine or two doses for all other COVID-19 vaccines). Abbreviations: CHU Saint-Pierre, Centre Hospitalier Universitaire Saint-Pierre; CIRI-IT, Centro Interuniversitario di Ricerca sull’Influenza e le altre Infezioni Trasmissibili; GTPUH, Hospital Universitario Germans Trias i Pujol; HUVH, Hospital Universitari Vall d’Hebron; ICU, intensive care unit; IQR, interquartile range; NA, not applicable; SARI, severe acute respiratory infection; SD, standard deviation; UZA, Universitair Ziekenhuis Antwerpen

### Statistical Analyses

We conducted all analyses on a pooled dataset combining data from the five different study sites (Table S1). We performed all data transformations and statistical analyses based on a pre-specified Statistical Analysis Plan, using the statistical programming environment R (version 4.0.2) and used the R package “*renv*” to ensure reproducibility of the analyses [20].

We estimated the IC prevalence among SARI patients using both the main and alternative definitions. We assessed patients’ characteristics, such as demographics, comorbidities, or severity outcomes by calculating counts and percentages by IC status (for both the main and the alternative definition) and by SARS-CoV-2 test status. We assessed the evolution of the prevalence of IC over time by calendar quarter and, alternatively, by modeling the IC status as a function of the study sites, SARS-CoV-2 test status and disease onset date (smoothed time trends with penalized cubic regression splines [21]. We calculated the 95% confidence intervals (CIs) for all prevalence estimates using the exact binomial method [22].

To further assess changes in the prevalence of ICs over time, we performed a linear trend analysis which incorporated IC prevalence, SARS-CoV-2 test status and date of symptom onset. These variables were fitted into a linear regression model, where the prevalence was modeled as a function of the date of symptom onset, test status, and their interaction.

Additionally, we conducted sensitivity analyses by repeating the above analyses using the sensitivity definitions for SARI and SARS-CoV-2 test status.

#### Missing Data

We included only study participants with complete information on key variables, i.e., SARS-CoV-2 test status, sex, age, study site, date of symptom onset, vaccination history, IC status, and chronic conditions, while for other variables, missingness was allowed and whenever > 5% of missing data were present for a given variable, we added a ‘missing’ category to the descriptive tables.

## Results

We included a total of 5280 study participants, recruited from 01 June 2021 to 31 May 2023, with recruitment start at HUVH in Spain and CIRI-IT in Italy not starting until 30 Oct 2021 and 16 Nov 2021, respectively. Approximately one third of SARI patients (36.4%, 1924) tested positive for SARS-CoV-2, while two-thirds (63.6%, 3356) tested negative (Table 3).

**Table 3:** Demographics, clinical outcomes of study population overall, by IC status (main), and by SARS-CoV-2 test status

				SARS-CoV-2 test status of individuals hospitalized with SARI ^d^									
	All individuals hospitalized with SARI ^c^			Positive				Negative					
	Overall (*N* = 5280)	IC ^a^ (*N* = 732)	Non-IC (*N* = 4548)	Overall (*N* = 1924)	IC (*N* = 274)	Non-IC (*N* = 1650)		Overall (*N* = 3356)			IC (*N* = 458)		Non-IC (*N* = 2898)
**Demographics**													
**Age**,** (years)**													
Mean (SD)	67.5 (17.0)	62.5 (15.2)	68.3 (17.1)	66.9 (16.6)	63.0 (14.7)	67.6 (16.8)		67.9 (17.1)			62.2 (15.5)		68.8 (17.2)
Median (IQR)	71.0 (58.0, 80.0)	65.0 (54.0, 74.0)	72.0 (58.0, 81.0)	70.0 (57.0, 79.0)	65.0 (55.0, 74.0)	71.0 (57.0, 80.0)		71.0 (58.0, 81.0)			65.0 (53.0, 73.0)		72.0 (59.0, 82.0)
Range	18.0–103.0	18.0–93.0	18.0–103.0	18.0–102.0	21.0–93.0	18.0–102.0		18.0–103.0			18.0–92.0		18.0–103.0
**Sex**,** n (%)**													
Male	2982 (56.5)	423 (57.8)	2559 (56.3)	1119 (58.2)	150 (54.7)	969 (58.7)		1863 (55.5)			273 (59.6)		1590 (54.9)
Female	2298 (43.5)	309 (42.2)	1989 (43.7)	805 (41.8)	124 (45.3)	681 (41.3)		1493 (44.5)			185 (40.4)		1308 (45.1)
**Country**,** n (%)**													
Belgium	568 (10.8)	83 (11.3)	485 (10.7)	306 (15.9)	60 (21.9)	246 (14.9)		262 (7.8)			23 (5.0)		239 (8.2)
Italy	1289 (24.4)	73 (10.0)	1216 (26.7)	481 (25.0)	31 (11.3)	450 (27.3)		808 (24.1)			42 (9.2)		766 (26.4)
Spain	3423 (64.8)	576 (78.7)	2847 (62.6)	1137 (59.1)	183 (66.8)	954 (57.8)		2286 (68.1)			393 (85.8)		1893 (65.3)
**Study site**,** n (%)**													
CIRI-IT	1289 (24.4)	73 (10.0)	1216 (26.7)	481 (25.0)	31 (11.3)	450 (27.3)		808 (24.1)			42 (9.2)		766 (26.4)
GTPUH	1744 (33.0)	274 (37.4)	1470 (32.3)	459 (23.9)	49 (17.9)	410 (24.8)		1285 (38.3)			225 (49.1)		1060 (36.6)
CHU Saint-Pierre	173 (3.3)	6 (0.8)	167 (3.7)	52 (2.7)	2 (0.7)	50 (3.0)		121 (3.6)			4 (0.9)		117 (4.0)
UZA	395 (7.5)	77 (10.5)	318 (7.0)	254 (13.2)	58 (21.2)	196 (11.9)		141 (4.2)			19 (4.1)		122 (4.2)
HUVH	1679 (31.8)	302 (41.3)	1377 (30.3)	678 (35.2)	134 (48.9)	544 (33.0)		1001 (29.8)			168 (36.7)		833 (28.7)
**Smoking**,** n (%)**													
Never smoker	2009 (38.0)	299 (40.8)	1710 (37.6)	760 (39.5)	119 (43.4)	641 (38.8)		1249 (37.2)			180 (39.3)		1069 (36.9)
Former smoker	1347 (25.5)	200 (27.3)	1147 (25.2)	468 (24.3)	49 (17.9)	419 (25.4)		879 (26.2)			151 (33.0)		728 (25.1)
Current smoker	870 (16.5)	107 (14.6)	763 (16.8)	227 (11.8)	31 (11.3)	196 (11.9)		643 (19.2)			76 (16.6)		567 (19.6)
Missing	1054 (20.0)	126 (17.2)	928 (20.4)	469 (24.4)	75 (27.4)	394 (23.9)		585 (17.4)			51 (11.1)		534 (18.4)
**Long-term care facility residence**,** n (%)**													
Yes	201 (3.9)	13 (1.9)	188 (4.2)	59 (3.1)	4 (1.5)	55 (3.4)		142 (4.3)			9 (2.0)		133 (4.7)
**Vaccination status at time of hospital admission**													
Unvaccinated	641 (12.1)	50 (6.8)	591 (13.0)	417 (21.7)	25 (9.1)	392 (23.8)		224 (6.7)			25 (5.5)		199 (6.9)
Incomplete primary series	134 (2.5)	14 (1.9)	120 (2.6)	48 (2.5)	5 (1.8)	43 (2.6)		86 (2.6)			9 (2.0)		77 (2.7)
Primary series completed but no boosters	1309 (24.8)	183 (25.0)	1126 (24.8)	454 (23.6)	52 (19.0)	402 (24.4)		855 (25.5)			131 (28.6)		724 (25.0)
At least one booster dose	3196 (60.5)	485 (66.3)	2711 (59.6)	1005 (52.2)	192 (70.1)	813 (49.3)		2191 (65.3)			293 (64.0)		1898 (65.5)
**Number of booster doses** ^**b**^, **n (%)**													
0	1309 (29.1)	183 (27.4)	1126 (29.3)	454 (31.1)	52 (21.3)	402 (33.1)		855 (28.1)			131 (30.9)		724 (27.6)
1	2375 (52.7)	335 (50.1)	2040 (53.2)	794 (54.4)	137 (56.1)	657 (54.1)		1581 (51.9)			198 (46.7)		1383 (52.7)
2	775 (17.2)	125 (18.7)	650 (16.9)	195 (13.4)	42 (17.2)	153 (12.6)		580 (19.0)			83 (19.6)		497 (19.0)
3+	46 (1.0)	25 (3.7)	21 (0.5)	16 (1.1)	13 (5.3)	3 (0.2)		30 (1.0)			12 (2.8)		18 (0.7)
**Time since last vaccine dose**,** (days)**													
Mean (SD)	187.8 (138.8)	197.5 (142.5)	186.1 (138.1)	175.7 (115.8)	184.4 (117.7)	174.0 (115.4)		193.6 (148.2)			205.0 (154.6)		191.8 (147.1)
Median (IQR)	156.0 (81.0, 260.0)	165.0 (85.5, 274.8)	155.0 (80.0, 258.0)	163.0 (89.0, 233.5)	163.0 (98.0, 257.0)	163.0 (88.0, 230.8)		153.0 (77.8, 287.0)			166.0 (83.0, 296.0)		150.0 (76.0, 285.0)
Range	1.0–782.0	1.0–710.0	1.0–782.0	1.0–662.0	1.0–662.0	1.0–653.0		1.0–782.0			2.0–710.0		1.0–782.0
**Time since last vaccine dose**,** n (%)**													
< 2 months	798 (17.2)	109 (16.0)	689 (17.4)	225 (14.9)	36 (14.5)	189 (15.0)		573 (18.3)			73 (16.9)		500 (18.5)
[2–4) months	970 (20.9)	132 (19.4)	838 (21.2)	316 (21.0)	50 (20.1)	266 (21.1)		654 (20.9)			82 (18.9)		572 (21.2)
[4–6) months	916 (19.7)	130 (19.1)	786 (19.9)	325 (21.6)	51 (20.5)	274 (21.8)		591 (18.9)			79 (18.2)		512 (19.0)
[6–8) months	637 (13.7)	93 (13.6)	544 (13.7)	286 (19.0)	40 (16.1)	246 (19.6)		351 (11.2)			53 (12.2)		298 (11.0)
≥ 8 months	1318 (28.4)	218 (32.0)	1100 (27.8)	355 (23.6)	72 (28.9)	283 (22.5)		963 (30.7)			146 (33.7)		817 (30.3)
**Symptoms**													
**SARI symptoms**													
Cough	3577 (71.4)	456 (65.0)	3121 (72.5)	1246 (73.2)	177 (70.5)	1069 (73.7)	2331 (70.5)			279 (61.9)			2052 (71.8)
Fever	3100 (62.2)	450 (64.2)	2650 (61.8)	1110 (65.3)	166 (66.4)	944 (65.1)	1990 (60.5)			284 (63.0)			1706 (60.2)
Shortness of breath	3524 (70.1)	446 (63.4)	3078 (71.2)	1235 (71.9)	155 (61.0)	1080 (73.8)	2289 (69.2)			291 (64.7)			1998 (69.9)
Anosmia, ageusia or dysgeusia	172 (3.6)	21 (3.2)	151 (3.7)	140 (9.0)	14 (6.5)	126 (9.3)	32 (1.0)			7 (1.6)			25 (0.9)
**Number of SARI symptoms**,** n (%)**													
1	1152 (24.5)	208 (32.4)	944 (23.2)	374 (24.1)	66 (31.1)	308 (23.0)	778 (24.7)			142 (33.0)			636 (23.4)
2	2215 (47.1)	281 (43.8)	1934 (47.6)	611 (39.3)	88 (41.5)	523 (39.0)	1604 (50.9)			193 (44.9)			1411 (51.9)
3+	1336 (28.4)	153 (23.8)	1183 (29.1)	568 (36.6)	58 (27.4)	510 (38.0)	768 (24.4)			95 (22.1)			673 (24.7)
**Hospital outcomes**													
**Length of stay**,** (days)**													
Mean (SD)	10.1 (13.8)	11.1 (16.1)	10.0 (13.4)	12.2 (18.0)	14.0 (21.1)	12.0 (17.4)		8.9 (10.4)		9.3 (11.8)			8.9 (10.2)
Median (IQR)	7.0 (4.0, 12.0)	6.0 (4.0, 12.0)	7.0 (4.0, 12.0)	7.0 (4.0, 14.0)	6.0 (4.0, 14.0)	7.0 (4.0, 14.0)		6.0 (3.0, 11.0)		6.0 (4.0, 11.0)			6.0 (3.0, 11.0)
Range	1.0–403.0	1.0–173.0	1.0–403.0	1.0–403.0	1.0–141.0	1.0–403.0		1.0–173.0		1.0–173.0			1.0–166.0
**Severity**,** n (%)**													
Hospitalization without ICU admission or death	4564 (86.5)	628 (85.8)	3936 (86.6)	1525 (79.3)	227 (82.8)	1298 (78.7)		3039 (90.6)		401 (87.6)			2638 (91.0)
ICU admission without death	334 (6.3)	36 (4.9)	298 (6.6)	207 (10.8)	18 (6.6)	189 (11.5)		127 (3.8)		18 (3.9)			109 (3.8)
ICU admission (with or without death)	435 (8.2)	63 (8.6)	372 (8.2)	286 (14.9)	39 (14.2)	247 (15.0)		149 (4.4)		24 (5.2)			125 (4.3)
In-hospital death	381 (7.2)	68 (9.3)	313 (6.9)	191 (9.9)	29 (10.6)	162 (9.8)		190 (5.7)		39 (8.5)			151 (5.2)
**Comorbidities**													
**Number of comorbidities other than IC** ^**a**^, **n (%)**													
No other comorbidities	968 (18.3%)	64 (8.7%)	904 (19.9%)	386 (20.1%)	23 (8.4%)	363 (22.0%)		582 (17.3%)	41 (9.0%)			541 (18.7%)	
1 other comorbidity	1157 (21.9%)	180 (24.6%)	977 (21.5%)	419 (21.8%)	73 (26.6%)	346 (21.0%)		738 (22.0%)	107 (23.4%)			631 (21.8%)	
2 other comorbidities	1191 (22.6%)	179 (24.5%)	1012 (22.3%)	471 (24.5%)	72 (26.3%)	399 (24.2%)		720 (21.5%)	107 (23.4%)			613 (21.2%)	
3 + other comorbidities	1964 (37.2%)	309 (42.2%)	1655 (36.4%)	648 (33.7%)	106 (38.7%)	542 (32.8%)		1316 (39.2%)	203 (44.3%)			1113 (38.4%)	
**Comorbidities** ^**e**^, **n (%)**													
Asthma	401 (7.6%)	46 (6.3%)	355 (7.8%)	116 (6.0%)	16 (5.8%)	100 (6.1%)		285 (8.5%)	30 (6.6%)			255 (8.8%)	
Lung disease	1737 (32.9%)	283 (38.7%)	1454 (32.0%)	513 (26.7%)	94 (34.3%)	419 (25.4%)		1224 (36.5%)	189 (41.3%)			1035 (35.7%)	
Cardiovascular disease	2106 (39.9%)	250 (34.2%)	1856 (40.8%)	743 (38.6%)	91 (33.2%)	652 (39.5%)		1363 (40.6%)	159 (34.7%)			1204 (41.5%)	
Hypertension	2623 (49.7%)	318 (43.4%)	2305 (50.7%)	966 (50.2%)	125 (45.6%)	841 (51.0%)		1657 (49.4%)	193 (42.1%)			1464 (50.5%)	
Chronic liver disease	399 (7.6%)	88 (12.0%)	311 (6.8%)	121 (6.3%)	30 (10.9%)	91 (5.5%)		278 (8.3%)	58 (12.7%)			220 (7.6%)	
Chronic kidney disease	904 (17.1%)	195 (26.6%)	709 (15.6%)	344 (17.9%)	78 (28.5%)	266 (16.1%)		560 (16.7%)	117 (25.5%)			443 (15.3%)	
Type 2 diabetes	1390 (26.3%)	176 (24.0%)	1214 (26.7%)	503 (26.1%)	65 (23.7%)	438 (26.5%)		887 (26.4%)	111 (24.2%)			776 (26.8%)	
Cancer	1172 (22.2%)	383 (52.3%)	789 (17.3%)	424 (22.0%)	128 (46.7%)	296 (17.9%)		748 (22.3%)	255 (55.7%)			493 (17.0%)	
Alternative IC ^f^	1521 (28.8%)	732 (100.0%)	789 (17.3%)	570 (29.6%)	274 (100.0%)	296 (17.9%)		951 (28.3%)	458 (100.0%)			493 (17.0%)	

^a^ Main IC definition (Table 1); ^b^ Among the patients eligible to receive booster (completed primary series); ^c^ Main SARI definition (Table 1); ^d^ Main SARS-CoV-2 test status definition (Table 1); ^e^ Not mutually exclusive; ^f^ Alternative IC definition – main IC and cancer (Table 1). Abbreviations: CHU Saint-Pierre, Centre Hospitalier Universitaire Saint-Pierre; CI, confidence interval; CIRI-IT, Centro Interuniversitario di Ricerca sull’Influenza e le altre Infezioni Trasmissibili; GTPUH, Hospital Universitario Germans Trias i Pujol; HUVH, Hospital Universitari Vall d’Hebron; IC, immunocompromising condition; ICU, intensive care unit; IQR, interquartile range; n, number; N, number of SARI patients; SARI, severe acute respiratory infection; SARS-CoV-2, severe acute respiratory syndrome coronavirus 2; SD, standard deviation; UZA, Universitair Ziekenhuis Antwerpen. A square bracket “[“ and “]” in a range denotes that the number is included in the range, while regular brackets “(“ and “)” in a range means that the number is not included

### Prevalence of ICs among Hospitalized SARI Patients

**Fig. 1:**
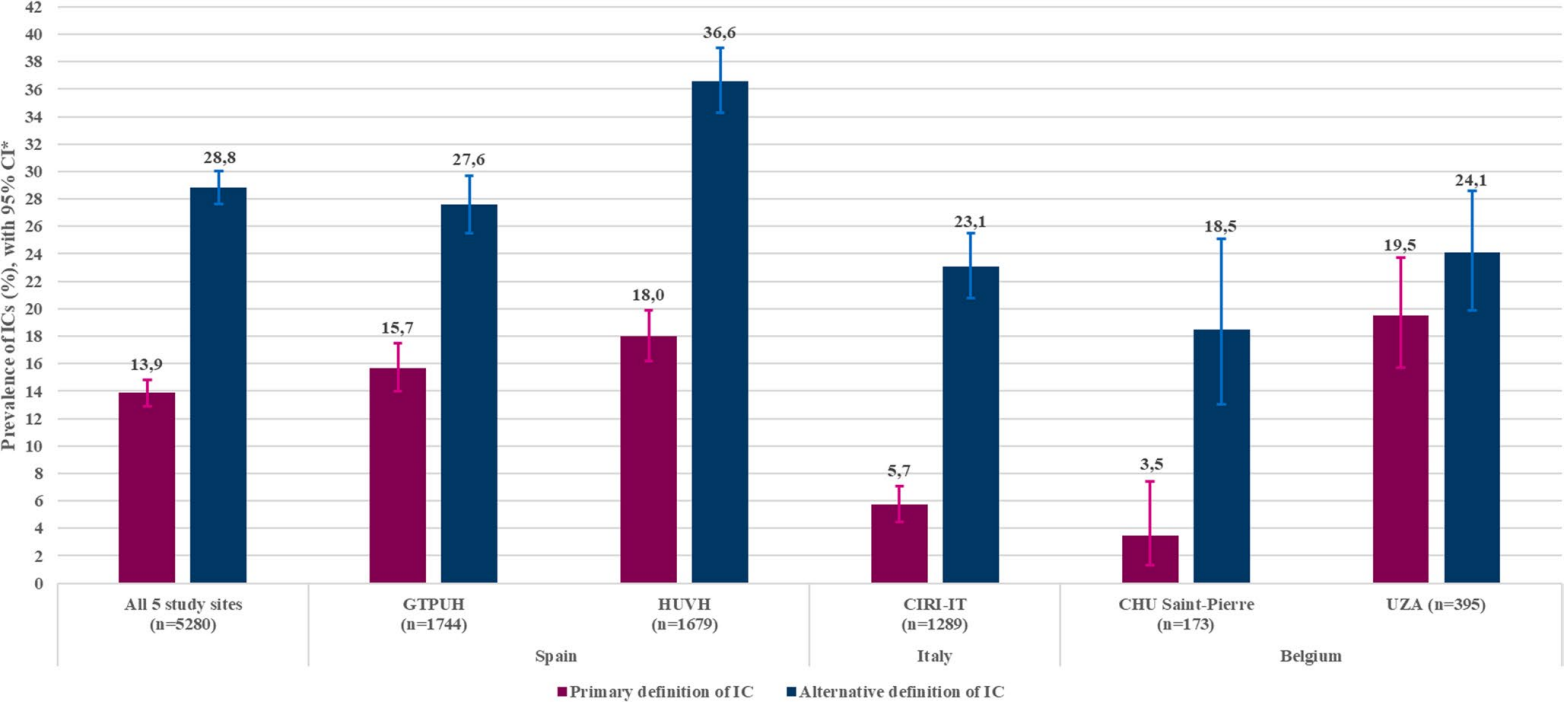
IC prevalence among hospitalized SARI patients, overall and by study site. Abbreviations: CHU Saint-Pierre, Centre Hospitalier Universitaire Saint-Pierre; CI, confidence interval; CIRI-IT, Centro Interuniversitario di Ricerca sull'Influenza e le altre Infezioni Trasmissibili; GTPUH, Hospital Universitario Germans Trias i Pujol; HUVH, Hospital Universitari Vall d’Hebron; IC, immunocompromising condition; n, number of SARI patients; UZA, Universitair Ziekenhuis Antwerpen

**Fig. 2:**
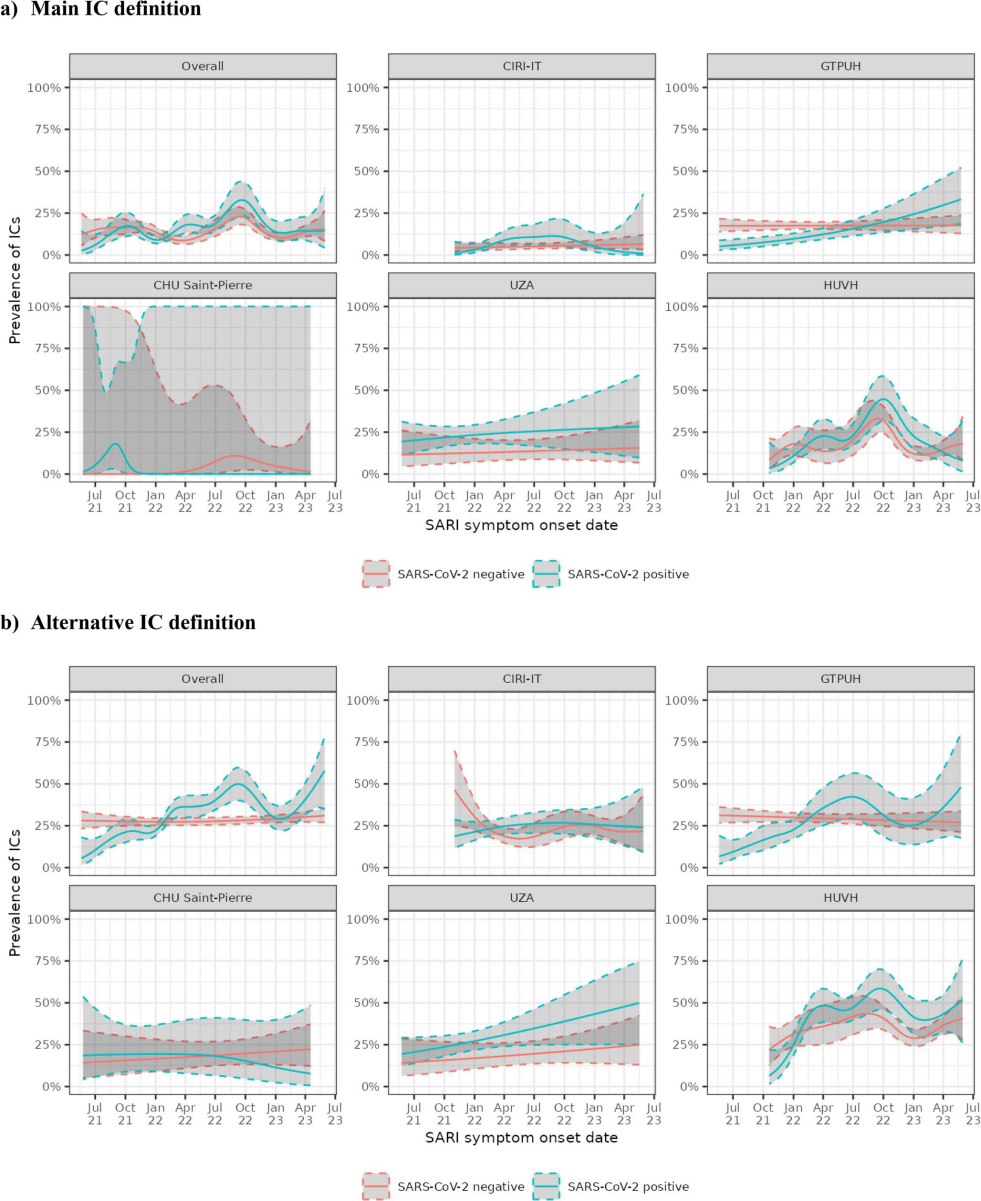
IC prevalence over time among hospitalized SARI, by SARS-CoV-2 test status: overall and by study site. (**a**) Main IC definition. (**b**) Alternative IC definition. Abbreviations: CHU Saint-Pierre, Centre Hospitalier Universitaire Saint-Pierre; CI, confidence interval; CIRI-IT, Centro Interuniversitario di Ricerca sull'Influenza e le altre Infezioni Trasmissibili; GTPUH, Hospital Universitario Germans Trias i Pujol; HUVH, Hospital Universitari Vall d’Hebron; IC, immunocompromising condition; n, number of SARI patients; SARI, severe acute respiratory infection; SARS-CoV-2, severe acute respiratory syndrome coronavirus 2; UZA, Universitair Ziekenhuis Antwerpen

Among the 5280 SARI-hospitalized patients, the overall IC prevalence based on the main definition was 13.9% (95% CI: 12.9–14.8) (Fig. 1; Table 4); increasing from 8.3% (95% CI: 3.7–15.8) at the start of the study to a peak of 22.0% (95% CI: 17.9–26.4) in Q3 2022, declining thereafter to 11.7% in Q1 2023 (Fig. 2a).

**Table 4:** Prevalence of IC patients among hospitalized SARI, overall, by SARS-CoV-2 test status and calendar time

IC definitions ^a^	Time period ^b^		All individuals hospitalized for SARI ^c^				SARS–CoV–2 test status of individuals hospitalized for SARI ^d^							
							Positive				Negative			
					Prevalence				Prevalence				Prevalence	
			*n*	(*N*)	%	95% CI	*n*	(*N*)	%	95% CI	*n*	(*N*)	%	95% CI
Main IC definition	**Overall** ^**b**^	01 Jun 2021 – 31 May 2023	732	(5280)	13.9	12.9 – 14.8	274	(1924)	14.2	12.7 – 15.9	458	(3356)	13.6	12.5 – 14.9
	**Q2 2021**†	01 Jun 2021 – 30 Jun 2021	8	(96)	8.3	3.7 – 15.8	1	(29)	3.4	0.1 – 17.8	7	(67)	10.4	4.3 – 20.3
	**Q3 2021**	01 Jul 2021 – 30 Sep 2021	52	(378)	13.8	10.4 – 17.6	18	(186)	9.7	5.8 – 14.9	34	(192)	17.7	12.6 – 23.9
	**Q4 2021**	01 Oct 2021 – 31 Dec 2021	149	(1017)	14.7	12.5 – 17.0	50	(391)	12.8	9.6 – 16.5	99	(626)	15.8	13 – 18.9
	**Q1 2022**	01 Jan 2022 – 31 Mar 2022	111	(1112)	10.0	8.3 – 11.9	55	(523)	10.5	8.0 – 13.5	56	(589)	9.5	7.3 – 12.2
	**Q2 2022**	01 Apr 2021 – 30 Jun 2021	83	(538)	15.4	12.5 – 18.8	53	(293)	18.1	13.9 – 23.0	30	(245)	12.2	8.4 – 17.0
	**Q3 2022**	01 Jul 2022 – 30 Sep 2022	85	(387)	22.0	17.9 – 26.4	38	(160)	23.8	17.4 – 31.1	47	(227)	20.7	15.6 – 26.6
	**Q4 2022**	01 Oct 2022 – 31 Dec 2022	120	(764)	15.7	13.2 – 18.5	36	(179)	20.1	14.5 – 26.7	84	(585)	14.4	11.6 – 17.5
	**Q1 2023**	01 Jan 2023 – 31 Mar 2023	83	(710)	11.7	9.4 – 14.3	18	(116)	15.5	9.5 – 23.4	65	(594)	10.9	8.5 – 13.7
	**Q2 2023**†	01 Apr 2023 – 31 May 2023	41	(278)	14.7	10.8 – 19.5	5	(47)	10.6	3.5 – 23.1	36	(231)	15.6	11.2 – 20.9
Alternative IC definition ^a^	**Overall** ^**b**^	01 Jun 2021 – 31 May 2023	1521	(5280)	28.8	27.6 – 30.0	570	(1924)	29.6	27.6 – 31.7	951	(3356)	28.3	26.8 – 29.9
	**Q2 2021**†	01 Jun 2021 – 30 Jun 2021	18	(96)	18.8	11.5 – 28.0	1	(29)	3.4	0.1 – 17.8	17	(67)	25.4	15.5 – 37.5
	**Q3 2021**	01 Jul 2021 – 30 Sep 2021	88	(378)	23.3	19.1 – 27.9	28	(186)	15.1	10.2 – 21.0	60	(192)	31.2	24.8 – 38.3
	**Q4 2021**	01 Oct 2021 – 31 Dec 2021	258	(1017)	25.4	22.7 – 28.2	85	(391)	21.7	17.8 – 26.2	173	(626)	27.6	24.2 – 31.3
	**Q1 2022**	01 Jan 2022 – 31 Mar 2022	301	(1112)	27.1	24.5 – 29.8	149	(523)	28.5	24.7 – 32.6	152	(589)	25.8	22.3 – 29.5
	**Q2 2022**	01 Apr 2021 – 30 Jun 2021	177	(538)	32.9	28.9 – 37.0	112	(293)	38.2	32.6 – 44.1	65	(245)	26.5	21.1 – 32.5
	**Q3 2022**	01 Jul 2022 – 30 Sep 2022	143	(387)	37.0	32.1 – 42.0	71	(160)	44.4	36.5 – 52.4	72	(227)	31.7	25.7 – 38.2
	**Q4 2022**	01 Oct 2022 – 31 Dec 2022	225	(764)	29.5	26.2 – 32.8	63	(179)	35.2	28.2 – 42.7	162	(585)	27.7	24.1 – 31.5
	**Q1 2023**	01 Jan 2023 – 31 Mar 2023	204	(710)	28.7	25.4 – 32.2	39	(116)	33.6	25.1 – 43.0	165	(594)	27.8	24.2 – 31.6
	**Q2 2023**†	01 Apr 2023 – 31 May 2023	107	(278)	38.5	32.7 – 44.5	22	(47)	46.8	32.1 – 61.9	85	(231)	36.8	30.6 – 43.4

^a^The alternative IC definition is IC by main definition and/or cancer (Table 1); ^b^ Overall, entire study period 01 June 2021–31 May 2023, quarters based on the symptom onset date; Q, calendar time in fiscal quarter; †, this fiscal quarter included one out of three months; ^c^ Main SARI definition (Table 1); ^d^ Main SARS-CoV-2 test status definition (Table 1). Abbreviations: *CI* confidence interval, *IC* immunocompromising condition, *n* number of IC individuals, *N* number of SARI patients, *SARI* severe acute respiratory infection

Using the broader alternative definition that included cancer patients, overall IC prevalence was 28.8% (95% CI: 27.6–30.0), increasing from 18.8% (95% CI: 11.5–28.0) at the start of study to a peak of 38.5% (CI 32.7–44.5) in Q2 2023 (Table 4; Figs. 1 and 2b). Estimates varied substantially by study site (Fig. 1; Table 5, Table S3), with overall IC prevalence ranging from 3.5% (CHU Saint-Pierre) to 19.5% (UZA) using the main definition, and 18.5% (CHU Saint-Pierre) to 36.6% (HUVH) using the alternative definition (Fig. 1; Table 5, Table S3). Figure 2 and Table S3 present the IC prevalence over time for each study site separately for both the main and the alternative IC definitions.

**Table 5:** IC prevalence among hospitalized SARI patients in three European countries (June 2021-May 2023), by study site

Country	Study site	All SARI ^a^	Main IC definition ^b^			Alternative IC definition ^b^		
				Prevalence			Prevalence	
		*N*	*n*	%	95% CI	*n*	%	95% CI
Spain	GTPUH	1744	274	15.7	14.0–17.5	481	27.6	25.5–29.7
	HUVH	1679	302	18.0	16.2–19.9	615	36.6	34.3–39.0
Italy	CIRI-IT	1289	73	5.7	4.50–7.1	298	23.1	20.8–25.5
Belgium	UZA	395	77	19.5	15.70–23.7	95	24.1	19.9–28.6
	CHU Saint-Pierre	173	6	3.5	1.30–7.4	32	18.5	13.0–25.1

^a^ Main SARI definition (Table 1); ^b^ The alternative IC definition is IC by main definition and/or cancer (Table 1). Abbreviations: CHU Saint-Pierre, Centre Hospitalier Universitaire Saint-Pierre; CI, confidence interval; CIRI-IT, Centro Interuniversitario di Ricerca sull’Influenza e le altre Infezioni Trasmissibili; GTPUH, Hospital Universitario Germans Trias i Pujol; HUVH, Hospital Universitari Vall d’Hebron; IC, immunocompromising condition, number; N, number of SARI patients; SARI, severe acute respiratory infection; UZA, Universitair Ziekenhuis Antwerpen

Among the 1924 SARS-CoV-2-positive SARI patients, the overall IC prevalence based on the main definition was 14.2% (95% CI: 12.7–15.9), increasing from 3.4% (95% CI: 0.1–17.8) to a peak of 23.8% (95% CI: 17.4–31.1) in Q3 2022, thereafter declining to 10.6% by Q2 2023 (Table 4). Using the alternative definition, IC prevalence among all SARI was 29.6% (95% CI: 27.6–31.7), increasing from 3.4% (95% CI: 0.1–17.8) to a peak of 46.8% (95% CI: 32.1–61.9) in Q2 2023. In contrast, among the 3356 SARS-CoV-2-negative SARI patients, IC prevalence did not have any consistent pattern over time, fluctuating between 9.5% and 20.7% for the main IC definition and 25.4% to 36.8% for the alternative IC definition (Table 4).

**Fig. 3:**
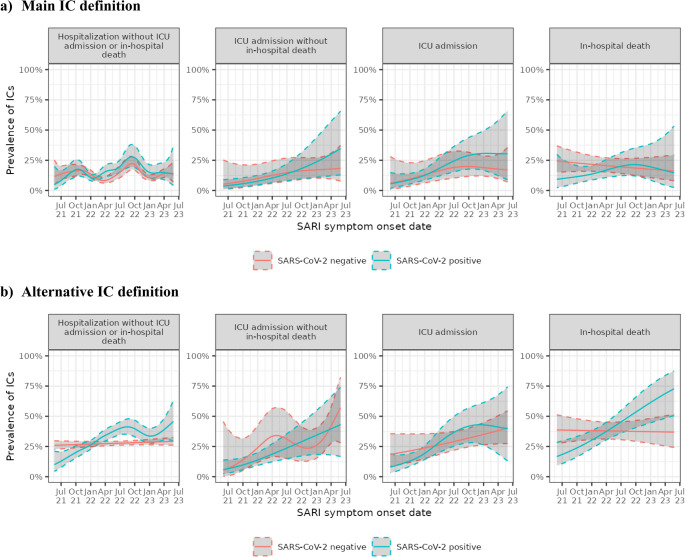
IC prevalence over time among hospitalized SARI, by SARS-CoV-2 test status and level of SARI severity (**a**) Main IC definition. (**b**) Alternative IC definition. Abbreviations: CHU Saint-Pierre, Centre Hospitalier Universitaire Saint-Pierre; CI, confidence interval; CIRI-IT, Centro Interuniversitario di Ricerca sull'Influenza e le altre Infezioni Trasmissibili; GTPUH, Hospital Universitario Germans Trias i Pujol; HUVH, Hospital Universitari Vall d’Hebron; IC, immunocompromising condition; n, number of SARI patients; SARI, severe acute respiratory infection; SARS-CoV-2, severe acute respiratory syndrome coronavirus 2; UZA, Universitair Ziekenhuis Antwerpen

This difference in IC prevalence between SARS-CoV-2-positive and negative SARI patients over time was more evident when using the alternative IC definition (Fig. 2b; Table 4), and was especially pronounced among study participants who died in the hospital (Fig. 3b, Table S4, and Table S5).

The analysis for linear trend confirmed the observed increases in IC prevalence over time among SARS-CoV-2-positives, with a greater increase than among SARS-CoV-2-negatives (the main IC definition, coefficient, 0.00014, 95% CI: 0.00012–0.00016, *P* < 0.001 for the interaction term of symptom onset date and SARS-CoV-2 test status). It also confirmed that the coefficient associated with the trend term was higher. The difference between results using different IC definitions was most pronounced at CHU Saint-Pierre (Fig. 1). Among the five study sites, CHU Saint-Pierre served the smallest region (Brussels city center and south, Table S1), and showed relatively more males and younger patients compared with other sites (Table S10).

The alternative definition of IC showed less fluctuation in IC prevalence over time for negative cases, when defining SARI patients by both the main and the alternative (WHO) definitions as used in sensitivity analyses, compared to the main IC definition (Figure S1a and S1b). However, for the WHO definition of SARI, which requires fever AND cough, less fluctuation in IC prevalence over time was seen also for positive cases (Figure S1b, Table S6).

### Demographic Characteristics of Hospitalized SARI Patients

The majority of the SARI study population was elderly (median age 71.0 years), male (56.5%), and recruited from Spain (64.8%) as shown in Table 3. Using the main IC definition, IC individuals were younger than non-IC (median age 65.0 v. 72.0 years, respectively) and less likely to reside in an LTCF (1.9% v. 4.2%, respectively). They were also less likely to be unvaccinated against COVID-19 at the time of SARI hospitalization (6.8% v. 13.0%, respectively) and had a slightly longer median time since last vaccine dose (165 v. 155 days, respectively) (Table 3). Over time (from Q2 2021 to Q2 2023), the percent unvaccinated decreased from 25% (2/8 SARI cases) to 2.4% (1/41 SARI cases) among IC patients (main definition) and from 33.0% (29/88 SARI cases) to 10.5% (25/237 SARI cases) among non-IC (Table S8**)**. Similarly, using the alternative IC definition, which included patients with cancers, the percent unvaccinated decreased over time from 11.1% to 3.7% among IC patients, and from 37.2% to 12.9% among non-IC patients (Table S9).

Stratifying by SARS-CoV-2 test status, most demographic results remained similar; however, differences in vaccination status between IC patients (by main definition) and non-IC were more pronounced among SARS-CoV-2 positives. In this group, only 9.1% of IC were unvaccinated, compared with 23.8% of non-IC (Table 3). Conversely, among SARS-CoV-2 negatives, the percentage unvaccinated was similar (5.5% for IC and 6.9% for non-IC).

Results were similar when the alternative IC definition was used (Table S7).

### Clinical Characteristics of Hospitalized SARI Patients

The majority (67.3%) of SARI cases reported two or more SARI symptoms. Cough (71.4%) and shortness of breath (70.1%) were the most frequent, followed by fever (62%). Fever was slightly more common among IC patients than non-IC (64.2% v. 61.8%, respectively), whereas cough and shortness of breath were less common (65.0% v. 72.5%, respectively, for cough; 63.4% v. 71.2% respectively, for shortness of breath) (Table 3). Anosmia, ageusia or dysgeusia were reported for only 3.6% of SARI cases, with 9.0% of SARS-CoV-2-positive cases reporting these symptoms versus 1.0% of SARS-CoV-2 negatives. Among SARS-CoV-2 positives, anosmia, ageusia or dysgeusia were less likely to be reported among IC (6.5%) than non-IC (9.3%).

The overall mean length of hospital stay for SARI was 10.1 days; one day higher in IC versus non-IC patients (11.1 v. 10.0 days), a difference which was higher in case of SARS-CoV-2 positives (14.0 v. 12.0 days, respectively) compared with negatives (9.3 v. 8.9 days, respectively) as shown in Table 3.

Overall, 8.2% of SARI cases were admitted to the intensive care unit (ICU) and 7.2% experienced an in-hospital death, with estimates varying widely by study site, lowest at CIRI-IT in Italy, 1.6% and 2.6%, respectively, and the highest ICU admission of 14.4% at UZA in Belgium and the highest in-hospital death of 11.2% in GTPUH in Spain (Table S10, Table S11). Compared to SARS-CoV-2-negative cases, SARS-CoV-2 positives were substantially more likely to be admitted to the ICU (14.9% v. 4.4%, respectively) and to experience in-hospital death (9.9% v. 5.7%, respectively) as presented in Table 3.

Comparing IC with non-IC SARI patients as per the main IC definition, the percent admitted to the ICU was similar (8.6% and 8.2%, respectively). However, in-hospital deaths occurred more frequently among IC patients than non-IC (9.3% v. 6.9%, respectively), with the percent of in-hospital death being more similar among SARS-CoV-2 positives (10.6% for IC patients v. 9.8% non-IC) than among SARS-CoV-2-negatives (8.5% for IC patients v. 5.2% for non-IC) (Table 3). Similar results for in-hospital death were observed when comparing the alternative IC definition with the main IC definition, but the percentage admitted to the ICU was slightly lower for IC patients than non-IC (6.8% v. 8.8%, respectively) (Table S7).

### Prevalence of Comorbidities in Hospitalized SARI Patients

Overall, most SARI cases (81.7%) had at least one non-IC comorbidity and more than a third (37.2%) had at least three (Table 3). Using the main IC definition, co-morbidities (other than IC) were more common in IC than non-IC patients, with 91.3% v. 80.1%, respectively, having at least one co-morbidity and 42.2% v. 36.4%, having at least three. Using the alternative IC definition, differences were more pronounced, with 95.8% v. 76.0%, respectively, having at least one co-morbidity and 51.0% v. 31.6%, respectively, having at least three (Table S7).

Among IC patients, using the main definition, cancer was the most common co-morbidity (52.3%) followed by hypertension (43.4%), lung disease (38.7%) and cardiovascular disease (34.2%). Among non-IC, hypertension was the most common (50.7%) followed by cardiovascular discussionsease (40.8%), lung disease (32.0%), and type 2 diabetes (26.7%); only 17.3% had cancer. Among study participants with multiple comorbidities, the most prevalent was a combination of cardiovascular disease and hypertension (Figure S2). Results were similar when stratified by SARS-CoV-2 test status.

## Discussion

Across three European countries, this study demonstrates that throughout the COVID-19 pandemic, IC individuals represented a high percentage of hospitalized SARI cases that persisted until the transition into endemicity, overall accounting for 13.9% of cases when using a relatively strict main IC definition and 28.8% of cases when using a broader alternative definition including cancer patients. This compares to an expected prevalence in the European and US general population of 1–7%, as estimated in studies using various IC definitions and methods for ascertaining prevalence [3, 5, 23, 24]. In particular, the ongoing INFORM study, which has used National Health Service (NHS) data to analyze a random 25% sample of the entire population of England aged 12 years and older, found an IC prevalence of 3.9% using a similarly broad definition that included cancer patients and additionally included those with end stage-kidney disease [23]. Thus, taken together, these findings suggest that IC patients bear a highly disproportionate burden of SARI in hospitals.

Further, consistent with our hypothesis, IC prevalence among SARS-CoV-2-positive SARI cases appeared to increase from the earlier to later periods of the pandemic, peaking at greater than 20% by the end of 2022 for the stricter IC definition and nearly 50% for the broader IC definition by mid-2023. Although patterns over time were not always consistent and there was variability by study site, this roughly corresponded to increasing background rates of COVID-19 vaccination and SARS-CoV-2 exposure in the general population over time as well as relaxing of non-pharmaceutical interventions to prevent infections (e.g., shielding or physical distancing) and increased availability of antiviral treatments, which are often contraindicated for those with certain ICs. Indeed, stratifying by SARS-CoV-2 test status, a linear trend analysis confirmed that the observed increases in IC prevalence over time were significantly greater for SARS-CoV-2-positive SARI patients than SARS-CoV-2-negative patients.

The disproportionately high proportion of IC among hospitalized SARI and COVID-19 patients is in fact not surprising given that IC patients are known to have an increased risk of severe respiratory infection [23, 25]. For example, the INFORM study has found that, after adjusting for sex, age, and number of co-morbidities, compared to non-IC, IC individuals have a 2-fold increased risk of COVID-19 hospitalization and death, with certain ICs such as solid organ or stem cell transplantation and recently-treated hematologic cancer being associated with greater than 10-fold increases in risk. Further, when analyses were limited to those who had been vaccinated with at least three doses of a COVID-19 vaccine, IC individuals remained at elevated risk [23]. An analysis of nationwide US claims data found that IC individuals were 4 times more likely to be hospitalized for SARI than non-IC [5].

In our study, IC, hypertension and chronic kidney disease tended to be more commonly observed in SARS-CoV-2-positive study participants, while other comorbidities, such as cardiovascular and respiratory comorbidities (common comorbidities in the general adult population), tended to be more frequent in SARS-CoV-2–negative study participants. In-hospital death was higher in IC patients than non-IC individuals, regardless of SARS-CoV-2 test status and IC definition used.

Although definitions of individuals with ICs vary across studies, making direct comparisons challenging, individuals with ICs generally have a poorer response to vaccination against respiratory infections compared to individuals without ICs. Data from over 89,000 adults aged 18 years and older, hospitalized with COVID-19-like illness in the United States, showed vaccine effectiveness against COVID-19-associated hospitalization of 77% in individuals with ICs, compared with 90% in individuals without ICs [26]. Approximately one in five individuals with ICs (recipients of solid organ transplants, people with rare autoimmune diseases, and individuals with lymphoid malignancies) have no serological responses to COVID-19 vaccines, even after three or more vaccine doses; the immunosuppressant administered and underlying disease type are strongly associated with the serological response [27]. 13- and 23-valent pneumococcal conjugate vaccination of IC individuals generally does not confer significant effectiveness against pneumococcal disease and pneumococcal pneumonia [28]. Similarly, the influenza vaccine effectiveness against influenza-related hospitalization in IC individuals has been notably low, e.g., 5% versus 41% in non-IC during the 2017–2018 influenza season in the United States [29]. For respiratory syncytial virus (RSV), a US study of > 36,000 adults aged ≥ 60 years, estimated vaccine effectiveness against hospitalization due to RSV-like illness during the first season of vaccine roll-out at 73% in IC individuals compared with 80% in non-IC, although further assessment is needed given the broadness of the IC definition that was used [30].

Thus, although IC individuals are more vulnerable to respiratory infections than the general population, they often have limited preventive and treatment options and, furthermore, have traditionally been largely excluded from clinical trials of vaccines, monoclonal antibodies, and antivirals [31]. Other non-pharmaceutical preventive measures such as shielding may come with high personal costs that can affect both quality of life and social equity [32]. Pre-exposure prophylaxis with long-acting monoclonal antibodies (i.e., passive immunization) is a potentially important alternative to vaccines for IC individuals, with the notable success of such therapies for the prevention of RSV infection in young infants [33]. However, the development of such therapies has also proven challenging in the rapidly evolving SARS-CoV-2 variant landscape [34, 35].

Strengths of this secondary data use study include the COVIDRIVE platform, which offers high quality testing data across a large dataset, enabling IC and other comorbidities to be effectively captured. As a multi-center and multi-country study, the findings provide insights from three European countries using consistent definitions for both SARI and IC, enhancing international generalizability.

Limitations of this study include inability to capture the sub-categories of IC within the COVIDRIVE dataset, which prevented more granular estimates on the types of IC. The study may also include incidental SARI cases, i.e., patients for which the primary reason for hospitalization was a condition unrelated to SARI but the patient met the inclusion criteria (e.g., a patient hospitalized for an orthopedic injury who also had cough and fever within five days prior to admission and as such was defined as SARI patient). Misclassification of SARS-CoV-2 test status was also theoretically possible, as antigen test results were not collected, while it could happen that hospitalization and thus PCR testing occurred too late for the virus to be detected and gave a false-negative PCR result. However, we expect this to have happened rarely in our population of symptomatic hospitalized SARI patients. Additionally, because COVIDRIVE primarily focused on effectiveness of COVID-19 vaccines, data on other viral or bacterial pathogens as potential causes of SARI were not captured. A considerable degree of heterogeneity was observed between study sites, though this could not be explained by study site characteristics. Due to varying data collection time points across study sites, comparisons were challenging. ICU admission included both severe patients directly admitted to the ICU and those that were initially admitted to the ward but progressed to the ICU care. Finally, the results are specific to adult populations with similar healthcare behaviors and access to medical care as the study participants.

## Conclusion

This study highlights a notably elevated prevalence of ICs and significant comorbidities among individuals hospitalized with SARI in Europe. The disproportionately high prevalence of IC compared with what would be expected in the general population, underscores the critical need for targeted interventions. These interventions should aim to mitigate risk and enhance health outcomes for this vulnerable group. Since IC individuals are more susceptible to severe infection and often exhibit suboptimal responses to vaccines, it is vital to consider additional protective measures.

## Supplementary Information

Below is the link to the electronic supplementary material.

Supplementary Material 1 (DOCX 3.43 MB)

## Data Availability

De-identified data that underlie the results reported here may be obtained in accordance with the id.DRIVE data access policy.
