# Supplementary material for: Changing Prevalence of Immunocompromising Conditions in Patients Hospitalized with Severe Acute Respiratory Infection in Europe: Insights from the COVIDRIVE Study
Source: J Epidemiol Glob Health. 2026 Mar 9;16(1):32. doi: 10.1007/s44197-025-00503-w (PMC12976257; doi:10.1007/s44197-025-00503-w)
Supplement: Supplementary file 1 — Supplementary Material 1 (DOCX 3.43 MB) [file 44197_2025_503_MOESM1_ESM.docx]

**Prevalence of immunocompromising and other chronic conditions in patients hospitalized with severe acute respiratory infection in Europe: results from the id.DRIVE study**

**Supplementary material**

[Table S1. Characteristics of study sites included in secondary COVIDRIVE data analysis 2](#_Toc207910897)

[Table S2. ICD-10 codes for chronic comorbidities included in secondary COVIDRIVE data analysis of hospitalized SARI patients in three European countries 3](#_Toc207910898)

[Table S3. IC prevalence among hospitalized SARI patients, overall, by SARS-CoV-2 test status and by calendar time, per study site 4](#_Toc207910899)

[Table S4. IC prevalence (main definition) among hospitalized SARI patients, for subgroups defined by in-hospital outcome severity levels, by SARS-CoV-2 test status, and by calendar time 9](#_Toc207910900)

[Table S5. IC prevalence (alternative definition) among hospitalized SARI patients, for subgroups defined by in-hospital outcome severity levels, by SARS-CoV-2 test status, and by calendar time 11](#_Toc207910901)

[Table S6. IC prevalence among hospitalized SARI patients (alternative WHO definition), overall by SARS-CoV-2 test status, and by calendar time 13](#_Toc207910902)

[Table S7. Demographics, clinical outcomes of study population, overall, by IC status (alternative definition), and by SARS-CoV-2 test status 14](#_Toc207910903)

[Table S8. Demographic and clinical characteristics of hospitalized SARI patients in three European countries, by SARS-CoV-2 test status, by IC status (main definition), and by calendar time 18](#_Toc207910904)

[Table S9. Demographic and clinical characteristics of hospitalized SARI patients in three European countries, by SARS-CoV-2 test status, by IC status (alternative definition), and by calendar time 55](#_Toc207910905)

[Table S10. Demographic and clinical characteristics of hospitalized SARI patients in three European countries, by study site, and by IC status (main definition) 88](#_Toc207910906)

[Table S11. Demographic and clinical characteristics of hospitalized SARI patients in three European countries, by study site, and by IC status (alternative definition) 94](#_Toc207910907)

[Figure S1. IC prevalence over time among hospitalized SARI, by SARS-CoV-2 test status, comparing different SARI and SARS-CoV-2 test status definitions 100](#_Toc207910908)

[Figure S2. Most frequent (25) combinations of chronic conditions in hospitalized SARI patients, by SARS-CoV-2 test status 101](#_Toc207910909)

Table S1. Characteristics of study sites included in secondary COVIDRIVE data analysis

| **Country,**  **study site,**  ***city*** | **No. of hospitals*,* public/**  **private** | **Type of care** | **Specialization** | **No. of all beds** | **No. of ICU beds** | **Catchment population of the hospital/ network (n)** | **Catchment population relative to total population of region (%)** | **Date of recruitment start** | **Date of recruitment end** |
| --- | --- | --- | --- | --- | --- | --- | --- | --- | --- |
| **Belgium** |  |  |  |  |  |  |  |  |  |
| UZA,  *Antwerp* | 1,  public | Tertiary | General hospital | 605 | 45 | 1,800,000 | 100% of Antwerp province inhabitants and surroundings | 01 Jun 2021 | 18 May 2023 |
| CHU Saint-Pierre,  *Brussels* | 1,  public | Tertiary | General hospital which is a HIV reference hospital | 582 | 30 | 100,000 | All South and Center of Brussels inhabitants | 01 Jun 2021 | 17 Apr 2023 |
| **Italy** |  |  |  |  |  |  |  |  |  |
| CIRI-IT, | 4, |  |  |  |  |  |  | 16 Nov 2021 | 30 May 2023 |
| *Genoa* | public | Tertiary | General hospital with specific focus on hematology, oncology and neuroscience | 1,200 | 51 | 700,000 | 55% of all hospital admissions in Genoa metropolitan area and central Liguria |  |  |
| *Milan* | public | Tertiary | General hospital, home to Nord Italia Transplant program (NITp), the regional centre of reference for the treatment of rare diseases | 716 | 55 | 1,350,000 | 100% all Lombardy region inhabitants |  |  |
| *Rome* | public | Tertiary | General hospital | 450 | 26 | 4,356,000 | 100%, all Rome region inhabitants |  |  |
| *Bari* | public | Tertiary | General hospital which is a Regional Transplant Center, main surgery hospital and oncology orientation center | 1,550 | 27 | 1,262,000 | 100% all Puglia region inhabitants |  |  |
| **Spain** |  |  |  |  |  |  |  |  |  |
| GTPUH, *Badalona* | 1,  public | Tertiary | General hospital | 794 | 94 | 800,000 | 100% All Badalona inhabitants and surroundings | 01 Jun 2021 | 26 May 2023 |
| HUVH,  *Barcelona* | 1, public | Tertiary | General hospital | 1,154 | 56 | 430,000 (primary, secondary >2 M) | NA | 30 Oct 2021 | 31 May 2023 |

Abbreviations: CHU Saint-Pierre, Centre Hospitalier Universitaire Saint-Pierre; CIRI-IT, Centro Interuniversitario di Ricerca sull'Influenza e le altre Infezioni Trasmissibili; GTPUH, Hospital Universitario Germans Trias i Pujol; HUVH, Hospital Universitari Vall d’Hebron; ICU, intensive care unit; n, number of SARI patients; M, million; NA, not applicable; No., number; UZA, Universitair Ziekenhuis Antwerpen.

Table S2. ICD-10 codes for chronic comorbidities included in secondary COVIDRIVE data analysis of hospitalized SARI patients in three European countries

| **Chronic condition** | **ICD-10 codes** | **Including** | **Excluding** |
| --- | --- | --- | --- |
| **Asthma** | J45, J46 | predominantly allergic asthma, nonallergic asthma, status asthmaticus, acute severe asthma | acute severe asthma, chronic asthmatic (obstructive) bronchitis, chronic obstructive asthma, eosinophilic asthma, lung diseases due to external agents. |
| **Lung disease** | A15-16, A19, A31.0, B33.4, E84.0, J40-44, J47, J60-70, J80-84, J85-86, J90-91, J92.9, J93-94, J95-99 | tuberculosis (pulmonary, miliary but not that of other systems), atypical mycobacteria, cystic fibrosis, COPD, bronchiectasis and other chronic sequelae of infections, chronic lung diseases due to external agents, interstitial lung diseases, pleural diseases, respiratory failure. | acute respiratory infections, lung cancer, diseases of pulmonary circulation, pleural plaques without asbestos, previous uncomplicated pneumothorax. |
| **Cardio-vascular disease** | A52.0, B37.6, I01-02, I05-09, I11.0, I13.0, I13.2, I20-25, I26-28, I30-43, I44- 46, I48, I49.0, I49.5, I50-52, I70-71, Q20-Q28 | all conditions of heart & large vessels that are chronic or likely to have chronic sequelae. Cardiovascular syphilis, endo-, myo- and pericarditis, rheumatic fever, chronic rheumatic heart diseases, congenital malformations, hypertensive (renal) diseases with heart failure, ischemic heart diseases, diseases of pulmonary circulation, atherosclerosis, cardiomyopathies, most conduction disorders, heart failure, aortic aneurysms & dissection, other heart diseases and their complications. | uncomplicated hypertension, previous uncomplicated pulmonary embolism (with no lasting cardiac insufficiency), paroxysmal tachycardias, most cases of premature depolarization. |
| **Hyper-tension** | I10, I11.9, I12, I13.1, I13.9, I15 | essential (primary) hypertension, secondary hypertension | hypertensive heart/renal disease with (congestive) heart failure |
| **Chronic liver disease** | B18, B19, K70.1-4, K70.9, K71.1, K71.3, K71.4, K71.6-9, K72.1, K72.9, K73, K74, K75.2, K75.4, K75.8, K75.9, K76.1, K76.2, K76.5, K76.6, K76.9 | all conditions of the liver that are chronic or likely to have chronic sequelae | acute liver disease, liver cancer / metastasis, liver transplant |
| **Chronic kidney disease** | I12-13, M10.30, N00-19, N20.0, N25-27, N28.0, N28.9, Q63.9, Z90.5 |  | clinically nonsignificant kidney cysts |
| **Type 2 diabetes** | E11 | non-insulin dependent diabetes mellitus (adult-onset, maturity-onset, nonketotic, stable, type II, non-insulin-dependent diabetes of the young) |  |
| **Cancer** | C00-97, D37-48, Z85, Z92.3, Z92.6. | all malignant neoplasms (both solid and hematologic) with potential to metastasize, either in treatment, active follow-up, or <5 years post curative treatment. | benign & in situ neoplasms. Basal cell carcinomas. Any cancer previously treated with curative intent & in complete remission for ≥5 years. |

Abbreviations: COPD, chronic obstructive pulmonary disease; ICD, international classification of diseases, SARI, severe acute respiratory infection

Table S3. IC prevalence among hospitalized SARI patients, overall, by SARS-CoV-2 test status and by calendar time, per study site

**Panel A. Study site CIRI-IT (CIRI-IT, Centro Interuniversitario di Ricerca sull'Influenza e le altre Infezioni Trasmissibili), Italy**

| **IC  definition ^a^** | **Period ^b^** | | | | **All SARI ^c^** | | | | **SARS-CoV-2 test status ^d^** | | | | | | | |
| --- | --- | --- | --- | --- | --- | --- | --- | --- | --- | --- | --- | --- | --- | --- | --- | --- |
|  |  |  |  |  |  |  |  |  | **Positive** | | | | **Negative** | | | |
|  |  |  |  |  |  | | **Prevalence** | |  | | **Prevalence** | |  | | **Prevalence** | |
|  |  |  |  |  | **n** | **(N)** | **%** | **95% CI** | **n** | **(N)** | **%** | **95% CI** | **n** | **(N)** | **%** | **95% CI** |
| **Main  definition** | **Overall** |  |  |  | 73 | (1,289) | 5.7 | 4.5 - 7.1 | 31 | (481) | 6.4 | 4.4 - 9.0 | 42 | (808) | 5.2 | 3.8 - 7.0 |
|  | **Q2 2021**† | 01 June 2021 | – | 30 Jun 2021 | 0 | (0) | NA | NA | 0 | (0) | NA | NA | 0 | (0) | NA | NA |
|  | **Q3 2021** | 01 Jul 2021 | – | 30 Sep 2021 | 0 | (0) | NA | NA | 0 | (0) | NA | NA | 0 | (0) | NA | NA |
|  | **Q4 2021** | 01 Oct 2021 | – | 31 Dec 2021 | 5 | (87) | 5.7 | 1.9 - 12.9 | 2 | (57) | 3.5 | 0.4 - 12.1 | 3 | (30) | 10.0 | 2.1 - 26.5 |
|  | **Q1 2022** | 01 Jan 2022 | – | 31 Mar 2022 | 22 | (503) | 4.4 | 2.8 - 6.5 | 11 | (212) | 5.2 | 2.6 - 9.1 | 11 | (291) | 3.8 | 1.9 - 6.7 |
|  | **Q2 2022** | 01 Apr 2021 | – | 30 Jun 2021 | 15 | (212) | 7.1 | 4.0 - 11.4 | 9 | (80) | 11.2 | 5.3 - 20.3 | 6 | (132) | 4.5 | 1.7 - 9.6 |
|  | **Q3 2022** | 01 Jul 2022 | – | 30 Sep 2022 | 9 | (87) | 10.3 | 4.8 - 18.7 | 4 | (46) | 8.7 | 2.4 - 20.8 | 5 | (41) | 12.2 | 4.1 - 26.2 |
|  | **Q4 2022** | 01 Oct 2022 | – | 31 Dec 2022 | 9 | (170) | 5.3 | 2.4 - 9.8 | 4 | (49) | 8.2 | 2.3 - 19.6 | 5 | (121) | 4.1 | 1.4 - 9.4 |
|  | **Q1 2023** | 01 Jan 2023 | – | 31 Mar 2023 | 11 | (193) | 5.7 | 2.9 - 10.0 | 1 | (32) | 3.1 | 0.1 - 16.2 | 10 | (161) | 6.2 | 3.0 - 11.1 |
|  | **Q2 2023**† | 01 Apr 2023 | – | 31 May 2023 | 2 | (37) | 5.4 | 0.7 - 18.2 | 0 | (5) | 0.0 | 0.0 - 52.2 | 2 | (32) | 6.2 | 0.8 - 20.8 |
| **Alternative  definition** | **Overall** |  |  |  | 298 | (1,289) | 23.1 | 20.8 - 25.5 | 115 | (481) | 23.9 | 20.2 - 28.0 | 183 | (808) | 22.6 | 19.8 - 25.7 |
|  | **Q2 2021** | 01 June 2021 | – | 30 Jun 2021 | 0 | (0) | NA | NA | 0 | (0) | NA | NA | 0 | (0) | NA | NA |
|  | **Q3 2021** | 01 Jul 2021 | – | 30 Sep 2021 | 0 | (0) | NA | NA | 0 | (0) | NA | NA | 0 | (0) | NA | NA |
|  | **Q4 2021** | 01 Oct 2021 | – | 31 Dec 2021 | 24 | (87) | 27.6 | 18.5 - 38.2 | 11 | (57) | 19.3 | 10.0 - 31.9 | 13 | (30) | 43.3 | 25.5 - 62.6 |
|  | **Q1 2022** | 01 Jan 2022 | – | 31 Mar 2022 | 110 | (503) | 21.9 | 18.3 - 25.7 | 50 | (212) | 23.6 | 18.0 - 29.9 | 60 | (291) | 20.6 | 16.1 - 25.7 |
|  | **Q2 2022** | 01 Apr 2021 | – | 30 Jun 2021 | 45 | (212) | 21.2 | 15.9 - 27.4 | 21 | (80) | 26.2 | 17.0 - 37.3 | 24 | (132) | 18.2 | 12.0 - 25.8 |
|  | **Q3 2022** | 01 Jul 2022 | – | 30 Sep 2022 | 23 | (87) | 26.4 | 17.6 - 37.0 | 12 | (46) | 26.1 | 14.3 - 41.1 | 11 | (41) | 26.8 | 14.2 - 42.9 |
|  | **Q4 2022** | 01 Oct 2022 | – | 31 Dec 2022 | 44 | (170) | 25.9 | 19.5 - 33.1 | 13 | (49) | 26.5 | 14.9 - 41.1 | 31 | (121) | 25.6 | 18.1 - 34.4 |
|  | **Q1 2023** | 01 Jan 2023 | – | 31 Mar 2023 | 41 | (193) | 21.2 | 15.7 - 27.7 | 6 | (32) | 18.8 | 7.2 - 36.4 | 35 | (161) | 21.7 | 15.6 - 28.9 |
|  | **Q2 2023**† | 01 Apr 2023 | – | 31 May 2023 | 11 | (37) | 29.7 | 15.9 - 47.0 | 2 | (5) | 40.0 | 5.3 - 85.3 | 9 | (32) | 28.1 | 13.7 - 46.7 |

^a^ Main and alternative definition of IC. The alternative definition of IC was IC by main definition and/or cancer (Table 1); ^b^ Overall, entire study period 01 June 2021-31 May 2023; ^c^ Main SARI definition (Table 1); ^d^ Main SARS-CoV-2 test status definition (Table 1); †, this fiscal quarter included 1 out of 3 months. Abbreviations: CI, confidence interval; IC, immunocompromising condition; n, number of individuals with ICs; N, number of SARI patients; NA, not applicable; Q, calendar time in fiscal quarter; SARI, severe acute respiratory infection; SARS-CoV-2, severe acute respiratory syndrome coronavirus 2

**(Table S3 continued)**

**Panel B. Study site GTPUH (Hospital Universitario Germans Trias i Pujol), Spain**

| **IC  definition ^a^** | **Period ^b^** | | | | **All SARI ^c^** | | | | **SARS-CoV-2 test status ^d^** | | | | | | | |
| --- | --- | --- | --- | --- | --- | --- | --- | --- | --- | --- | --- | --- | --- | --- | --- | --- |
|  |  |  |  |  |  |  |  |  | **Positive** | | | | **Negative** | | | |
|  |  |  |  |  |  | | **Prevalence** | |  | | **Prevalence** | |  | | **Prevalence** | |
|  |  |  |  |  | **n** | **(N)** | **%** | **95% CI** | **n** | **(N)** | **%** | **95% CI** | **n** | **(N)** | **%** | **95% CI** |
| **Main  definition** | **Overall** |  |  |  | 274 | (1,744) | 15.7 | 14.0 - 17.5 | 49 | (459) | 10.7 | 8.0 - 13.9 | 225 | (1,285) | 17.5 | 15.5 - 19.7 |
|  | **Q2 2021**† | 01 June 2021 | – | 30 Jun 2021 | 8 | (80) | 10.0 | 4.4 - 18.8 | 1 | (22) | 4.5 | 0.1 - 22.8 | 7 | (58) | 12.1 | 5.0 - 23.3 |
|  | **Q3 2021** | 01 Jul 2021 | – | 30 Sep 2021 | 40 | (278) | 14.4 | 10.5 - 19.1 | 7 | (116) | 6.0 | 2.5 - 12 | 33 | (162) | 20.4 | 14.5 - 27.4 |
|  | **Q4 2021** | 01 Oct 2021 | – | 31 Dec 2021 | 84 | (550) | 15.3 | 12.4 - 18.6 | 11 | (120) | 9.2 | 4.7 - 15.8 | 73 | (430) | 17.0 | 13.5 - 20.9 |
|  | **Q1 2022** | 01 Jan 2022 | – | 31 Mar 2022 | 42 | (315) | 13.3 | 9.8 - 17.6 | 8 | (105) | 7.6 | 3.3 - 14.5 | 34 | (210) | 16.2 | 11.5 - 21.9 |
|  | **Q2 2022** | 01 Apr 2021 | – | 30 Jun 2021 | 26 | (103) | 25.2 | 17.2 - 34.8 | 8 | (31) | 25.8 | 11.9 - 44.6 | 18 | (72) | 25.0 | 15.5 - 36.6 |
|  | **Q3 2022** | 01 Jul 2022 | – | 30 Sep 2022 | 22 | (117) | 18.8 | 12.2 - 27.1 | 7 | (23) | 30.4 | 13.2 - 52.9 | 15 | (94) | 16.0 | 9.2 - 25.0 |
|  | **Q4 2022** | 01 Oct 2022 | – | 31 Dec 2022 | 21 | (125) | 16.8 | 10.7 - 24.5 | 1 | (21) | 4.8 | 0.1 - 23.8 | 20 | (104) | 19.2 | 12.2 - 28.1 |
|  | **Q1 2023** | 01 Jan 2023 | – | 31 Mar 2023 | 20 | (127) | 15.7 | 9.9 - 23.3 | 4 | (15) | 26.7 | 7.8 - 55.1 | 16 | (112) | 14.3 | 8.4 - 22.2 |
|  | **Q2 2023**† | 01 Apr 2023 | – | 31 May 2023 | 11 | (49) | 22.4 | 11.8 - 36.6 | 2 | (6) | 33.3 | 4.3 - 77.7 | 9 | (43) | 20.9 | 10.0 - 36.0 |
| **Alternative  definition** | **Overall** |  |  |  | 481 | (1,744) | 27.6 | 25.5 - 29.7 | 101 | (459) | 22.0 | 18.3 - 26.1 | 380 | (1,285) | 29.6 | 27.1 - 32.2 |
|  | **Q2 2021** | 01 June 2021 | – | 30 Jun 2021 | 18 | (80) | 22.5 | 13.9 - 33.2 | 1 | (22) | 4.5 | 0.1 - 22.8 | 17 | (58) | 29.3 | 18.1 - 42.7 |
|  | **Q3 2021** | 01 Jul 2021 | – | 30 Sep 2021 | 69 | (278) | 24.8 | 19.9 - 30.3 | 14 | (116) | 12.1 | 6.8 - 19.4 | 55 | (162) | 34.0 | 26.7 - 41.8 |
|  | **Q4 2021** | 01 Oct 2021 | – | 31 Dec 2021 | 142 | (550) | 25.8 | 22.2 - 29.7 | 25 | (120) | 20.8 | 14.0 - 29.2 | 117 | (430) | 27.2 | 23.1 - 31.7 |
|  | **Q1 2022** | 01 Jan 2022 | – | 31 Mar 2022 | 97 | (315) | 30.8 | 25.7 - 36.2 | 27 | (105) | 25.7 | 17.7 - 35.2 | 70 | (210) | 33.3 | 27.0 - 40.1 |
|  | **Q2 2022** | 01 Apr 2021 | – | 30 Jun 2021 | 38 | (103) | 36.9 | 27.6 - 47.0 | 10 | (31) | 32.3 | 16.7 - 51.4 | 28 | (72) | 38.9 | 27.6 - 51.1 |
|  | **Q3 2022** | 01 Jul 2022 | – | 30 Sep 2022 | 35 | (117) | 29.9 | 21.8 - 39.1 | 13 | (23) | 56.5 | 34.5 - 76.8 | 22 | (94) | 23.4 | 15.3 - 33.3 |
|  | **Q4 2022** | 01 Oct 2022 | – | 31 Dec 2022 | 30 | (125) | 24.0 | 16.8 - 32.5 | 3 | (21) | 14.3 | 3.0 - 36.3 | 27 | (104) | 26.0 | 17.9 - 35.5 |
|  | **Q1 2023** | 01 Jan 2023 | – | 31 Mar 2023 | 34 | (127) | 26.8 | 19.3 - 35.4 | 5 | (15) | 33.3 | 11.8 - 61.6 | 29 | (112) | 25.9 | 18.1 - 35.0 |
|  | **Q2 2023**† | 01 Apr 2023 | – | 31 May 2023 | 18 | (49) | 36.7 | 23.4 - 51.7 | 3 | (6) | 50.0 | 11.8 - 88.2 | 15 | (43) | 34.9 | 21.0 - 50.9 |

^a^ Main and alternative definition of IC. The alternative definition of IC was IC by main definition and/or cancer (Table 1); ^b^ Overall, entire study period 01 June 2021-31 May 2023; ^c^ Main SARI definition (Table 1); ^d^ Main SARS-CoV-2 test status definition (Table 1); †, this fiscal quarter included 1 out of 3 months. Abbreviations: CI, confidence interval; IC, immunocompromising condition; n, number of individuals with ICs; N, number of SARI patients; Q, calendar time in fiscal quarter; SARI, severe acute respiratory infection; SARS-CoV-2, severe acute respiratory syndrome coronavirus 2

**(Table S3 continued)**

**Panel C. Study site CHU Saint-Pierre (Centre Hospitalier Universitaire Saint-Pierre), Belgium**

| **IC definition ^a^** | **Period ^b^** | | | | **All SARI ^c^** | | | | **SARS-CoV-2 test status ^d^** | | | | | | | |
| --- | --- | --- | --- | --- | --- | --- | --- | --- | --- | --- | --- | --- | --- | --- | --- | --- |
|  |  |  |  |  |  |  |  |  | **Positive** | | | | **Negative** | | | |
|  |  |  |  |  |  | | **Prevalence** | |  | | **Prevalence** | |  | | **Prevalence** | |
|  |  |  |  |  | **n** | **(N)** | **%** | **95% CI** | **n** | **(N)** | **%** | **95% CI** | **n** | **(N)** | **%** | **95% CI** |
| **Main  definition** | **Overall** |  |  |  | 6 | (173) | 3.5 | 1.3 - 7.4 | 2 | (52) | 3.8 | 0.5 - 13.2 | 4 | (121) | 3.3 | 0.9 - 8.2 |
|  | **Q2 2021**† | 01 June 2021 | – | 30 Jun 2021 | 0 | (0) | NA | NA | 0 | (0) | NA | NA | 0 | (0) | NA | NA |
|  | **Q3 2021** | 01 Jul 2021 | – | 30 Sep 2021 | 2 | (26) | 7.7 | 0.9 - 25.1 | 2 | (13) | 15.4 | 1.9 - 45.4 | 0 | (13) | 0.0 | 0.0 - 24.7 |
|  | **Q4 2021** | 01 Oct 2021 | – | 31 Dec 2021 | 0 | (0) | NA | NA | 0 | (0) | NA | NA | 0 | (0) | NA | NA |
|  | **Q1 2022** | 01 Jan 2022 | – | 31 Mar 2022 | 0 | (0) | NA | NA | 0 | (0) | NA | NA | 0 | (0) | NA | NA |
|  | **Q2 2022** | 01 Apr 2021 | – | 30 Jun 2021 | 0 | (0) | NA | NA | 0 | (0) | NA | NA | 0 | (0) | NA | NA |
|  | **Q3 2022** | 01 Jul 2022 | – | 30 Sep 2022 | 1 | (9) | 11.1 | 0.3 - 48.2 | 0 | (4) | 0.0 | 0.0 - 60.2 | 1 | (5) | 20.0 | 0.5 - 71.6 |
|  | **Q4 2022** | 01 Oct 2022 | – | 31 Dec 2022 | 2 | (44) | 4.5 | 0.6 - 15.5 | 0 | (8) | 0.0 | 0.0 - 36.9 | 2 | (36) | 5.6 | 0.7 - 18.7 |
|  | **Q1 2023** | 01 Jan 2023 | – | 31 Mar 2023 | 1 | (25) | 40.0 | 0.1 - 20.4 | 0 | (3) | 0.0 | 0.0 - 70.8 | 1 | (22) | 4.5 | 0.1 - 22.8 |
|  | **Q2 2023**† | 01 Apr 2023 | – | 31 May 2023 | 0 | (0) | NA | NA | 0 | (0) | NA | NA | 0 | (0) | NA | NA |
| **Alternative  definition** | **Overall** |  |  |  | 32 | (173) | 18.5 | 13.0 - 25.1 | 9 | (52) | 17.3 | 8.2 - 30.3 | 23 | (121) | 19.0 | 12.4 - 27.1 |
|  | **Q2 2021** | 01 June 2021 | – | 30 Jun 2021 | 0 | (0) | NA | NA | 0 | (0) | NA | NA | 0 | (0) | NA | NA |
|  | **Q3 2021** | 01 Jul 2021 | – | 30 Sep 2021 | 5 | (26) | 19.2 | 6.6 - 39.4 | 3 | (13) | 23.1 | 5.0 - 53.8 | 2 | (13) | 15.4 | 1.9 - 45.4 |
|  | **Q4 2021** | 01 Oct 2021 | – | 31 Dec 2021 | 5 | (28) | 17.9 | 6.1 - 36.9 | 2 | (12) | 16.7 | 2.1 - 48.4 | 3 | (16) | 18.8 | 4.0 - 45.6 |
|  | **Q1 2022** | 01 Jan 2022 | – | 31 Mar 2022 | 4 | (25) | 16.0 | 4.5 - 36.1 | 1 | (9) | 11.1 | 0.3 - 48.2 | 3 | (16) | 18.8 | 4.0 - 45.6 |
|  | **Q2 2022** | 01 Apr 2021 | – | 30 Jun 2021 | 0 | (0) | NA | NA | 0 | (0) | NA | NA | 0 | (0) | NA | NA |
|  | **Q3 2022** | 01 Jul 2022 | – | 30 Sep 2022 | 3 | (9) | 33.3 | 7.5 - 70.1 | 2 | (4) | 50.0 | 6.8 - 93.2 | 1 | (5) | 20.0 | 0.5 - 71.6 |
|  | **Q4 2022** | 01 Oct 2022 | – | 31 Dec 2022 | 6 | (44) | 13.6 | 5.2 - 27.4 | 1 | (8) | 12.5 | 0.3 - 52.7 | 5 | (36) | 13.9 | 4.7 - 29.5 |
|  | **Q1 2023** | 01 Jan 2023 | – | 31 Mar 2023 | 7 | (25) | 28.0 | 12.1 - 49.4 | 0 | (3) | 0.0 | 0.0 - 70.8 | 7 | (22) | 31.8 | 13.9 - 54.9 |
|  | **Q2 2023**† | 01 Apr 2023 | – | 31 May 2023 | 2 | (11) | 18.2 | 2.3 - 51.8 | 0 | (1) | 0.0 | 0.0 - 97.5 | 2 | (10) | 20.0 | 2.5 - 55.6 |

^a^ Main and alternative definition of IC. The alternative definition of IC was IC by main definition and/or cancer (Table 1); ^b^ Overall, entire study period 01 June 2021-31 May 2023; ^c^ Main SARI definition (Table 1); ^d^ Main SARS-CoV-2 test status definition (Table 1); †, this fiscal quarter included 1 out of 3 months. Abbreviations: CI, confidence interval; IC, immunocompromising condition; n, number of individuals with ICs; N, number of SARI patients; NA, not applicable; Q, calendar time in fiscal quarter; SARI, severe acute respiratory infection; SARS-CoV-2, severe acute respiratory syndrome coronavirus 2

**(Table S3 continued)**

**Panel D. Study site UZA (Universitair Ziekenhuis Antwerpen), Belgium**

| **IC  definition ^a^** | **Period ^b^** | | | | **All SARI ^c^** | | | | **SARS-CoV-2 test status ^d^** | | | | | | | |
| --- | --- | --- | --- | --- | --- | --- | --- | --- | --- | --- | --- | --- | --- | --- | --- | --- |
|  |  |  |  |  |  |  |  |  | **Positive** | | | | **Negative** | | | |
|  |  |  |  |  |  | | **Prevalence** | |  | | **Prevalence** | |  | | **Prevalence** | |
|  |  |  |  |  | **n** | **(N)** | **%** | **95% CI** | **n** | **(N)** | **%** | **95% CI** | **n** | **(N)** | **%** | **95% CI** |
| **Main  definition** | **Overall** |  |  |  | 77 | (395) | 19.5 | 15.7 - 23.7 | 58 | (254) | 22.8 | 17.8 - 28.5 | 19 | (141) | 13.5 | 8.3 - 20.2 |
|  | **Q2 2021**† | 01 June 2021 | – | 30 Jun 2021 | 0 | (0) | NA | NA | 0 | (0) | NA | NA | 0 | (0) | NA | NA |
|  | **Q3 2021** | 01 Jul 2021 | – | 30 Sep 2021 | 10 | (74) | 13.5 | 6.7 - 23.5 | 9 | (57) | 15.8 | 7.5 - 27.9 | 1 | (17) | 5.9 | 0.1 - 28.7 |
|  | **Q4 2021** | 01 Oct 2021 | – | 31 Dec 2021 | 34 | (134) | 25.4 | 18.3 - 33.6 | 29 | (109) | 26.6 | 18.6 - 35.9 | 5 | (25) | 20.0 | 6.8 - 40.7 |
|  | **Q1 2022** | 01 Jan 2022 | – | 31 Mar 2022 | 16 | (72) | 22.2 | 13.3 - 33.6 | 12 | (48) | 25.0 | 13.6 - 39.6 | 4 | (24) | 16.7 | 4.7 - 37.4 |
|  | **Q2 2022** | 01 Apr 2021 | – | 30 Jun 2021 | 4 | (18) | 22.2 | 6.4 - 47.6 | 3 | (12) | 25.0 | 5.5 - 57.2 | 1 | (6) | 16.7 | 0.4 - 64.1 |
|  | **Q3 2022** | 01 Jul 2022 | – | 30 Sep 2022 | 1 | (16) | 6.2 | 0.2 - 30.2 | 1 | (9) | 11.1 | 0.3 - 48.2 | 0 | (7) | 0.0 | 0.0 - 41.0 |
|  | **Q4 2022** | 01 Oct 2022 | – | 31 Dec 2022 | 5 | (30) | 16.7 | 5.6 - 34.7 | 2 | (5) | 40.0 | 5.3 - 85.3 | 3 | (25) | 12.0 | 2.5 - 31.2 |
|  | **Q1 2023** | 01 Jan 2023 | – | 31 Mar 2023 | 6 | (32) | 18.8 | 7.2 - 36.4 | 2 | (7) | 28.6 | 3.7 - 71.0 | 4 | (25) | 16.0 | 4.5 - 36.1 |
|  | **Q2 2023**† | 01 Apr 2023 | – | 31 May 2023 | 1 | (6) | 16.7 | 0.4 - 64.1 | 0 | (1) | 0.0 | 0.0 - 97.5 | 1 | (5) | 20.0 | 0.5 - 71.6 |
| **Alternative  definition** | **Overall** |  |  |  | 95 | (395) | 24.1 | 19.9 - 28.6 | 68 | (254) | 26.8 | 21.4 - 32.7 | 27 | (141) | 19.1 | 13.0 - 26.6 |
|  | **Q2 2021** | 01 June 2021 | – | 30 Jun 2021 | 0 | (0) | NA | NA | 0 | (0) | NA | NA | 0 | (0) | NA | NA |
|  | **Q3 2021** | 01 Jul 2021 | – | 30 Sep 2021 | 14 | (74) | 18.9 | 10.7 - 29.7 | 11 | (57) | 19.3 | 10.0 - 31.9 | 3 | (17) | 17.6 | 3.8 - 43.4 |
|  | **Q4 2021** | 01 Oct 2021 | – | 31 Dec 2021 | 37 | (134) | 27.6 | 20.2 - 36 | 32 | (109) | 29.4 | 21.0 - 38.8 | 5 | (25) | 20.0 | 6.8 - 40.7 |
|  | **Q1 2022** | 01 Jan 2022 | – | 31 Mar 2022 | 16 | (72) | 22.2 | 13.3 - 33.6 | 12 | (48) | 25.0 | 13.6 - 39.6 | 4 | (24) | 16.7 | 4.7 - 37.4 |
|  | **Q2 2022** | 01 Apr 2021 | – | 30 Jun 2021 | 6 | (18) | 33.3 | 13.3 - 59.0 | 5 | (12) | 41.7 | 15.2 - 72.3 | 1 | (6) | 16.7 | 0.4 - 64.1 |
|  | **Q3 2022** | 01 Jul 2022 | – | 30 Sep 2022 | 4 | (16) | 25.0 | 7.3 - 52.4 | 2 | (9) | 22.2 | 2.8 - 60.0 | 2 | (7) | 28.6 | 3.7 - 71.0 |
|  | **Q4 2022** | 01 Oct 2022 | – | 31 Dec 2022 | 6 | (30) | 20.0 | 7.7 - 38.6 | 2 | (5) | 40.0 | 5.3 - 85.3 | 4 | (25) | 16.0 | 4.5 - 36.1 |
|  | **Q1 2023** | 01 Jan 2023 | – | 31 Mar 2023 | 10 | (32) | 31.2 | 16.1 - 50.0 | 3 | (7) | 42.9 | 9.9 - 81.6 | 7 | (25) | 28.0 | 12.1 - 49.4 |
|  | **Q2 2023**† | 01 Apr 2023 | – | 31 May 2023 | 2 | 6) | 33.3 | 4.3 - 77.7 | 1 | (1) | 100.0 | 2.5 - 100.0 | 1 | (5) | 20.0 | 0.5 - 71.6 |

^a^ Main and alternative definition of IC. The alternative definition of IC was IC by main definition and/or cancer (Table 1); ^b^ Overall, entire study period 01 June 2021-31 May 2023; ^c^ Main SARI definition (Table 1); ^d^ Main SARS-CoV-2 test status definition (Table 1); †, this fiscal quarter included 1 out of 3 months. Abbreviations: CI, confidence interval; IC, immunocompromising condition; n, number of individuals with ICs; N, number of SARI patients; NA, not applicable; Q, calendar time in fiscal quarter; SARI, severe acute respiratory infection; SARS-CoV-2, severe acute respiratory syndrome coronavirus 2

**(Table S3 continued)**

**Panel E. Study site HUVH (Hospital Universitari Vall d’Hebron), Spain**

| **IC  definition ^a^** | **Period ^b^** | | | | **All SARI ^c^** | | | | **SARS-CoV-2 test status ^d^** | | | | | | | |
| --- | --- | --- | --- | --- | --- | --- | --- | --- | --- | --- | --- | --- | --- | --- | --- | --- |
|  |  |  |  |  |  |  |  |  | **Positive** | | | | **Negative** | | | |
|  |  |  |  |  |  | | **Prevalence** | |  | | **Prevalence** | |  | | **Prevalence** | |
|  |  |  |  |  | **n** | **(N)** | **%** | **95% CI** | **n** | **(N)** | **%** | **95% CI** | **n** | **(N)** | **%** | **95% CI** |
| **Main  definition** | **Overall** |  |  |  | 302 | (1,679) | 18.0 | 16.2 - 19.9 | 134 | (678) | 19.8 | 16.8 - 23.0 | 168 | (1,001) | 16.8 | 14.5 - 19.2 |
|  | **Q2 2021***†* | 01 June 2021 | – | 30 Jun 2021 | 0 | (0) | NA | NA | 0 | (0) | NA | NA | 0 | (0) | NA | NA |
|  | **Q3 2021** | 01 Jul 2021 | – | 30 Sep 2021 | 0 | (0) | NA | NA | 0 | (0) | NA | NA | 0 | (0) | NA | NA |
|  | **Q4 2021** | 01 Oct 2021 | – | 31 Dec 2021 | 26 | (218) | 11.9 | 7.9 - 17.0 | 8 | (93) | 8.6 | 3.8 - 16.2 | 18 | (125) | 14.4 | 8.8 - 21.8 |
|  | **Q1 2022** | 01 Jan 2022 | – | 31 Mar 2022 | 31 | (197) | 15.7 | 10.9 - 21.6 | 24 | (149) | 16.1 | 10.6 - 23.0 | 7 | (48) | 14.6 | 6.1 - 27.8 |
|  | **Q2 2022** | 01 Apr 2021 | – | 30 Jun 2021 | 38 | (203) | 18.7 | 13.6 - 24.8 | 33 | (169) | 19.5 | 13.8 - 26.3 | 5 | (34) | 14.7 | 5.0 - 31.1 |
|  | **Q3 2022** | 01 Jul 2022 | – | 30 Sep 2022 | 52 | (158) | 32.9 | 25.7 - 40.8 | 26 | (78) | 33.3 | 23.1 - 44.9 | 26 | (80) | 32.5 | 22.4 - 43.9 |
|  | **Q4 2022** | 01 Oct 2022 | – | 31 Dec 2022 | 83 | (395) | 21.0 | 17.1 - 25.4 | 29 | (96) | 30.2 | 21.3 - 40.4 | 54 | (299) | 18.1 | 13.9 - 22.9 |
|  | **Q1 2023** | 01 Jan 2023 | – | 31 Mar 2023 | 45 | (333) | 13.5 | 10.0 - 17.7 | 11 | (59) | 18.6 | 9.7 - 30.9 | 34 | (274) | 12.4 | 8.7 - 16.9 |
|  | **Q2 2023**† | 01 Apr 2023 | – | 31 May 2023 | 27 | (175) | 15.4 | 10.4 - 21.6 | 3 | (34) | 8.8 | 1.9 - 23.7 | 24 | (141) | 17.0 | 11.2 - 24.3 |
| **Alternative  definition** | **Overall** |  |  |  | 615 | (1,679) | 36.6 | 34.3 - 39.0 | 277 | (678) | 40.9 | 37.1 - 44.7 | 338 | (1,001) | 33.8 | 30.8 - 36.8 |
|  | **Q2 2021** | 01 June 2021 | – | 30 Jun 2021 | 0 | (0) | NA | NA | 0 | (0) | NA | NA | 0 | (0) | NA | NA |
|  | **Q3 2021** | 01 Jul 2021 | – | 30 Sep 2021 | 0 | (0) | NA | NA | 0 | (0) | NA | NA | 0 | (0) | NA | NA |
|  | **Q4 2021** | 01 Oct 2021 | – | 31 Dec 2021 | 50 | (218) | 22.9 | 17.5 - 29.1 | 15 | (93) | 16.1 | 9.3 - 25.2 | 35 | (125) | 28.0 | 20.3 - 36.7 |
|  | **Q1 2022** | 01 Jan 2022 | – | 31 Mar 2022 | 74 | (197) | 37.6 | 30.8 - 44.7 | 59 | (149) | 39.6 | 31.7 - 47.9 | 15 | (48) | 31.2 | 18.7 - 46.3 |
|  | **Q2 2022** | 01 Apr 2021 | – | 30 Jun 2021 | 88 | (203) | 43.3 | 36.4 - 50.5 | 76 | (169) | 45.0 | 37.3 - 52.8 | 12 | (34) | 35.3 | 19.7 - 53.5 |
|  | **Q3 2022** | 01 Jul 2022 | – | 30 Sep 2022 | 78 | (158) | 49.4 | 41.3 - 57.4 | 42 | (78) | 53.8 | 42.2 - 65.2 | 36 | (80) | 45.0 | 33.8 - 56.5 |
|  | **Q4 2022** | 01 Oct 2022 | – | 31 Dec 2022 | 139 | (395) | 35.2 | 30.5 - 40.1 | 44 | (96) | 45.8 | 35.6 - 56.3 | 95 | (299) | 31.8 | 26.5 - 37.4 |
|  | **Q1 2023** | 01 Jan 2023 | – | 31 Mar 2023 | 112 | (333) | 33.6 | 28.6 - 39.0 | 25 | (59) | 42.4 | 29.6 - 55.9 | 87 | (274) | 31.8 | 26.3 - 37.6 |
|  | **Q2 2023**† | 01 Apr 2023 | – | 31 May 2023 | 74 | (175) | 42.3 | 34.9 - 50.0 | 16 | (34) | 47.1 | 29.8 - 64.9 | 58 | (141) | 41.1 | 32.9 - 49.7 |

^a^ Main and alternative definition of IC. The alternative definition of IC was IC by main definition and/or cancer (Table 1); ^b^ Overall, entire study period 01 June 2021-31 May 2023; ^c^ Main SARI definition (Table 1); ^d^ Main SARS-CoV-2 test status definition (Table 1); †, this fiscal quarter included 1 out of 3 months. Abbreviations: CI, confidence interval; IC, immunocompromising condition; n, number of individuals with ICs; N, number of SARI patients; NA, not applicable; Q, calendar time in fiscal quarter; SARI, severe acute respiratory infection; SARS-CoV-2, severe acute respiratory syndrome coronavirus 2

Table S4. IC prevalence (main definition) among hospitalized SARI patients, for subgroups defined by in-hospital outcome severity levels, by SARS-CoV-2 test status, and by calendar time

| **SARI severity level** | **Period ^b^** | | | | **All SARI ^c^** | | | | **SARS-CoV-2 test status ^d^** | | | | | | | | |
| --- | --- | --- | --- | --- | --- | --- | --- | --- | --- | --- | --- | --- | --- | --- | --- | --- | --- |
|  |  |  |  |  |  |  |  |  | **Positive** | | | | **Negative** | | | | |
|  |  |  |  |  |  |  | **IC ^a^ prevalence** | |  |  | **IC ^a^ prevalence** | | |  |  | **IC ^a^ prevalence** | |
|  |  |  |  |  | **n** | **(N)** | **%** | **95% CI** | **n** | **(N)** | **%** | **95% CI** | | **n** | **(N)** | **%** | **95% CI** |
| **Hospitalization without ICU admission or in-hospital death** | **Overall** |  |  |  | 628 | (4,564) | 13.8 | 12.8 - 14.8 | 227 | (1,525) | 14.9 | 13.1 - 16.8 | | 401 | (3,039) | 13.2 | 12.0 - 14.5 |
|  | **Q2 2021** | 01 June 2021 | – | 30 Jun 2021 | 7 | (79) | 8.9 | 3.6 - 17.4 | 1 | (21) | 4.8 | 0.1 - 23.8 | | 6 | (58) | 10.3 | 3.9 - 21.2 |
|  | **Q3 2021** | 01 Jul 2021 | – | 30 Sep 2021 | 37 | (268) | 13.8 | 9.9 - 18.5 | 12 | (115) | 10.4 | 5.5 - 17.5 | | 25 | (153) | 16.3 | 10.9 - 23.2 |
|  | **Q4 2021** | 01 Oct 2021 | – | 31 Dec 2021 | 127 | (798) | 15.9 | 13.4 - 18.6 | 42 | (258) | 16.3 | 12.0 - 21.4 | | 85 | (540) | 15.7 | 12.8 - 19.1 |
|  | **Q1 2022** | 01 Jan 2022 | – | 31 Mar 2022 | 96 | (1,006) | 9.5 | 7.8 - 11.5 | 44 | (438) | 10.0 | 7.4 - 13.3 | | 52 | (568) | 9.2 | 6.9 - 11.8 |
|  | **Q2 2022** | 01 Apr 2021 | – | 30 Jun 2021 | 73 | (492) | 14.8 | 11.8 - 18.3 | 45 | (256) | 17.6 | 13.1 - 22.8 | | 28 | (236) | 11.9 | 8.0 - 16.7 |
|  | **Q3 2022** | 01 Jul 2022 | – | 30 Sep 2022 | 75 | (344) | 21.8 | 17.6 - 26.5 | 32 | (136) | 23.5 | 16.7 - 31.6 | | 43 | (208) | 20.7 | 15.4 - 26.8 |
|  | **Q4 2022** | 01 Oct 2022 | – | 31 Dec 2022 | 101 | (691) | 14.6 | 12.1 - 17.5 | 29 | (151) | 19.2 | 13.3 - 26.4 | | 72 | (540) | 13.3 | 10.6 - 16.5 |
|  | **Q1 2023** | 01 Jan 2023 | – | 31 Mar 2023 | 78 | (647) | 12.1 | 9.6 - 14.8 | 18 | (109) | 16.5 | 10.1 - 24.8 | | 60 | (538) | 11.2 | 8.6 - 14.1 |
|  | **Q2 2023**† | 01 Apr 2023 | – | 31 May 2023 | 34 | (239) | 14.2 | 10.1 - 19.3 | 4 | (41) | 9.8 | 2.7 - 23.1 | | 30 | (198) | 15.2 | 10.5 - 20.9 |
| **ICU admission with or without in-hospital death** | **Overall** |  |  |  | 63 | (435) | 14.5 | 11.3 - 18.1 | 39 | (286) | 13.6 | 9.9 - 18.2 | | 24 | (149) | 16.1 | 10.6 - 23.0 |
|  | **Q2 2021** | 01 June 2021 | – | 30 Jun 2021 | 0 | (10) | 0.0 | 0.0 - 30.8 | 0 | (8) | 0.0 | 0.0 - 36.9 | | 0 | (2) | 0.0 | 0.0 - 84.2 |
|  | **Q3 2021** | 01 Jul 2021 | – | 30 Sep 2021 | 7 | (66) | 10.6 | 4.4 - 20.6 | 6 | (58) | 10.3 | 3.9 - 21.2 | | 1 | (8) | 12.5 | 0.3 - 52.7 |
|  | **Q4 2021** | 01 Oct 2021 | – | 31 Dec 2021 | 11 | (142) | 7.7 | 3.9 - 13.4 | 8 | (112) | 7.1 | 3.1 - 13.6 | | 3 | (30) | 10.0 | 2.1 - 26.5 |
|  | **Q1 2022** | 01 Jan 2022 | – | 31 Mar 2022 | 10 | (64) | 15.6 | 7.8 - 26.9 | 9 | (54) | 16.7 | 7.9 - 29.3 | | 1 | (10) | 10.0 | 0.3 - 44.5 |
|  | **Q2 2022** | 01 Apr 2021 | – | 30 Jun 2021 | 7 | (22) | 31.8 | 13.9 - 54.9 | 5 | (17) | 29.4 | 10.3 - 56.0 | | 2 | (5) | 40.0 | 5.3 - 85.3 |
|  | **Q3 2022** | 01 Jul 2022 | – | 30 Sep 2022 | 7 | (25) | 28.0 | 12.1 - 49.4 | 5 | (14) | 35.7 | 12.8 - 64.9 | | 2 | (11) | 18.2 | 2.3 - 51.8 |
|  | **Q4 2022** | 01 Oct 2022 | – | 31 Dec 2022 | 12 | (45) | 26.7 | 14.6 - 41.9 | 5 | (14) | 35.7 | 12.8 - 64.9 | | 7 | (31) | 22.6 | 9.6 - 41.1 |
|  | **Q1 2023** | 01 Jan 2023 | – | 31 Mar 2023 | 5 | (37) | 13.5 | 4.5 - 28.8 | 0 | (5) | 0.0 | 0.0 - 52.2 | | 5 | (32) | 15.6 | 5.3 - 32.8 |
|  | **Q2 2023**† | 01 Apr 2023 | – | 31 May 2023 | 4 | (24) | 16.7 | 4.7 - 37.4 | 1 | (4) | 25.0 | 0.6 - 80.6 | | 3 | (20) | 15.0 | 3.2 - 37.9 |

**(Table S4 continued)**

| **SARI severity level** | **Period ^b^** | | | | **All SARI ^c^** | | | | **SARS-CoV-2 test status ^d^** | | | | | | | | |
| --- | --- | --- | --- | --- | --- | --- | --- | --- | --- | --- | --- | --- | --- | --- | --- | --- | --- |
|  |  |  |  |  |  |  |  |  | **Positive** | | | | **Negative** | | | | |
|  |  |  |  |  |  |  | **IC ^a^ prevalence** | |  |  | **IC ^a^ prevalence** | | |  |  | **IC ^a^ prevalence** | |
|  |  |  |  |  | **n** | **(N)** | **%** | **95% CI** | **n** | **(N)** | **%** | **95% CI** | | **n** | **(N)** | **%** | **95% CI** |
| **ICU admission without in-hospital death** | **Overall** |  |  |  | 36 | (334) | 10.8 | 7.7 - 14.6 | 18 | (207) | 8.7 | 5.2 - 13.4 | | 18 | (127) | 14.2 | 8.6 - 21.5 |
|  | **Q2 2021** | 01 June 2021 | – | 30 Jun 2021 | 0 | (9) | 0.0 | 0.0 - 33.6 | 0 | (7) | 0.0 | 0.0 - 41.0 | | 0 | (2) | 0.0 | 0.0 - 84.2 |
|  | **Q3 2021** | 01 Jul 2021 | – | 30 Sep 2021 | 4 | (54) | 7.4 | 2.1 - 17.9 | 3 | (46) | 6.5 | 1.4 - 17.9 | | 1 | (8) | 12.5 | 0.3 - 52.7 |
|  | **Q4 2021** | 01 Oct 2021 | – | 31 Dec 2021 | 4 | (104) | 3.8 | 1.1 - 9.6 | 3 | (80) | 3.8 | 0.8 - 10.6 | | 1 | (24) | 4.2 | 0.1 - 21.1 |
|  | **Q1 2022** | 01 Jan 2022 | – | 31 Mar 2022 | 5 | (46) | 10.9 | 3.6 - 23.6 | 4 | (37) | 10.8 | 3.0 - 25.4 | | 1 | (9) | 11.1 | 0.3 - 48.2 |
|  | **Q2 2022** | 01 Apr 2021 | – | 30 Jun 2021 | 5 | (16) | 31.2 | 11.0 - 58.7 | 3 | (12) | 25.0 | 5.5 - 57.2 | | 2 | (4) | 50.0 | 6.8 - 93.2 |
|  | **Q3 2022** | 01 Jul 2022 | – | 30 Sep 2022 | 3 | (16) | 18.8 | 4.0 - 45.6 | 1 | (7) | 14.3 | 0.4 - 57.9 | | 2 | (9) | 22.2 | 2.8 - 60.0 |
|  | **Q4 2022** | 01 Oct 2022 | – | 31 Dec 2022 | 7 | (36) | 19.4 | 8.2 - 36.0 | 3 | (10) | 30.0 | 6.7 - 65.2 | | 4 | (26) | 15.4 | 4.4 - 34.9 |
|  | **Q1 2023** | 01 Jan 2023 | – | 31 Mar 2023 | 4 | (32) | 12.5 | 3.5 - 29.0 | 0 | (5) | 0.0 | 0.0 - 52.2 | | 4 | (27) | 14.8 | 4.2 - 33.7 |
|  | **Q2 2023**† | 01 Apr 2023 | – | 31 May 2023 | 4 | (21) | 19.0 | 5.4 - 41.9 | 1 | (3) | 33.3 | 0.8 - 90.6 | | 3 | (18) | 16.7 | 3.6 - 41.4 |
| **In-hospital death** | **Overall** |  |  |  | 68 | (381) | 17.8 | 14.1 - 22.1 | 29 | (191) | 15.2 | 10.4 - 21.1 | | 39 | (190) | 20.5 | 15.0 - 27.0 |
|  | **Q2 2021** | 01 June 2021 | – | 30 Jun 2021 | 1 | (8) | 12.5 | 0.3 - 52.7 | 0 | (1) | 0.0 | 0.0 - 97.5 | | 1 | (7) | 14.3 | 0.4 - 57.9 |
|  | **Q3 2021** | 01 Jul 2021 | – | 30 Sep 2021 | 11 | (56) | 19.6 | 10.2 - 32.4 | 3 | (25) | 12.0 | 2.5 - 31.2 | | 8 | (31) | 25.8 | 11.9 - 44.6 |
|  | **Q4 2021** | 01 Oct 2021 | – | 31 Dec 2021 | 18 | (115) | 15.7 | 9.5 - 23.6 | 5 | (53) | 9.4 | 3.1 - 20.7 | | 13 | (62) | 21.0 | 11.7 - 33.2 |
|  | **Q1 2022** | 01 Jan 2022 | – | 31 Mar 2022 | 10 | (60) | 16.7 | 8.3 - 28.5 | 7 | (48) | 14.6 | 6.1 - 27.8 | | 3 | (12) | 25.0 | 5.5 - 57.2 |
|  | **Q2 2022** | 01 Apr 2021 | – | 30 Jun 2021 | 5 | (29) | 17.2 | 5.8 - 35.8 | 5 | (24) | 20.8 | 7.1 - 42.2 | | 0 | (5) | 0.0 | 0.0 - 52.2 |
|  | **Q3 2022** | 01 Jul 2022 | – | 30 Sep 2022 | 7 | (27) | 25.9 | 11.1 - 46.3 | 5 | (17) | 29.4 | 10.3 - 56.0 | | 2 | (10) | 20.0 | 2.5 - 55.6 |
|  | **Q4 2022** | 01 Oct 2022 | – | 31 Dec 2022 | 12 | (37) | 32.4 | 18.0 - 49.8 | 4 | (18) | 22.2 | 6.4 - 47.6 | | 8 | (19) | 42.1 | 20.3 - 66.5 |
|  | **Q1 2023** | 01 Jan 2023 | – | 31 Mar 2023 | 1 | (31) | 3.2 | 0.1 - 16.7 | 0 | (2) | 0.0 | 0.0 - 84.2 | | 1 | (29) | 3.4 | 0.1 - 17.8 |
|  | **Q2 2023**† | 01 Apr 2023 | – | 31 May 2023 | 3 | (18) | 16.7 | 3.6 - 41.4 | 0 | (3) | 0.0 | 0.0 - 70.8 | | 3 | (15) | 20.0 | 4.3 - 48.1 |

^a^ Main definition of IC (Table 1); ^b^ Overall, the entire study time from 01 June 2021 to 31 May 2023; Time periods are divided according to fiscal quarters. ^c^ Main SARI definition (Table 1); ^d^ Main SARS-CoV-2 test status definition (Table 1); †, this fiscal quarter included 1 out of 3 months. Abbreviations: CI, confidence interval; IC, immunocompromising condition; ICU, intensive care unit; n, number of individuals with ICs; N, number of SARI patients; Q, calendar time in fiscal quarter; SARI, severe acute respiratory infection; SARS-CoV-2, severe acute respiratory syndrome coronavirus 2

Table S5. IC prevalence (alternative definition) among hospitalized SARI patients, for subgroups defined by in-hospital outcome severity levels, by SARS-CoV-2 test status, and by calendar time

| **SARI severity level** | **Period ^b^** | | | | **All SARI ^c^** | | | | | **SARS-CoV-2 test status ^d^** | | | | | | | | |
| --- | --- | --- | --- | --- | --- | --- | --- | --- | --- | --- | --- | --- | --- | --- | --- | --- | --- | --- |
|  |  |  |  |  |  |  |  |  |  | **Positive** | | | | **Negative** | | | | |
|  |  |  |  |  |  |  | | **IC ^a^ prevalence** | |  |  | **IC ^a^ prevalence** | |  | |  | **IC ^a^ prevalence** | |
|  |  |  |  |  | **n** | **(N)** | | **%** | **95% CI** | **n** | **(N)** | **%** | **95% CI** | **n** | | **(N)** | **%** | **95% CI** |
| **Hospitalization without ICU admission or in-hospital death** | **Overall** |  |  |  | 1,315 | | (4,564) | 28.8 | 27.5 - 30.2 | 472 | (1,525) | 31.0 | 28.6 - 33.3 | 843 | (3,039) | | 27.7 | 26.2 - 29.4 |
|  | **Q2 2021** | 01 June 2021 | – | 30 Jun 2021 | 15 | | (79) | 19.0 | 11.0 - 29.4 | 1 | (21) | 4.8 | 0.1 - 23.8 | 14 | (58) | | 24.1 | 13.9 - 37.2 |
|  | **Q3 2021** | 01 Jul 2021 | – | 30 Sep 2021 | 63 | | (268) | 23.5 | 18.6 - 29.0 | 18 | (115) | 15.7 | 9.5 - 23.6 | 45 | (153) | | 29.4 | 22.3 - 37.3 |
|  | **Q4 2021** | 01 Oct 2021 | – | 31 Dec 2021 | 216 | | (798) | 27.1 | 24.0 - 30.3 | 68 | (258) | 26.4 | 21.1 - 32.2 | 148 | (540) | | 27.4 | 23.7 - 31.4 |
|  | **Q1 2022** | 01 Jan 2022 | – | 31 Mar 2022 | 269 | | (1,006) | 26.7 | 24.0 - 29.6 | 125 | (438) | 28.5 | 24.4 - 33.0 | 144 | (568) | | 25.4 | 21.8 - 29.1 |
|  | **Q2 2022** | 01 Apr 2021 | – | 30 Jun 2021 | 157 | | (492) | 31.9 | 27.8 - 36.2 | 95 | (256) | 37.1 | 31.2 - 43.3 | 62 | (236) | | 26.3 | 20.8 - 32.4 |
|  | **Q3 2022** | 01 Jul 2022 | – | 30 Sep 2022 | 125 | | (344) | 36.3 | 31.2 - 41.7 | 59 | (136) | 43.4 | 34.9 - 52.1 | 66 | (208) | | 31.7 | 25.5 - 38.5 |
|  | **Q4 2022** | 01 Oct 2022 | – | 31 Dec 2022 | 195 | | (691) | 28.2 | 24.9 - 31.7 | 50 | (151) | 33.1 | 25.7 - 41.2 | 145 | (540) | | 26.9 | 23.2 - 30.8 |
|  | **Q1 2023** | 01 Jan 2023 | – | 31 Mar 2023 | 186 | | (647) | 28.7 | 25.3 - 32.4 | 37 | (109) | 33.9 | 25.1 - 43.6 | 149 | (538) | | 27.7 | 24.0 - 31.7 |
|  | **Q2 2023**† | 01 Apr 2023 | – | 31 May 2023 | 89 | | (239) | 37.2 | 31.1 - 43.7 | 19 | (41) | 46.3 | 30.7 - 62.6 | 70 | (198) | | 35.4 | 28.7 - 42.4 |
| **ICU admission with or without in-hospital death** | **Overall** |  |  |  | 104 | | (435) | 23.9 | 20.0 - 28.2 | 58 | (286) | 20.3 | 15.8 - 25.4 | 46 | (149) | | 30.9 | 23.6 - 39.0 |
|  | **Q2 2021** | 01 June 2021 | – | 30 Jun 2021 | 0 | | (10) | 0.0 | 0.0 - 30.8 | 0 | (8) | 0.0 | 0.0 - 36.9 | 0 | (2) | | 0.0 | 0.0 - 84.2 |
|  | **Q3 2021** | 01 Jul 2021 | – | 30 Sep 2021 | 10 | | (66) | 15.2 | 7.5 - 26.1 | 8 | (58) | 13.8 | 6.1 - 25.4 | 2 | (8) | | 25.0 | 3.2 - 65.1 |
|  | **Q4 2021** | 01 Oct 2021 | – | 31 Dec 2021 | 19 | | (142) | 13.4 | 8.3 - 20.1 | 13 | (112) | 11.6 | 6.3 - 19.0 | 6 | (30) | | 20.0 | 7.7 - 38.6 |
|  | **Q1 2022** | 01 Jan 2022 | – | 31 Mar 2022 | 18 | | (64) | 28.1 | 17.6 - 40.8 | 14 | (54) | 25.9 | 15.0 - 39.7 | 4 | (10) | | 40.0 | 12.2 - 73.8 |
|  | **Q2 2022** | 01 Apr 2021 | – | 30 Jun 2021 | 11 | | (22) | 50.0 | 28.2 - 71.8 | 8 | (17) | 47.1 | 23.0 - 72.2 | 3 | (5) | | 60.0 | 14.7 - 94.7 |
|  | **Q3 2022** | 01 Jul 2022 | – | 30 Sep 2022 | 8 | | (25) | 32.0 | 14.9 - 53.5 | 6 | (14) | 42.9 | 17.7 - 71.1 | 2 | (11) | | 18.2 | 2.3 - 51.8 |
|  | **Q4 2022** | 01 Oct 2022 | – | 31 Dec 2022 | 17 | | (45) | 37.8 | 23.8 - 53.5 | 7 | (14) | 50.0 | 23.0 - 77.0 | 10 | (31) | | 32.3 | 16.7 - 51.4 |
|  | **Q1 2023** | 01 Jan 2023 | – | 31 Mar 2023 | 11 | | (37) | 29.7 | 15.9 - 47.0 | 1 | (5) | 20.0 | 0.5 - 71.6 | 10 | (32) | | 31.2 | 16.1 - 50.0 |
|  | **Q2 2023**† | 01 Apr 2023 | – | 31 May 2023 | 10 | | (24) | 41.7 | 22.1 - 63.4 | 1 | (4) | 25.0 | 0.6 - 80.6 | 9 | (20) | | 45.0 | 23.1 - 68.5 |

**(Table S5 continued)**

| **SARI severity level** | **Period ^b^** | | | | **All SARI ^c^** | | | | **SARS-CoV-2 test status ^d^** | | | | | | | | |
| --- | --- | --- | --- | --- | --- | --- | --- | --- | --- | --- | --- | --- | --- | --- | --- | --- | --- |
|  |  |  |  |  |  |  |  |  | **Positive** | | | | **Negative** | | | | |
|  |  |  |  |  |  |  | **IC ^a^ prevalence** | |  |  | **IC ^a^ prevalence** | | |  |  | **IC ^a^ prevalence** | |
|  |  |  |  |  | **n** | **(N)** | **%** | **95% CI** | **n** | **(N)** | **%** | **95% CI** | | **n** | **(N)** | **%** | **95% CI** |
| **ICU admission without in-hospital death** | **Overall** |  |  |  | 66 | (334) | 19.8 | 15.6 - 24.4 | 30 | (207) | 14.5 | 10.0 - 20.0 | | 36 | (127) | 28.3 | 20.7 - 37.0 |
|  | **Q2 2021** | 01 June 2021 | – | 30 Jun 2021 | 0 | (9) | 0.0 | 0.0 - 33.6 | 0 | (7) | 0.0 | 0.0 - 41.0 | | 0 | (2) | 0.0 | 0.0 - 84.2 |
|  | **Q3 2021** | 01 Jul 2021 | – | 30 Sep 2021 | 6 | (54) | 11.1 | 4.2 - 22.6 | 4 | (46) | 8.7 | 2.4 - 20.8 | | 2 | (8) | 25.0 | 3.2 - 65.1 |
|  | **Q4 2021** | 01 Oct 2021 | – | 31 Dec 2021 | 9 | (104) | 8.7 | 4.0 - 15.8 | 6 | (80) | 7.5 | 2.8 - 15.6 | | 3 | (24) | 12.5 | 2.7 - 32.4 |
|  | **Q1 2022** | 01 Jan 2022 | – | 31 Mar 2022 | 12 | (46) | 26.1 | 14.3 - 41.1 | 9 | (37) | 24.3 | 11.8 - 41.2 | | 3 | (9) | 33.3 | 7.5 - 70.1 |
|  | **Q2 2022** | 01 Apr 2021 | – | 30 Jun 2021 | 7 | (16) | 43.8 | 19.8 - 70.1 | 4 | (12) | 33.3 | 9.9 - 65.1 | | 3 | (4) | 75.0 | 19.4 - 99.4 |
|  | **Q3 2022** | 01 Jul 2022 | – | 30 Sep 2022 | 3 | (16) | 18.8 | 4.0 - 45.6 | 1 | (7) | 14.3 | 0.4 - 57.9 | | 2 | (9) | 22.2 | 2.8 - 60.0 |
|  | **Q4 2022** | 01 Oct 2022 | – | 31 Dec 2022 | 9 | (36) | 25.0 | 12.1 - 42.2 | 4 | (10) | 40.0 | 12.2 - 73.8 | | 5 | (26) | 19.2 | 6.6 - 39.4 |
|  | **Q1 2023** | 01 Jan 2023 | – | 31 Mar 2023 | 10 | (32) | 31.2 | 16.1 - 50.0 | 1 | (5) | 20.0 | 0.5 - 71.6 | | 9 | (27) | 33.3 | 16.5 - 54.0 |
|  | **Q2 2023**† | 01 Apr 2023 | – | 31 May 2023 | 10 | (21) | 47.6 | 25.7 - 70.2 | 1 | (3) | 33.3 | 0.8 - 90.6 | | 9 | (18) | 50.0 | 26.0 - 74.0 |
| **In-hospital death** | **Overall** |  |  |  | 140 | (381) | 36.7 | 31.9 - 41.8 | 68 | (191) | 35.6 | 28.8 - 42.8 | | 72 | (190) | 37.9 | 31.0 - 45.2 |
|  | **Q2 2021** | 01 June 2021 | – | 30 Jun 2021 | 3 | (8) | 37.5 | 8.5 - 75.5 | 0 | (1) | 0.0 | 0.0 - 97.5 | | 3 | (7) | 42.9 | 9.9 - 81.6 |
|  | **Q3 2021** | 01 Jul 2021 | – | 30 Sep 2021 | 19 | (56) | 33.9 | 21.8 - 47.8 | 6 | (25) | 24.0 | 9.4 - 45.1 | | 13 | (31) | 41.9 | 24.5 - 60.9 |
|  | **Q4 2021** | 01 Oct 2021 | – | 31 Dec 2021 | 33 | (115) | 28.7 | 20.6 - 37.9 | 11 | (53) | 20.8 | 10.8 - 34.1 | | 22 | (62) | 35.5 | 23.7 - 48.7 |
|  | **Q1 2022** | 01 Jan 2022 | – | 31 Mar 2022 | 20 | (60) | 33.3 | 21.7 - 46.7 | 15 | (48) | 31.2 | 18.7 - 46.3 | | 5 | (12) | 41.7 | 15.2 - 72.3 |
|  | **Q2 2022** | 01 Apr 2021 | – | 30 Jun 2021 | 13 | (29) | 44.8 | 26.4 - 64.3 | 13 | (24) | 54.2 | 32.8 - 74.4 | | 0 | (5) | 0.0 | 0.0 - 52.2 |
|  | **Q3 2022** | 01 Jul 2022 | – | 30 Sep 2022 | 15 | (27) | 55.6 | 35.3 - 74.5 | 11 | (17) | 64.7 | 38.3 - 85.8 | | 4 | (10) | 40.0 | 12.2 - 73.8 |
|  | **Q4 2022** | 01 Oct 2022 | – | 31 Dec 2022 | 21 | (37) | 56.8 | 39.5 - 72.9 | 9 | (18) | 50.0 | 26.0 - 74.0 | | 12 | (19) | 63.2 | 38.4 - 83.7 |
|  | **Q1 2023** | 01 Jan 2023 | – | 31 Mar 2023 | 8 | (31) | 25.8 | 11.9 - 44.6 | 1 | (2) | 50.0 | 1.3 - 98.7 | | 7 | (29) | 24.1 | 10.3 - 43.5 |
|  | **Q2 2023**† | 01 Apr 2023 | – | 31 May 2023 | 8 | (18) | 44.4 | 21.5 - 69.2 | 2 | (3) | 66.7 | 9.4 - 99.2 | | 6 | (15) | 40.0 | 16.3 - 67.7 |

^a^ Alternative definition of IC (Table 1); ^b^ Overall, the entire study time from 01 June 2021 to 31 May 2023; Time periods are divided according to fiscal quarters; ^c^ Main SARI definition (Table 1); ^d^ Main SARS-CoV-2 test status definition (Table 1); †, this fiscal quarter included 1 out of 3 months. Abbreviations: CI, confidence interval; IC, immunocompromising condition; ICU, intensive care unit; n, number of individuals with ICs; N, number of SARI patients; Q, calendar time in fiscal quarter; SARI, severe acute respiratory infection; SARS-CoV-2, severe acute respiratory syndrome coronavirus 2

Table S6. IC prevalence among hospitalized SARI patients (alternative WHO definition), overall by SARS-CoV-2 test status, and by calendar time

| **IC definition ^a^** | **Period ^b^** | | | | **All SARI ^c^** | | | | **SARS-CoV-2 test status ^d^** | | | | | | | | | |
| --- | --- | --- | --- | --- | --- | --- | --- | --- | --- | --- | --- | --- | --- | --- | --- | --- | --- | --- |
|  |  |  |  |  |  |  |  |  | **Positive** | | | | **Negative** | | | | | |
|  |  |  |  |  |  |  | **Prevalence** | |  |  | **Prevalence** | |  |  | | | **Prevalence** | |
|  |  |  |  |  | **n** | **(N)** | **%** | **95% CI** | **n** | **(N)** | **%** | **95% CI** | **n** | | **(N)** | **%** | | **95% CI** |
| **Main**  **definition** | **Overall** |  |  |  | 262 | (2,173) | 12.1 | 10.7 - 13.5 | 103 | (764) | 13.5 | 11.1 - 16.1 | 159 | | (1,409) | 11.3 | | 9.7 - 13.1 |
|  | **Q2 2021** | 01 June 2021 | – | 30 Jun 2021 | 3 | (32) | 9.4 | 2 - 25 | 0 | (11) | 0 | 0 - 28.5 | 3 | | (21) | 14.3 | | 3 - 36.3 |
|  | **Q3 2021** | 01 Jul 2021 | – | 30 Sep 2021 | 14 | (93) | 15.1 | 8.5 - 24 | 6 | (65) | 9.2 | 3.5 - 19 | 8 | | (28) | 28.6 | | 13.2 - 48.7 |
|  | **Q4 2021** | 01 Oct 2021 | – | 31 Dec 2021 | 38 | (257) | 14.8 | 10.7 - 19.7 | 21 | (139) | 15.1 | 9.6 - 22.2 | 17 | | (118) | 14.4 | | 8.6 - 22.1 |
|  | **Q1 2022** | 01 Jan 2022 | – | 31 Mar 2022 | 34 | (491) | 6.9 | 4.8 - 9.5 | 20 | (205) | 9.8 | 6.1 - 14.7 | 14 | | (286) | 4.9 | | 2.7 - 8.1 |
|  | **Q2 2022** | 01 Apr 2021 | – | 30 Jun 2021 | 29 | (265) | 10.9 | 7.5 - 15.3 | 19 | (120) | 15.8 | 9.8 - 23.6 | 10 | | (145) | 6.9 | | 3.4 - 12.3 |
|  | **Q3 2022** | 01 Jul 2022 | – | 30 Sep 2022 | 28 | (155) | 18.1 | 12.4 - 25 | 13 | (71) | 18.3 | 10.1 - 29.3 | 15 | | (84) | 17.9 | | 10.4 - 27.7 |
|  | **Q4 2022** | 01 Oct 2022 | – | 31 Dec 2022 | 54 | (394) | 13.7 | 10.5 - 17.5 | 16 | (83) | 19.3 | 11.4 - 29.4 | 38 | | (311) | 12.2 | | 8.8 - 16.4 |
|  | **Q1 2023** | 01 Jan 2023 | – | 31 Mar 2023 | 43 | (377) | 11.4 | 8.4 - 15.1 | 6 | (57) | 10.5 | 4 - 21.5 | 37 | | (320) | 11.6 | | 8.3 - 15.6 |
|  | **Q2 2023**† | 01 Apr 2023 | – | 31 May 2023 | 19 | (109) | 17.4 | 10.8 - 25.9 | 2 | (13) | 15.4 | 1.9 - 45.4 | 17 | | (96) | 17.7 | | 10.7 - 26.8 |
| **Alternative**  **definition** | **Overall** |  |  |  | 599 | (2,173) | 27.6 | 25.7 - 29.5 | 211 | (764) | 27.6 | 24.5 - 30.9 | 388 | | (1,409) | 27.5 | | 25.2 - 30 |
|  | **Q2 2021** | 01 June 2021 | – | 30 Jun 2021 | 5 | (32) | 15.6 | 5.3 - 32.8 | 0 | (11) | 0 | 0 - 28.5 | 5 | | (21) | 23.8 | | 8.2 - 47.2 |
|  | **Q3 2021** | 01 Jul 2021 | – | 30 Sep 2021 | 19 | (93) | 20.4 | 12.8 - 30.1 | 10 | (65) | 15.4 | 7.6 - 26.5 | 9 | | (28) | 32.1 | | 15.9 - 52.4 |
|  | **Q4 2021** | 01 Oct 2021 | – | 31 Dec 2021 | 66 | (257) | 25.7 | 20.5 - 31.5 | 31 | (139) | 22.3 | 15.7 - 30.1 | 35 | | (118) | 29.7 | | 21.6 - 38.8 |
|  | **Q1 2022** | 01 Jan 2022 | – | 31 Mar 2022 | 118 | (491) | 24 | 20.3 - 28.1 | 55 | (205) | 26.8 | 20.9 - 33.4 | 63 | | (286) | 22 | | 17.4 - 27.3 |
|  | **Q2 2022** | 01 Apr 2021 | – | 30 Jun 2021 | 77 | (265) | 29.1 | 23.7 - 34.9 | 43 | (120) | 35.8 | 27.3 - 45.1 | 34 | | (145) | 23.4 | | 16.8 - 31.2 |
|  | **Q3 2022** | 01 Jul 2022 | – | 30 Sep 2022 | 48 | (155) | 31 | 23.8 - 38.9 | 24 | (71) | 33.8 | 23 - 46 | 24 | | (84) | 28.6 | | 19.2 - 39.5 |
|  | **Q4 2022** | 01 Oct 2022 | – | 31 Dec 2022 | 113 | (394) | 28.7 | 24.3 - 33.4 | 27 | (83) | 32.5 | 22.6 - 43.7 | 86 | | (311) | 27.7 | | 22.8 - 33 |
|  | **Q1 2023** | 01 Jan 2023 | – | 31 Mar 2023 | 111 | (377) | 29.4 | 24.9 - 34.3 | 16 | (57) | 28.1 | 17 - 41.5 | 95 | | (320) | 29.7 | | 24.7 - 35 |
|  | **Q2 2023**† | 01 Apr 2023 | – | 31 May 2023 | 42 | (109) | 38.5 | 29.4 - 48.3 | 5 | (13) | 38.5 | 13.9 - 68.4 | 37 | | (96) | 38.5 | | 28.8 - 49 |

^a^ Main and alternative definition of IC (Table 1); ^b^ Overall, the entire study time from 01 June 2021 to 31 May 2023; Time periods are divided according to fiscal quarters; ^c^ Alternative SARI definition (Table 1); ^d^ Main SARS-CoV-2 test status definition (Table 1); †, this fiscal quarter included 1 out of 3 months. Abbreviations: CI, confidence interval; IC, immunocompromising condition; n, number of individuals with ICs; N, number of SARI patients; Q, calendar time in fiscal quarter; SARI, severe acute respiratory infection; SARS-CoV-2, severe acute respiratory syndrome coronavirus 2

Table S7. Demographics, clinical outcomes of study population, overall, by IC status (alternative definition), and by SARS-CoV-2 test status

| **Covariates** | **All SARI ^c^** | | | **SARS-CoV-2 test status ^d^** | | | | | | |
| --- | --- | --- | --- | --- | --- | --- | --- | --- | --- | --- |
|  |  |  |  | **Positive** | | | | **Negative** | | |
|  | **Overall**  **(N=5,280)** | **IC a**  **(N=1,521)** | **Non-IC**  **(N=3,759)** | **Overall**  **(N=1,924)** | **IC a**  **(N=570)** | | **Non-IC  (N=1,354)** | **Overall (N=3,356)** | **IC a  (N=951)** | **Non-IC  (N=2,405)** |
| **DEMOGRAPHICS** | | | | | | | | | | |
| **Age (years)** |  |  |  |  |  | |  |  |  |  |
| Mean (SD) | 67.5 (17.0) | 67.7 (15.0) | 67.5 (17.7) | 66.9 (16.6) | 68.1 (14.7) | | 66.5 (17.4) | 67.9 (17.1) | 67.4 (15.2) | 68.0 (17.8) |
| Median  (IQR) | 71.0  (58.0, 80.0) | 70.0  (59.0, 78.0) | 71.0  (57.0, 81.0) | 70.0  (57.0, 79.0) | 71.0  (59.0, 78.0) | | 70.0  (55.0, 79.8) | 71.0  (58.0, 81.0) | 69.0  (59.0, 78.0) | 72.0  (58.0, 82.0) |
| Range | 18.0 - 103.0 | 18.0 - 99.0 | 18.0 - 103.0 | 18.0 - 102.0 | 21.0 - 98.0 | | 18.0 - 102.0 | 18.0 - 103.0 | 18.0 - 99.0 | 18.0 - 103.0 |
| **Sex, n (%)** |  |  |  |  |  | |  |  |  |  |
| Male | 2,982 (56.5) | 912 (60.0) | 2,070 (55.1) | 1,119 (58.2) | 336 (58.9) | | 783 (57.8) | 1,863 (55.5) | 576 (60.6) | 1,287 (53.5) |
| Female | 2,298 (43.5) | 609 (40.0) | 1,689 (44.9) | 805 (41.8) | 234 (41.1) | | 571 (42.2) | 1,493 (44.5) | 375 (39.4) | 1,118 (46.5) |
| **Country, n (%)** |  |  |  |  |  | |  |  |  |  |
| Belgium | 568 (10.8) | 127 (8.3) | 441 (11.7) | 306 (15.9) | 77 (13.5) | | 229 (16.9) | 262 (7.8) | 50 (5.3) | 212 (8.8) |
| Italy | 1,289 (24.4) | 298 (19.6) | 991 (26.4) | 481 (25.0) | 115 (20.2) | | 366 (27.0) | 808 (24.1) | 183 (19.2) | 625 (26.0) |
| Spain | 3,423 (64.8) | 1,096 (72.1) | 2,327 (61.9) | 1,137 (59.1) | 378 (66.3) | | 759 (56.1) | 2,286 (68.1) | 718 (75.5) | 1,568 (65.2) |
| **Study site, n (%)** |  |  |  |  |  | |  |  |  |  |
| CIRI-IT | 1,289 (24.4) | 298 (19.6) | 991 (26.4) | 481 (25.0) | 115 (20.2) | | 366 (27.0) | 808 (24.1) | 183 (19.2) | 625 (26.0) |
| GTPUH | 1,744 (33.0) | 481 (31.6) | 1,263 (33.6) | 459 (23.9) | 101 (17.7) | | 358 (26.4) | 1,285 (38.3) | 380 (40.0) | 905 (37.6) |
| CHU Saint-Pierre | 173 (3.3) | 32 (2.1) | 141 (3.8) | 52 (2.7) | 9 (1.6) | | 43 (3.2) | 121 (3.6) | 23 (2.4) | 98 (4.1) |
| UZA | 395 (7.5) | 95 (6.2) | 300 (8.0) | 254 (13.2) | 68 (11.9) | | 186 (13.7) | 141 (4.2) | 27 (2.8) | 114 (4.7) |
| HUVH | 1,679 (31.8) | 615 (40.4) | 1,064 (28.3) | 678 (35.2) | 277 (48.6) | | 401 (29.6) | 1,001 (29.8) | 338 (35.5) | 663 (27.6) |
| **Smoking, n (%)** |  |  |  |  |  | |  |  |  |  |
| Never smoker | 2,009 (38.0) | 546 (35.9) | 1,463 (38.9) | 760 (39.5) | 216 (37.9) | | 544 (40.2) | 1,249 (37.2) | 330 (34.7) | 919 (38.2) |
| Former smoker | 1,347 (25.5) | 488 (32.1) | 859 (22.9) | 468 (24.3) | 156 (27.4) | | 312 (23.0) | 879 (26.2) | 332 (34.9) | 547 (22.7) |
| Current smoker | 870 (16.5) | 216 (14.2) | 654 (17.4) | 227 (11.8) | 71 (12.5) | | 156 (11.5) | 643 (19.2) | 145 (15.2) | 498 (20.7) |
| Missing | 1,054 (20.0) | 271 (17.8) | 783 (20.8) | 469 (24.4) | 127 (22.3) | | 342 (25.3) | 585 (17.4) | 144 (15.1) | 441 (18.3) |
| **Long-term care facility residence, n (%)** |  |  |  |  |  | |  |  |  |  |
| Yes | 201 (3.9) | 42 (2.8) | 159 (4.3) | 59 (3.1) | 14 (2.6) | 45 (3.4) | | 142 (4.3) | 28 (3.0) | 114 (4.9) |
| **VACCINATION STATUS AT TIME OF HOSPITAL ADMISSION** | | | | | | | | | | |
| Unvaccinated, n (%) | 641 (12.1) | 94 (6.2) | 547 (14.6) | 417 (21.7) | 46 (8.1) | | 371 (27.4) | 224 (6.7) | 48 (5.0) | 176 (7.3) |
| Incomplete primary series, n (%) | 134 (2.5) | 31 (2.0) | 103 (2.7) | 48 (2.5) | 12 (2.1) | | 36 (2.7) | 86 (2.6) | 19 (2.0) | 67 (2.8) |
| Primary series completed but no boosters, n (%) | 1,309 (24.8) | 355 (23.3) | 954 (25.4) | 454 (23.6) | 111 (19.5) | | 343 (25.3) | 855 (25.5) | 244 (25.7) | 611 (25.4) |
| At least one booster dose, n (%) | 3,196 (60.5) | 1,041 (68.4) | 2,155 (57.3) | 1,005 (52.2) | 401 (70.4) | | 604 (44.6) | 2,191 (65.3) | 640 (67.3) | 1,551 (64.5) |
| **Number of booster doses^b^, n (%)** |  |  |  |  |  | |  |  |  |  |
| 0 | 1,309 (29.1) | 355 (25.4) | 954 (30.7) | 454 (31.1) | 111 (21.7) | | 343 (36.2) | 855 (28.1) | 244 (27.6) | 611 (28.3) |
| 1 | 2,375 (52.7) | 732 (52.4) | 1,643 (52.8) | 794 (54.4) | 298 (58.2) | | 496 (52.4) | 1,581 (51.9) | 434 (49.1) | 1,147 (53.1) |
| 2 | 775 (17.2) | 277 (19.8) | 498 (16.0) | 195 (13.4) | 88 (17.2) | | 107 (11.3) | 580 (19.0) | 189 (21.4) | 391 (18.1) |
| 3+ | 46 (1.0) | 32 (2.3) | 14 (0.5) | 16 (1.1) | 15 (2.9) | | 1 (0.1) | 30 (1.0) | 17 (1.9) | 13 (0.6) |
| **Time since last vaccine dose (days)** |  |  |  |  |  | |  |  |  |  |
| Mean (SD) | 187.8 (138.8) | 192.6 (139.6) | 185.7 (138.4) | 175.7 (115.8) | 182.4 (118.3) | | 172.1 (114.4) | 193.6 (148.2) | 198.5 (150.4) | 191.7 (147.4) |
| Median  (IQR) | 156.0  (81.0, 260.0) | 162.0  (85.0, 267.0) | 153.0  (79.0, 258.0) | 163.0  (89.0, 233.5) | 163.5  (92.8, 249.2) | | 163.0  (86.5, 227.0) | 153.0  (77.8, 287.0) | 159.0  (80.5, 284.5) | 150.0  (76.0, 288.0) |
| Range | 1.0 - 782.0 | 1.0 - 755.0 | 1.0 - 782.0 | 1.0 - 662.0 | 1.0 - 662.0 | | 1.0 - 653.0 | 1.0 - 782.0 | 1.0 - 755.0 | 1.0 - 782.0 |
| **Time since last vaccine dose, n (%)** |  |  |  |  |  | |  |  |  |  |
| <2 months | 798 (17.2) | 227 (15.9) | 571 (17.8) | 225 (14.9) | 73 (13.9) | | 152 (15.5) | 573 (18.3) | 154 (17.1) | 419 (18.8) |
| [2 - 4) months | 970 (20.9) | 298 (20.9) | 672 (20.9) | 316 (21.0) | 117 (22.3) | | 199 (20.2) | 654 (20.9) | 181 (20.0) | 473 (21.2) |
| [4 - 6) months | 916 (19.7) | 271 (19.0) | 645 (20.1) | 325 (21.6) | 99 (18.9) | | 226 (23.0) | 591 (18.9) | 172 (19.0) | 419 (18.8) |
| [6 - 8) months | 637 (13.7) | 206 (14.4) | 431 (13.4) | 286 (19.0) | 92 (17.6) | | 194 (19.7) | 351 (11.2) | 114 (12.6) | 237 (10.6) |
| ≥8 months | 1,318 (28.4) | 425 (29.8) | 893 (27.8) | 355 (23.6) | 143 (27.3) | | 212 (21.6) | 963 (30.7) | 282 (31.2) | 681 (30.6) |
| **SYMPTOMS** | | | | | | | | | | |
| **SARI symptoms ^e,f^, n (%)** |  |  |  |  |  | |  |  |  |  |
| Cough | 3,577 (71.4) | 961 (66.3) | 2,616 (73.5) | 1,246 (73.2) | 340 (66.7) | | 906 (76.0) | 2,331 (70.5) | 621 (66.1) | 1,710 (72.2) |
| Fever | 3,100 (62.2) | 942 (65.1) | 2,158 (61.0) | 1,110 (65.3) | 345 (67.5) | | 765 (64.4) | 1,990 (60.5) | 597 (63.8) | 1,393 (59.3) |
| Shortness of breath | 3,524 (70.1) | 951 (65.5) | 2,573 (71.9) | 1,235 (71.9) | 335 (65.3) | | 900 (74.7) | 2,289 (69.2) | 616 (65.7) | 1,673 (70.5) |
| Anosmia, ageusia or dysgeusia | 172 (3.6) | 47 (3.4) | 125 (3.7) | 140 (9.0) | 34 (7.3) | | 106 (9.7) | 32 (1.0) | 13 (1.4) | 19 (0.8) |
| **Number of SARI symptoms, n (%)** |  |  |  |  |  | |  |  |  |  |
| 1 | 1,152 (24.5) | 396 (28.9) | 756 (22.7) | 374 (24.1) | 139 (30.1) | | 235 (21.5) | 778 (24.7) | 257 (28.3) | 521 (23.2) |
| 2 | 2,215 (47.1) | 619 (45.2) | 1,596 (47.9) | 611 (39.3) | 186 (40.3) | | 425 (39.0) | 1,604 (50.9) | 433 (47.7) | 1,171 (52.2) |
| 3+ | 1,336 (28.4) | 354 (25.9) | 982 (29.5) | 568 (36.6) | 137 (29.7) | | 431 (39.5) | 768 (24.4) | 217 (23.9) | 551 (24.6) |
| **HOSPITAL OUTCOMES** | | | | | | | | | | |
| **Length of stay, (days)** |  |  |  |  |  | |  |  |  |  |
| Mean (SD) | 10.1 (13.8) | 10.8 (14.3) | 9.9 (13.5) | 12.2 (18.0) | 13.1 (18.1) | | 11.9 (18.0) | 8.9 (10.4) | 9.4 (11.3) | 8.8 (10.1) |
| Median  (IQR) | 7.0  (4.0, 12.0) | 7.0  (4.0, 12.0) | 7.0  (4.0, 12.0) | 7.0  (4.0, 14.0) | 7.0  (4.0, 14.8) | | 7.0  (4.0, 14.0) | 6.0  (3.0, 11.0) | 6.0  (4.0, 11.0) | 6.0  (3.0, 11.0) |
| Range | 1.0 - 403.0 | 1.0 - 173.0 | 1.0 - 403.0 | 1.0 - 403.0 | 1.0 - 146.0 | | 1.0 - 403.0 | 1.0 - 173.0 | 1.0 - 173.0 | 1.0 - 166.0 |
| **Severe outcomes, n (%)** |  |  |  |  |  | |  |  |  |  |
| Hospitalization without ICU | 4,564 (86.5) | 1,315 (86.5) | 3,249 (86.5) | 1,525 (79.3) | 472 (82.8) | | 1,053 (77.8) | 3,039 (90.6) | 843 (88.6) | 2,196 (91.3) |
| ICU admission without in-hospital death | 334 (6.3) | 66 (4.3) | 268 (7.1) | 207 (10.8) | 30 (5.3) | | 177 (13.1) | 127 (3.8) | 36 (3.8) | 91 (3.8) |
| ICU admission (with or without in-hospital death) | 435 (8.2) | 104 (6.8) | 331 (8.8) | 286 (14.9) | 58 (10.2) | | 228 (16.8) | 149 (4.4) | 46 (4.8) | 103 (4.3) |
| In-hospital death | 381 (7.2) | 140 (9.2) | 241 (6.4) | 191 (9.9) | 68 (11.9) | | 123 (9.1) | 190 (5.7) | 72 (7.6) | 118 (4.9) |
| **COMORBIDITIES** | | | | | | | | | | |
| **Number of comorbidities other than IC, n (%)** |  |  |  |  |  | |  |  |  |  |
| No comorbid conditions | 968 (18.3) | 64 (4.2) | 904 (24.0) | 386 (20.1) | 23 (4.0) | | 363 (26.8) | 582 (17.3) | 41 (4.3) | 541 (22.5) |
| At least 1 comorbidity | 1,157 (21.9) | 342 (22.5) | 815 (21.7) | 419 (21.8) | 143 (25.1) | | 276 (20.4) | 738 (22.0) | 199 (20.9) | 539 (22.4) |
| 2 comorbidities | 1,191 (22.6) | 339 (22.3) | 852 (22.7) | 471 (24.5) | 135 (23.7) | | 336 (24.8) | 720 (21.5) | 204 (21.5) | 516 (21.5) |
| 3+ comorbidities | 1,964 (37.2) | 776 (51.0) | 1,188 (31.6) | 648 (33.7) | 269 (47.2) | | 379 (28.0) | 1,316 (39.2) | 507 (53.3) | 809 (33.6) |
| **Number with specific comorbidities, n (%)** |  |  |  |  |  | |  |  |  |  |
| Asthma | 401 (7.6) | 88 (5.8) | 313 (8.3) | 116 (6.0) | 26 (4.6) | | 90 (6.6) | 285 (8.5) | 62 (6.5) | 223 (9.3) |
| Lung disease | 1,737 (32.9) | 575 (37.8) | 1,162 (30.9) | 513 (26.7) | 185 (32.5) | | 328 (24.2) | 1,224 (36.5) | 390 (41.0) | 834 (34.7) |
| Cardiovascular disease | 2,106 (39.9) | 605 (39.8) | 1,501 (39.9) | 743 (38.6) | 222 (38.9) | | 521 (38.5) | 1,363 (40.6) | 383 (40.3) | 980 (40.7) |
| Hypertension | 2,623 (49.7) | 728 (47.9) | 1,895 (50.4) | 966 (50.2) | 273 (47.9) | | 693 (51.2) | 1,657 (49.4) | 455 (47.8) | 1,202 (50.0) |
| Chronic liver disease | 399 (7.6) | 153 (10.1) | 246 (6.5) | 121 (6.3) | 52 (9.1) | | 69 (5.1) | 278 (8.3) | 101 (10.6) | 177 (7.4) |
| Chronic kidney disease | 904 (17.1) | 320 (21.0) | 584 (15.5) | 344 (17.9) | 126 (22.1) | | 218 (16.1) | 560 (16.7) | 194 (20.4) | 366 (15.2) |
| Type 2 diabetes | 1,390 (26.3) | 395 (26.0) | 995 (26.5) | 503 (26.1) | 147 (25.8) | | 356 (26.3) | 887 (26.4) | 248 (26.1) | 639 (26.6) |
| Cancer | 1,172 (22.2) | 1,172 (77.1) | 0 (0.0) | 424 (22.0) | 424 (74.4) | | 0 (0.0) | 748 (22.3) | 748 (78.7) | 0 (0.0) |
| Main IC ^g^ | 732 (13.9) | 732 (48.1) | 0 (0.0) | 274 (14.2) | 274 (48.1) | | 0 (0.0) | 458 (13.6) | 458 (48.2) | 0 (0.0) |

^a^ Alternative definition of IC (Table 1); ^b^ Among the patients eligible to receive booster (completed primary series); ^c^ Main SARI definition (Table 1); ^d^ Main SARS-CoV-2 test status definition (Table 1); ^e^ Not mutually exclusive; ^f^ The recording of individual SARI symptoms became mandatory after COVIDRIVE master protocol v4.0 (October 10, 2022), therefore records of SARI patients admitted to hospital before this date may lack that information; ^g^ Main definition of IC (Table 1). Abbreviations: CI, confidence interval; CIRI-IT, Centro Interuniversitario di Ricerca sull'Influenza e le altre Infezioni Trasmissibili; GTPUH, Hospital Universitario Germans Trias i Pujol; HUVH, Hospital Universitari Vall d’Hebron; IC, immunocompromising condition; ICU, intensive care unit; IQR, interquartile range; n, number; SARI, severe acute respiratory infection; SARS-CoV-2, severe acute respiratory syndrome coronavirus 2; SD, standard deviation; UZA, Universitair Ziekenhuis Antwerpen. A square bracket “[“ and “]” in a range denotes that the number is included in the range, while regular brackets “(“ and “)” in a range means that the number is not included.

Table S8. Demographic and clinical characteristics of hospitalized SARI patients in three European countries, by SARS-CoV-2 test status, by IC status (main definition), and by calendar time

| **Covariates per period ^b^** | **SARI ^f^** | | | **SARS-CoV-2 test status ^g^** | | | | | |
| --- | --- | --- | --- | --- | --- | --- | --- | --- | --- |
|  |  |  |  | **Positive** | | | **Negative** | | |
|  | **Overall (N=5,280)** | **IC ^a^**  **(N=1,521)** | **Non-IC (N=3,759)** | **Overall  (N=1,924)** | **IC ^a^**  **(N=570)** | **Non-IC (N=1,354)** | **Overall (N=3,356)** | **IC ^a^**  **(N=951)** | **Non-IC (N=2,405)** |
| **Q2 2021** |  |  |  |  |  |  |  |  |  |
| **Total** | **96** | **8** | **88** | **29** | **1** | **28** | **67** | **7** | **60** |
| **DEMOGRAPHICS** | | | | | | | | | |
| **Age (years)** |  |  |  |  |  |  |  |  |  |
| Mean (SD) | 64.2 (18.8) | 57.0 (14.9) | 64.9 (19.0) | 49.9 (16.2) | 54.0 (NA) | 49.8 (16.5) | 70.4 (16.4) | 57.4 (16.0) | 72.0 (15.9) |
| Median  (IQR) | 68.5  (48.5, 80.0) | 58.0  (46.5, 66.0) | 70.0  (49.8, 81.0) | 47.0  (41.0, 61.0) | 54.0  (54.0, 54.0) | 47.0  (40.8, 61.2) | 74.0  (64.0, 82.0) | 62.0  (46.0, 67.0) | 75.0  (65.0, 83.2) |
| Range | 22.0 - 97.0 | 34.0 - 80.0 | 22.0 - 97.0 | 22.0 - 89.0 | 54.0 - 54.0 | 22.0 - 89.0 | 27.0 - 97.0 | 34.0 - 80.0 | 27.0 - 97.0 |
| **Sex, n (%)** |  |  |  |  |  |  |  |  |  |
| Male | 58 (60.4) | 7 (87.5) | 51 (58.0) | 18 (62.1) | 1 (100.0) | 17 (60.7) | 40 (59.7) | 6 (85.7) | 34 (56.7) |
| Female | 38 (39.6) | 1 (12.5) | 37 (42.0) | 11 (37.9) | 0 (0.0) | 11 (39.3) | 27 (40.3) | 1 (14.3) | 26 (43.3) |
| **Country, n (%)** |  |  |  |  |  |  |  |  |  |
| Belgium | 16 (16.7) | 0 (0.0) | 16 (18.2) | 7 (24.1) | 0 (0.0) | 7 (25.0) | 9 (13.4) | 0 (0.0) | 9 (15.0) |
| Italy | 0 (0.0) | 0 (0.0) | 0 (0.0) | 0 (0.0) | 0 (0.0) | 0 (0.0) | 0 (0.0) | 0 (0.0) | 0 (0.0) |
| Spain | 80 (83.3) | 8 (100.0) | 72 (81.8) | 22 (75.9) | 1 (100.0) | 21 (75.0) | 58 (86.6) | 7 (100.0) | 51 (85.0) |
| **Study site, n (%)** |  |  |  |  |  |  |  |  |  |
| CIRI-IT | 0 (0.0) | 0 (0.0) | 0 (0.0) | 0 (0.0) | 0 (0.0) | 0 (0.0) | 0 (0.0) | 0 (0.0) | 0 (0.0) |
| GTPUH | 80 (83.3) | 8 (100.0) | 72 (81.8) | 22 (75.9) | 1 (100.0) | 21 (75.0) | 58 (86.6) | 7 (100.0) | 51 (85.0) |
| CHU Saint-Pierre | 3 (3.1) | 0 (0.0) | 3 (3.4) | 1 (3.4) | 0 (0.0) | 1 (3.6) | 2 (3.0) | 0 (0.0) | 2 (3.3) |
| UZA | 13 (13.5) | 0 (0.0) | 13 (14.8) | 6 (20.7) | 0 (0.0) | 6 (21.4) | 7 (10.4) | 0 (0.0) | 7 (11.7) |
| HUVH | 0 (0.0) | 0 (0.0) | 0 (0.0) | 0 (0.0) | 0 (0.0) | 0 (0.0) | 0 (0.0) | 0 (0.0) | 0 (0.0) |
| **Smoking, n (%)** |  |  |  |  |  |  |  |  |  |
| Never smoker | 41 (42.7) | 2 (25.0) | 39 (44.3) | 14 (48.3) | 0 (0.0) | 14 (50.0) | 27 (40.3) | 2 (28.6) | 25 (41.7) |
| Former smoker | 24 (25.0) | 4 (50.0) | 20 (22.7) | 5 (17.2) | 1 (100.0) | 4 (14.3) | 19 (28.4) | 3 (42.9) | 16 (26.7) |
| Current smoker | 20 (20.8) | 2 (25.0) | 18 (20.5) | 3 (10.3) | 0 (0.0) | 3 (10.7) | 17 (25.4) | 2 (28.6) | 15 (25.0) |
| Missing | 11 (11.5) | 0 (0.0) | 11 (12.5) | 7 (24.1) | 0 (0.0) | 7 (25.0) | 4 (6.0) | 0 (0.0) | 4 (6.7) |
| **Long-term care facility residence, n (%)** |  |  |  |  |  |  |  |  |  |
| Yes | 4 (4.3) | 0 (0.0) | 4 (4.7) | 0 (0.0) | 0 (0.0) | 0 (0.0) | 4 (6.2) | 0 (0.0) | 4 (6.9) |
| **VACCINATION STATUS AT TIME OF HOSPITAL ADMISSION** | | | | | | | | | |
| Unvaccinated, n (%) | 31 (32.3) | 2 (25.0) | 29 (33.0) | 18 (62.1) | 0 (0.0) | 18 (64.3) | 13 (19.4) | 2 (28.6) | 11 (18.3) |
| Incomplete primary series, n (%) | 20 (20.8) | 3 (37.5) | 17 (19.3) | 6 (20.7) | 1 (100.0) | 5 (17.9) | 14 (20.9) | 2 (28.6) | 12 (20.0) |
| Primary series completed but no boosters, n (%) | 45 (46.9) | 3 (37.5) | 42 (47.7) | 5 (17.2) | 0 (0.0) | 5 (17.9) | 40 (59.7) | 3 (42.9) | 37 (61.7) |
| At least one booster dose, n (%) | 0 (0.0) | 0 (0.0) | 0 (0.0) | 0 (0.0) | 0 (0.0) | 0 (0.0) | 0 (0.0) | 0 (0.0) | 0 (0.0) |
| **Number of booster doses ^c^, n (%)** |  |  |  |  |  |  |  |  |  |
| 0 | 45 (100.0) | 3 (100.0) | 42 (100.0) | 5 (100.0) | 0 | 5 (100.0) | 40 (100.0) | 3 (100.0) | 37 (100.0) |
| 1 | 0 (0.0) | 0 (0.0) | 0 (0.0) | 0 (0.0) | 0 | 0 (0.0) | 0 (0.0) | 0 (0.0) | 0 (0.0) |
| 2 | 0 (0.0) | 0 (0.0) | 0 (0.0) | 0 (0.0) | 0 | 0 (0.0) | 0 (0.0) | 0 (0.0) | 0 (0.0) |
| 3+ | 0 (0.0) | 0 (0.0) | 0 (0.0) | 0 (0.0) | 0 | 0 (0.0) | 0 (0.0) | 0 (0.0) | 0 (0.0) |
| **Time since last vaccine dose (days)** |  |  |  |  |  |  |  |  |  |
| Mean (SD) | 43.9 (28.8) | 36.8 (24.6) | 44.7 (29.3) | 31.0 (29.8) | 23.0 (NA) | 31.8 (31.3) | 46.6 (28.1) | 39.6 (26.4) | 47.3 (28.5) |
| Median  (IQR) | 39.0  (19.0, 67.0) | 28.5  (23.8, 36.2) | 42.0  (18.5, 68.0) | 15.0  (12.0, 37.5) | 23.0  (23.0, 23.0) | 14.5  (11.5, 43.8) | 42.0  (26.0, 68.5) | 31.0  (26.0, 38.0) | 43.0  (26.0, 69.0) |
| Range | 1.0 - 130.0 | 18.0 - 85.0 | 1.0 - 130.0 | 9.0 - 98.0 | 23.0 - 23.0 | 9.0 - 98.0 | 1.0 - 130.0 | 18.0 - 85.0 | 1.0 - 130.0 |
| **Time since last vaccine dose, n (%)** |  |  |  |  |  |  |  |  |  |
| <2 months | 45 (69.2) | 5 (83.3) | 40 (67.8) | 9 (81.8) | 1 (100.0) | 8 (80.0) | 36 (66.7) | 4 (80.0) | 32 (65.3) |
| [2 - 4) months | 18 (27.7) | 1 (16.7) | 17 (28.8) | 2 (18.2) | 0 (0.0) | 2 (20.0) | 16 (29.6) | 1 (20.0) | 15 (30.6) |
| [4 - 6) months | 2 (3.1) | 0 (0.0) | 2 (3.4) | 0 (0.0) | 0 (0.0) | 0 (0.0) | 2 (3.7) | 0 (0.0) | 2 (4.1) |
| [6 - 8) months | 0 (0.0) | 0 (0.0) | 0 (0.0) | 0 (0.0) | 0 (0.0) | 0 (0.0) | 0 (0.0) | 0 (0.0) | 0 (0.0) |
| ≥8 months | 0 (0.0) | 0 (0.0) | 0 (0.0) | 0 (0.0) | 0 (0.0) | 0 (0.0) | 0 (0.0) | 0 (0.0) | 0 (0.0) |
| **SYMPTOMS** | | | | | | | | | |
| **SARI symptoms ^d,e^, n (%)** |  |  |  |  |  |  |  |  |  |
| Cough | 46 (48.4) | 3 (37.5) | 43 (49.4) | 20 (69.0) | 0 (0.0) | 20 (71.4) | 26 (39.4) | 3 (42.9) | 23 (39.0) |
| Fever | 77 (81.1) | 8 (100.0) | 69 (79.3) | 20 (71.4) | 1 (100.0) | 19 (70.4) | 57 (85.1) | 7 (100.0) | 50 (83.3) |
| Shortness of breath | 69 (71.9) | 5 (62.5) | 64 (72.7) | 20 (69.0) | 1 (100.0) | 19 (67.9) | 49 (73.1) | 4 (57.1) | 45 (75.0) |
| Anosmia, ageusia or dysgeusia | 6 (6.7) | 0 (0.0) | 6 (7.4) | 6 (22.2) | 0 (0.0) | 6 (23.1) | 0 (0.0) | 0 (0.0) | 0 (0.0) |
| **Number of SARI symptoms, n (%)** |  |  |  |  |  |  |  |  |  |
| 1 | 8 (9.1) | 1 (12.5) | 7 (8.8) | 1 (3.8) | 0 (0.0) | 1 (4.0) | 7 (11.3) | 1 (14.3) | 6 (10.9) |
| 2 | 64 (72.7) | 6 (75.0) | 58 (72.5) | 16 (61.5) | 1 (100.0) | 15 (60.0) | 48 (77.4) | 5 (71.4) | 43 (78.2) |
| 3+ | 16 (18.2) | 1 (12.5) | 15 (18.8) | 9 (34.6) | 0 (0.0) | 9 (36.0) | 7 (11.3) | 1 (14.3) | 6 (10.9) |
| **HOSPITAL OUTCOMES** | | | | | | | | | |
| **Length of stay (days)** |  |  |  |  |  |  |  |  |  |
| Mean (SD) | 9.4 (7.4) | 12.0 (8.5) | 9.2 (7.4) | 7.0 (4.2) | 6.0 (NA) | 7.1 (4.3) | 10.4 (8.3) | 12.9 (8.7) | 10.1 (8.3) |
| Median  (IQR) | 8.0  (4.8, 11.0) | 9.5  (5.8, 16.5) | 8.0  (4.0, 11.0) | 6.0  (4.0, 9.0) | 6.0  (6.0, 6.0) | 6.0  (4.0, 9.0) | 8.0  (5.0, 12.5) | 11.0  (6.5, 19.0) | 8.0  (5.0, 11.2) |
| Range | 1.0 - 50.0 | 3.0 - 25.0 | 1.0 - 50.0 | 2.0 - 18.0 | 6.0 - 6.0 | 2.0 - 18.0 | 1.0 - 50.0 | 3.0 - 25.0 | 1.0 - 50.0 |
| **Severe outcomes, n (%)** |  |  |  |  |  |  |  |  |  |
| Hospitalization without ICU admission or in-hospital death | 79 (82.3) | 7 (87.5) | 72 (81.8) | 21 (72.4) | 1 (100.0) | 20 (71.4) | 58 (86.6) | 6 (85.7) | 52 (86.7) |
| ICU admission without in-hospital death | 9 (9.4) | 0 (0.0) | 9 (10.2) | 7 (24.1) | 0 (0.0) | 7 (25.0) | 2 (3.0) | 0 (0.0) | 2 (3.3) |
| ICU admission (with or without in-hospital death) | 10 (10.4) | 0 (0.0) | 10 (11.4) | 8 (27.6) | 0 (0.0) | 8 (28.6) | 2 (3.0) | 0 (0.0) | 2 (3.3) |
| In-hospital death | 8 (8.3) | 1 (12.5) | 7 (8.0) | 1 (3.4) | 0 (0.0) | 1 (3.6) | 7 (10.4) | 1 (14.3) | 6 (10.0) |
| **COMORBIDITIES** | | | | | | | | | |
| **Number of comorbidities other than IC, n (%)** |  |  |  |  |  |  |  |  |  |
| No comorbid conditions | 23 (24.0) | 1 (12.5) | 22 (25.0) | 17 (58.6) | 0 (0.0) | 17 (60.7) | 6 (9.0) | 1 (14.3) | 5 (8.3) |
| 1 comorbidity | 17 (17.7) | 2 (25.0) | 15 (17.0) | 4 (13.8) | 0 (0.0) | 4 (14.3) | 13 (19.4) | 2 (28.6) | 11 (18.3) |
| 2 comorbidities | 21 (21.9) | 4 (50.0) | 17 (19.3) | 3 (10.3) | 1 (100.0) | 2 (7.1) | 18 (26.9) | 3 (42.9) | 15 (25.0) |
| 3+ comorbidities | 35 (36.5) | 1 (12.5) | 34 (38.6) | 5 (17.2) | 0 (0.0) | 5 (17.9) | 30 (44.8) | 1 (14.3) | 29 (48.3) |
| **Number with specific comorbidities ^d^, n (%)** |  |  |  |  |  |  |  |  |  |
| Asthma | 7 (7.3) | 0 (0.0) | 7 (8.0) | 0 (0.0) | 0 (0.0) | 0 (0.0) | 7 (10.4) | 0 (0.0) | 7 (11.7) |
| Lung disease | 22 (22.9) | 1 (12.5) | 21 (23.9) | 2 (6.9) | 0 (0.0) | 2 (7.1) | 20 (29.9) | 1 (14.3) | 19 (31.7) |
| Cardiovascular disease | 37 (38.5) | 2 (25.0) | 35 (39.8) | 5 (17.2) | 1 (100.0) | 4 (14.3) | 32 (47.8) | 1 (14.3) | 31 (51.7) |
| Hypertension | 53 (55.2) | 4 (50.0) | 49 (55.7) | 10 (34.5) | 1 (100.0) | 9 (32.1) | 43 (64.2) | 3 (42.9) | 40 (66.7) |
| Chronic liver disease | 9 (9.4) | 0 (0.0) | 9 (10.2) | 1 (3.4) | 0 (0.0) | 1 (3.6) | 8 (11.9) | 0 (0.0) | 8 (13.3) |
| Chronic kidney disease | 18 (18.8) | 1 (12.5) | 17 (19.3) | 4 (13.8) | 0 (0.0) | 4 (14.3) | 14 (20.9) | 1 (14.3) | 13 (21.7) |
| Type 2 diabetes | 27 (28.1) | 1 (12.5) | 26 (29.5) | 5 (17.2) | 0 (0.0) | 5 (17.9) | 22 (32.8) | 1 (14.3) | 21 (35.0) |
| Cancer | 15 (15.6) | 5 (62.5) | 10 (11.4) | 0 (0.0) | 0 (0.0) | 0 (0.0) | 15 (22.4) | 5 (71.4) | 10 (16.7) |
| Alternative IC ^h^ | 18 (18.8) | 8 (100.0) | 10 (11.4) | 1 (3.4) | 1 (100.0) | 0 (0.0) | 17 (25.4) | 7 (100.0) | 10 (16.7) |
| **Q3 2021** |  |  |  |  |  |  |  |  |  |
| **Total** | **378** | **52** | **326** | **186** | **18** | **168** | **192** | **34** | **158** |
| **DEMOGRAPHICS** | | | | | | | | | |
| **Age (years)** |  |  |  |  |  |  |  |  |  |
| Mean (SD) | 64.5 (18.4) | 63.9 (14.9) | 64.6 (18.9) | 58.8 (19.2) | 55.8 (17.0) | 59.1 (19.5) | 70.1 (15.6) | 68.3 (11.8) | 70.4 (16.3) |
| Median  (IQR) | 69.0  (50.0, 79.0) | 68.5  (55.0, 75.2) | 69.0  (49.2, 80.0) | 57.0  (44.0, 77.0) | 55.0  (44.2, 69.2) | 57.5  (43.8, 78.0) | 73.0  (62.0, 80.0) | 71.0  (62.5, 75.8) | 73.5  (62.2, 81.0) |
| Range | 18.0 - 99.0 | 22.0 - 85.0 | 18.0 - 99.0 | 18.0 - 95.0 | 22.0 - 79.0 | 18.0 - 95.0 | 19.0 - 99.0 | 31.0 - 85.0 | 19.0 - 99.0 |
| **Sex, n (%)** |  |  |  |  |  |  |  |  |  |
| Male | 224 (59.3) | 31 (59.6) | 193 (59.2) | 102 (54.8) | 8 (44.4) | 94 (56.0) | 122 (63.5) | 23 (67.6) | 99 (62.7) |
| Female | 154 (40.7) | 21 (40.4) | 133 (40.8) | 84 (45.2) | 10 (55.6) | 74 (44.0) | 70 (36.5) | 11 (32.4) | 59 (37.3) |
| **Country, n (%)** |  |  |  |  |  |  |  |  |  |
| Belgium | 100 (26.5) | 12 (23.1) | 88 (27.0) | 70 (37.6) | 11 (61.1) | 59 (35.1) | 30 (15.6) | 1 (2.9) | 29 (18.4) |
| Italy | 0 (0.0) | 0 (0.0) | 0 (0.0) | 0 (0.0) | 0 (0.0) | 0 (0.0) | 0 (0.0) | 0 (0.0) | 0 (0.0) |
| Spain | 278 (73.5) | 40 (76.9) | 238 (73.0) | 116 (62.4) | 7 (38.9) | 109 (64.9) | 162 (84.4) | 33 (97.1) | 129 (81.6) |
| **Study site, n (%)** |  |  |  |  |  |  |  |  |  |
| CIRI-IT | 0 (0.0) | 0 (0.0) | 0 (0.0) | 0 (0.0) | 0 (0.0) | 0 (0.0) | 0 (0.0) | 0 (0.0) | 0 (0.0) |
| GTPUH | 278 (73.5) | 40 (76.9) | 238 (73.0) | 116 (62.4) | 7 (38.9) | 109 (64.9) | 162 (84.4) | 33 (97.1) | 129 (81.6) |
| CHU Saint-Pierre | 26 (6.9) | 2 (3.8) | 24 (7.4) | 13 (7.0) | 2 (11.1) | 11 (6.5) | 13 (6.8) | 0 (0.0) | 13 (8.2) |
| UZA | 74 (19.6) | 10 (19.2) | 64 (19.6) | 57 (30.6) | 9 (50.0) | 48 (28.6) | 17 (8.9) | 1 (2.9) | 16 (10.1) |
| HUVH | 0 (0.0) | 0 (0.0) | 0 (0.0) | 0 (0.0) | 0 (0.0) | 0 (0.0) | 0 (0.0) | 0 (0.0) | 0 (0.0) |
| **Smoking, n (%)** |  |  |  |  |  |  |  |  |  |
| Never smoker | 129 (34.1) | 14 (26.9) | 115 (35.3) | 75 (40.3) | 2 (11.1) | 73 (43.5) | 54 (28.1) | 12 (35.3) | 42 (26.6) |
| Former smoker | 83 (22.0) | 16 (30.8) | 67 (20.6) | 18 (9.7) | 0 (0.0) | 18 (10.7) | 65 (33.9) | 16 (47.1) | 49 (31.0) |
| Current smoker | 52 (13.8) | 6 (11.5) | 46 (14.1) | 12 (6.5) | 3 (16.7) | 9 (5.4) | 40 (20.8) | 3 (8.8) | 37 (23.4) |
| Missing | 114 (30.2) | 16 (30.8) | 98 (30.1) | 81 (43.5) | 13 (72.2) | 68 (40.5) | 33 (17.2) | 3 (8.8) | 30 (19.0) |
| **Long-term care facility residence, n (%)** |  |  |  |  |  |  |  |  |  |
| Yes | 12 (3.3) | 1 (2.1) | 11 (3.5) | 4 (2.2) | 0 (0.0) | 4 (2.5) | 8 (4.3) | 1 (3.1) | 7 (4.6) |
| **VACCINATION STATUS AT TIME OF HOSPITAL ADMISSION** | | | | | | | | | |
| Unvaccinated, n (%) | 111 (29.4) | 8 (15.4) | 103 (31.6) | 90 (48.4) | 5 (27.8) | 85 (50.6) | 21 (10.9) | 3 (8.8) | 18 (11.4) |
| Incomplete primary series, n (%) | 23 (6.1) | 2 (3.8) | 21 (6.4) | 14 (7.5) | 0 (0.0) | 14 (8.3) | 9 (4.7) | 2 (5.9) | 7 (4.4) |
| Primary series completed but no boosters, n (%) | 244 (64.6) | 42 (80.8) | 202 (62.0) | 82 (44.1) | 13 (72.2) | 69 (41.1) | 162 (84.4) | 29 (85.3) | 133 (84.2) |
| At least one booster dose, n (%) | 0 (0.0) | 0 (0.0) | 0 (0.0) | 0 (0.0) | 0 (0.0) | 0 (0.0) | 0 (0.0) | 0 (0.0) | 0 (0.0) |
| **Number of booster doses ^c^, n (%)** |  |  |  |  |  |  |  |  |  |
| 0 | 244 (100.0) | 42 (100.0) | 202 (100.0) | 82 (100.0) | 13 (100.0) | 69 (100.0) | 162 (100.0) | 29 (100.0) | 133 (100.0) |
| 1 | 0 (0.0) | 0 (0.0) | 0 (0.0) | 0 (0.0) | 0 (0.0) | 0 (0.0) | 0 (0.0) | 0 (0.0) | 0 (0.0) |
| 2 | 0 (0.0) | 0 (0.0) | 0 (0.0) | 0 (0.0) | 0 (0.0) | 0 (0.0) | 0 (0.0) | 0 (0.0) | 0 (0.0) |
| 3+ | 0 (0.0) | 0 (0.0) | 0 (0.0) | 0 (0.0) | 0 (0.0) | 0 (0.0) | 0 (0.0) | 0 (0.0) | 0 (0.0) |
| **Time since last vaccine dose (days)** |  |  |  |  |  |  |  |  |  |
| Mean (SD) | 87.5 (47.6) | 85.2 (38.1) | 88.0 (49.3) | 74.4 (47.9) | 85.5 (43.8) | 72.7 (48.5) | 94.9 (45.9) | 85.1 (36.2) | 97.1 (47.6) |
| Median  (IQR) | 87.0  (51.5, 123.0) | 86.0  (58.8, 112.0) | 87.0  (50.5, 126.0) | 77.0  (29.2, 112.0) | 83.0  (58.0, 112.0) | 73.0  (23.5, 108.5) | 91.0  (64.0, 129.0) | 87.0  (60.5, 102.5) | 93.5  (65.8, 131.0) |
| Range | 1.0 - 216.0 | 14.0 - 158.0 | 1.0 - 216.0 | 1.0 - 173.0 | 14.0 - 156.0 | 1.0 - 173.0 | 1.0 - 216.0 | 17.0 - 158.0 | 1.0 - 216.0 |
| **Time since last vaccine dose, n (%)** |  |  |  |  |  |  |  |  |  |
| <2 months | 75 (28.1) | 12 (27.3) | 63 (28.3) | 38 (39.6) | 4 (30.8) | 34 (41.0) | 37 (21.6) | 8 (25.8) | 29 (20.7) |
| [2 - 4) months | 119 (44.6) | 24 (54.5) | 95 (42.6) | 39 (40.6) | 7 (53.8) | 32 (38.6) | 80 (46.8) | 17 (54.8) | 63 (45.0) |
| [4 - 6) months | 71 (26.6) | 8 (18.2) | 63 (28.3) | 19 (19.8) | 2 (15.4) | 17 (20.5) | 52 (30.4) | 6 (19.4) | 46 (32.9) |
| [6 - 8) months | 2 (0.7) | 0 (0.0) | 2 (0.9) | 0 (0.0) | 0 (0.0) | 0 (0.0) | 2 (1.2) | 0 (0.0) | 2 (1.4) |
| ≥8 months | 0 (0.0) | 0 (0.0) | 0 (0.0) | 0 (0.0) | 0 (0.0) | 0 (0.0) | 0 (0.0) | 0 (0.0) | 0 (0.0) |
| **SYMPTOMS** | | | | | | | | | |
| **SARI symptoms ^d,e^, n (%)** |  |  |  |  |  |  |  |  |  |
| Cough | 231 (66.0) | 32 (66.7) | 199 (65.9) | 131 (80.4) | 14 (93.3) | 117 (79.1) | 100 (53.5) | 18 (54.5) | 82 (53.2) |
| Fever | 172 (49.0) | 25 (53.2) | 147 (48.4) | 94 (58.0) | 8 (57.1) | 86 (58.1) | 78 (41.3) | 17 (51.5) | 61 (39.1) |
| Shortness of breath | 266 (73.7) | 34 (68.0) | 232 (74.6) | 127 (75.1) | 12 (75.0) | 115 (75.2) | 139 (72.4) | 22 (64.7) | 117 (74.1) |
| Anosmia, ageusia or dysgeusia | 16 (5.1) | 3 (7.0) | 13 (4.8) | 14 (9.9) | 3 (30.0) | 11 (8.4) | 2 (1.1) | 0 (0.0) | 2 (1.4) |
| **Number of SARI symptoms, n (%)** |  |  |  |  |  |  |  |  |  |
| 1 | 106 (34.2) | 18 (43.9) | 88 (32.7) | 37 (26.8) | 3 (30.0) | 34 (26.6) | 69 (40.1) | 15 (48.4) | 54 (38.3) |
| 2 | 140 (45.2) | 13 (31.7) | 127 (47.2) | 51 (37.0) | 2 (20.0) | 49 (38.3) | 89 (51.7) | 11 (35.5) | 78 (55.3) |
| 3+ | 64 (20.6) | 10 (24.4) | 54 (20.1) | 50 (36.2) | 5 (50.0) | 45 (35.2) | 14 (8.1) | 5 (16.1) | 9 (6.4) |
| **HOSPITAL OUTCOMES** | | | | | | | | | |
| **Length of stay (days)** |  |  |  |  |  |  |  |  |  |
| Mean (SD) | 11.4 (13.5) | 12.8 (21.6) | 11.2 (11.8) | 13.4 (16.9) | 22.6 (33.7) | 12.4 (13.8) | 9.5 (8.8) | 7.6 (7.4) | 9.9 (9.0) |
| Median  (IQR) | 7.0  (4.0, 14.0) | 6.0  (3.0, 12.2) | 7.0  (4.0, 14.0) | 8.0  (4.0, 15.0) | 8.5  (4.5, 22.2) | 7.5  (4.0, 15.0) | 7.0  (4.0, 13.0) | 5.0  (2.2, 10.5) | 7.0  (4.0, 13.0) |
| Range | 1.0 - 122.0 | 1.0 - 122.0 | 1.0 - 80.0 | 1.0 - 122.0 | 1.0 - 122.0 | 1.0 - 80.0 | 1.0 - 76.0 | 1.0 - 30.0 | 1.0 - 76.0 |
| **Severe outcomes, n (%)** |  |  |  |  |  |  |  |  |  |
| Hospitalization without ICU admission or in-hospital death | 268 (70.9) | 37 (71.2) | 231 (70.9) | 115 (61.8) | 12 (66.7) | 103 (61.3) | 153 (79.7) | 25 (73.5) | 128 (81.0) |
| ICU admission without in-hospital death | 54 (14.3) | 4 (7.7) | 50 (15.3) | 46 (24.7) | 3 (16.7) | 43 (25.6) | 8 (4.2) | 1 (2.9) | 7 (4.4) |
| ICU admission (with or without in-hospital death) | 66 (17.5) | 7 (13.5) | 59 (18.1) | 58 (31.2) | 6 (33.3) | 52 (31.0) | 8 (4.2) | 1 (2.9) | 7 (4.4) |
| In-hospital death | 56 (14.8) | 11 (21.2) | 45 (13.8) | 25 (13.4) | 3 (16.7) | 22 (13.1) | 31 (16.1) | 8 (23.5) | 23 (14.6) |
| **COMORBIDITIES** | | | | | | | | | |
| **Number of comorbidities other than IC, n (%)** |  |  |  |  |  |  |  |  |  |
| No comorbid conditions | 96 (25.4) | 5 (9.6) | 91 (27.9) | 74 (39.8) | 4 (22.2) | 70 (41.7) | 22 (11.5) | 1 (2.9) | 21 (13.3) |
| 1 comorbidity | 82 (21.7) | 17 (32.7) | 65 (19.9) | 37 (19.9) | 7 (38.9) | 30 (17.9) | 45 (23.4) | 10 (29.4) | 35 (22.2) |
| 2 comorbidities | 81 (21.4) | 9 (17.3) | 72 (22.1) | 43 (23.1) | 3 (16.7) | 40 (23.8) | 38 (19.8) | 6 (17.6) | 32 (20.3) |
| 3+ comorbidities | 119 (31.5) | 21 (40.4) | 98 (30.1) | 32 (17.2) | 4 (22.2) | 28 (16.7) | 87 (45.3) | 17 (50.0) | 70 (44.3) |
| **Number with specific comorbidities ^d^, n (%)** |  |  |  |  |  |  |  |  |  |
| Asthma | 28 (7.4) | 3 (5.8) | 25 (7.7) | 13 (7.0) | 2 (11.1) | 11 (6.5) | 15 (7.8) | 1 (2.9) | 14 (8.9) |
| Lung disease | 111 (29.4) | 14 (26.9) | 97 (29.8) | 33 (17.7) | 2 (11.1) | 31 (18.5) | 78 (40.6) | 12 (35.3) | 66 (41.8) |
| Cardiovascular disease | 114 (30.2) | 9 (17.3) | 105 (32.2) | 37 (19.9) | 1 (5.6) | 36 (21.4) | 77 (40.1) | 8 (23.5) | 69 (43.7) |
| Hypertension | 155 (41.0) | 18 (34.6) | 137 (42.0) | 64 (34.4) | 4 (22.2) | 60 (35.7) | 91 (47.4) | 14 (41.2) | 77 (48.7) |
| Chronic liver disease | 19 (5.0) | 4 (7.7) | 15 (4.6) | 9 (4.8) | 2 (11.1) | 7 (4.2) | 10 (5.2) | 2 (5.9) | 8 (5.1) |
| Chronic kidney disease | 72 (19.0) | 8 (15.4) | 64 (19.6) | 27 (14.5) | 3 (16.7) | 24 (14.3) | 45 (23.4) | 5 (14.7) | 40 (25.3) |
| Type 2 diabetes | 99 (26.2) | 13 (25.0) | 86 (26.4) | 43 (23.1) | 4 (22.2) | 39 (23.2) | 56 (29.2) | 9 (26.5) | 47 (29.7) |
| Cancer | 72 (19.0) | 36 (69.2) | 36 (11.0) | 17 (9.1) | 7 (38.9) | 10 (6.0) | 55 (28.6) | 29 (85.3) | 26 (16.5) |
| Alternative IC ^h^ | 88 (23.3) | 52 (100.0) | 36 (11.0) | 28 (15.1) | 18 (100.0) | 10 (6.0) | 60 (31.2) | 34 (100.0) | 26 (16.5) |
| **Q4 2021** |  |  |  |  |  |  |  |  |  |
| **Total** | **1,017** | **149** | **868** | **391** | **50** | **341** | **626** | **99** | **527** |
| **DEMOGRAPHICS** | | | | | | | | | |
| **Age (years)** |  |  |  |  |  |  |  |  |  |
| Mean (SD) | 66.9 (16.4) | 63.5 (14.4) | 67.4 (16.7) | 62.7 (15.3) | 60.8 (14.1) | 63.0 (15.5) | 69.5 (16.6) | 64.9 (14.4) | 70.3 (16.8) |
| Median  (IQR) | 68.0  (57.0, 79.0) | 66.0  (56.0, 73.0) | 69.0  (57.0, 80.0) | 65.0  (53.0, 73.5) | 61.5  (54.2, 72.0) | 65.0  (53.0, 74.0) | 72.0  (59.0, 83.0) | 67.0  (58.5, 74.5) | 73.0  (60.0, 84.0) |
| Range | 18.0 - 103.0 | 23.0 - 90.0 | 18.0 - 103.0 | 27.0 - 95.0 | 27.0 - 84.0 | 27.0 - 95.0 | 18.0 - 103.0 | 23.0 - 90.0 | 18.0 - 103.0 |
| **Sex, n (%)** |  |  |  |  |  |  |  |  |  |
| Male | 581 (57.1) | 86 (57.7) | 495 (57.0) | 229 (58.6) | 24 (48.0) | 205 (60.1) | 352 (56.2) | 62 (62.6) | 290 (55.0) |
| Female | 436 (42.9) | 63 (42.3) | 373 (43.0) | 162 (41.4) | 26 (52.0) | 136 (39.9) | 274 (43.8) | 37 (37.4) | 237 (45.0) |
| **Country, n (%)** |  |  |  |  |  |  |  |  |  |
| Belgium | 162 (15.9) | 34 (22.8) | 128 (14.7) | 121 (30.9) | 29 (58.0) | 92 (27.0) | 41 (6.5) | 5 (5.1) | 36 (6.8) |
| Italy | 87 (8.6) | 5 (3.4) | 82 (9.4) | 57 (14.6) | 2 (4.0) | 55 (16.1) | 30 (4.8) | 3 (3.0) | 27 (5.1) |
| Spain | 768 (75.5) | 110 (73.8) | 658 (75.8) | 213 (54.5) | 19 (38.0) | 194 (56.9) | 555 (88.7) | 91 (91.9) | 464 (88.0) |
| **Study site, n (%)** |  |  |  |  |  |  |  |  |  |
| CIRI-IT | 87 (8.6) | 5 (3.4) | 82 (9.4) | 57 (14.6) | 2 (4.0) | 55 (16.1) | 30 (4.8) | 3 (3.0) | 27 (5.1) |
| GTPUH | 550 (54.1) | 84 (56.4) | 466 (53.7) | 120 (30.7) | 11 (22.0) | 109 (32.0) | 430 (68.7) | 73 (73.7) | 357 (67.7) |
| CHU Saint-Pierre | 28 (2.8) | 0 (0.0) | 28 (3.2) | 12 (3.1) | 0 (0.0) | 12 (3.5) | 16 (2.6) | 0 (0.0) | 16 (3.0) |
| UZA | 134 (13.2) | 34 (22.8) | 100 (11.5) | 109 (27.9) | 29 (58.0) | 80 (23.5) | 25 (4.0) | 5 (5.1) | 20 (3.8) |
| HUVH | 218 (21.4) | 26 (17.4) | 192 (22.1) | 93 (23.8) | 8 (16.0) | 85 (24.9) | 125 (20.0) | 18 (18.2) | 107 (20.3) |
| **Smoking, n (%)** |  |  |  |  |  |  |  |  |  |
| Never smoker | 431 (42.4) | 54 (36.2) | 377 (43.4) | 151 (38.6) | 15 (30.0) | 136 (39.9) | 280 (44.7) | 39 (39.4) | 241 (45.7) |
| Former smoker | 245 (24.1) | 39 (26.2) | 206 (23.7) | 81 (20.7) | 7 (14.0) | 74 (21.7) | 164 (26.2) | 32 (32.3) | 132 (25.0) |
| Current smoker | 114 (11.2) | 15 (10.1) | 99 (11.4) | 23 (5.9) | 2 (4.0) | 21 (6.2) | 91 (14.5) | 13 (13.1) | 78 (14.8) |
| Missing | 227 (22.3) | 41 (27.5) | 186 (21.4) | 136 (34.8) | 26 (52.0) | 110 (32.3) | 91 (14.5) | 15 (15.2) | 76 (14.4) |
| **Long-term care facility residence, n (%)** |  |  |  |  |  |  |  |  |  |
| Yes | 52 (5.4) | 3 (2.1) | 49 (5.9) | 9 (2.4) | 1 (2.0) | 8 (2.4) | 43 (7.3) | 2 (2.2) | 41 (8.3) |
| **VACCINATION STATUS AT TIME OF HOSPITAL ADMISSION** | | | | | | | | | |
| Unvaccinated, n (%) | 175 (17.2) | 16 (10.7) | 159 (18.3) | 143 (36.6) | 11 (22.0) | 132 (38.7) | 32 (5.1) | 5 (5.1) | 27 (5.1) |
| Incomplete primary series, n (%) | 26 (2.6) | 3 (2.0) | 23 (2.6) | 6 (1.5) | 1 (2.0) | 5 (1.5) | 20 (3.2) | 2 (2.0) | 18 (3.4) |
| Primary series completed but no boosters, n (%) | 528 (51.9) | 65 (43.6) | 463 (53.3) | 177 (45.3) | 18 (36.0) | 159 (46.6) | 351 (56.1) | 47 (47.5) | 304 (57.7) |
| At least one booster dose, n (%) | 288 (28.3) | 65 (43.6) | 223 (25.7) | 65 (16.6) | 20 (40.0) | 45 (13.2) | 223 (35.6) | 45 (45.5) | 178 (33.8) |
| **Number of booster doses ^c^, n (%)** |  |  |  |  |  |  |  |  |  |
| 0 | 528 (64.7) | 65 (50.0) | 463 (67.5) | 177 (73.1) | 18 (47.4) | 159 (77.9) | 351 (61.1) | 47 (51.1) | 304 (63.1) |
| 1 | 288 (35.3) | 65 (50.0) | 223 (32.5) | 65 (26.9) | 20 (52.6) | 45 (22.1) | 223 (38.9) | 45 (48.9) | 178 (36.9) |
| 2 | 0 (0.0) | 0 (0.0) | 0 (0.0) | 0 (0.0) | 0 (0.0) | 0 (0.0) | 0 (0.0) | 0 (0.0) | 0 (0.0) |
| 3+ | 0 (0.0) | 0 (0.0) | 0 (0.0) | 0 (0.0) | 0 (0.0) | 0 (0.0) | 0 (0.0) | 0 (0.0) | 0 (0.0) |
| **Time since last vaccine dose (days)** |  |  |  |  |  |  |  |  |  |
| Mean (SD) | 125.6 (78.6) | 107.0 (76.8) | 129.1 (78.5) | 143.0 (75.5) | 119.9 (79.2) | 147.2 (74.2) | 118.3 (78.8) | 101.7 (75.6) | 121.5 (79.1) |
| Median  (IQR) | 138.5  (48.0, 186.0) | 92.0  (39.0, 168.0) | 145.0  (51.0, 188.0) | 161.0  (73.8, 196.0) | 80.0  (49.0, 196.0) | 164.0  (106.0, 196.0) | 123.5  (42.0, 183.0) | 92.5  (34.5, 162.2) | 128.0  (43.8, 186.0) |
| Range | 1.0 - 327.0 | 2.0 - 282.0 | 1.0 - 327.0 | 1.0 - 316.0 | 13.0 - 282.0 | 1.0 - 316.0 | 1.0 - 327.0 | 2.0 - 266.0 | 1.0 - 327.0 |
| **Time since last vaccine dose, n (%)** |  |  |  |  |  |  |  |  |  |
| <2 months | 242 (28.7) | 50 (37.6) | 192 (27.1) | 46 (18.5) | 11 (28.2) | 35 (16.7) | 196 (33.0) | 39 (41.5) | 157 (31.4) |
| [2 - 4) months | 128 (15.2) | 25 (18.8) | 103 (14.5) | 38 (15.3) | 11 (28.2) | 27 (12.9) | 90 (15.2) | 14 (14.9) | 76 (15.2) |
| [4 - 6) months | 229 (27.2) | 28 (21.1) | 201 (28.3) | 81 (32.7) | 6 (15.4) | 75 (35.9) | 148 (24.9) | 22 (23.4) | 126 (25.2) |
| [6 - 8) months | 195 (23.2) | 25 (18.8) | 170 (24.0) | 62 (25.0) | 9 (23.1) | 53 (25.4) | 133 (22.4) | 16 (17.0) | 117 (23.4) |
| ≥8 months | 48 (5.7) | 5 (3.8) | 43 (6.1) | 21 (8.5) | 2 (5.1) | 19 (9.1) | 27 (4.5) | 3 (3.2) | 24 (4.8) |
| **SYMPTOMS** | | | | | | | | | |
| **SARI symptoms ^d,e^, n (%)** |  |  |  |  |  |  |  |  |  |
| Cough | 539 (60.8) | 74 (53.6) | 465 (62.1) | 226 (80.1) | 33 (80.5) | 193 (80.1) | 313 (51.7) | 41 (42.3) | 272 (53.5) |
| Fever | 472 (53.7) | 91 (65.5) | 381 (51.5) | 189 (67.3) | 31 (75.6) | 158 (65.8) | 283 (47.3) | 60 (61.2) | 223 (44.6) |
| Shortness of breath | 638 (71.5) | 80 (57.6) | 558 (74.1) | 221 (77.8) | 26 (63.4) | 195 (80.2) | 417 (68.6) | 54 (55.1) | 363 (71.2) |
| Anosmia, ageusia or dysgeusia | 44 (5.5) | 8 (6.8) | 36 (5.3) | 40 (17.8) | 7 (29.2) | 33 (16.4) | 4 (0.7) | 1 (1.1) | 3 (0.6) |
| **Number of SARI symptoms, n (%)** |  |  |  |  |  |  |  |  |  |
| 1 | 317 (39.9) | 57 (49.1) | 260 (38.3) | 44 (19.7) | 5 (21.7) | 39 (19.5) | 273 (47.8) | 52 (55.9) | 221 (46.2) |
| 2 | 306 (38.5) | 37 (31.9) | 269 (39.7) | 74 (33.2) | 6 (26.1) | 68 (34.0) | 232 (40.6) | 31 (33.3) | 201 (42.1) |
| 3+ | 171 (21.5) | 22 (19.0) | 149 (22.0) | 105 (47.1) | 12 (52.2) | 93 (46.5) | 66 (11.6) | 10 (10.8) | 56 (11.7) |
| **HOSPITAL OUTCOMES** | | | | | | | | | |
| **Length of stay (days)** |  |  |  |  |  |  |  |  |  |
| Mean (SD) | 11.8 (18.4) | 10.3 (14.0) | 12.0 (19.0) | 15.4 (26.6) | 11.3 (18.3) | 16.0 (27.6) | 9.5 (9.7) | 9.8 (11.3) | 9.4 (9.4) |
| Median  (IQR) | 7.0  (4.0, 13.0) | 6.0  (4.0, 12.0) | 8.0  (4.0, 13.0) | 9.0  (5.0, 16.0) | 5.5  (4.0, 11.8) | 10.0  (5.0, 17.0) | 7.0  (4.0, 11.0) | 6.0  (4.0, 11.5) | 7.0  (4.0, 11.0) |
| Range | 1.0 - 403.0 | 1.0 - 104.0 | 1.0 - 403.0 | 1.0 - 403.0 | 1.0 - 104.0 | 1.0 - 403.0 | 1.0 - 96.0 | 1.0 - 70.0 | 1.0 - 96.0 |
| **Severe outcomes, n (%)** |  |  |  |  |  |  |  |  |  |
| Hospitalization without ICU admission or in-hospital death | 798 (78.5) | 127 (85.2) | 671 (77.3) | 258 (66.0) | 42 (84.0) | 216 (63.3) | 540 (86.3) | 85 (85.9) | 455 (86.3) |
| ICU admission without in-hospital death | 104 (10.2) | 4 (2.7) | 100 (11.5) | 80 (20.5) | 3 (6.0) | 77 (22.6) | 24 (3.8) | 1 (1.0) | 23 (4.4) |
| ICU admission (with or without in-hospital death) | 142 (14.0) | 11 (7.4) | 131 (15.1) | 112 (28.6) | 8 (16.0) | 104 (30.5) | 30 (4.8) | 3 (3.0) | 27 (5.1) |
| In-hospital death | 115 (11.3) | 18 (12.1) | 97 (11.2) | 53 (13.6) | 5 (10.0) | 48 (14.1) | 62 (9.9) | 13 (13.1) | 49 (9.3) |
| **COMORBIDITIES** | | | | | | | | | |
| **Number of comorbidities other than IC, n (%)** |  |  |  |  |  |  |  |  |  |
| No comorbid conditions | 202 (19.9) | 7 (4.7) | 195 (22.5) | 119 (30.4) | 4 (8.0) | 115 (33.7) | 83 (13.3) | 3 (3.0) | 80 (15.2) |
| 1 comorbidity | 228 (22.4) | 48 (32.2) | 180 (20.7) | 91 (23.3) | 18 (36.0) | 73 (21.4) | 137 (21.9) | 30 (30.3) | 107 (20.3) |
| 2 comorbidities | 214 (21.0) | 36 (24.2) | 178 (20.5) | 74 (18.9) | 11 (22.0) | 63 (18.5) | 140 (22.4) | 25 (25.3) | 115 (21.8) |
| 3+ comorbidities | 373 (36.7) | 58 (38.9) | 315 (36.3) | 107 (27.4) | 17 (34.0) | 90 (26.4) | 266 (42.5) | 41 (41.4) | 225 (42.7) |
| **Number with specific comorbidities ^d^, n (%)** |  |  |  |  |  |  |  |  |  |
| Asthma | 76 (7.5) | 12 (8.1) | 64 (7.4) | 24 (6.1) | 6 (12.0) | 18 (5.3) | 52 (8.3) | 6 (6.1) | 46 (8.7) |
| Lung disease | 303 (29.8) | 49 (32.9) | 254 (29.3) | 75 (19.2) | 13 (26.0) | 62 (18.2) | 228 (36.4) | 36 (36.4) | 192 (36.4) |
| Cardiovascular disease | 394 (38.7) | 42 (28.2) | 352 (40.6) | 121 (30.9) | 12 (24.0) | 109 (32.0) | 273 (43.6) | 30 (30.3) | 243 (46.1) |
| Hypertension | 519 (51.0) | 70 (47.0) | 449 (51.7) | 166 (42.5) | 21 (42.0) | 145 (42.5) | 353 (56.4) | 49 (49.5) | 304 (57.7) |
| Chronic liver disease | 72 (7.1) | 14 (9.4) | 58 (6.7) | 20 (5.1) | 6 (12.0) | 14 (4.1) | 52 (8.3) | 8 (8.1) | 44 (8.3) |
| Chronic kidney disease | 191 (18.8) | 35 (23.5) | 156 (18.0) | 57 (14.6) | 13 (26.0) | 44 (12.9) | 134 (21.4) | 22 (22.2) | 112 (21.3) |
| Type 2 diabetes | 259 (25.5) | 28 (18.8) | 231 (26.6) | 97 (24.8) | 9 (18.0) | 88 (25.8) | 162 (25.9) | 19 (19.2) | 143 (27.1) |
| Cancer | 210 (20.6) | 101 (67.8) | 109 (12.6) | 66 (16.9) | 31 (62.0) | 35 (10.3) | 144 (23.0) | 70 (70.7) | 74 (14.0) |
| Alternative IC ^h^ | 258 (25.4) | 149 (100.0) | 109 (12.6) | 85 (21.7) | 50 (100.0) | 35 (10.3) | 173 (27.6) | 99 (100.0) | 74 (14.0) |
| **Q1 2022** |  |  |  |  |  |  |  |  |  |
| **Total** | **1,112** | **111** | **1,001** | **523** | **55** | **468** | **589** | **56** | **533** |
| **DEMOGRAPHICS** | | | | | | | | | |
| **Age (years)** |  |  |  |  |  |  |  |  |  |
| Mean (SD) | 68.5 (17.2) | 62.9 (15.2) | 69.1 (17.3) | 68.7 (16.2) | 62.0 (15.5) | 69.5 (16.2) | 68.3 (17.9) | 63.7 (14.9) | 68.8 (18.2) |
| Median  (IQR) | 72.0  (58.0, 82.0) | 66.0  (52.5, 74.0) | 73.0  (59.0, 83.0) | 72.0  (59.0, 81.0) | 65.0  (53.5, 73.5) | 73.0  (60.0, 82.0) | 72.0  (58.0, 83.0) | 69.0  (52.8, 74.2) | 73.0  (58.0, 83.0) |
| Range | 18.0 - 101.0 | 21.0 - 90.0 | 18.0 - 101.0 | 20.0 - 99.0 | 21.0 - 90.0 | 20.0 - 99.0 | 18.0 - 101.0 | 28.0 - 90.0 | 18.0 - 101.0 |
| **Sex, n (%)** |  |  |  |  |  |  |  |  |  |
| Male | 630 (56.7) | 65 (58.6) | 565 (56.4) | 300 (57.4) | 28 (50.9) | 272 (58.1) | 330 (56.0) | 37 (66.1) | 293 (55.0) |
| Female | 482 (43.3) | 46 (41.4) | 436 (43.6) | 223 (42.6) | 27 (49.1) | 196 (41.9) | 259 (44.0) | 19 (33.9) | 240 (45.0) |
| **Country, n (%)** |  |  |  |  |  |  |  |  |  |
| Belgium | 97 (8.7) | 16 (14.4) | 81 (8.1) | 57 (10.9) | 12 (21.8) | 45 (9.6) | 40 (6.8) | 4 (7.1) | 36 (6.8) |
| Italy | 503 (45.2) | 22 (19.8) | 481 (48.1) | 212 (40.5) | 11 (20.0) | 201 (42.9) | 291 (49.4) | 11 (19.6) | 280 (52.5) |
| Spain | 512 (46.0) | 73 (65.8) | 439 (43.9) | 254 (48.6) | 32 (58.2) | 222 (47.4) | 258 (43.8) | 41 (73.2) | 217 (40.7) |
| **Study site, n (%)** |  |  |  |  |  |  |  |  |  |
| CIRI-IT | 503 (45.2) | 22 (19.8) | 481 (48.1) | 212 (40.5) | 11 (20.0) | 201 (42.9) | 291 (49.4) | 11 (19.6) | 280 (52.5) |
| GTPUH | 315 (28.3) | 42 (37.8) | 273 (27.3) | 105 (20.1) | 8 (14.5) | 97 (20.7) | 210 (35.7) | 34 (60.7) | 176 (33.0) |
| CHU Saint-Pierre | 25 (2.2) | 0 (0.0) | 25 (2.5) | 9 (1.7) | 0 (0.0) | 9 (1.9) | 16 (2.7) | 0 (0.0) | 16 (3.0) |
| UZA | 72 (6.5) | 16 (14.4) | 56 (5.6) | 48 (9.2) | 12 (21.8) | 36 (7.7) | 24 (4.1) | 4 (7.1) | 20 (3.8) |
| HUVH | 197 (17.7) | 31 (27.9) | 166 (16.6) | 149 (28.5) | 24 (43.6) | 125 (26.7) | 48 (8.1) | 7 (12.5) | 41 (7.7) |
| **Smoking, n (%)** |  |  |  |  |  |  |  |  |  |
| Never smoker | 336 (30.2) | 46 (41.4) | 290 (29.0) | 186 (35.6) | 29 (52.7) | 157 (33.5) | 150 (25.5) | 17 (30.4) | 133 (25.0) |
| Former smoker | 232 (20.9) | 31 (27.9) | 201 (20.1) | 114 (21.8) | 8 (14.5) | 106 (22.6) | 118 (20.0) | 23 (41.1) | 95 (17.8) |
| Current smoker | 101 (9.1) | 12 (10.8) | 89 (8.9) | 45 (8.6) | 5 (9.1) | 40 (8.5) | 56 (9.5) | 7 (12.5) | 49 (9.2) |
| Missing | 443 (39.8) | 22 (19.8) | 421 (42.1) | 178 (34.0) | 13 (23.6) | 165 (35.3) | 265 (45.0) | 9 (16.1) | 256 (48.0) |
| **Long-term care facility residence, n (%)** |  |  |  |  |  |  |  |  |  |
| Yes | 33 (3.0) | 1 (0.9) | 32 (3.2) | 13 (2.5) | 0 (0.0) | 13 (2.8) | 20 (3.4) | 1 (1.9) | 19 (3.6) |
| **VACCINATION STATUS AT TIME OF HOSPITAL ADMISSION** | | | | | | | | | |
| Unvaccinated, n (%) | 142 (12.8) | 5 (4.5) | 137 (13.7) | 104 (19.9) | 2 (3.6) | 102 (21.8) | 38 (6.5) | 3 (5.4) | 35 (6.6) |
| Incomplete primary series, n (%) | 30 (2.7) | 1 (0.9) | 29 (2.9) | 15 (2.9) | 1 (1.8) | 14 (3.0) | 15 (2.5) | 0 (0.0) | 15 (2.8) |
| Primary series completed but no boosters, n (%) | 221 (19.9) | 21 (18.9) | 200 (20.0) | 122 (23.3) | 10 (18.2) | 112 (23.9) | 99 (16.8) | 11 (19.6) | 88 (16.5) |
| At least one booster dose, n (%) | 719 (64.7) | 84 (75.7) | 635 (63.4) | 282 (53.9) | 42 (76.4) | 240 (51.3) | 437 (74.2) | 42 (75.0) | 395 (74.1) |
| **Number of booster doses ^c^, n (%)** |  |  |  |  |  |  |  |  |  |
| 0 | 221 (23.5) | 21 (20.0) | 200 (24.0) | 122 (30.2) | 10 (19.2) | 112 (31.8) | 99 (18.5) | 11 (20.8) | 88 (18.2) |
| 1 | 715 (76.1) | 82 (78.1) | 633 (75.8) | 280 (69.3) | 41 (78.8) | 239 (67.9) | 435 (81.2) | 41 (77.4) | 394 (81.6) |
| 2 | 4 (0.4) | 2 (1.9) | 2 (0.2) | 2 (0.5) | 1 (1.9) | 1 (0.3) | 2 (0.4) | 1 (1.9) | 1 (0.2) |
| 3+ | 0 (0.0) | 0 (0.0) | 0 (0.0) | 0 (0.0) | 0 (0.0) | 0 (0.0) | 0 (0.0) | 0 (0.0) | 0 (0.0) |
| **Time since last vaccine dose (days)** |  |  |  |  |  |  |  |  |  |
| Mean (SD) | 113.9 (78.0) | 121.1 (77.2) | 113.0 (78.1) | 129.1 (84.3) | 132.1 (80.9) | 128.6 (84.9) | 102.4 (70.7) | 110.1 (72.3) | 101.6 (70.6) |
| Median  (IQR) | 92.0  (60.0, 143.0) | 106.0  (67.0, 146.8) | 91.5  (60.0, 141.0) | 106.0  (67.0, 177.0) | 130.0  (69.0, 148.0) | 102.5  (67.0, 189.0) | 83.0  (56.0, 124.5) | 83.0  (63.0, 132.0) | 83.5  (55.0, 123.0) |
| Range | 1.0 - 374.0 | 1.0 - 329.0 | 2.0 - 374.0 | 1.0 - 367.0 | 1.0 - 329.0 | 4.0 - 367.0 | 2.0 - 374.0 | 2.0 - 326.0 | 2.0 - 374.0 |
| **Time since last vaccine dose, n (%)** |  |  |  |  |  |  |  |  |  |
| <2 months | 236 (24.3) | 21 (19.8) | 215 (24.9) | 82 (19.6) | 9 (17.0) | 73 (19.9) | 154 (27.9) | 12 (22.6) | 142 (28.5) |
| [2 - 4) months | 414 (42.7) | 42 (39.6) | 372 (43.1) | 162 (38.7) | 17 (32.1) | 145 (39.6) | 252 (45.7) | 25 (47.2) | 227 (45.6) |
| [4 - 6) months | 148 (15.3) | 26 (24.5) | 122 (14.1) | 74 (17.7) | 19 (35.8) | 55 (15.0) | 74 (13.4) | 7 (13.2) | 67 (13.5) |
| [6 - 8) months | 72 (7.4) | 5 (4.7) | 67 (7.8) | 40 (9.5) | 0 (0.0) | 40 (10.9) | 32 (5.8) | 5 (9.4) | 27 (5.4) |
| ≥8 months | 100 (10.3) | 12 (11.3) | 88 (10.2) | 61 (14.6) | 8 (15.1) | 53 (14.5) | 39 (7.1) | 4 (7.5) | 35 (7.0) |
| **SYMPTOMS** | | | | | | | | | |
| **SARI symptoms ^d,e^, n (%)** |  |  |  |  |  |  |  |  |  |
| Cough | 672 (66.8) | 51 (52.6) | 621 (68.3) | 298 (68.2) | 28 (62.2) | 270 (68.9) | 374 (65.7) | 23 (44.2) | 351 (67.9) |
| Fever | 691 (68.6) | 64 (66.0) | 627 (68.8) | 308 (70.3) | 33 (73.3) | 275 (70.0) | 383 (67.2) | 31 (59.6) | 352 (68.0) |
| Shortness of breath | 722 (71.4) | 65 (67.0) | 657 (71.9) | 333 (75.2) | 31 (67.4) | 302 (76.1) | 389 (68.5) | 34 (66.7) | 355 (68.7) |
| Anosmia, ageusia or dysgeusia | 49 (5.1) | 3 (3.5) | 46 (5.3) | 40 (9.7) | 2 (5.3) | 38 (10.1) | 9 (1.6) | 1 (2.1) | 8 (1.6) |
| **Number of SARI symptoms, n (%)** |  |  |  |  |  |  |  |  |  |
| 1 | 247 (25.8) | 35 (41.2) | 212 (24.3) | 91 (22.1) | 11 (28.9) | 80 (21.4) | 156 (28.6) | 24 (51.1) | 132 (26.5) |
| 2 | 373 (39.0) | 28 (32.9) | 345 (39.6) | 151 (36.7) | 15 (39.5) | 136 (36.4) | 222 (40.7) | 13 (27.7) | 209 (42.0) |
| 3+ | 337 (35.2) | 22 (25.9) | 315 (36.1) | 170 (41.3) | 12 (31.6) | 158 (42.2) | 167 (30.6) | 10 (21.3) | 157 (31.5) |
| **HOSPITAL OUTCOMES** | | | | | | | | | |
| **Length of stay (days)** |  |  |  |  |  |  |  |  |  |
| Mean (SD) | 9.4 (10.7) | 12.3 (19.2) | 9.1 (9.2) | 11.4 (13.7) | 17.4 (25.5) | 10.7 (11.5) | 7.6 (6.4) | 7.2 (6.9) | 7.7 (6.3) |
| Median  (IQR) | 6.0  (4.0, 12.0) | 6.0  (4.0, 12.5) | 6.0  (4.0, 12.0) | 7.0  (4.0, 14.0) | 9.0  (4.0, 16.5) | 7.0  (4.0, 14.0) | 6.0  (3.0, 10.0) | 5.0  (3.0, 8.0) | 6.0  (3.0, 10.0) |
| Range | 1.0 - 141.0 | 1.0 - 141.0 | 1.0 - 105.0 | 1.0 - 141.0 | 1.0 - 141.0 | 1.0 - 105.0 | 1.0 - 46.0 | 1.0 - 44.0 | 1.0 - 46.0 |
| **Severe outcomes, n (%)** |  |  |  |  |  |  |  |  |  |
| Hospitalization without ICU admission or in-hospital death | 1,006 (90.5) | 96 (86.5) | 910 (90.9) | 438 (83.7) | 44 (80.0) | 394 (84.2) | 568 (96.4) | 52 (92.9) | 516 (96.8) |
| ICU admission without in-hospital death | 46 (4.1) | 5 (4.5) | 41 (4.1) | 37 (7.1) | 4 (7.3) | 33 (7.1) | 9 (1.5) | 1 (1.8) | 8 (1.5) |
| ICU admission (with or without in-hospital death) | 64 (5.8) | 10 (9.0) | 54 (5.4) | 48 (9.2) | 9 (16.4) | 45 (9.6) | 10 (1.7) | 1 (1.8) | 9 (1.7) |
| In-hospital death | 60 (5.4) | 10 (9.0) | 50 (5.0) | 48 (9.2) | 7 (12.7) | 41 (8.8) | 12 (2.0) | 3 (5.4) | 9 (1.7) |
| **COMORBIDITIES** | | | | | | | | | |
| **Number of comorbidities other than IC, n (%)** |  |  |  |  |  |  |  |  |  |
| No comorbid conditions | 214 (19.2) | 10 (9.0) | 204 (20.4) | 95 (18.2) | 5 (9.1) | 90 (19.2) | 119 (20.2) | 5 (8.9) | 114 (21.4) |
| 1 comorbidity | 272 (24.5) | 25 (22.5) | 247 (24.7) | 126 (24.1) | 14 (25.5) | 112 (23.9) | 146 (24.8) | 11 (19.6) | 135 (25.3) |
| 2 comorbidities | 247 (22.2) | 26 (23.4) | 221 (22.1) | 121 (23.1) | 14 (25.5) | 107 (22.9) | 126 (21.4) | 12 (21.4) | 114 (21.4) |
| 3+ comorbidities | 379 (34.1) | 50 (45.0) | 329 (32.9) | 181 (34.6) | 22 (40.0) | 159 (34.0) | 198 (33.6) | 28 (50.0) | 170 (31.9) |
| **Number with specific comorbidities ^d^, n (%)** |  |  |  |  |  |  |  |  |  |
| Asthma | 66 (5.9) | 6 (5.4) | 60 (6.0) | 34 (6.5) | 3 (5.5) | 31 (6.6) | 32 (5.4) | 3 (5.4) | 29 (5.4) |
| Lung disease | 285 (25.6) | 41 (36.9) | 244 (24.4) | 114 (21.8) | 18 (32.7) | 96 (20.5) | 171 (29.0) | 23 (41.1) | 148 (27.8) |
| Cardiovascular disease | 432 (38.8) | 45 (40.5) | 387 (38.7) | 218 (41.7) | 24 (43.6) | 194 (41.5) | 214 (36.3) | 21 (37.5) | 193 (36.2) |
| Hypertension | 549 (49.4) | 49 (44.1) | 500 (50.0) | 267 (51.1) | 25 (45.5) | 242 (51.7) | 282 (47.9) | 24 (42.9) | 258 (48.4) |
| Chronic liver disease | 71 (6.4) | 15 (13.5) | 56 (5.6) | 34 (6.5) | 7 (12.7) | 27 (5.8) | 37 (6.3) | 8 (14.3) | 29 (5.4) |
| Chronic kidney disease | 174 (15.6) | 30 (27.0) | 144 (14.4) | 88 (16.8) | 16 (29.1) | 72 (15.4) | 86 (14.6) | 14 (25.0) | 72 (13.5) |
| Type 2 diabetes | 318 (28.6) | 31 (27.9) | 287 (28.7) | 154 (29.4) | 12 (21.8) | 142 (30.3) | 164 (27.8) | 19 (33.9) | 145 (27.2) |
| Cancer | 243 (21.9) | 53 (47.7) | 190 (19.0) | 112 (21.4) | 18 (32.7) | 94 (20.1) | 131 (22.2) | 35 (62.5) | 96 (18.0) |
| Alternative IC ^h^ | 301 (27.1) | 111 (100.0) | 190 (19.0) | 149 (28.5) | 55 (100.0) | 94 (20.1) | 152 (25.8) | 56 (100.0) | 96 (18.0) |
| **Q2 2022** |  |  |  |  |  |  |  |  |  |
| **Total** | **538** | **83** | **455** | **293** | **53** | **240** | **245** | **30** | **215** |
| **DEMOGRAPHICS** | | | | | | | | | |
| **Age (years)** |  |  |  |  |  |  |  |  |  |
| Mean (SD) | 70.1 (16.0) | 64.4 (16.1) | 71.1 (15.8) | 70.2 (15.8) | 64.8 (15.7) | 71.4 (15.6) | 69.9 (16.3) | 63.6 (17.1) | 70.8 (16.1) |
| Median  (IQR) | 74.0  (62.0, 82.0) | 67.0  (55.5, 75.5) | 75.0  (64.0, 82.5) | 74.0  (62.0, 82.0) | 67.0  (58.0, 76.0) | 75.0  (64.0, 83.0) | 74.0  (61.0, 82.0) | 65.0  (51.5, 75.0) | 74.0  (64.0, 82.0) |
| Range | 19.0 - 102.0 | 21.0 - 93.0 | 19.0 - 102.0 | 22.0 - 102.0 | 28.0 - 93.0 | 22.0 - 102.0 | 19.0 - 95.0 | 21.0 - 92.0 | 19.0 - 95.0 |
| **Sex, n (%)** |  |  |  |  |  |  |  |  |  |
| Male | 315 (58.6) | 43 (51.8) | 272 (59.8) | 174 (59.4) | 27 (50.9) | 147 (61.2) | 141 (57.6) | 16 (53.3) | 125 (58.1) |
| Female | 223 (41.4) | 40 (48.2) | 183 (40.2) | 119 (40.6) | 26 (49.1) | 93 (38.8) | 104 (42.4) | 14 (46.7) | 90 (41.9) |
| **Country, n (%)** |  |  |  |  |  |  |  |  |  |
| Belgium | 20 (3.7) | 4 (4.8) | 16 (3.5) | 13 (4.4) | 3 (5.7) | 10 (4.2) | 7 (2.9) | 1 (3.3) | 6 (2.8) |
| Italy | 212 (39.4) | 15 (18.1) | 197 (43.3) | 80 (27.3) | 9 (17.0) | 71 (29.6) | 132 (53.9) | 6 (20.0) | 126 (58.6) |
| Spain | 306 (56.9) | 64 (77.1) | 242 (53.2) | 200 (68.3) | 41 (77.4) | 159 (66.2) | 106 (43.3) | 23 (76.7) | 83 (38.6) |
| **Study site, n (%)** |  |  |  |  |  |  |  |  |  |
| CIRI-IT | 212 (39.4) | 15 (18.1) | 197 (43.3) | 80 (27.3) | 9 (17.0) | 71 (29.6) | 132 (53.9) | 6 (20.0) | 126 (58.6) |
| GTPUH | 103 (19.1) | 26 (31.3) | 77 (16.9) | 31 (10.6) | 8 (15.1) | 23 (9.6) | 72 (29.4) | 18 (60.0) | 54 (25.1) |
| CHU Saint-Pierre | 2 (0.4) | 0 (0.0) | 2 (0.4) | 1 (0.3) | 0 (0.0) | 1 (0.4) | 1 (0.4) | 0 (0.0) | 1 (0.5) |
| UZA | 18 (3.3) | 4 (4.8) | 14 (3.1) | 12 (4.1) | 3 (5.7) | 9 (3.8) | 6 (2.4) | 1 (3.3) | 5 (2.3) |
| HUVH | 203 (37.7) | 38 (45.8) | 165 (36.3) | 169 (57.7) | 33 (62.3) | 136 (56.7) | 34 (13.9) | 5 (16.7) | 29 (13.5) |
| **Smoking, n (%)** |  |  |  |  |  |  |  |  |  |
| Never smoker | 181 (33.6) | 36 (43.4) | 145 (31.9) | 120 (41.0) | 24 (45.3) | 96 (40.0) | 61 (24.9) | 12 (40.0) | 49 (22.8) |
| Former smoker | 164 (30.5) | 27 (32.5) | 137 (30.1) | 103 (35.2) | 18 (34.0) | 85 (35.4) | 61 (24.9) | 9 (30.0) | 52 (24.2) |
| Current smoker | 72 (13.4) | 11 (13.3) | 61 (13.4) | 39 (13.3) | 5 (9.4) | 34 (14.2) | 33 (13.5) | 6 (20.0) | 27 (12.6) |
| Missing | 121 (22.5) | 9 (10.8) | 112 (24.6) | 31 (10.6) | 6 (11.3) | 25 (10.4) | 90 (36.7) | 3 (10.0) | 87 (40.5) |
| **Long-term care facility residence, n (%)** |  |  |  |  |  |  |  |  |  |
| Yes | 24 (4.6) | 4 (5.2) | 20 (4.5) | 14 (5.0) | 2 (4.3) | 12 (5.1) | 10 (4.1) | 2 (6.7) | 8 (3.8) |
| **VACCINATION STATUS AT TIME OF HOSPITAL ADMISSION** | | | | | | | | | |
| Unvaccinated, n (%) | 33 (6.1) | 4 (4.8) | 29 (6.4) | 22 (7.5) | 3 (5.7) | 19 (7.9) | 11 (4.5) | 1 (3.3) | 10 (4.7) |
| Incomplete primary series, n (%) | 7 (1.3) | 1 (1.2) | 6 (1.3) | 4 (1.4) | 1 (1.9) | 3 (1.2) | 3 (1.2) | 0 (0.0) | 3 (1.4) |
| Primary series completed but no boosters, n (%) | 57 (10.6) | 11 (13.3) | 46 (10.1) | 24 (8.2) | 5 (9.4) | 19 (7.9) | 33 (13.5) | 6 (20.0) | 27 (12.6) |
| At least one booster dose, n (%) | 441 (82.0) | 67 (80.7) | 374 (82.2) | 243 (82.9) | 44 (83.0) | 199 (82.9) | 198 (80.8) | 23 (76.7) | 175 (81.4) |
| **Number of booster doses ^c^, n (%)** |  |  |  |  |  |  |  |  |  |
| 0 | 57 (11.4) | 11 (14.1) | 46 (11.0) | 24 (9.0) | 5 (10.2) | 19 (8.7) | 33 (14.3) | 6 (20.7) | 27 (13.4) |
| 1 | 414 (83.1) | 55 (70.5) | 359 (85.5) | 223 (83.5) | 34 (69.4) | 189 (86.7) | 191 (82.7) | 21 (72.4) | 170 (84.2) |
| 2 | 27 (5.4) | 12 (15.4) | 15 (3.6) | 20 (7.5) | 10 (20.4) | 10 (4.6) | 7 (3.0) | 2 (6.9) | 5 (2.5) |
| 3+ | 0 (0.0) | 0 (0.0) | 0 (0.0) | 0 (0.0) | 0 (0.0) | 0 (0.0) | 0 (0.0) | 0 (0.0) | 0 (0.0) |
| **Time since last vaccine dose (days)** |  |  |  |  |  |  |  |  |  |
| Mean (SD) | 175.0 (71.3) | 177.7 (81.1) | 174.5 (69.4) | 184.9 (71.9) | 174.7 (85.4) | 187.2 (68.4) | 163.6 (69.0) | 182.8 (74.3) | 160.9 (68.0) |
| Median  (IQR) | 173.0  (132.0, 210.0) | 178.0  (127.0, 224.5) | 172.0  (132.0, 206.8) | 187.0  (146.5, 219.0) | 177.0  (110.5, 220.2) | 187.0  (149.0, 219.0) | 159.0  (122.2, 191.8) | 178.0  (142.0, 225.0) | 156.0  (120.0, 190.0) |
| Range | 1.0 - 440.0 | 1.0 - 363.0 | 1.0 - 440.0 | 1.0 - 440.0 | 1.0 - 363.0 | 13.0 - 440.0 | 1.0 - 428.0 | 23.0 - 338.0 | 1.0 - 428.0 |
| **Time since last vaccine dose, n (%)** |  |  |  |  |  |  |  |  |  |
| <2 months | 22 (4.4) | 8 (10.1) | 14 (3.3) | 13 (4.8) | 6 (12.0) | 7 (3.2) | 9 (3.8) | 2 (6.9) | 7 (3.4) |
| [2 - 4) months | 78 (15.4) | 11 (13.9) | 67 (15.7) | 31 (11.4) | 8 (16.0) | 23 (10.4) | 47 (20.1) | 3 (10.3) | 44 (21.5) |
| [4 - 6) months | 181 (35.8) | 22 (27.8) | 159 (37.3) | 79 (29.2) | 11 (22.0) | 68 (30.8) | 102 (43.6) | 11 (37.9) | 91 (44.4) |
| [6 - 8) months | 167 (33.1) | 23 (29.1) | 144 (33.8) | 111 (41.0) | 15 (30.0) | 96 (43.4) | 56 (23.9) | 8 (27.6) | 48 (23.4) |
| ≥8 months | 57 (11.3) | 15 (19.0) | 42 (9.9) | 37 (13.7) | 10 (20.0) | 27 (12.2) | 20 (8.5) | 5 (17.2) | 15 (7.3) |
| **SYMPTOMS** | | | | | | | | | |
| **SARI symptoms ^d,e^, n (%)** |  |  |  |  |  |  |  |  |  |
| Cough | 391 (72.8) | 54 (65.1) | 337 (74.2) | 201 (68.8) | 37 (69.8) | 164 (68.6) | 190 (77.6) | 17 (56.7) | 173 (80.5) |
| Fever | 353 (65.9) | 45 (54.2) | 308 (68.0) | 178 (61.2) | 29 (54.7) | 149 (62.6) | 175 (71.4) | 16 (53.3) | 159 (74.0) |
| Shortness of breath | 366 (68.3) | 50 (61.0) | 316 (69.6) | 199 (68.2) | 29 (54.7) | 170 (71.1) | 167 (68.4) | 21 (72.4) | 146 (67.9) |
| Anosmia, ageusia or dysgeusia | 17 (3.3) | 1 (1.3) | 16 (3.7) | 12 (4.3) | 1 (2.0) | 11 (4.8) | 5 (2.1) | 0 (0.0) | 5 (2.4) |
| **Number of SARI symptoms, n (%)** |  |  |  |  |  |  |  |  |  |
| 1 | 132 (25.6) | 31 (39.7) | 101 (23.1) | 87 (31.5) | 22 (44.9) | 65 (28.6) | 45 (18.8) | 9 (31.0) | 36 (17.1) |
| 2 | 206 (40.0) | 31 (39.7) | 175 (40.0) | 98 (35.5) | 15 (30.6) | 83 (36.6) | 108 (45.2) | 16 (55.2) | 92 (43.8) |
| 3+ | 177 (34.4) | 16 (20.5) | 161 (36.8) | 91 (33.0) | 12 (24.5) | 79 (34.8) | 86 (36.0) | 4 (13.8) | 82 (39.0) |
| **HOSPITAL OUTCOMES** | | | | | | | | | |
| **Length of stay (days)** |  |  |  |  |  |  |  |  |  |
| Mean (SD) | 10.3 (14.1) | 12.1 (17.8) | 10.0 (13.3) | 11.2 (15.7) | 14.4 (21.4) | 10.6 (14.1) | 9.2 (11.9) | 8.2 (7.1) | 9.3 (12.4) |
| Median  (IQR) | 6.0  (3.0, 11.0) | 6.0  (4.0, 10.5) | 6.0  (3.0, 11.0) | 6.0  (3.0, 12.0) | 6.0  (3.0, 11.0) | 6.0  (3.0, 12.0) | 6.0  (3.0, 11.0) | 6.0  (4.0, 9.8) | 6.0  (2.0, 11.0) |
| Range | 1.0 - 146.0 | 1.0 - 95.0 | 1.0 - 146.0 | 1.0 - 146.0 | 1.0 - 95.0 | 1.0 - 146.0 | 1.0 - 94.0 | 1.0 - 32.0 | 1.0 - 94.0 |
| **Severe outcomes, n (%)** |  |  |  |  |  |  |  |  |  |
| Hospitalization without ICU admission or in-hospital death | 492 (91.6) | 73 (88.0) | 419 (92.3) | 256 (87.7) | 45 (84.9) | 211 (88.3) | 236 (96.3) | 28 (93.3) | 208 (96.7) |
| ICU admission without in-hospital death | 16 (3.0) | 5 (6.0) | 11 (2.4) | 12 (4.1) | 3 (5.7) | 9 (3.8) | 4 (1.6) | 2 (6.7) | 2 (0.9) |
| ICU admission (with or without in-hospital death) | 22 (4.1) | 7 (8.4) | 15 (3.3) | 17 (5.8) | 5 (9.4) | 12 (5.0) | 5 (2.0) | 2 (6.7) | 3 (1.4) |
| In-hospital death | 29 (5.4) | 5 (6.0) | 24 (5.3) | 24 (8.2) | 5 (9.4) | 19 (7.9) | 5 (2.0) | 0 (0.0) | 5 (2.3) |
| **COMORBIDITIES** | | | | | | | | | |
| **Number of comorbidities other than IC, n (%)** |  |  |  |  |  |  |  |  |  |
| No comorbid conditions | 87 (16.2) | 9 (10.8) | 78 (17.1) | 35 (11.9) | 6 (11.3) | 29 (12.1) | 52 (21.2) | 3 (10.0) | 49 (22.8) |
| 1 comorbidity | 110 (20.4) | 17 (20.5) | 93 (20.4) | 63 (21.5) | 10 (18.9) | 53 (22.1) | 47 (19.2) | 7 (23.3) | 40 (18.6) |
| 2 comorbidities | 131 (24.3) | 21 (25.3) | 110 (24.2) | 78 (26.6) | 14 (26.4) | 64 (26.7) | 53 (21.6) | 7 (23.3) | 46 (21.4) |
| 3+ comorbidities | 210 (39.0) | 36 (43.4) | 174 (38.2) | 117 (39.9) | 23 (43.4) | 94 (39.2) | 93 (38.0) | 13 (43.3) | 80 (37.2) |
| **Number with specific comorbidities ^d^, n (%)** |  |  |  |  |  |  |  |  |  |
| Asthma | 28 (5.2) | 5 (6.0) | 23 (5.1) | 17 (5.8) | 3 (5.7) | 14 (5.8) | 11 (4.5) | 2 (6.7) | 9 (4.2) |
| Lung disease | 195 (36.2) | 27 (32.5) | 168 (36.9) | 97 (33.1) | 19 (35.8) | 78 (32.5) | 98 (40.0) | 8 (26.7) | 90 (41.9) |
| Cardiovascular disease | 235 (43.7) | 27 (32.5) | 208 (45.7) | 133 (45.4) | 17 (32.1) | 116 (48.3) | 102 (41.6) | 10 (33.3) | 92 (42.8) |
| Hypertension | 286 (53.2) | 39 (47.0) | 247 (54.3) | 170 (58.0) | 30 (56.6) | 140 (58.3) | 116 (47.3) | 9 (30.0) | 107 (49.8) |
| Chronic liver disease | 30 (5.6) | 8 (9.6) | 22 (4.8) | 17 (5.8) | 5 (9.4) | 12 (5.0) | 13 (5.3) | 3 (10.0) | 10 (4.7) |
| Chronic kidney disease | 89 (16.5) | 24 (28.9) | 65 (14.3) | 59 (20.1) | 15 (28.3) | 44 (18.3) | 30 (12.2) | 9 (30.0) | 21 (9.8) |
| Type 2 diabetes | 125 (23.2) | 20 (24.1) | 105 (23.1) | 62 (21.2) | 13 (24.5) | 49 (20.4) | 63 (25.7) | 7 (23.3) | 56 (26.0) |
| Cancer | 132 (24.5) | 38 (45.8) | 94 (20.7) | 80 (27.3) | 21 (39.6) | 59 (24.6) | 52 (21.2) | 17 (56.7) | 35 (16.3) |
| Alternative IC ^h^ | 177 (32.9) | 83 (100.0) | 94 (20.7) | 112 (38.2) | 53 (100.0) | 59 (24.6) | 65 (26.5) | 30 (100.0) | 35 (16.3) |
| **Q3 2022** |  |  |  |  |  |  |  |  |  |
| **Total** | **387** | **85** | **302** | **160** | **38** | **122** | **227** | **47** | **180** |
| **DEMOGRAPHICS** | | | | | | | | | |
| **Age (years)** |  |  |  |  |  |  |  |  |  |
| Mean (SD) | 67.3 (16.3) | 62.9 (15.7) | 68.5 (16.2) | 69.4 (14.9) | 64.8 (13.2) | 70.8 (15.2) | 65.8 (17.0) | 61.4 (17.5) | 67.0 (16.8) |
| Median  (IQR) | 70.0  (58.0, 79.5) | 64.0  (56.0, 74.0) | 73.0  (58.2, 81.0) | 72.5  (60.8, 81.0) | 64.5  (59.0, 74.0) | 74.0  (62.0, 82.0) | 68.0  (56.0, 78.5) | 63.0  (55.5, 75.0) | 70.0  (57.0, 80.2) |
| Range | 18.0 - 96.0 | 18.0 - 89.0 | 18.0 - 96.0 | 19.0 - 94.0 | 35.0 - 89.0 | 19.0 - 94.0 | 18.0 - 96.0 | 18.0 - 87.0 | 18.0 - 96.0 |
| **Sex, n (%)** |  |  |  |  |  |  |  |  |  |
| Male | 219 (56.6) | 49 (57.6) | 170 (56.3) | 99 (61.9) | 21 (55.3) | 78 (63.9) | 120 (52.9) | 28 (59.6) | 92 (51.1) |
| Female | 168 (43.4) | 36 (42.4) | 132 (43.7) | 61 (38.1) | 17 (44.7) | 44 (36.1) | 107 (47.1) | 19 (40.4) | 88 (48.9) |
| **Country, n (%)** |  |  |  |  |  |  |  |  |  |
| Belgium | 25 (6.5) | 2 (2.4) | 23 (7.6) | 13 (8.1) | 1 (2.6) | 12 (9.8) | 12 (5.3) | 1 (2.1) | 11 (6.1) |
| Italy | 87 (22.5) | 9 (10.6) | 78 (25.8) | 46 (28.8) | 4 (10.5) | 42 (34.4) | 41 (18.1) | 5 (10.6) | 36 (20.0) |
| Spain | 275 (71.1) | 74 (87.1) | 201 (66.6) | 101 (63.1) | 33 (86.8) | 68 (55.7) | 174 (76.7) | 41 (87.2) | 133 (73.9) |
| **Study site, n (%)** |  |  |  |  |  |  |  |  |  |
| CIRI-IT | 87 (22.5) | 9 (10.6) | 78 (25.8) | 46 (28.8) | 4 (10.5) | 42 (34.4) | 41 (18.1) | 5 (10.6) | 36 (20.0) |
| GTPUH | 117 (30.2) | 22 (25.9) | 95 (31.5) | 23 (14.4) | 7 (18.4) | 16 (13.1) | 94 (41.4) | 15 (31.9) | 79 (43.9) |
| CHU Saint-Pierre | 9 (2.3) | 1 (1.2) | 8 (2.6) | 4 (2.5) | 0 (0.0) | 4 (3.3) | 5 (2.2) | 1 (2.1) | 4 (2.2) |
| UZA | 16 (4.1) | 1 (1.2) | 15 (5.0) | 9 (5.6) | 1 (2.6) | 8 (6.6) | 7 (3.1) | 0 (0.0) | 7 (3.9) |
| HUVH | 158 (40.8) | 52 (61.2) | 106 (35.1) | 78 (48.8) | 26 (68.4) | 52 (42.6) | 80 (35.2) | 26 (55.3) | 54 (30.0) |
| **Smoking, n (%)** |  |  |  |  |  |  |  |  |  |
| Never smoker | 171 (44.2) | 41 (48.2) | 130 (43.0) | 66 (41.2) | 17 (44.7) | 49 (40.2) | 105 (46.3) | 24 (51.1) | 81 (45.0) |
| Former smoker | 100 (25.8) | 17 (20.0) | 83 (27.5) | 44 (27.5) | 7 (18.4) | 37 (30.3) | 56 (24.7) | 10 (21.3) | 46 (25.6) |
| Current smoker | 78 (20.2) | 15 (17.6) | 63 (20.9) | 37 (23.1) | 8 (21.1) | 29 (23.8) | 41 (18.1) | 7 (14.9) | 34 (18.9) |
| Missing | 38 (9.8) | 12 (14.1) | 26 (8.6) | 13 (8.1) | 6 (15.8) | 7 (5.7) | 25 (11.0) | 6 (12.8) | 19 (10.6) |
| **Long-term care facility residence, n (%)** |  |  |  |  |  |  |  |  |  |
| Yes | 20 (5.3) | 1 (1.2) | 19 (6.4) | 8 (5.2) | 1 (2.9) | 7 (5.8) | 12 (5.4) | 0 (0.0) | 12 (6.9) |
| **VACCINATION STATUS AT TIME OF HOSPITAL ADMISSION** | | | | | | | | | |
| Unvaccinated, n (%) | 27 (7.0) | 5 (5.9) | 22 (7.3) | 14 (8.8) | 2 (5.3) | 12 (9.8) | 13 (5.7) | 3 (6.4) | 10 (5.6) |
| Incomplete primary series, n (%) | 7 (1.8) | 3 (3.5) | 4 (1.3) | 1 (0.6) | 1 (2.6) | 0 (0.0) | 6 (2.6) | 2 (4.3) | 4 (2.2) |
| Primary series completed but no boosters, n (%) | 32 (8.3) | 7 (8.2) | 25 (8.3) | 13 (8.1) | 3 (7.9) | 10 (8.2) | 19 (8.4) | 4 (8.5) | 15 (8.3) |
| At least one booster dose, n (%) | 321 (82.9) | 70 (82.4) | 251 (83.1) | 132 (82.5) | 32 (84.2) | 100 (82.0) | 189 (83.3) | 38 (80.9) | 151 (83.9) |
| **Number of booster doses ^c^, n (%)** |  |  |  |  |  |  |  |  |  |
| 0 | 32 (9.1) | 7 (9.1) | 25 (9.1) | 13 (9.0) | 3 (8.6) | 10 (9.1) | 19 (9.1) | 4 (9.5) | 15 (9.0) |
| 1 | 279 (79.0) | 50 (64.9) | 229 (83.0) | 109 (75.2) | 22 (62.9) | 87 (79.1) | 170 (81.7) | 28 (66.7) | 142 (85.5) |
| 2 | 42 (11.9) | 20 (26.0) | 22 (8.0) | 23 (15.9) | 10 (28.6) | 13 (11.8) | 19 (9.1) | 10 (23.8) | 9 (5.4) |
| 3+ | 0 (0.0) | 0 (0.0) | 0 (0.0) | 0 (0.0) | 0 (0.0) | 0 (0.0) | 0 (0.0) | 0 (0.0) | 0 (0.0) |
| **Time since last vaccine dose (days)** |  |  |  |  |  |  |  |  |  |
| Mean (SD) | 245.5 (85.7) | 242.8 (104.1) | 246.3 (79.9) | 223.2 (85.2) | 220.2 (109.9) | 224.2 (76.0) | 260.7 (82.9) | 261.3 (96.3) | 260.6 (79.3) |
| Median  (IQR) | 251.5  (202.8, 288.0) | 254.0  (165.0, 301.5) | 251.0  (209.8, 285.0) | 229.5  (178.2, 268.8) | 229.5  (148.5, 284.8) | 229.5  (190.0, 265.8) | 259.5  (220.5, 297.0) | 258.0  (193.2, 309.2) | 262.0  (228.2, 292.0) |
| Range | 1.0 - 529.0 | 1.0 - 522.0 | 1.0 - 529.0 | 1.0 - 494.0 | 1.0 - 494.0 | 7.0 - 487.0 | 1.0 - 529.0 | 80.0 - 522.0 | 1.0 - 529.0 |
| **Time since last vaccine dose, n (%)** |  |  |  |  |  |  |  |  |  |
| <2 months | 12 (3.3) | 3 (3.8) | 9 (3.2) | 8 (5.5) | 3 (8.3) | 5 (4.5) | 4 (1.9) | 0 (0.0) | 4 (2.4) |
| [2 - 4) months | 14 (3.9) | 7 (8.8) | 7 (2.5) | 8 (5.5) | 4 (11.1) | 4 (3.6) | 6 (2.8) | 3 (6.8) | 3 (1.8) |
| [4 - 6) months | 39 (10.8) | 13 (16.2) | 26 (9.3) | 22 (15.1) | 7 (19.4) | 15 (13.6) | 17 (7.9) | 6 (13.6) | 11 (6.5) |
| [6 - 8) months | 86 (23.9) | 12 (15.0) | 74 (26.4) | 41 (28.1) | 5 (13.9) | 36 (32.7) | 45 (21.0) | 7 (15.9) | 38 (22.4) |
| ≥8 months | 209 (58.1) | 45 (56.2) | 164 (58.6) | 67 (45.9) | 17 (47.2) | 50 (45.5) | 142 (66.4) | 28 (63.6) | 114 (67.1) |
| **SYMPTOMS** | | | | | | | | | |
| **SARI symptoms ^d,e^, n (%)** |  |  |  |  |  |  |  |  |  |
| Cough | 252 (65.6) | 50 (59.5) | 202 (67.3) | 105 (66.5) | 20 (54.1) | 85 (70.2) | 147 (65.0) | 30 (63.8) | 117 (65.4) |
| Fever | 240 (62.3) | 54 (64.3) | 186 (61.8) | 106 (66.7) | 26 (70.3) | 80 (65.6) | 134 (59.3) | 28 (59.6) | 106 (59.2) |
| Shortness of breath | 273 (70.9) | 56 (65.9) | 217 (72.3) | 110 (69.2) | 23 (60.5) | 87 (71.9) | 163 (72.1) | 33 (70.2) | 130 (72.6) |
| Anosmia, ageusia or dysgeusia | 15 (4.1) | 1 (1.2) | 14 (4.9) | 13 (8.6) | 0 (0.0) | 13 (11.4) | 2 (0.9) | 1 (2.1) | 1 (0.6) |
| **Number of SARI symptoms, n (%)** |  |  |  |  |  |  |  |  |  |
| 1 | 99 (27.2) | 23 (27.7) | 76 (27.0) | 45 (30.2) | 11 (30.6) | 34 (30.1) | 54 (25.1) | 12 (25.5) | 42 (25.0) |
| 2 | 173 (47.5) | 45 (54.2) | 128 (45.6) | 57 (38.3) | 20 (55.6) | 37 (32.7) | 116 (54.0) | 25 (53.2) | 91 (54.2) |
| 3+ | 92 (25.3) | 15 (18.1) | 77 (27.4) | 47 (31.5) | 5 (13.9) | 42 (37.2) | 45 (20.9) | 10 (21.3) | 35 (20.8) |
| **HOSPITAL OUTCOMES** | | | | | | | | | |
| **Length of stay (days)** |  |  |  |  |  |  |  |  |  |
| Mean (SD) | 10.4 (12.3) | 12.7 (15.3) | 9.7 (11.2) | 12.4 (16.0) | 15.6 (20.5) | 11.4 (14.3) | 9.0 (8.5) | 10.3 (9.0) | 8.6 (8.3) |
| Median  (IQR) | 7.0  (4.0, 12.0) | 8.0  (4.0, 12.0) | 6.0  (3.0, 12.0) | 7.0  (4.0, 14.2) | 7.5  (4.0, 18.8) | 6.0  (3.2, 13.0) | 7.0  (3.0, 11.0) | 8.0  (5.0, 11.5) | 6.0  (3.0, 11.0) |
| Range | 1.0 - 98.0 | 1.0 - 98.0 | 1.0 - 83.0 | 1.0 - 98.0 | 1.0 - 98.0 | 1.0 - 83.0 | 1.0 - 57.0 | 1.0 - 39.0 | 1.0 - 57.0 |
| **Severe outcomes, n (%)** |  |  |  |  |  |  |  |  |  |
| Hospitalization without ICU admission or in-hospital death | 344 (88.9) | 75 (88.2) | 269 (89.1) | 136 (85.0) | 32 (84.2) | 104 (85.2) | 208 (91.6) | 43 (91.5) | 165 (91.7) |
| ICU admission without in-hospital death | 16 (4.1) | 3 (3.5) | 13 (4.3) | 7 (4.4) | 1 (2.6) | 6 (4.9) | 9 (4.0) | 2 (4.3) | 7 (3.9) |
| ICU admission (with or without in-hospital death) | 25 (6.5) | 7 (8.2) | 18 (6.0) | 14 (8.8) | 5 (13.2) | 9 (7.4) | 11 (4.8) | 2 (4.3) | 9 (5.0) |
| In-hospital death | 27 (7.0) | 7 (8.2) | 20 (6.6) | 17 (10.6) | 5 (13.2) | 12 (9.8) | 10 (4.4) | 2 (4.3) | 8 (4.4) |
| **COMORBIDITIES** | | | | | | | | | |
| **Number of comorbidities other than IC, n (%)** |  |  |  |  |  |  |  |  |  |
| No comorbid conditions | 56 (14.5) | 9 (10.6) | 47 (15.6) | 14 (8.8) | 2 (5.3) | 12 (9.8) | 42 (18.5) | 7 (14.9) | 35 (19.4) |
| 1 comorbidity | 91 (23.5) | 20 (23.5) | 71 (23.5) | 34 (21.2) | 9 (23.7) | 25 (20.5) | 57 (25.1) | 11 (23.4) | 46 (25.6) |
| 2 comorbidities | 97 (25.1) | 22 (25.9) | 75 (24.8) | 49 (30.6) | 13 (34.2) | 36 (29.5) | 48 (21.1) | 9 (19.1) | 39 (21.7) |
| 3+ comorbidities | 143 (37.0) | 34 (40.0) | 109 (36.1) | 63 (39.4) | 14 (36.8) | 49 (40.2) | 80 (35.2) | 20 (42.6) | 60 (33.3) |
| **Number with specific comorbidities ^d^, n (%)** |  |  |  |  |  |  |  |  |  |
| Asthma | 28 (7.2) | 3 (3.5) | 25 (8.3) | 6 (3.8) | 0 (0.0) | 6 (4.9) | 22 (9.7) | 3 (6.4) | 19 (10.6) |
| Lung disease | 152 (39.3) | 44 (51.8) | 108 (35.8) | 65 (40.6) | 21 (55.3) | 44 (36.1) | 87 (38.3) | 23 (48.9) | 64 (35.6) |
| Cardiovascular disease | 150 (38.8) | 26 (30.6) | 124 (41.1) | 70 (43.8) | 12 (31.6) | 58 (47.5) | 80 (35.2) | 14 (29.8) | 66 (36.7) |
| Hypertension | 182 (47.0) | 31 (36.5) | 151 (50.0) | 81 (50.6) | 13 (34.2) | 68 (55.7) | 101 (44.5) | 18 (38.3) | 83 (46.1) |
| Chronic liver disease | 22 (5.7) | 8 (9.4) | 14 (4.6) | 11 (6.9) | 4 (10.5) | 7 (5.7) | 11 (4.8) | 4 (8.5) | 7 (3.9) |
| Chronic kidney disease | 73 (18.9) | 19 (22.4) | 54 (17.9) | 32 (20.0) | 7 (18.4) | 25 (20.5) | 41 (18.1) | 12 (25.5) | 29 (16.1) |
| Type 2 diabetes | 103 (26.6) | 22 (25.9) | 81 (26.8) | 50 (31.2) | 12 (31.6) | 38 (31.1) | 53 (23.3) | 10 (21.3) | 43 (23.9) |
| Cancer | 106 (27.4) | 48 (56.5) | 58 (19.2) | 56 (35.0) | 23 (60.5) | 33 (27.0) | 50 (22.0) | 25 (53.2) | 25 (13.9) |
| Alternative IC ^h^ | 143 (37.0) | 85 (100.0) | 58 (19.2) | 71 (44.4) | 38 (100.0) | 33 (27.0) | 72 (31.7) | 47 (100.0) | 25 (13.9) |
| **Q4 2022** |  |  |  |  |  |  |  |  |  |
| **Total** | **764** | **120** | **644** | **179** | **36** | **143** | **585** | **84** | **501** |
| **DEMOGRAPHICS** | | | | | | | | | |
| **Age (years)** |  |  |  |  |  |  |  |  |  |
| Mean (SD) | 66.6 (17.4) | 58.8 (16.1) | 68.0 (17.3) | 69.0 (15.9) | 62.9 (14.3) | 70.5 (16.0) | 65.9 (17.8) | 57.0 (16.6) | 67.3 (17.6) |
| Median  (IQR) | 69.5  (56.0, 79.0) | 60.0  (48.8, 70.0) | 72.0  (58.0, 81.0) | 72.0  (60.0, 80.0) | 66.0  (52.5, 73.8) | 74.0  (63.0, 82.0) | 68.0  (55.0, 79.0) | 58.5  (47.0, 69.0) | 71.0  (57.0, 80.0) |
| Range | 18.0 - 100.0 | 18.0 - 89.0 | 18.0 - 100.0 | 23.0 - 94.0 | 37.0 - 89.0 | 23.0 - 94.0 | 18.0 - 100.0 | 18.0 - 83.0 | 18.0 - 100.0 |
| **Sex, n (%)** |  |  |  |  |  |  |  |  |  |
| Male | 409 (53.5) | 67 (55.8) | 342 (53.1) | 104 (58.1) | 24 (66.7) | 80 (55.9) | 305 (52.1) | 43 (51.2) | 262 (52.3) |
| Female | 355 (46.5) | 53 (44.2) | 302 (46.9) | 75 (41.9) | 12 (33.3) | 63 (44.1) | 280 (47.9) | 41 (48.8) | 239 (47.7) |
| **Country, n (%)** |  |  |  |  |  |  |  |  |  |
| Belgium | 74 (9.7) | 7 (5.8) | 67 (10.4) | 13 (7.3) | 2 (5.6) | 11 (7.7) | 61 (10.4) | 5 (6.0) | 56 (11.2) |
| Italy | 170 (22.3) | 9 (7.5) | 161 (25.0) | 49 (27.4) | 4 (11.1) | 45 (31.5) | 121 (20.7) | 5 (6.0) | 116 (23.2) |
| Spain | 520 (68.1) | 104 (86.7) | 416 (64.6) | 117 (65.4) | 30 (83.3) | 87 (60.8) | 403 (68.9) | 74 (88.1) | 329 (65.7) |
| **Study site, n (%)** |  |  |  |  |  |  |  |  |  |
| CIRI-IT | 170 (22.3) | 9 (7.5) | 161 (25.0) | 49 (27.4) | 4 (11.1) | 45 (31.5) | 121 (20.7) | 5 (6.0) | 116 (23.2) |
| GTPUH | 125 (16.4) | 21 (17.5) | 104 (16.1) | 21 (11.7) | 1 (2.8) | 20 (14.0) | 104 (17.8) | 20 (23.8) | 84 (16.8) |
| CHU Saint-Pierre | 44 (5.8) | 2 (1.7) | 42 (6.5) | 8 (4.5) | 0 (0.0) | 8 (5.6) | 36 (6.2) | 2 (2.4) | 34 (6.8) |
| UZA | 30 (3.9) | 5 (4.2) | 25 (3.9) | 5 (2.8) | 2 (5.6) | 3 (2.1) | 25 (4.3) | 3 (3.6) | 22 (4.4) |
| HUVH | 395 (51.7) | 83 (69.2) | 312 (48.4) | 96 (53.6) | 29 (80.6) | 67 (46.9) | 299 (51.1) | 54 (64.3) | 245 (48.9) |
| **Smoking, n (%)** |  |  |  |  |  |  |  |  |  |
| Never smoker | 310 (40.6) | 49 (40.8) | 261 (40.5) | 72 (40.2) | 18 (50.0) | 54 (37.8) | 238 (40.7) | 31 (36.9) | 207 (41.3) |
| Former smoker | 197 (25.8) | 30 (25.0) | 167 (25.9) | 48 (26.8) | 5 (13.9) | 43 (30.1) | 149 (25.5) | 25 (29.8) | 124 (24.8) |
| Current smoker | 206 (27.0) | 27 (22.5) | 179 (27.8) | 43 (24.0) | 5 (13.9) | 38 (26.6) | 163 (27.9) | 22 (26.2) | 141 (28.1) |
| Missing | 51 (6.7) | 14 (11.7) | 37 (5.7) | 16 (8.9) | 8 (22.2) | 8 (5.6) | 35 (6.0) | 6 (7.1) | 29 (5.8) |
| **Long-term care facility residence, n (%)** |  |  |  |  |  |  |  |  |  |
| Yes | 22 (2.9) | 1 (0.8) | 21 (3.3) | 5 (2.8) | 0 (0.0) | 5 (3.6) | 17 (3.0) | 1 (1.2) | 16 (3.3) |
| **VACCINATION STATUS AT TIME OF HOSPITAL ADMISSION** | | | | | | | | | |
| Unvaccinated, n (%) | 51 (6.7) | 6 (5.0) | 45 (7.0) | 11 (6.1) | 2 (5.6) | 9 (6.3) | 40 (6.8) | 4 (4.8) | 36 (7.2) |
| Incomplete primary series, n (%) | 7 (0.9) | 0 (0.0) | 7 (1.1) | 0 (0.0) | 0 (0.0) | 0 (0.0) | 7 (1.2) | 0 (0.0) | 7 (1.4) |
| Primary series completed but no boosters, n (%) | 92 (12.0) | 17 (14.2) | 75 (11.6) | 21 (11.7) | 2 (5.6) | 19 (13.3) | 71 (12.1) | 15 (17.9) | 56 (11.2) |
| At least one booster dose, n (%) | 614 (80.4) | 97 (80.8) | 517 (80.3) | 147 (82.1) | 32 (88.9) | 115 (80.4) | 467 (79.8) | 65 (77.4) | 402 (80.2) |
| **Number of booster doses ^c^, n (%)** |  |  |  |  |  |  |  |  |  |
| 0 | 92 (13.0) | 17 (14.9) | 75 (12.7) | 21 (12.5) | 2 (5.9) | 19 (14.2) | 71 (13.2) | 15 (18.8) | 56 (12.2) |
| 1 | 346 (49.0) | 53 (46.5) | 293 (49.5) | 76 (45.2) | 14 (41.2) | 62 (46.3) | 270 (50.2) | 39 (48.8) | 231 (50.4) |
| 2 | 251 (35.6) | 33 (28.9) | 218 (36.8) | 62 (36.9) | 11 (32.4) | 51 (38.1) | 189 (35.1) | 22 (27.5) | 167 (36.5) |
| 3+ | 17 (2.4) | 11 (9.6) | 6 (1.0) | 9 (5.4) | 7 (20.6) | 2 (1.5) | 8 (1.5) | 4 (5.0) | 4 (0.9) |
| **Time since last vaccine dose (days)** |  |  |  |  |  |  |  |  |  |
| Mean (SD) | 253.5 (154.4) | 280.6 (139.0) | 248.3 (156.7) | 264.9 (150.7) | 268.8 (107.6) | 263.9 (160.2) | 250.0 (155.4) | 285.6 (150.7) | 243.8 (155.6) |
| Median  (IQR) | 296.0  (77.0, 364.0) | 286.5  (197.0, 372.2) | 300.0  (67.5, 361.5) | 301.0  (135.8, 366.0) | 253.5  (216.0, 321.5) | 318.0  (75.2, 366.8) | 295.0  (73.0, 361.0) | 295.0  (169.2, 384.0) | 295.0  (64.0, 358.0) |
| Range | 2.0 - 606.0 | 23.0 - 606.0 | 2.0 - 576.0 | 2.0 - 575.0 | 23.0 - 498.0 | 2.0 - 575.0 | 2.0 - 606.0 | 23.0 - 606.0 | 2.0 - 576.0 |
| **Time since last vaccine dose, n (%)** |  |  |  |  |  |  |  |  |  |
| <2 months | 145 (20.3) | 10 (8.8) | 135 (22.5) | 27 (16.1) | 2 (5.9) | 25 (18.7) | 118 (21.7) | 8 (10.0) | 110 (23.7) |
| [2 - 4) months | 59 (8.3) | 7 (6.1) | 52 (8.7) | 14 (8.3) | 1 (2.9) | 13 (9.7) | 45 (8.3) | 6 (7.5) | 39 (8.4) |
| [4 - 6) months | 34 (4.8) | 8 (7.0) | 26 (4.3) | 8 (4.8) | 1 (2.9) | 7 (5.2) | 26 (4.8) | 7 (8.8) | 19 (4.1) |
| [6 - 8) months | 40 (5.6) | 17 (14.9) | 23 (3.8) | 16 (9.5) | 10 (29.4) | 6 (4.5) | 24 (4.4) | 7 (8.8) | 17 (3.7) |
| ≥8 months | 435 (61.0) | 72 (63.2) | 363 (60.6) | 103 (61.3) | 20 (58.8) | 83 (61.9) | 332 (60.9) | 52 (65.0) | 280 (60.2) |
| **SYMPTOMS** | | | | | | | | | |
| **SARI symptoms ^d,e^, n (%)** |  |  |  |  |  |  |  |  |  |
| Cough | 629 (82.3) | 91 (75.8) | 538 (83.5) | 140 (78.2) | 27 (75.0) | 113 (79.0) | 489 (83.6) | 64 (76.2) | 425 (84.8) |
| Fever | 485 (64.2) | 76 (63.3) | 409 (64.3) | 116 (64.8) | 24 (66.7) | 92 (64.3) | 369 (64.0) | 52 (61.9) | 317 (64.3) |
| Shortness of breath | 519 (68.3) | 80 (66.7) | 439 (68.6) | 125 (69.8) | 21 (58.3) | 104 (72.7) | 394 (67.8) | 59 (70.2) | 335 (67.4) |
| Anosmia, ageusia or dysgeusia | 9 (1.2) | 3 (2.6) | 6 (1.0) | 6 (3.4) | 1 (2.9) | 5 (3.6) | 3 (0.5) | 2 (2.5) | 1 (0.2) |
| **Number of SARI symptoms, n (%)** |  |  |  |  |  |  |  |  |  |
| 1 | 105 (14.4) | 28 (24.3) | 77 (12.5) | 31 (17.8) | 10 (29.4) | 21 (15.0) | 74 (13.3) | 18 (22.2) | 56 (11.8) |
| 2 | 414 (56.6) | 52 (45.2) | 362 (58.8) | 88 (50.6) | 14 (41.2) | 74 (52.9) | 326 (58.5) | 38 (46.9) | 288 (60.5) |
| 3+ | 212 (29.0) | 35 (30.4) | 177 (28.7) | 55 (31.6) | 10 (29.4) | 45 (32.1) | 157 (28.2) | 25 (30.9) | 132 (27.7) |
| **HOSPITAL OUTCOMES** | | | | | | | | | |
| **Length of stay (days)** |  |  |  |  |  |  |  |  |  |
| Mean (SD) | 9.7 (14.8) | 11.8 (18.2) | 9.4 (14.1) | 10.8 (16.9) | 9.6 (10.2) | 11.1 (18.2) | 9.4 (14.1) | 12.7 (20.7) | 8.8 (12.7) |
| Median  (IQR) | 6.0  (4.0, 11.0) | 7.0  (4.8, 13.2) | 6.0  (4.0, 11.0) | 8.0  (4.0, 13.0) | 6.0  (4.0, 12.5) | 8.0  (4.5, 13.0) | 6.0  (4.0, 10.0) | 7.0  (5.0, 13.2) | 6.0  (4.0, 10.0) |
| Range | 1.0 - 201.0 | 1.0 - 173.0 | 1.0 - 201.0 | 1.0 - 201.0 | 1.0 - 52.0 | 1.0 - 201.0 | 1.0 - 173.0 | 1.0 - 173.0 | 1.0 - 166.0 |
| **Severe outcomes, n (%)** |  |  |  |  |  |  |  |  |  |
| Hospitalization without ICU admission or in-hospital death | 691 (90.4) | 101 (84.2) | 590 (91.6) | 151 (84.4) | 29 (80.6) | 122 (85.3) | 540 (92.3) | 72 (85.7) | 468 (93.4) |
| ICU admission without in-hospital death | 36 (4.7) | 7 (5.8) | 29 (4.5) | 10 (5.6) | 3 (8.3) | 7 (4.9) | 26 (4.4) | 4 (4.8) | 22 (4.4) |
| ICU admission (with or without in-hospital death) | 45 (5.9) | 12 (10.0) | 33 (5.1) | 14 (7.8) | 5 (13.9) | 9 (6.3) | 31 (5.3) | 7 (8.3) | 24 (4.8) |
| In-hospital death | 37 (4.8) | 12 (10.0) | 25 (3.9) | 18 (10.1) | 4 (11.1) | 14 (9.8) | 19 (3.2) | 8 (9.5) | 11 (2.2) |
| **COMORBIDITIES** | | | | | | | | | |
| **Number of comorbidities other than IC, n (%)** |  |  |  |  |  |  |  |  |  |
| No comorbid conditions | 125 (16.4) | 11 (9.2) | 114 (17.7) | 15 (8.4) | 2 (5.6) | 13 (9.1) | 110 (18.8) | 9 (10.7) | 101 (20.2) |
| 1 comorbidity | 166 (21.7) | 28 (23.3) | 138 (21.4) | 36 (20.1) | 10 (27.8) | 26 (18.2) | 130 (22.2) | 18 (21.4) | 112 (22.4) |
| 2 comorbidities | 177 (23.2) | 25 (20.8) | 152 (23.6) | 58 (32.4) | 10 (27.8) | 48 (33.6) | 119 (20.3) | 15 (17.9) | 104 (20.8) |
| 3+ comorbidities | 296 (38.7) | 56 (46.7) | 240 (37.3) | 70 (39.1) | 14 (38.9) | 56 (39.2) | 226 (38.6) | 42 (50.0) | 184 (36.7) |
| **Number with specific comorbidities ^d^, n (%)** |  |  |  |  |  |  |  |  |  |
| Asthma | 71 (9.3) | 6 (5.0) | 65 (10.1) | 13 (7.3) | 1 (2.8) | 12 (8.4) | 58 (9.9) | 5 (6.0) | 53 (10.6) |
| Lung disease | 289 (37.8) | 51 (42.5) | 238 (37.0) | 68 (38.0) | 12 (33.3) | 56 (39.2) | 221 (37.8) | 39 (46.4) | 182 (36.3) |
| Cardiovascular disease | 313 (41.0) | 51 (42.5) | 262 (40.7) | 75 (41.9) | 13 (36.1) | 62 (43.4) | 238 (40.7) | 38 (45.2) | 200 (39.9) |
| Hypertension | 372 (48.7) | 52 (43.3) | 320 (49.7) | 104 (58.1) | 16 (44.4) | 88 (61.5) | 268 (45.8) | 36 (42.9) | 232 (46.3) |
| Chronic liver disease | 71 (9.3) | 18 (15.0) | 53 (8.2) | 15 (8.4) | 4 (11.1) | 11 (7.7) | 56 (9.6) | 14 (16.7) | 42 (8.4) |
| Chronic kidney disease | 132 (17.3) | 41 (34.2) | 91 (14.1) | 43 (24.0) | 16 (44.4) | 27 (18.9) | 89 (15.2) | 25 (29.8) | 64 (12.8) |
| Type 2 diabetes | 204 (26.7) | 33 (27.5) | 171 (26.6) | 48 (26.8) | 8 (22.2) | 40 (28.0) | 156 (26.7) | 25 (29.8) | 131 (26.1) |
| Cancer | 159 (20.8) | 54 (45.0) | 105 (16.3) | 45 (25.1) | 18 (50.0) | 27 (18.9) | 114 (19.5) | 36 (42.9) | 78 (15.6) |
| Alternative IC ^h^ | 225 (29.5) | 120 (100.0) | 105 (16.3) | 63 (35.2) | 36 (100.0) | 27 (18.9) | 162 (27.7) | 84 (100.0) | 78 (15.6) |
| **Q1 2023** |  |  |  |  |  |  |  |  |  |
| **Total** | **710** | **83** | **627** | **116** | **18** | **98** | **279** | **38** | **529** |
| **DEMOGRAPHICS** | | | | | | | | | |
| **Age (years)** |  |  |  |  |  |  |  |  |  |
| Mean (SD) | 68.7 (16.5) | 62.5 (13.4) | 69.6 (16.7) | 74.2 (13.4) | 68.9 (10.4) | 75.2 (13.7) | 67.7 (16.8) | 60.8 (13.7) | 68.5 (17.0) |
| Median  (IQR) | 71.5  (58.0, 82.0) | 63.0  (55.0, 71.0) | 73.0  (60.0, 82.0) | 76.5  (67.0, 84.0) | 68.0  (61.8, 74.8) | 78.0  (69.0, 85.0) | 70.0  (57.0, 81.0) | 61.0  (53.0, 69.0) | 72.0  (58.0, 82.0) |
| Range | 18.0 - 99.0 | 27.0 - 89.0 | 18.0 - 99.0 | 23.0 - 98.0 | 51.0 - 87.0 | 23.0 - 98.0 | 18.0 - 99.0 | 27.0 - 89.0 | 18.0 - 99.0 |
| **Sex, n (%)** |  |  |  |  |  |  |  |  |  |
| Male | 380 (53.5) | 51 (61.4) | 329 (52.5) | 65 (56.0) | 13 (72.2) | 52 (53.1) | 315 (53.0) | 38 (58.5) | 277 (52.4) |
| Female | 330 (46.5) | 32 (38.6) | 298 (47.5) | 51 (44.0) | 5 (27.8) | 46 (46.9) | 279 (47.0) | 27 (41.5) | 252 (47.6) |
| **Country, n (%)** |  |  |  |  |  |  |  |  |  |
| Belgium | 57 (8.0) | 7 (8.4) | 50 (8.0) | 10 (8.6) | 2 (11.1) | 8 (8.2) | 47 (7.9) | 5 (7.7) | 42 (7.9) |
| Italy | 193 (27.2) | 11 (13.3) | 182 (29.0) | 32 (27.6) | 1 (5.6) | 31 (31.6) | 161 (27.1) | 10 (15.4) | 151 (28.5) |
| Spain | 460 (64.8) | 65 (78.3) | 395 (63.0) | 74 (63.8) | 15 (83.3) | 59 (60.2) | 386 (65.0) | 50 (76.9) | 336 (63.5) |
| **Study site, n (%)** |  |  |  |  |  |  |  |  |  |
| CIRI-IT | 193 (27.2) | 11 (13.3) | 182 (29.0) | 32 (27.6) | 1 (5.6) | 31 (31.6) | 161 (27.1) | 10 (15.4) | 151 (28.5) |
| GTPUH | 127 (17.9) | 20 (24.1) | 107 (17.1) | 15 (12.9) | 4 (22.2) | 11 (11.2) | 112 (18.9) | 16 (24.6) | 96 (18.1) |
| CHU Saint-Pierre | 25 (3.5) | 1 (1.2) | 24 (3.8) | 3 (2.6) | 0 (0.0) | 3 (3.1) | 22 (3.7) | 1 (1.5) | 21 (4.0) |
| UZA | 32 (4.5) | 6 (7.2) | 26 (4.1) | 7 (6.0) | 2 (11.1) | 5 (5.1) | 25 (4.2) | 4 (6.2) | 21 (4.0) |
| HUVH | 333 (46.9) | 45 (54.2) | 288 (45.9) | 59 (50.9) | 11 (61.1) | 48 (49.0) | 274 (46.1) | 34 (52.3) | 240 (45.4) |
| **Smoking, n (%)** |  |  |  |  |  |  |  |  |  |
| Never smoker | 307 (43.2) | 40 (48.2) | 267 (42.6) | 57 (49.1) | 11 (61.1) | 46 (46.9) | 250 (42.1) | 29 (44.6) | 221 (41.8) |
| Former smoker | 210 (29.6) | 21 (25.3) | 189 (30.1) | 37 (31.9) | 3 (16.7) | 34 (34.7) | 173 (29.1) | 18 (27.7) | 155 (29.3) |
| Current smoker | 159 (22.4) | 13 (15.7) | 146 (23.3) | 17 (14.7) | 2 (11.1) | 15 (15.3) | 142 (23.9) | 11 (16.9) | 131 (24.8) |
| Missing | 34 (4.8) | 9 (10.8) | 25 (4.0) | 5 (4.3) | 2 (11.1) | 3 (3.1) | 29 (4.9) | 7 (10.8) | 22 (4.2) |
| **Long-term care facility residence, n (%)** |  |  |  |  |  |  |  |  |  |
| Yes | 23 (3.3) | 1 (1.3) | 22 (3.5) | 3 (2.7) | 0 (0.0) | 3 (3.1) | 20 (3.4) | 1 (1.6) | 19 (3.6) |
| **VACCINATION STATUS AT TIME OF HOSPITAL ADMISSION** | | | | | | | | | |
| Unvaccinated, n (%) | 45 (6.3) | 3 (3.6) | 42 (6.7) | 9 (7.8) | 0 (0.0) | 9 (9.2) | 36 (6.1) | 3 (4.6) | 33 (6.2) |
| Incomplete primary series, n (%) | 11 (1.5) | 1 (1.2) | 10 (1.6) | 1 (0.9) | 0 (0.0) | 1 (1.0) | 10 (1.7) | 1 (1.5) | 9 (1.7) |
| Primary series completed but no boosters, n (%) | 67 (9.4) | 10 (12.0) | 57 (9.1) | 9 (7.8) | 1 (5.6) | 8 (8.2) | 58 (9.8) | 9 (13.8) | 49 (9.3) |
| At least one booster dose, n (%) | 587 (82.7) | 69 (83.1) | 518 (82.6) | 97 (83.6) | 17 (94.4) | 80 (81.6) | 490 (82.5) | 52 (80.0) | 438 (82.8) |
| **Number of booster doses ^c^, n (%)** |  |  |  |  |  |  |  |  |  |
| 0 | 67 (10.2) | 10 (12.7) | 57 (9.9) | 9 (8.5) | 1 (5.6) | 8 (9.1) | 58 (10.6) | 9 (14.8) | 49 (10.1) |
| 1 | 253 (38.7) | 21 (26.6) | 232 (40.3) | 31 (29.2) | 4 (22.2) | 27 (30.7) | 222 (40.5) | 17 (27.9) | 205 (42.1) |
| 2 | 311 (47.6) | 36 (45.6) | 275 (47.8) | 59 (55.7) | 7 (38.9) | 52 (59.1) | 252 (46.0) | 29 (47.5) | 223 (45.8) |
| 3+ | 23 (3.5) | 12 (15.2) | 11 (1.9) | 7 (6.6) | 6 (33.3) | 1 (1.1) | 16 (2.9) | 6 (9.8) | 10 (2.1) |
| **Time since last vaccine dose (days)** |  |  |  |  |  |  |  |  |  |
| Mean (SD) | 288.8 (171.7) | 314.4 (167.9) | 285.3 (172.1) | 274.8 (164.3) | 321.8 (153.4) | 265.3 (165.6) | 291.5 (173.1) | 312.2 (172.9) | 288.9 (173.2) |
| Median  (IQR) | 296.0  (125.0, 431.0) | 322.5  (150.8, 443.0) | 282.0  (124.0, 427.0) | 230.0  (129.0, 425.5) | 342.0  (194.2, 415.5) | 181.0  (128.0, 425.0) | 318.5  (125.0, 431.8) | 318.5  (136.5, 455.5) | 318.0  (123.0, 431.0) |
| Range | 10.0 - 731.0 | 69.0 - 662.0 | 10.0 - 731.0 | 42.0 - 662.0 | 105.0 - 662.0 | 42.0 - 653.0 | 10.0 - 731.0 | 69.0 - 652.0 | 10.0 - 731.0 |
| **Time since last vaccine dose, n (%)** |  |  |  |  |  |  |  |  |  |
| <2 months | 19 (2.9) | 0 (0.0) | 19 (3.2) | 2 (1.9) | 0 (0.0) | 2 (2.2) | 17 (3.0) | 0 (0.0) | 17 (3.4) |
| [2 - 4) months | 127 (19.1) | 13 (16.2) | 114 (19.5) | 19 (17.8) | 2 (11.1) | 17 (19.1) | 108 (19.4) | 11 (17.7) | 97 (19.6) |
| [4 - 6) months | 139 (20.9) | 12 (15.0) | 127 (21.7) | 28 (26.2) | 3 (16.7) | 25 (28.1) | 111 (19.9) | 9 (14.5) | 102 (20.6) |
| [6 - 8) months | 23 (3.5) | 5 (6.2) | 18 (3.1) | 5 (4.7) | 0 (0.0) | 5 (5.6) | 18 (3.2) | 5 (8.1) | 13 (2.6) |
| ≥8 months | 357 (53.7) | 50 (62.5) | 307 (52.5) | 53 (49.5) | 13 (72.2) | 40 (44.9) | 304 (54.5) | 37 (59.7) | 267 (53.8) |
| **SYMPTOMS** | | | | | | | | | |
| **SARI symptoms ^d,e^, n (%)** |  |  |  |  |  |  |  |  |  |
| Cough | 600 (84.5) | 69 (83.1) | 531 (84.7) | 93 (80.2) | 15 (83.3) | 78 (79.6) | 507 (85.4) | 54 (83.1) | 453 (85.6) |
| Fever | 464 (66.3) | 61 (74.4) | 403 (65.2) | 77 (67.5) | 11 (61.1) | 66 (68.8) | 387 (66.0) | 50 (78.1) | 337 (64.6) |
| Shortness of breath | 454 (64.0) | 47 (57.3) | 407 (64.9) | 68 (58.6) | 9 (50.0) | 59 (60.2) | 386 (65.1) | 38 (59.4) | 348 (65.8) |
| Anosmia, ageusia or dysgeusia | 11 (1.6) | 2 (2.6) | 9 (1.5) | 8 (7.3) | 0 (0.0) | 8 (8.6) | 3 (0.5) | 2 (3.3) | 1 (0.2) |
| **Number of SARI symptoms, n (%)** |  |  |  |  |  |  |  |  |  |
| 1 | 95 (14.1) | 11 (14.5) | 84 (14.1) | 23 (21.1) | 3 (18.8) | 20 (21.5) | 72 (12.8) | 8 (13.3) | 64 (12.7) |
| 2 | 381 (56.7) | 42 (55.3) | 339 (56.9) | 53 (48.6) | 11 (68.8) | 42 (45.2) | 328 (58.3) | 31 (51.7) | 297 (59.0) |
| 3+ | 196 (29.2) | 23 (30.3) | 173 (29.0) | 33 (30.3) | 2 (12.5) | 31 (33.3) | 163 (29.0) | 21 (35.0) | 142 (28.2) |
| **HOSPITAL OUTCOMES** | | | | | | | | | |
| **Length of stay (days)** |  |  |  |  |  |  |  |  |  |
| Mean (SD) | 9.0 (11.1) | 8.2 (8.8) | 9.1 (11.3) | 10.3 (12.9) | 9.3 (13.6) | 10.5 (12.9) | 8.7 (10.6) | 7.8 (6.9) | 8.8 (11.0) |
| Median  (IQR) | 6.0  (3.0, 10.0) | 5.0  (3.0, 10.0) | 6.0  (3.0, 11.0) | 6.0  (3.0, 12.0) | 5.0  (3.0, 7.0) | 6.0  (3.0, 12.8) | 6.0  (3.0, 10.0) | 5.0  (3.0, 11.0) | 6.0  (4.0, 10.0) |
| Range | 1.0 - 155.0 | 1.0 - 51.0 | 1.0 - 155.0 | 1.0 - 92.0 | 1.0 - 51.0 | 1.0 - 92.0 | 1.0 - 155.0 | 1.0 - 34.0 | 1.0 - 155.0 |
| **Severe outcomes, n (%)** |  |  |  |  |  |  |  |  |  |
| Hospitalization without ICU admission or in-hospital death | 647 (91.1) | 78 (94.0) | 569 (90.7) | 109 (94.0) | 18 (100.0) | 91 (92.9) | 538 (90.6) | 60 (92.3) | 478 (90.4) |
| ICU admission without in-hospital death | 32 (4.5) | 4 (4.8) | 28 (4.5) | 5 (4.3) | 0 (0.0) | 5 (5.1) | 27 (4.5) | 4 (6.2) | 23 (4.3) |
| ICU admission (with or without in-hospital death) | 37 (5.2) | 5 (6.0) | 32 (5.1) | 5 (4.3) | 0 (0.0) | 5 (5.1) | 32 (5.4) | 5 (7.7) | 27 (5.1) |
| In-hospital death | 31 (4.4) | 1 (1.2) | 30 (4.8) | 2 (1.7) | 0 (0.0) | 2 (2.0) | 29 (4.9) | 1 (1.5) | 28 (5.3) |
| **COMORBIDITIES** | | | | | | | | | |
| **Number of comorbidities other than IC, n (%)** |  |  |  |  |  |  |  |  |  |
| No comorbid conditions | 132 (18.6) | 8 (9.6) | 124 (19.8) | 15 (12.9) | 0 (0.0) | 15 (15.3) | 117 (19.7) | 8 (12.3) | 109 (20.6) |
| 1 comorbidity | 133 (18.7) | 15 (18.1) | 118 (18.8) | 22 (19.0) | 5 (27.8) | 17 (17.3) | 111 (18.7) | 10 (15.4) | 101 (19.1) |
| 2 comorbidities | 161 (22.7) | 29 (34.9) | 132 (21.1) | 26 (22.4) | 4 (22.2) | 22 (22.4) | 135 (22.7) | 25 (38.5) | 110 (20.8) |
| 3+ comorbidities | 284 (40.0) | 31 (37.3) | 253 (40.4) | 53 (45.7) | 9 (50.0) | 44 (44.9) | 231 (38.9) | 22 (33.8) | 209 (39.5) |
| **Number with specific comorbidities ^d^, n (%)** |  |  |  |  |  |  |  |  |  |
| Asthma | 61 (8.6) | 5 (6.0) | 56 (8.9) | 3 (2.6) | 0 (0.0) | 3 (3.1) | 58 (9.8) | 5 (7.7) | 53 (10.0) |
| Lung disease | 259 (36.5) | 35 (42.2) | 224 (35.7) | 42 (36.2) | 9 (50.0) | 33 (33.7) | 217 (36.5) | 26 (40.0) | 191 (36.1) |
| Cardiovascular disease | 300 (42.3) | 30 (36.1) | 270 (43.1) | 59 (50.9) | 8 (44.4) | 51 (52.0) | 241 (40.6) | 22 (33.8) | 219 (41.4) |
| Hypertension | 361 (50.8) | 37 (44.6) | 324 (51.7) | 75 (64.7) | 11 (61.1) | 64 (65.3) | 286 (48.1) | 26 (40.0) | 260 (49.1) |
| Chronic liver disease | 63 (8.9) | 10 (12.0) | 53 (8.5) | 6 (5.2) | 0 (0.0) | 6 (6.1) | 57 (9.6) | 10 (15.4) | 47 (8.9) |
| Chronic kidney disease | 105 (14.8) | 22 (26.5) | 83 (13.2) | 25 (21.6) | 5 (27.8) | 20 (20.4) | 80 (13.5) | 17 (26.2) | 63 (11.9) |
| Type 2 diabetes | 189 (26.6) | 19 (22.9) | 170 (27.1) | 30 (25.9) | 5 (27.8) | 25 (25.5) | 159 (26.8) | 14 (21.5) | 145 (27.4) |
| Cancer | 154 (21.7) | 33 (39.8) | 121 (19.3) | 28 (24.1) | 7 (38.9) | 21 (21.4) | 126 (21.2) | 26 (40.0) | 100 (18.9) |
| Alternative IC ^h^ | 204 (28.7) | 83 (100.0) | 121 (19.3) | 39 (33.6) | 18 (100.0) | 21 (21.4) | 165 (27.8) | 65 (100.0) | 100 (18.9) |
| **Q2 2023** |  |  |  |  |  |  |  |  |  |
| **Total** | **278** | **41** | **237** | **47** | **5** | **42** | **231** | **36** | **195** |
| **DEMOGRAPHICS** | | | | | | | | | |
| **Age (years)** |  |  |  |  |  |  |  |  |  |
| Mean (SD) | 66.0 (16.9) | 62.3 (16.3) | 66.6 (17.0) | 70.4 (15.8) | 66.4 (18.0) | 70.9 (15.7) | 65.1 (17.0) | 61.7 (16.3) | 65.7 (17.1) |
| Median  (IQR) | 68.0  (58.0, 79.0) | 66.0  (57.0, 72.0) | 69.0  (58.0, 80.0) | 73.0  (60.5, 81.5) | 67.0  (63.0, 72.0) | 74.0  (59.8, 81.8) | 68.0  (58.0, 77.0) | 66.0  (56.8, 72.2) | 68.0  (58.0, 78.5) |
| Range | 18.0 - 97.0 | 19.0 - 90.0 | 18.0 - 97.0 | 27.0 - 97.0 | 40.0 - 90.0 | 27.0 - 97.0 | 18.0 - 94.0 | 19.0 - 83.0 | 18.0 - 94.0 |
| **Sex, n (%)** |  |  |  |  |  |  |  |  |  |
| Male | 166 (59.7) | 24 (58.5) | 142 (59.9) | 28 (59.6) | 4 (80.0) | 24 (57.1) | 138 (59.7) | 20 (55.6) | 118 (60.5) |
| Female | 112 (40.3) | 17 (41.5) | 95 (40.1) | 19 (40.4) | 1 (20.0) | 18 (42.9) | 93 (40.3) | 16 (44.4) | 77 (39.5) |
| **Country, n (%)** |  |  |  |  |  |  |  |  |  |
| Belgium | 17 (6.1) | 1 (2.4) | 16 (6.8) | 2 (4.3) | 0 (0.0) | 2 (4.8) | 15 (6.5) | 1 (2.8) | 14 (7.2) |
| Italy | 37 (13.3) | 2 (4.9) | 35 (14.8) | 5 (10.6) | 0 (0.0) | 5 (11.9) | 32 (13.9) | 2 (5.6) | 30 (15.4) |
| Spain | 224 (80.6) | 38 (92.7) | 186 (78.5) | 40 (85.1) | 5 (100.0) | 35 (83.3) | 184 (79.7) | 33 (91.7) | 151 (77.4) |
| **Study site, n (%)** |  |  |  |  |  |  |  |  |  |
| CIRI-IT | 37 (13.3) | 2 (4.9) | 35 (14.8) | 5 (10.6) | 0 (0.0) | 5 (11.9) | 32 (13.9) | 2 (5.6) | 30 (15.4) |
| GTPUH | 49 (17.6) | 11 (26.8) | 38 (16.0) | 6 (12.8) | 2 (40.0) | 4 (9.5) | 43 (18.6) | 9 (25.0) | 34 (17.4) |
| CHU Saint-Pierre | 11 (4.0) | 0 (0.0) | 11 (4.6) | 1 (2.1) | 0 (0.0) | 1 (2.4) | 10 (4.3) | 0 (0.0) | 10 (5.1) |
| UZA | 6 (2.2) | 1 (2.4) | 5 (2.1) | 1 (2.1) | 0 (0.0) | 1 (2.4) | 5 (2.2) | 1 (2.8) | 4 (2.1) |
| HUVH | 175 (62.9) | 27 (65.9) | 148 (62.4) | 34 (72.3) | 3 (60.0) | 31 (73.8) | 141 (61.0) | 24 (66.7) | 117 (60.0) |
| **Smoking, n (%)** |  |  |  |  |  |  |  |  |  |
| Never smoker | 103 (37.1) | 17 (41.5) | 86 (36.3) | 19 (40.4) | 3 (60.0) | 16 (38.1) | 84 (36.4) | 14 (38.9) | 70 (35.9) |
| Former smoker | 92 (33.1) | 15 (36.6) | 77 (32.5) | 18 (38.3) | 0 (0.0) | 18 (42.9) | 74 (32.0) | 15 (41.7) | 59 (30.3) |
| Current smoker | 68 (24.5) | 6 (14.6) | 62 (26.2) | 8 (17.0) | 1 (20.0) | 7 (16.7) | 60 (26.0) | 5 (13.9) | 55 (28.2) |
| Missing | 15 (5.4) | 3 (7.3) | 12 (5.1) | 2 (4.3) | 1 (20.0) | 1 (2.4) | 13 (5.6) | 2 (5.6) | 11 (5.6) |
| **Long-term care facility residence, n (%)** |  |  |  |  |  |  |  |  |  |
| Yes | 11 (4.0) | 1 (2.4) | 10 (4.2) | 3 (6.4) | 0 (0.0) | 3 (7.1) | 8 (3.5) | 1 (2.8) | 7 (3.6) |
| **VACCINATION STATUS AT TIME OF HOSPITAL ADMISSION** | | | | | | | | | |
| Unvaccinated, n (%) | 26 (9.4) | 1 (2.4) | 25 (10.5) | 6 (12.8) | 0 (0.0) | 6 (14.3) | 20 (8.7) | 1 (2.8) | 19 (9.7) |
| Incomplete primary series, n (%) | 3 (1.1) | 0 (0.0) | 3 (1.3) | 1 (2.1) | 0 (0.0) | 1 (2.4) | 2 (0.9) | 0 (0.0) | 2 (1.0) |
| Primary series completed but no boosters, n (%) | 23 (8.3) | 7 (17.1) | 16 (6.8) | 1 (2.1) | 0 (0.0) | 1 (2.4) | 22 (9.5) | 7 (19.4) | 15 (7.7) |
| At least one booster dose, n (%) | 226 (81.3) | 33 (80.5) | 193 (81.4) | 39 (83.0) | 5 (100.0) | 34 (81.0) | 187 (81.0) | 28 (77.8) | 159 (81.5) |
| **Number of booster doses ^c^, n (%)** |  |  |  |  |  |  |  |  |  |
| 0 | 23 (9.2) | 7 (17.5) | 16 (7.7) | 1 (2.5) | 0 (0.0) | 1 (2.9) | 22 (10.5) | 7 (20.0) | 15 (8.6) |
| 1 | 80 (32.1) | 9 (22.5) | 71 (34.0) | 10 (25.0) | 2 (40.0) | 8 (22.9) | 70 (33.5) | 7 (20.0) | 63 (36.2) |
| 2 | 140 (56.2) | 22 (55.0) | 118 (56.5) | 29 (72.5) | 3 (60.0) | 26 (74.3) | 111 (53.1) | 19 (54.3) | 92 (52.9) |
| 3+ | 6 (2.4) | 2 (5.0) | 4 (1.9) | 0 (0.0) | 0 (0.0) | 0 (0.0) | 6 (2.9) | 2 (5.7) | 4 (2.3) |
| **Time since last vaccine dose (days)** |  |  |  |  |  |  |  |  |  |
| Mean (SD) | 314.4 (179.5) | 325.9 (191.8) | 312.2 (177.5) | 273.0 (156.8) | 303.4 (164.0) | 268.7 (157.7) | 322.4 (182.8) | 329.1 (197.4) | 321.1 (180.4) |
| Median  (IQR) | 213.5  (166.0, 487.0) | 221.0  (165.8, 498.2) | 211.0  (166.0, 486.2) | 195.0  (170.0, 438.0) | 217.0  (174.0, 434.0) | 191.5  (169.0, 447.2) | 216.0  (166.0, 489.0) | 225.0  (157.5, 507.5) | 215.0  (166.0, 487.5) |
| Range | 44.0 - 782.0 | 105.0 - 710.0 | 44.0 - 782.0 | 62.0 - 563.0 | 169.0 - 523.0 | 62.0 - 563.0 | 44.0 - 782.0 | 105.0 - 710.0 | 44.0 - 782.0 |
| **Time since last vaccine dose, n (%)** |  |  |  |  |  |  |  |  |  |
| <2 months | 2 (0.8) | 0 (0.0) | 2 (0.9) | 0 (0.0) | 0 (0.0) | 0 (0.0) | 2 (0.9) | 0 (0.0) | 2 (1.1) |
| [2 - 4) months | 13 (5.2) | 2 (5.0) | 11 (5.2) | 3 (7.3) | 0 (0.0) | 3 (8.3) | 10 (4.7) | 2 (5.7) | 8 (4.5) |
| [4 - 6) months | 73 (29.0) | 13 (32.5) | 60 (28.3) | 14 (34.1) | 2 (40.0) | 12 (33.3) | 59 (28.0) | 11 (31.4) | 48 (27.3) |
| [6 - 8) months | 52 (20.6) | 6 (15.0) | 46 (21.7) | 11 (26.8) | 1 (20.0) | 10 (27.8) | 41 (19.4) | 5 (14.3) | 36 (20.5) |
| ≥8 months | 112 (44.4) | 19 (47.5) | 93 (43.9) | 13 (31.7) | 2 (40.0) | 11 (30.6) | 99 (46.9) | 17 (48.6) | 82 (46.6) |
| **SYMPTOMS** | | | | | | | | | |
| **SARI symptoms ^d,e^, n (%)** |  |  |  |  |  |  |  |  |  |
| Cough | 217 (78.6) | 32 (78.0) | 185 (78.7) | 32 (69.6) | 3 (60.0) | 29 (70.7) | 185 (80.4) | 29 (80.6) | 156 (80.4) |
| Fever | 146 (52.9) | 26 (63.4) | 120 (51.1) | 22 (46.8) | 3 (60.0) | 19 (45.2) | 124 (54.1) | 23 (63.9) | 101 (52.3) |
| Shortness of breath | 217 (78.1) | 29 (70.7) | 188 (79.3) | 32 (68.1) | 3 (60.0) | 29 (69.0) | 185 (80.1) | 26 (72.2) | 159 (81.5) |
| Anosmia, ageusia or dysgeusia | 5 (1.8) | 0 (0.0) | 5 (2.1) | 1 (2.2) | 0 (0.0) | 1 (2.4) | 4 (1.8) | 0 (0.0) | 4 (2.1) |
| **Number of SARI symptoms, n (%)** |  |  |  |  |  |  |  |  |  |
| 1 | 43 (15.8) | 4 (10.0) | 39 (16.8) | 15 (32.6) | 1 (20.0) | 14 (34.1) | 28 (12.4) | 3 (8.6) | 25 (13.1) |
| 2 | 158 (58.1) | 27 (67.5) | 131 (56.5) | 23 (50.0) | 4 (80.0) | 19 (46.3) | 135 (59.7) | 23 (65.7) | 112 (58.6) |
| 3+ | 71 (26.1) | 9 (22.5) | 62 (26.7) | 8 (17.4) | 0 (0.0) | 8 (19.5) | 63 (27.9) | 9 (25.7) | 54 (28.3) |
| **HOSPITAL OUTCOMES** | | | | | | | | | |
| **Length of stay (days)** |  |  |  |  |  |  |  |  |  |
| Mean (SD) | 9.1 (10.9) | 6.3 (4.8) | 9.6 (11.6) | 9.5 (11.3) | 7.0 (8.5) | 9.8 (11.6) | 9.0 (10.9) | 6.2 (4.3) | 9.6 (11.6) |
| Median  (IQR) | 6.0  (3.0, 11.0) | 5.0  (4.0, 7.0) | 6.0  (3.0, 11.0) | 7.0  (4.0, 10.0) | 4.0  (3.0, 5.0) | 7.0  (4.2, 10.0) | 6.0  (3.0, 11.0) | 5.0  (4.0, 7.0) | 6.0  (3.0, 12.0) |
| Range | 1.0 - 101.0 | 1.0 - 22.0 | 1.0 - 101.0 | 1.0 - 70.0 | 1.0 - 22.0 | 1.0 - 70.0 | 1.0 - 101.0 | 1.0 - 21.0 | 1.0 - 101.0 |
| **Severe outcomes, n (%)** |  |  |  |  |  |  |  |  |  |
| Hospitalization without ICU admission or in-hospital death | 239 (86.0) | 34 (82.9) | 205 (86.5) | 41 (87.2) | 4 (80.0) | 37 (88.1) | 198 (85.7) | 30 (83.3) | 168 (86.2) |
| ICU admission without in-hospital death | 21 (7.6) | 4 (9.8) | 17 (7.2) | 3 (6.4) | 1 (20.0) | 2 (4.8) | 18 (7.8) | 3 (8.3) | 15 (7.7) |
| ICU admission (with or without in-hospital death) | 24 (8.6) | 4 (9.8) | 20 (8.4) | 4 (8.5) | 1 (20.0) | 3 (7.1) | 20 (8.7) | 3 (8.3) | 17 (8.7) |
| In-hospital death | 18 (6.5) | 3 (7.3) | 15 (6.3) | 3 (6.4) | 0 (0.0) | 3 (7.1) | 15 (6.5) | 3 (8.3) | 12 (6.2) |
| **COMORBIDITIES** | | | | | | | | | |
| **Number of comorbidities other than IC, n (%)** |  |  |  |  |  |  |  |  |  |
| No comorbid conditions | 33 (11.9) | 4 (9.8) | 29 (12.2) | 2 (4.3) | 0 (0.0) | 2 (4.8) | 31 (13.4) | 4 (11.1) | 27 (13.8) |
| 1 comorbidity | 58 (20.9) | 8 (19.5) | 50 (21.1) | 6 (12.8) | 0 (0.0) | 6 (14.3) | 52 (22.5) | 8 (22.2) | 44 (22.6) |
| 2 comorbidities | 62 (22.3) | 7 (17.1) | 55 (23.2) | 19 (40.4) | 2 (40.0) | 17 (40.5) | 43 (18.6) | 5 (13.9) | 38 (19.5) |
| 3+ comorbidities | 125 (45.0) | 22 (53.7) | 103 (43.5) | 20 (42.6) | 3 (60.0) | 17 (40.5) | 105 (45.5) | 19 (52.8) | 86 (44.1) |
| **Number with specific comorbidities ^d^, n (%)** |  |  |  |  |  |  |  |  |  |
| Asthma | 36 (12.9) | 6 (14.6) | 30 (12.7) | 6 (12.8) | 1 (20.0) | 5 (11.9) | 30 (13.0) | 5 (13.9) | 25 (12.8) |
| Lung disease | 121 (43.5) | 21 (51.2) | 100 (42.2) | 17 (36.2) | 0 (0.0) | 17 (40.5) | 104 (45.0) | 21 (58.3) | 83 (42.6) |
| Cardiovascular disease | 131 (47.1) | 18 (43.9) | 113 (47.7) | 25 (53.2) | 3 (60.0) | 22 (52.4) | 106 (45.9) | 15 (41.7) | 91 (46.7) |
| Hypertension | 146 (52.5) | 18 (43.9) | 128 (54.0) | 29 (61.7) | 4 (80.0) | 25 (59.5) | 117 (50.6) | 14 (38.9) | 103 (52.8) |
| Chronic liver disease | 42 (15.1) | 11 (26.8) | 31 (13.1) | 8 (17.0) | 2 (40.0) | 6 (14.3) | 34 (14.7) | 9 (25.0) | 25 (12.8) |
| Chronic kidney disease | 50 (18.0) | 15 (36.6) | 35 (14.8) | 9 (19.1) | 3 (60.0) | 6 (14.3) | 41 (17.7) | 12 (33.3) | 29 (14.9) |
| Type 2 diabetes | 66 (23.7) | 9 (22.0) | 57 (24.1) | 14 (29.8) | 2 (40.0) | 12 (28.6) | 52 (22.5) | 7 (19.4) | 45 (23.1) |
| Cancer | 81 (29.1) | 15 (36.6) | 66 (27.8) | 20 (42.6) | 3 (60.0) | 17 (40.5) | 61 (26.4) | 12 (33.3) | 49 (25.1) |
| Alternative IC ^h^ | 107 (38.5) | 41 (100.0) | 66 (27.8) | 22 (46.8) | 5 (100.0) | 17 (40.5) | 85 (36.8) | 36 (100.0) | 49 (25.1) |

^a^ Main definition of IC (Table 1); ^b^ Overall, the entire study time from 01 June 2021 to 31 May 2023, quarters based on symptom onset date; Time periods are divided according to fiscal quarters, in brackets number of study sites in each quarter; ^c^ Among the patients eligible to receive booster (completed primary series); ^d^ Not mutually exclusive; ^e^ The recording of individual SARI symptoms became mandatory after COVIDRIVE master protocol v4.0 (October 10, 2022), therefore records of SARI patients admitted to hospital before this date may lack that information; ^f^ Main SARI definition (Table 1); ^g^ Main SARS-CoV-2 test status definition (Table 1); ^h^ Alternative IC definition – main IC and cancer (Table 1). Abbreviations: CI, confidence interval; CIRI-IT, Centro Interuniversitario di Ricerca sull'Influenza e le altre Infezioni Trasmissibili; GTPUH, Hospital Universitario Germans Trias i Pujol; HUVH, Hospital Universitari Vall d’Hebron; IC, immunocompromising condition; ICU, intensive care unit; IQR, interquartile range; n, number; Q, calendar time in fiscal quarter; SARI, severe acute respiratory infection; SARS-CoV-2, severe acute respiratory syndrome coronavirus 2; SD, standard deviation; UZA, Universitair Ziekenhuis Antwerpen. A square bracket “[“ and “]” in a range denotes that the number is included in the range, while regular brackets “(“ and “)” in a range means that the number is not included.

Table S9. Demographic and clinical characteristics of hospitalized SARI patients in three European countries, by SARS-CoV-2 test status, by IC status (alternative definition), and by calendar time

| **Covariates per period ^b^** | **SARI ^f^** | | | **SARS-CoV-2 test status ^g^** | | | | | |
| --- | --- | --- | --- | --- | --- | --- | --- | --- | --- |
|  |  |  |  | **Positive** | | | **Negative** | | |
|  | **Overall (N=5,280)** | **IC ^a^ (N=1,521)** | **Non-IC  (N=3,759)** | **Overall (N=1,924)** | **IC ^a^  (N=570)** | **Non-IC  (N=1,354)** | **Overall (N=3,356)** | **IC ^a^  (N=951)** | **Non-IC (N=2,405)** |
| **Q2 2021** |  |  |  |  |  |  |  |  |  |
| **Total** | **96** | **18** | **78** | **29** | **1** | **28** | **67** | **17** | **50** |
| **DEMOGRAPHICS** | | | | | | | | | |
| **Age (years)** |  |  |  |  |  |  |  |  |  |
| Mean (SD) | 64.2 (18.8) | 68.4 (16.7) | 63.3 (19.2) | 49.9 (16.2) | 54.0 (NA) | 49.8 (16.5) | 70.4 (16.4) | 69.2 (16.8) | 70.9 (16.4) |
| Median  (IQR) | 68.5  (48.5, 80.0) | 70.5  (56.0, 81.5) | 68.0  (47.0, 78.5) | 47.0  (41.0, 61.0) | 54.0  (54.0, 54.0) | 47.0  (40.8, 61.2) | 74.0  (64.0, 82.0) | 72.0  (62.0, 82.0) | 74.0  (64.2, 82.0) |
| Range | 22.0 - 97.0 | 34.0 - 92.0 | 22.0 - 97.0 | 22.0 - 89.0 | 54.0 - 54.0 | 22.0 - 89.0 | 27.0 - 97.0 | 34.0 - 92.0 | 27.0 - 97.0 |
| **Sex, n (%)** |  |  |  |  |  |  |  |  |  |
| Male | 58 (60.4) | 15 (83.3) | 43 (55.1) | 18 (62.1) | 1 (100.0) | 17 (60.7) | 40 (59.7) | 14 (82.4) | 26 (52.0) |
| Female | 38 (39.6) | 3 (16.7) | 35 (44.9) | 11 (37.9) | 0 (0.0) | 11 (39.3) | 27 (40.3) | 3 (17.6) | 24 (48.0) |
| **Country, n (%)** |  |  |  |  |  |  |  |  |  |
| Belgium | 16 (16.7) | 0 (0.0) | 16 (20.5) | 7 (24.1) | 0 (0.0) | 7 (25.0) | 9 (13.4) | 0 (0.0) | 9 (18.0) |
| Italy | 0 (0.0) | 0 (0.0) | 0 (0.0) | 0 (0.0) | 0 (0.0) | 0 (0.0) | 0 (0.0) | 0 (0.0) | 0 (0.0) |
| Spain | 80 (83.3) | 18 (100.0) | 62 (79.5) | 22 (75.9) | 1 (100.0) | 21 (75.0) | 58 (86.6) | 17 (100.0) | 41 (82.0) |
| **Study site, n (%)** |  |  |  |  |  |  |  |  |  |
| CIRI-IT | 0 (0.0) | 0 (0.0) | 0 (0.0) | 0 (0.0) | 0 (0.0) | 0 (0.0) | 0 (0.0) | 0 (0.0) | 0 (0.0) |
| GTPUH | 80 (83.3) | 18 (100.0) | 62 (79.5) | 22 (75.9) | 1 (100.0) | 21 (75.0) | 58 (86.6) | 17 (100.0) | 41 (82.0) |
| CHU Saint-Pierre | 3 (3.1) | 0 (0.0) | 3 (3.8) | 1 (3.4) | 0 (0.0) | 1 (3.6) | 2 (3.0) | 0 (0.0) | 2 (4.0) |
| UZA | 13 (13.5) | 0 (0.0) | 13 (16.7) | 6 (20.7) | 0 (0.0) | 6 (21.4) | 7 (10.4) | 0 (0.0) | 7 (14.0) |
| HUVH | 0 (0.0) | 0 (0.0) | 0 (0.0) | 0 (0.0) | 0 (0.0) | 0 (0.0) | 0 (0.0) | 0 (0.0) | 0 (0.0) |
| **Smoking, n (%)** |  |  |  |  |  |  |  |  |  |
| Never smoker | 41 (42.7) | 5 (27.8) | 36 (46.2) | 14 (48.3) | 0 (0.0) | 14 (50.0) | 27 (40.3) | 5 (29.4) | 22 (44.0) |
| Former smoker | 24 (25.0) | 10 (55.6) | 14 (17.9) | 5 (17.2) | 1 (100.0) | 4 (14.3) | 19 (28.4) | 9 (52.9) | 10 (20.0) |
| Current smoker | 20 (20.8) | 3 (16.7) | 17 (21.8) | 3 (10.3) | 0 (0.0) | 3 (10.7) | 17 (25.4) | 3 (17.6) | 14 (28.0) |
| Missing | 11 (11.5) | 0 (0.0) | 11 (14.1) | 7 (24.1) | 0 (0.0) | 7 (25.0) | 4 (6.0) | 0 (0.0) | 4 (8.0) |
| **Long-term care facility residence, n (%)** |  |  |  |  |  |  |  |  |  |
| Yes | 4 (4.3) | 1 (5.6) | 3 (3.9) | 0 (0.0) | 0 (0.0) | 0 (0.0) | 4 (6.2) | 1 (5.9) | 3 (6.2) |
| **VACCINATION STATUS AT TIME OF HOSPITAL ADMISSION** | | | | | | | | | |
| Unvaccinated, n (%) | 31 (32.3) | 2 (11.1) | 29 (37.2) | 18 (62.1) | 0 (0.0) | 18 (64.3) | 13 (19.4) | 2 (11.8) | 11 (22.0) |
| Incomplete primary series, n (%) | 20 (20.8) | 5 (27.8) | 15 (19.2) | 6 (20.7) | 1 (100.0) | 5 (17.9) | 14 (20.9) | 4 (23.5) | 10 (20.0) |
| Primary series completed but no boosters, n (%) | 45 (46.9) | 11 (61.1) | 34 (43.6) | 5 (17.2) | 0 (0.0) | 5 (17.9) | 40 (59.7) | 11 (64.7) | 29 (58.0) |
| At least one booster dose, n (%) | 0 (0.0) | 0 (0.0) | 0 (0.0) | 0 (0.0) | 0 (0.0) | 0 (0.0) | 0 (0.0) | 0 (0.0) | 0 (0.0) |
| **Number of booster doses ^c^, n (%)** |  |  |  |  |  |  |  |  |  |
| 0 | 45 (100.0) | 11 (100.0) | 34 (100.0) | 5 (100.0) | 0 | 5 (100.0) | 40 (100.0) | 11 (100.0) | 29 (100.0) |
| 1 | 0 (0.0) | 0 (0.0) | 0 (0.0) | 0 (0.0) | 0 | 0 (0.0) | 0 (0.0) | 0 (0.0) | 0 (0.0) |
| 2 | 0 (0.0) | 0 (0.0) | 0 (0.0) | 0 (0.0) | 0 | 0 (0.0) | 0 (0.0) | 0 (0.0) | 0 (0.0) |
| 3+ | 0 (0.0) | 0 (0.0) | 0 (0.0) | 0 (0.0) | 0 | 0 (0.0) | 0 (0.0) | 0 (0.0) | 0 (0.0) |
| **Time since last vaccine dose (days)** |  |  |  |  |  |  |  |  |  |
| Mean (SD) | 43.9 (28.8) | 40.7 (22.1) | 45.0 (30.8) | 31.0 (29.8) | 23.0 (NA) | 31.8 (31.3) | 46.6 (28.1) | 41.9 (22.4) | 48.4 (30.1) |
| Median  (IQR) | 39.0  (19.0, 67.0) | 31.0  (24.5, 53.8) | 42.0  (17.0, 69.0) | 15.0  (12.0, 37.5) | 23.0  (23.0, 23.0) | 14.5  (11.5, 43.8) | 42.0  (26.0, 68.5) | 31.0  (25.5, 57.5) | 43.0  (27.5, 69.5) |
| Range | 1.0 - 130.0 | 18.0 - 85.0 | 1.0 - 130.0 | 9.0 - 98.0 | 23.0 - 23.0 | 9.0 - 98.0 | 1.0 - 130.0 | 18.0 - 85.0 | 1.0 - 130.0 |
| **Time since last vaccine dose, n (%)** |  |  |  |  |  |  |  |  |  |
| <2 months | 45 (69.2) | 12 (75.0) | 33 (67.3) | 9 (81.8) | 1 (100.0) | 8 (80.0) | 36 (66.7) | 11 (73.3) | 25 (64.1) |
| [2 - 4) months | 18 (27.7) | 4 (25.0) | 14 (28.6) | 2 (18.2) | 0 (0.0) | 2 (20.0) | 16 (29.6) | 4 (26.7) | 12 (30.8) |
| [4 - 6) months | 2 (3.1) | 0 (0.0) | 2 (4.1) | 0 (0.0) | 0 (0.0) | 0 (0.0) | 2 (3.7) | 0 (0.0) | 2 (5.1) |
| [6 - 8) months | 0 (0.0) | 0 (0.0) | 0 (0.0) | 0 (0.0) | 0 (0.0) | 0 (0.0) | 0 (0.0) | 0 (0.0) | 0 (0.0) |
| ≥8 months | 0 (0.0) | 0 (0.0) | 0 (0.0) | 0 (0.0) | 0 (0.0) | 0 (0.0) | 0 (0.0) | 0 (0.0) | 0 (0.0) |
| **SYMPTOMS** | | | | | | | | | |
| **SARI symptoms ^d,e^, n (%)** |  |  |  |  |  |  |  |  |  |
| Cough | 46 (48.4) | 5 (27.8) | 41 (53.2) | 20 (69.0) | 0 (0.0) | 20 (71.4) | 26 (39.4) | 5 (29.4) | 21 (42.9) |
| Fever | 77 (81.1) | 17 (94.4) | 60 (77.9) | 20 (71.4) | 1 (100.0) | 19 (70.4) | 57 (85.1) | 16 (94.1) | 41 (82.0) |
| Shortness of breath | 69 (71.9) | 14 (77.8) | 55 (70.5) | 20 (69.0) | 1 (100.0) | 19 (67.9) | 49 (73.1) | 13 (76.5) | 36 (72.0) |
| Anosmia, ageusia or dysgeusia | 6 (6.7) | 0 (0.0) | 6 (8.5) | 6 (22.2) | 0 (0.0) | 6 (23.1) | 0 (0.0) | 0 (0.0) | 0 (0.0) |
| **Number of SARI symptoms, n (%)** |  |  |  |  |  |  |  |  |  |
| 1 | 8 (9.1) | 2 (11.1) | 6 (8.6) | 1 (3.8) | 0 (0.0) | 1 (4.0) | 7 (11.3) | 2 (11.8) | 5 (11.1) |
| 2 | 64 (72.7) | 14 (77.8) | 50 (71.4) | 16 (61.5) | 1 (100.0) | 15 (60.0) | 48 (77.4) | 13 (76.5) | 35 (77.8) |
| 3+ | 16 (18.2) | 2 (11.1) | 14 (20.0) | 9 (34.6) | 0 (0.0) | 9 (36.0) | 7 (11.3) | 2 (11.8) | 5 (11.1) |
| **HOSPITAL OUTCOMES** | | | | | | | | | |
| **Length of stay (days)** |  |  |  |  |  |  |  |  |  |
| Mean (SD) | 9.4 (7.4) | 10.8 (6.5) | 9.1 (7.6) | 7.0 (4.2) | 6.0 (NA) | 7.1 (4.3) | 10.4 (8.3) | 11.1 (6.6) | 10.2 (8.8) |
| Median  (IQR) | 8.0  (4.8, 11.0) | 10.0  (6.0, 11.0) | 7.5  (4.0, 11.0) | 6.0  (4.0, 9.0) | 6.0  (6.0, 6.0) | 6.0  (4.0, 9.0) | 8.0  (5.0, 12.5) | 10.0  (6.0, 11.0) | 8.0  (4.2, 12.8) |
| Range | 1.0 - 50.0 | 3.0 - 25.0 | 1.0 - 50.0 | 2.0 - 18.0 | 6.0 - 6.0 | 2.0 - 18.0 | 1.0 - 50.0 | 3.0 - 25.0 | 1.0 - 50.0 |
| **Severe outcomes, n (%)** |  |  |  |  |  |  |  |  |  |
| Hospitalization without ICU admission or in-hospital death | 79 (82.3) | 15 (83.3) | 64 (82.1) | 21 (72.4) | 1 (100.0) | 20 (71.4) | 58 (86.6) | 14 (82.4) | 44 (88.0) |
| ICU admission without in-hospital death | 9 (9.4) | 0 (0.0) | 9 (11.5) | 7 (24.1) | 0 (0.0) | 7 (25.0) | 2 (3.0) | 0 (0.0) | 2 (4.0) |
| ICU admission (with or without in-hospital death) | 10 (10.4) | 0 (0.0) | 10 (12.8) | 8 (27.6) | 0 (0.0) | 8 (28.6) | 2 (3.0) | 0 (0.0) | 2 (4.0) |
| In-hospital death | 8 (8.3) | 3 (16.7) | 5 (6.4) | 1 (3.4) | 0 (0.0) | 1 (3.6) | 7 (10.4) | 3 (17.6) | 4 (8.0) |
| **COMORBIDITIES** | | | | | | | | | |
| **Number of comorbidities other than IC, n (%)** |  |  |  |  |  |  |  |  |  |
| No comorbid conditions | 23 (24.0) | 1 (5.6) | 22 (28.2) | 17 (58.6) | 0 (0.0) | 17 (60.7) | 6 (9.0) | 1 (5.9) | 5 (10.0) |
| 1 comorbidity | 17 (17.7) | 3 (16.7) | 14 (17.9) | 4 (13.8) | 0 (0.0) | 4 (14.3) | 13 (19.4) | 3 (17.6) | 10 (20.0) |
| 2 comorbidities | 21 (21.9) | 5 (27.8) | 16 (20.5) | 3 (10.3) | 1 (100.0) | 2 (7.1) | 18 (26.9) | 4 (23.5) | 14 (28.0) |
| 3+ comorbidities | 35 (36.5) | 9 (50.0) | 26 (33.3) | 5 (17.2) | 0 (0.0) | 5 (17.9) | 30 (44.8) | 9 (52.9) | 21 (42.0) |
| **Number with specific comorbidities ^d^, n (%)** |  |  |  |  |  |  |  |  |  |
| Asthma | 7 (7.3) | 1 (5.6) | 6 (7.7) | 0 (0.0) | 0 (0.0) | 0 (0.0) | 7 (10.4) | 1 (5.9) | 6 (12.0) |
| Lung disease | 22 (22.9) | 8 (44.4) | 14 (17.9) | 2 (6.9) | 0 (0.0) | 2 (7.1) | 20 (29.9) | 8 (47.1) | 12 (24.0) |
| Cardiovascular disease | 37 (38.5) | 8 (44.4) | 29 (37.2) | 5 (17.2) | 1 (100.0) | 4 (14.3) | 32 (47.8) | 7 (41.2) | 25 (50.0) |
| Hypertension | 53 (55.2) | 12 (66.7) | 41 (52.6) | 10 (34.5) | 1 (100.0) | 9 (32.1) | 43 (64.2) | 11 (64.7) | 32 (64.0) |
| Chronic liver disease | 9 (9.4) | 0 (0.0) | 9 (11.5) | 1 (3.4) | 0 (0.0) | 1 (3.6) | 8 (11.9) | 0 (0.0) | 8 (16.0) |
| Chronic kidney disease | 18 (18.8) | 4 (22.2) | 14 (17.9) | 4 (13.8) | 0 (0.0) | 4 (14.3) | 14 (20.9) | 4 (23.5) | 10 (20.0) |
| Type 2 diabetes | 27 (28.1) | 5 (27.8) | 22 (28.2) | 5 (17.2) | 0 (0.0) | 5 (17.9) | 22 (32.8) | 5 (29.4) | 17 (34.0) |
| Cancer | 15 (15.6) | 15 (83.3) | 0 (0.0) | 0 (0.0) | 0 (0.0) | 0 (0.0) | 15 (22.4) | 15 (88.2) | 0 (0.0) |
| Main IC ^h^ | 8 (8.3) | 8 (44.4) | 0 (0.0) | 1 (3.4) | 1 (100.0) | 0 (0.0) | 7 (10.4) | 7 (41.2) | 0 (0.0) |
| **Q3 2021** |  |  |  |  |  |  |  |  |  |
| **Total** | **378** | **88** | **290** | **186** | **28** | **158** | **192** | **60** | **132** |
| **DEMOGRAPHICS** | | | | | | | | | |
| **Age (years)** |  |  |  |  |  |  |  |  |  |
| Mean (SD) | 64.5 (18.4) | 66.3 (14.6) | 64.0 (19.3) | 58.8 (19.2) | 59.7 (17.6) | 58.6 (19.6) | 70.1 (15.6) | 69.4 (12.0) | 70.3 (17.0) |
| Median  (IQR) | 69.0  (50.0, 79.0) | 69.0  (58.0, 77.0) | 67.5  (49.0, 80.0) | 57.0  (44.0, 77.0) | 61.0  (48.0, 76.0) | 57.0  (43.0, 77.8) | 73.0  (62.0, 80.0) | 73.0  (61.8, 77.0) | 73.0  (63.0, 81.2) |
| Range | 18.0 - 99.0 | 22.0 - 99.0 | 18.0 - 98.0 | 18.0 - 95.0 | 22.0 - 87.0 | 18.0 - 95.0 | 19.0 - 99.0 | 31.0 - 99.0 | 19.0 - 98.0 |
| **Sex, n (%)** |  |  |  |  |  |  |  |  |  |
| Male | 224 (59.3) | 54 (61.4) | 170 (58.6) | 102 (54.8) | 14 (50.0) | 88 (55.7) | 122 (63.5) | 40 (66.7) | 82 (62.1) |
| Female | 154 (40.7) | 34 (38.6) | 120 (41.4) | 84 (45.2) | 14 (50.0) | 70 (44.3) | 70 (36.5) | 20 (33.3) | 50 (37.9) |
| **Country, n (%)** |  |  |  |  |  |  |  |  |  |
| Belgium | 100 (26.5) | 19 (21.6) | 81 (27.9) | 70 (37.6) | 14 (50.0) | 56 (35.4) | 30 (15.6) | 5 (8.3) | 25 (18.9) |
| Italy | 0 (0.0) | 0 (0.0) | 0 (0.0) | 0 (0.0) | 0 (0.0) | 0 (0.0) | 0 (0.0) | 0 (0.0) | 0 (0.0) |
| Spain | 278 (73.5) | 69 (78.4) | 209 (72.1) | 116 (62.4) | 14 (50.0) | 102 (64.6) | 162 (84.4) | 55 (91.7) | 107 (81.1) |
| **Study site, n (%)** |  |  |  |  |  |  |  |  |  |
| CIRI-IT | 0 (0.0) | 0 (0.0) | 0 (0.0) | 0 (0.0) | 0 (0.0) | 0 (0.0) | 0 (0.0) | 0 (0.0) | 0 (0.0) |
| GTPUH | 278 (73.5) | 69 (78.4) | 209 (72.1) | 116 (62.4) | 14 (50.0) | 102 (64.6) | 162 (84.4) | 55 (91.7) | 107 (81.1) |
| CHU Saint-Pierre | 26 (6.9) | 5 (5.7) | 21 (7.2) | 13 (7.0) | 3 (10.7) | 10 (6.3) | 13 (6.8) | 2 (3.3) | 11 (8.3) |
| UZA | 74 (19.6) | 14 (15.9) | 60 (20.7) | 57 (30.6) | 11 (39.3) | 46 (29.1) | 17 (8.9) | 3 (5.0) | 14 (10.6) |
| HUVH | 0 (0.0) | 0 (0.0) | 0 (0.0) | 0 (0.0) | 0 (0.0) | 0 (0.0) | 0 (0.0) | 0 (0.0) | 0 (0.0) |
| **Smoking, n (%)** |  |  |  |  |  |  |  |  |  |
| Never smoker | 129 (34.1) | 21 (23.9) | 108 (37.2) | 75 (40.3) | 4 (14.3) | 71 (44.9) | 54 (28.1) | 17 (28.3) | 37 (28.0) |
| Former smoker | 83 (22.0) | 33 (37.5) | 50 (17.2) | 18 (9.7) | 5 (17.9) | 13 (8.2) | 65 (33.9) | 28 (46.7) | 37 (28.0) |
| Current smoker | 52 (13.8) | 12 (13.6) | 40 (13.8) | 12 (6.5) | 3 (10.7) | 9 (5.7) | 40 (20.8) | 9 (15.0) | 31 (23.5) |
| Missing | 114 (30.2) | 22 (25.0) | 92 (31.7) | 81 (43.5) | 16 (57.1) | 65 (41.1) | 33 (17.2) | 6 (10.0) | 27 (20.5) |
| **Long-term care facility residence, n (%)** |  |  |  |  |  |  |  |  |  |
| Yes | 12 (3.3) | 4 (4.9) | 8 (2.8) | 4 (2.2) | 1 (4.0) | 3 (1.9) | 8 (4.3) | 3 (5.3) | 5 (3.9) |
| **VACCINATION STATUS AT TIME OF HOSPITAL ADMISSION** | | | | | | | | | |
| Unvaccinated, n (%) | 111 (29.4) | 13 (14.8) | 98 (33.8) | 90 (48.4) | 8 (28.6) | 82 (51.9) | 21 (10.9) | 5 (8.3) | 16 (12.1) |
| Incomplete primary series, n (%) | 23 (6.1) | 4 (4.5) | 19 (6.6) | 14 (7.5) | 2 (7.1) | 12 (7.6) | 9 (4.7) | 2 (3.3) | 7 (5.3) |
| Primary series completed but no boosters, n (%) | 244 (64.6) | 71 (80.7) | 173 (59.7) | 82 (44.1) | 18 (64.3) | 64 (40.5) | 162 (84.4) | 53 (88.3) | 109 (82.6) |
| At least one booster dose, n (%) | 0 (0.0) | 0 (0.0) | 0 (0.0) | 0 (0.0) | 0 (0.0) | 0 (0.0) | 0 (0.0) | 0 (0.0) | 0 (0.0) |
| **Number of booster doses ^c^, n (%)** |  |  |  |  |  |  |  |  |  |
| 0 | 244 (100.0) | 71 (100.0) | 173 (100.0) | 82 (100.0) | 18 (100.0) | 64 (100.0) | 162 (100.0) | 53 (100.0) | 109 (100.0) |
| 1 | 0 (0.0) | 0 (0.0) | 0 (0.0) | 0 (0.0) | 0 (0.0) | 0 (0.0) | 0 (0.0) | 0 (0.0) | 0 (0.0) |
| 2 | 0 (0.0) | 0 (0.0) | 0 (0.0) | 0 (0.0) | 0 (0.0) | 0 (0.0) | 0 (0.0) | 0 (0.0) | 0 (0.0) |
| 3+ | 0 (0.0) | 0 (0.0) | 0 (0.0) | 0 (0.0) | 0 (0.0) | 0 (0.0) | 0 (0.0) | 0 (0.0) | 0 (0.0) |
| **Time since last vaccine dose (days)** |  |  |  |  |  |  |  |  |  |
| Mean (SD) | 87.5 (47.6) | 90.8 (44.0) | 86.3 (48.9) | 74.4 (47.9) | 84.7 (41.4) | 71.7 (49.4) | 94.9 (45.9) | 93.0 (45.1) | 95.8 (46.5) |
| Median  (IQR) | 87.0  (51.5, 123.0) | 90.0  (60.5, 122.5) | 87.0  (49.5, 123.2) | 77.0  (29.2, 112.0) | 79.0  (61.8, 113.8) | 75.5  (22.5, 107.8) | 91.0  (64.0, 129.0) | 90.0  (60.5, 125.0) | 92.5  (66.0, 130.2) |
| Range | 1.0 - 216.0 | 10.0 - 216.0 | 1.0 - 195.0 | 1.0 - 173.0 | 14.0 - 156.0 | 1.0 - 173.0 | 1.0 - 216.0 | 10.0 - 216.0 | 1.0 - 195.0 |
| **Time since last vaccine dose, n (%)** |  |  |  |  |  |  |  |  |  |
| <2 months | 75 (28.1) | 19 (25.3) | 56 (29.2) | 38 (39.6) | 5 (25.0) | 33 (43.4) | 37 (21.6) | 14 (25.5) | 23 (19.8) |
| [2 - 4) months | 119 (44.6) | 35 (46.7) | 84 (43.8) | 39 (40.6) | 11 (55.0) | 28 (36.8) | 80 (46.8) | 24 (43.6) | 56 (48.3) |
| [4 - 6) months | 71 (26.6) | 20 (26.7) | 51 (26.6) | 19 (19.8) | 4 (20.0) | 15 (19.7) | 52 (30.4) | 16 (29.1) | 36 (31.0) |
| [6 - 8) months | 2 (0.7) | 1 (1.3) | 1 (0.5) | 0 (0.0) | 0 (0.0) | 0 (0.0) | 2 (1.2) | 1 (1.8) | 1 (0.9) |
| ≥8 months | 0 (0.0) | 0 (0.0) | 0 (0.0) | 0 (0.0) | 0 (0.0) | 0 (0.0) | 0 (0.0) | 0 (0.0) | 0 (0.0) |
| **SYMPTOMS** | | | | | | | | | |
| **SARI symptoms ^d,e^, n (%)** |  |  |  |  |  |  |  |  |  |
| Cough | 231 (66.0) | 51 (62.2) | 180 (67.2) | 131 (80.4) | 18 (78.3) | 113 (80.7) | 100 (53.5) | 33 (55.9) | 67 (52.3) |
| Fever | 172 (49.0) | 39 (47.6) | 133 (49.4) | 94 (58.0) | 16 (69.6) | 78 (56.1) | 78 (41.3) | 23 (39.0) | 55 (42.3) |
| Shortness of breath | 266 (73.7) | 63 (74.1) | 203 (73.6) | 127 (75.1) | 19 (76.0) | 108 (75.0) | 139 (72.4) | 44 (73.3) | 95 (72.0) |
| Anosmia, ageusia or dysgeusia | 16 (5.1) | 4 (5.3) | 12 (5.0) | 14 (9.9) | 3 (16.7) | 11 (8.9) | 2 (1.1) | 1 (1.7) | 1 (0.9) |
| **Number of SARI symptoms, n (%)** |  |  |  |  |  |  |  |  |  |
| 1 | 106 (34.2) | 28 (37.8) | 78 (33.1) | 37 (26.8) | 4 (22.2) | 33 (27.5) | 69 (40.1) | 24 (42.9) | 45 (38.8) |
| 2 | 140 (45.2) | 33 (44.6) | 107 (45.3) | 51 (37.0) | 7 (38.9) | 44 (36.7) | 89 (51.7) | 26 (46.4) | 63 (54.3) |
| 3+ | 64 (20.6) | 13 (17.6) | 51 (21.6) | 50 (36.2) | 7 (38.9) | 43 (35.8) | 14 (8.1) | 6 (10.7) | 8 (6.9) |
| **HOSPITAL OUTCOMES** | | | | | | | | | |
| **Length of stay (days)** |  |  |  |  |  |  |  |  |  |
| Mean (SD) | 11.4 (13.5) | 11.2 (17.0) | 11.5 (12.3) | 13.4 (16.9) | 17.5 (27.7) | 12.7 (14.1) | 9.5 (8.8) | 8.2 (7.0) | 10.1 (9.4) |
| Median  (IQR) | 7.0  (4.0, 14.0) | 6.0  (4.0, 11.2) | 7.5  (4.0, 14.0) | 8.0  (4.0, 15.0) | 7.5  (5.8, 13.2) | 8.0  (4.0, 15.0) | 7.0  (4.0, 13.0) | 5.5  (3.0, 11.0) | 7.0  (4.0, 13.2) |
| Range | 1.0 - 122.0 | 1.0 - 122.0 | 1.0 - 80.0 | 1.0 - 122.0 | 1.0 - 122.0 | 1.0 - 80.0 | 1.0 - 76.0 | 1.0 - 30.0 | 1.0 - 76.0 |
| **Severe outcomes, n (%)** |  |  |  |  |  |  |  |  |  |
| Hospitalization without ICU admission or in-hospital death | 268 (70.9) | 63 (71.6) | 205 (70.7) | 115 (61.8) | 18 (64.3) | 97 (61.4) | 153 (79.7) | 45 (75.0) | 108 (81.8) |
| ICU admission without in-hospital death | 54 (14.3) | 6 (6.8) | 48 (16.6) | 46 (24.7) | 4 (14.3) | 42 (26.6) | 8 (4.2) | 2 (3.3) | 6 (4.5) |
| ICU admission (with or without in-hospital death) | 66 (17.5) | 10 (11.4) | 56 (19.3) | 58 (31.2) | 8 (28.6) | 50 (31.6) | 8 (4.2) | 2 (3.3) | 6 (4.5) |
| In-hospital death | 56 (14.8) | 19 (21.6) | 37 (12.8) | 25 (13.4) | 6 (21.4) | 19 (12.0) | 31 (16.1) | 13 (21.7) | 18 (13.6) |
| **COMORBIDITIES** | | | | | | | | | |
| **Number of comorbidities other than IC, n (%)** |  |  |  |  |  |  |  |  |  |
| No comorbid conditions | 96 (25.4) | 5 (5.7) | 91 (31.4) | 74 (39.8) | 4 (14.3) | 70 (44.3) | 22 (11.5) | 1 (1.7) | 21 (15.9) |
| 1 comorbidity | 82 (21.7) | 23 (26.1) | 59 (20.3) | 37 (19.9) | 9 (32.1) | 28 (17.7) | 45 (23.4) | 14 (23.3) | 31 (23.5) |
| 2 comorbidities | 81 (21.4) | 21 (23.9) | 60 (20.7) | 43 (23.1) | 7 (25.0) | 36 (22.8) | 38 (19.8) | 14 (23.3) | 24 (18.2) |
| 3+ comorbidities | 119 (31.5) | 39 (44.3) | 80 (27.6) | 32 (17.2) | 8 (28.6) | 24 (15.2) | 87 (45.3) | 31 (51.7) | 56 (42.4) |
| **Number with specific comorbidities ^d^, n (%)** |  |  |  |  |  |  |  |  |  |
| Asthma | 28 (7.4) | 4 (4.5) | 24 (8.3) | 13 (7.0) | 2 (7.1) | 11 (7.0) | 15 (7.8) | 2 (3.3) | 13 (9.8) |
| Lung disease | 111 (29.4) | 30 (34.1) | 81 (27.9) | 33 (17.7) | 5 (17.9) | 28 (17.7) | 78 (40.6) | 25 (41.7) | 53 (40.2) |
| Cardiovascular disease | 114 (30.2) | 19 (21.6) | 95 (32.8) | 37 (19.9) | 3 (10.7) | 34 (21.5) | 77 (40.1) | 16 (26.7) | 61 (46.2) |
| Hypertension | 155 (41.0) | 34 (38.6) | 121 (41.7) | 64 (34.4) | 8 (28.6) | 56 (35.4) | 91 (47.4) | 26 (43.3) | 65 (49.2) |
| Chronic liver disease | 19 (5.0) | 6 (6.8) | 13 (4.5) | 9 (4.8) | 3 (10.7) | 6 (3.8) | 10 (5.2) | 3 (5.0) | 7 (5.3) |
| Chronic kidney disease | 72 (19.0) | 17 (19.3) | 55 (19.0) | 27 (14.5) | 7 (25.0) | 20 (12.7) | 45 (23.4) | 10 (16.7) | 35 (26.5) |
| Type 2 diabetes | 99 (26.2) | 24 (27.3) | 75 (25.9) | 43 (23.1) | 8 (28.6) | 35 (22.2) | 56 (29.2) | 16 (26.7) | 40 (30.3) |
| Cancer | 72 (19.0) | 72 (81.8) | 0 (0.0) | 17 (9.1) | 17 (60.7) | 0 (0.0) | 55 (28.6) | 55 (91.7) | 0 (0.0) |
| Main IC ^h^ | 52 (13.8) | 52 (59.1) | 0 (0.0) | 18 (9.7) | 18 (64.3) | 0 (0.0) | 34 (17.7) | 34 (56.7) | 0 (0.0) |
| **Q4 2021** |  |  |  |  |  |  |  |  |  |
| **Total** | **1,017** | **258** | **759** | **391** | **85** | **306** | **626** | **173** | **453** |
| **DEMOGRAPHICS** | | | | | | | | | |
| **Age (years)** |  |  |  |  |  |  |  |  |  |
| Mean (SD) | 66.9 (16.4) | 68.5 (14.4) | 66.3 (17.0) | 62.7 (15.3) | 65.1 (14.5) | 62.1 (15.5) | 69.5 (16.6) | 70.2 (14.1) | 69.2 (17.4) |
| Median  (IQR) | 68.0  (57.0, 79.0) | 71.0  (61.0, 78.0) | 67.0  (55.0, 79.0) | 65.0  (53.0, 73.5) | 68.0  (56.0, 76.0) | 63.0  (51.0, 73.0) | 72.0  (59.0, 83.0) | 72.0  (63.0, 79.0) | 72.0  (58.0, 83.0) |
| Range | 18.0 - 103.0 | 23.0 - 94.0 | 18.0 - 103.0 | 27.0 - 95.0 | 27.0 - 90.0 | 27.0 - 95.0 | 18.0 - 103.0 | 23.0 - 94.0 | 18.0 - 103.0 |
| **Sex, n (%)** |  |  |  |  |  |  |  |  |  |
| Male | 581 (57.1) | 150 (58.1) | 431 (56.8) | 229 (58.6) | 42 (49.4) | 187 (61.1) | 352 (56.2) | 108 (62.4) | 244 (53.9) |
| Female | 436 (42.9) | 108 (41.9) | 328 (43.2) | 162 (41.4) | 43 (50.6) | 119 (38.9) | 274 (43.8) | 65 (37.6) | 209 (46.1) |
| **Country, n (%)** |  |  |  |  |  |  |  |  |  |
| Belgium | 162 (15.9) | 42 (16.3) | 120 (15.8) | 121 (30.9) | 34 (40.0) | 87 (28.4) | 41 (6.5) | 8 (4.6) | 33 (7.3) |
| Italy | 87 (8.6) | 24 (9.3) | 63 (8.3) | 57 (14.6) | 11 (12.9) | 46 (15.0) | 30 (4.8) | 13 (7.5) | 17 (3.8) |
| Spain | 768 (75.5) | 192 (74.4) | 576 (75.9) | 213 (54.5) | 40 (47.1) | 173 (56.5) | 555 (88.7) | 152 (87.9) | 403 (89.0) |
| **Study site, n (%)** |  |  |  |  |  |  |  |  |  |
| CIRI-IT | 87 (8.6) | 24 (9.3) | 63 (8.3) | 57 (14.6) | 11 (12.9) | 46 (15.0) | 30 (4.8) | 13 (7.5) | 17 (3.8) |
| GTPUH | 550 (54.1) | 142 (55.0) | 408 (53.8) | 120 (30.7) | 25 (29.4) | 95 (31.0) | 430 (68.7) | 117 (67.6) | 313 (69.1) |
| CHU Saint-Pierre | 28 (2.8) | 5 (1.9) | 23 (3.0) | 12 (3.1) | 2 (2.4) | 10 (3.3) | 16 (2.6) | 3 (1.7) | 13 (2.9) |
| UZA | 134 (13.2) | 37 (14.3) | 97 (12.8) | 109 (27.9) | 32 (37.6) | 77 (25.2) | 25 (4.0) | 5 (2.9) | 20 (4.4) |
| HUVH | 218 (21.4) | 50 (19.4) | 168 (22.1) | 93 (23.8) | 15 (17.6) | 78 (25.5) | 125 (20.0) | 35 (20.2) | 90 (19.9) |
| **Smoking, n (%)** |  |  |  |  |  |  |  |  |  |
| Never smoker | 431 (42.4) | 86 (33.3) | 345 (45.5) | 151 (38.6) | 24 (28.2) | 127 (41.5) | 280 (44.7) | 62 (35.8) | 218 (48.1) |
| Former smoker | 245 (24.1) | 84 (32.6) | 161 (21.2) | 81 (20.7) | 18 (21.2) | 63 (20.6) | 164 (26.2) | 66 (38.2) | 98 (21.6) |
| Current smoker | 114 (11.2) | 27 (10.5) | 87 (11.5) | 23 (5.9) | 6 (7.1) | 17 (5.6) | 91 (14.5) | 21 (12.1) | 70 (15.5) |
| Missing | 227 (22.3) | 61 (23.6) | 166 (21.9) | 136 (34.8) | 37 (43.5) | 99 (32.4) | 91 (14.5) | 24 (13.9) | 67 (14.8) |
| **Long-term care facility residence, n (%)** |  |  |  |  |  |  |  |  |  |
| Yes | 52 (5.4) | 9 (3.7) | 43 (5.9) | 9 (2.4) | 3 (3.6) | 6 (2.0) | 43 (7.3) | 6 (3.7) | 37 (8.7) |
| **VACCINATION STATUS AT TIME OF HOSPITAL ADMISSION** | | | | | | | | | |
| Unvaccinated, n (%) | 175 (17.2) | 23 (8.9) | 152 (20.0) | 143 (36.6) | 16 (18.8) | 127 (41.5) | 32 (5.1) | 7 (4.0) | 25 (5.5) |
| Incomplete primary series, n (%) | 26 (2.6) | 5 (1.9) | 21 (2.8) | 6 (1.5) | 1 (1.2) | 5 (1.6) | 20 (3.2) | 4 (2.3) | 16 (3.5) |
| Primary series completed but no boosters, n (%) | 528 (51.9) | 126 (48.8) | 402 (53.0) | 177 (45.3) | 35 (41.2) | 142 (46.4) | 351 (56.1) | 91 (52.6) | 260 (57.4) |
| At least one booster dose, n (%) | 288 (28.3) | 104 (40.3) | 184 (24.2) | 65 (16.6) | 33 (38.8) | 32 (10.5) | 223 (35.6) | 71 (41.0) | 152 (33.6) |
| **Number of booster doses ^c^, n (%)** |  |  |  |  |  |  |  |  |  |
| 0 | 528 (64.7) | 126 (54.8) | 402 (68.6) | 177 (73.1) | 35 (51.5) | 142 (81.6) | 351 (61.1) | 91 (56.2) | 260 (63.1) |
| 1 | 288 (35.3) | 104 (45.2) | 184 (31.4) | 65 (26.9) | 33 (48.5) | 32 (18.4) | 223 (38.9) | 71 (43.8) | 152 (36.9) |
| 2 | 0 (0.0) | 0 (0.0) | 0 (0.0) | 0 (0.0) | 0 (0.0) | 0 (0.0) | 0 (0.0) | 0 (0.0) | 0 (0.0) |
| 3+ | 0 (0.0) | 0 (0.0) | 0 (0.0) | 0 (0.0) | 0 (0.0) | 0 (0.0) | 0 (0.0) | 0 (0.0) | 0 (0.0) |
| **Time since last vaccine dose (days)** |  |  |  |  |  |  |  |  |  |
| Mean (SD) | 125.6 (78.6) | 116.8 (78.9) | 129.0 (78.3) | 143.0 (75.5) | 121.7 (81.1) | 151.2 (71.7) | 118.3 (78.8) | 114.7 (78.2) | 119.7 (79.1) |
| Median  (IQR) | 138.5  (48.0, 186.0) | 106.0  (41.5, 189.0) | 144.0  (51.0, 185.0) | 161.0  (73.8, 196.0) | 99.0  (50.0, 201.0) | 166.0  (121.0, 195.0) | 123.5  (42.0, 183.0) | 126.0  (37.2, 186.0) | 123.0  (43.0, 181.2) |
| Range | 1.0 - 327.0 | 1.0 - 282.0 | 1.0 - 327.0 | 1.0 - 316.0 | 4.0 - 282.0 | 1.0 - 316.0 | 1.0 - 327.0 | 1.0 - 266.0 | 1.0 - 327.0 |
| **Time since last vaccine dose, n (%)** |  |  |  |  |  |  |  |  |  |
| <2 months | 242 (28.7) | 78 (33.2) | 164 (27.0) | 46 (18.5) | 20 (29.0) | 26 (14.5) | 196 (33.0) | 58 (34.9) | 138 (32.2) |
| [2 - 4) months | 128 (15.2) | 43 (18.3) | 85 (14.0) | 38 (15.3) | 19 (27.5) | 19 (10.6) | 90 (15.2) | 24 (14.5) | 66 (15.4) |
| [4 - 6) months | 229 (27.2) | 44 (18.7) | 185 (30.5) | 81 (32.7) | 9 (13.0) | 72 (40.2) | 148 (24.9) | 35 (21.1) | 113 (26.4) |
| [6 - 8) months | 195 (23.2) | 60 (25.5) | 135 (22.2) | 62 (25.0) | 16 (23.2) | 46 (25.7) | 133 (22.4) | 44 (26.5) | 89 (20.8) |
| ≥8 months | 48 (5.7) | 10 (4.3) | 38 (6.3) | 21 (8.5) | 5 (7.2) | 16 (8.9) | 27 (4.5) | 5 (3.0) | 22 (5.1) |
| **SYMPTOMS** | | | | | | | | | |
| **SARI symptoms ^d,e^, n (%)** |  |  |  |  |  |  |  |  |  |
| Cough | 539 (60.8) | 121 (52.4) | 418 (63.7) | 226 (80.1) | 48 (77.4) | 178 (80.9) | 313 (51.7) | 73 (43.2) | 240 (55.0) |
| Fever | 472 (53.7) | 144 (62.3) | 328 (50.6) | 189 (67.3) | 43 (69.4) | 146 (66.7) | 283 (47.3) | 101 (59.8) | 182 (42.4) |
| Shortness of breath | 638 (71.5) | 143 (61.9) | 495 (74.9) | 221 (77.8) | 45 (72.6) | 176 (79.3) | 417 (68.6) | 98 (58.0) | 319 (72.7) |
| Anosmia, ageusia or dysgeusia | 44 (5.5) | 12 (5.8) | 32 (5.4) | 40 (17.8) | 11 (25.6) | 29 (15.9) | 4 (0.7) | 1 (0.6) | 3 (0.7) |
| **Number of SARI symptoms, n (%)** |  |  |  |  |  |  |  |  |  |
| 1 | 317 (39.9) | 98 (48.3) | 219 (37.1) | 44 (19.7) | 11 (26.2) | 33 (18.2) | 273 (47.8) | 87 (54.0) | 186 (45.4) |
| 2 | 306 (38.5) | 67 (33.0) | 239 (40.4) | 74 (33.2) | 11 (26.2) | 63 (34.8) | 232 (40.6) | 56 (34.8) | 176 (42.9) |
| 3+ | 171 (21.5) | 38 (18.7) | 133 (22.5) | 105 (47.1) | 20 (47.6) | 85 (47.0) | 66 (11.6) | 18 (11.2) | 48 (11.7) |
| **HOSPITAL OUTCOMES** | | | | | | | | | |
| **Length of stay (days)** |  |  |  |  |  |  |  |  |  |
| Mean (SD) | 11.8 (18.4) | 10.9 (13.5) | 12.1 (19.8) | 15.4 (26.6) | 13.6 (18.8) | 15.9 (28.4) | 9.5 (9.7) | 9.6 (9.9) | 9.4 (9.7) |
| Median  (IQR) | 7.0  (4.0, 13.0) | 7.0  (4.0, 12.8) | 8.0  (4.0, 13.0) | 9.0  (5.0, 16.0) | 7.0  (5.0, 14.0) | 10.0  (5.0, 17.0) | 7.0  (4.0, 11.0) | 6.0  (4.0, 12.0) | 7.0  (4.0, 11.0) |
| Range | 1.0 - 403.0 | 1.0 - 104.0 | 1.0 - 403.0 | 1.0 - 403.0 | 1.0 - 104.0 | 1.0 - 403.0 | 1.0 - 96.0 | 1.0 - 70.0 | 1.0 - 96.0 |
| **Severe outcomes, n (%)** |  |  |  |  |  |  |  |  |  |
| Hospitalization without ICU admission or in-hospital death | 798 (78.5) | 216 (83.7) | 582 (76.7) | 258 (66.0) | 68 (80.0) | 190 (62.1) | 540 (86.3) | 148 (85.5) | 392 (86.5) |
| ICU admission without in-hospital death | 104 (10.2) | 9 (3.5) | 95 (12.5) | 80 (20.5) | 6 (7.1) | 74 (24.2) | 24 (3.8) | 3 (1.7) | 21 (4.6) |
| ICU admission (with or without in-hospital death) | 142 (14.0) | 19 (7.4) | 123 (16.2) | 112 (28.6) | 13 (15.3) | 99 (32.4) | 30 (4.8) | 6 (3.5) | 24 (5.3) |
| In-hospital death | 115 (11.3) | 33 (12.8) | 82 (10.8) | 53 (13.6) | 11 (12.9) | 42 (13.7) | 62 (9.9) | 22 (12.7) | 40 (8.8) |
| **COMORBIDITIES** | | | | | | | | | |
| **Number of comorbidities other than IC, n (%)** |  |  |  |  |  |  |  |  |  |
| No comorbid conditions | 202 (19.9) | 7 (2.7) | 195 (25.7) | 119 (30.4) | 4 (4.7) | 115 (37.6) | 83 (13.3) | 3 (1.7) | 80 (17.7) |
| 1 comorbidity | 228 (22.4) | 62 (24.0) | 166 (21.9) | 91 (23.3) | 28 (32.9) | 63 (20.6) | 137 (21.9) | 34 (19.7) | 103 (22.7) |
| 2 comorbidities | 214 (21.0) | 54 (20.9) | 160 (21.1) | 74 (18.9) | 19 (22.4) | 55 (18.0) | 140 (22.4) | 35 (20.2) | 105 (23.2) |
| 3+ comorbidities | 373 (36.7) | 135 (52.3) | 238 (31.4) | 107 (27.4) | 34 (40.0) | 73 (23.9) | 266 (42.5) | 101 (58.4) | 165 (36.4) |
| **Number with specific comorbidities ^d^, n (%)** |  |  |  |  |  |  |  |  |  |
| Asthma | 76 (7.5) | 14 (5.4) | 62 (8.2) | 24 (6.1) | 8 (9.4) | 16 (5.2) | 52 (8.3) | 6 (3.5) | 46 (10.2) |
| Lung disease | 303 (29.8) | 93 (36.0) | 210 (27.7) | 75 (19.2) | 23 (27.1) | 52 (17.0) | 228 (36.4) | 70 (40.5) | 158 (34.9) |
| Cardiovascular disease | 394 (38.7) | 103 (39.9) | 291 (38.3) | 121 (30.9) | 22 (25.9) | 99 (32.4) | 273 (43.6) | 81 (46.8) | 192 (42.4) |
| Hypertension | 519 (51.0) | 133 (51.6) | 386 (50.9) | 166 (42.5) | 33 (38.8) | 133 (43.5) | 353 (56.4) | 100 (57.8) | 253 (55.8) |
| Chronic liver disease | 72 (7.1) | 21 (8.1) | 51 (6.7) | 20 (5.1) | 8 (9.4) | 12 (3.9) | 52 (8.3) | 13 (7.5) | 39 (8.6) |
| Chronic kidney disease | 191 (18.8) | 62 (24.0) | 129 (17.0) | 57 (14.6) | 21 (24.7) | 36 (11.8) | 134 (21.4) | 41 (23.7) | 93 (20.5) |
| Type 2 diabetes | 259 (25.5) | 60 (23.3) | 199 (26.2) | 97 (24.8) | 17 (20.0) | 80 (26.1) | 162 (25.9) | 43 (24.9) | 119 (26.3) |
| Cancer | 210 (20.6) | 210 (81.4) | 0 (0.0) | 66 (16.9) | 66 (77.6) | 0 (0.0) | 144 (23.0) | 144 (83.2) | 0 (0.0) |
| Main IC ^h^ | 149 (14.7) | 149 (57.8) | 0 (0.0) | 50 (12.8) | 50 (58.8) | 0 (0.0) | 99 (15.8) | 99 (57.2) | 0 (0.0) |
| **Q1 2022** |  |  |  |  |  |  |  |  |  |
| **Total** | **1,112** | **301** | **811** | **523** | **149** | **374** | **589** | **152** | **437** |
| **DEMOGRAPHICS** | | | | | | | | | |
| **Age (years)** |  |  |  |  |  |  |  |  |  |
| Mean (SD) | 68.5 (17.2) | 68.7 (14.8) | 68.4 (18.0) | 68.7 (16.2) | 68.9 (15.1) | 68.6 (16.7) | 68.3 (17.9) | 68.5 (14.5) | 68.3 (19.0) |
| Median  (IQR) | 72.0  (58.0, 82.0) | 71.0  (58.0, 79.0) | 72.0  (58.0, 83.0) | 72.0  (59.0, 81.0) | 72.0  (59.0, 79.0) | 71.0  (59.0, 81.0) | 72.0  (58.0, 83.0) | 70.5  (58.0, 79.0) | 73.0  (58.0, 83.0) |
| Range | 18.0 - 101.0 | 21.0 - 95.0 | 18.0 - 101.0 | 20.0 - 99.0 | 21.0 - 95.0 | 20.0 - 99.0 | 18.0 - 101.0 | 28.0 - 94.0 | 18.0 - 101.0 |
| **Sex, n (%)** |  |  |  |  |  |  |  |  |  |
| Male | 630 (56.7) | 172 (57.1) | 458 (56.5) | 300 (57.4) | 83 (55.7) | 217 (58.0) | 330 (56.0) | 89 (58.6) | 241 (55.1) |
| Female | 482 (43.3) | 129 (42.9) | 353 (43.5) | 223 (42.6) | 66 (44.3) | 157 (42.0) | 259 (44.0) | 63 (41.4) | 196 (44.9) |
| **Country, n (%)** |  |  |  |  |  |  |  |  |  |
| Belgium | 97 (8.7) | 20 (6.6) | 77 (9.5) | 57 (10.9) | 13 (8.7) | 44 (11.8) | 40 (6.8) | 7 (4.6) | 33 (7.6) |
| Italy | 503 (45.2) | 110 (36.5) | 393 (48.5) | 212 (40.5) | 50 (33.6) | 162 (43.3) | 291 (49.4) | 60 (39.5) | 231 (52.9) |
| Spain | 512 (46.0) | 171 (56.8) | 341 (42.0) | 254 (48.6) | 86 (57.7) | 168 (44.9) | 258 (43.8) | 85 (55.9) | 173 (39.6) |
| **Study site, n (%)** |  |  |  |  |  |  |  |  |  |
| CIRI-IT | 503 (45.2) | 110 (36.5) | 393 (48.5) | 212 (40.5) | 50 (33.6) | 162 (43.3) | 291 (49.4) | 60 (39.5) | 231 (52.9) |
| GTPUH | 315 (28.3) | 97 (32.2) | 218 (26.9) | 105 (20.1) | 27 (18.1) | 78 (20.9) | 210 (35.7) | 70 (46.1) | 140 (32.0) |
| CHU Saint-Pierre | 25 (2.2) | 4 (1.3) | 21 (2.6) | 9 (1.7) | 1 (0.7) | 8 (2.1) | 16 (2.7) | 3 (2.0) | 13 (3.0) |
| UZA | 72 (6.5) | 16 (5.3) | 56 (6.9) | 48 (9.2) | 12 (8.1) | 36 (9.6) | 24 (4.1) | 4 (2.6) | 20 (4.6) |
| HUVH | 197 (17.7) | 74 (24.6) | 123 (15.2) | 149 (28.5) | 59 (39.6) | 90 (24.1) | 48 (8.1) | 15 (9.9) | 33 (7.6) |
| **Smoking, n (%)** |  |  |  |  |  |  |  |  |  |
| Never smoker | 336 (30.2) | 93 (30.9) | 243 (30.0) | 186 (35.6) | 56 (37.6) | 130 (34.8) | 150 (25.5) | 37 (24.3) | 113 (25.9) |
| Former smoker | 232 (20.9) | 80 (26.6) | 152 (18.7) | 114 (21.8) | 37 (24.8) | 77 (20.6) | 118 (20.0) | 43 (28.3) | 75 (17.2) |
| Current smoker | 101 (9.1) | 25 (8.3) | 76 (9.4) | 45 (8.6) | 12 (8.1) | 33 (8.8) | 56 (9.5) | 13 (8.6) | 43 (9.8) |
| Missing | 443 (39.8) | 103 (34.2) | 340 (41.9) | 178 (34.0) | 44 (29.5) | 134 (35.8) | 265 (45.0) | 59 (38.8) | 206 (47.1) |
| **Long-term care facility residence, n (%)** |  |  |  |  |  |  |  |  |  |
| Yes | 33 (3.0) | 7 (2.4) | 26 (3.2) | 13 (2.5) | 4 (2.8) | 9 (2.4) | 20 (3.4) | 3 (2.0) | 17 (3.9) |
| **VACCINATION STATUS AT TIME OF HOSPITAL ADMISSION** | | | | | | | | | |
| Unvaccinated, n (%) | 142 (12.8) | 16 (5.3) | 126 (15.5) | 104 (19.9) | 8 (5.4) | 96 (25.7) | 38 (6.5) | 8 (5.3) | 30 (6.9) |
| Incomplete primary series, n (%) | 30 (2.7) | 7 (2.3) | 23 (2.8) | 15 (2.9) | 5 (3.4) | 10 (2.7) | 15 (2.5) | 2 (1.3) | 13 (3.0) |
| Primary series completed but no boosters, n (%) | 221 (19.9) | 56 (18.6) | 165 (20.3) | 122 (23.3) | 31 (20.8) | 91 (24.3) | 99 (16.8) | 25 (16.4) | 74 (16.9) |
| At least one booster dose, n (%) | 719 (64.7) | 222 (73.8) | 497 (61.3) | 282 (53.9) | 105 (70.5) | 177 (47.3) | 437 (74.2) | 117 (77.0) | 320 (73.2) |
| **Number of booster doses ^c^, n (%)** |  |  |  |  |  |  |  |  |  |
| 0 | 221 (23.5) | 56 (20.1) | 165 (24.9) | 122 (30.2) | 31 (22.8) | 91 (34.0) | 99 (18.5) | 25 (17.6) | 74 (18.8) |
| 1 | 715 (76.1) | 220 (79.1) | 495 (74.8) | 280 (69.3) | 104 (76.5) | 176 (65.7) | 435 (81.2) | 116 (81.7) | 319 (81.0) |
| 2 | 4 (0.4) | 2 (0.7) | 2 (0.3) | 2 (0.5) | 1 (0.7) | 1 (0.4) | 2 (0.4) | 1 (0.7) | 1 (0.3) |
| 3+ | 0 (0.0) | 0 (0.0) | 0 (0.0) | 0 (0.0) | 0 (0.0) | 0 (0.0) | 0 (0.0) | 0 (0.0) | 0 (0.0) |
| **Time since last vaccine dose (days)** |  |  |  |  |  |  |  |  |  |
| Mean (SD) | 113.9 (78.0) | 117.0 (76.9) | 112.7 (78.5) | 129.1 (84.3) | 128.1 (84.6) | 129.6 (84.4) | 102.4 (70.7) | 106.1 (67.0) | 101.1 (72.0) |
| Median  (IQR) | 92.0  (60.0, 143.0) | 99.0  (66.0, 146.0) | 90.0  (58.0, 141.0) | 106.0  (67.0, 177.0) | 106.0  (68.0, 155.0) | 104.0  (67.0, 194.0) | 83.0  (56.0, 124.5) | 91.5  (62.8, 133.0) | 83.0  (55.0, 121.0) |
| Range | 1.0 - 374.0 | 1.0 - 374.0 | 2.0 - 367.0 | 1.0 - 367.0 | 1.0 - 345.0 | 4.0 - 367.0 | 2.0 - 374.0 | 2.0 - 374.0 | 2.0 - 358.0 |
| **Time since last vaccine dose, n (%)** |  |  |  |  |  |  |  |  |  |
| <2 months | 236 (24.3) | 60 (21.1) | 176 (25.7) | 82 (19.6) | 26 (18.4) | 56 (20.1) | 154 (27.9) | 34 (23.6) | 120 (29.5) |
| [2 - 4) months | 414 (42.7) | 127 (44.6) | 287 (41.9) | 162 (38.7) | 59 (41.8) | 103 (37.1) | 252 (45.7) | 68 (47.2) | 184 (45.2) |
| [4 - 6) months | 148 (15.3) | 53 (18.6) | 95 (13.9) | 74 (17.7) | 28 (19.9) | 46 (16.5) | 74 (13.4) | 25 (17.4) | 49 (12.0) |
| [6 - 8) months | 72 (7.4) | 15 (5.3) | 57 (8.3) | 40 (9.5) | 6 (4.3) | 34 (12.2) | 32 (5.8) | 9 (6.2) | 23 (5.7) |
| ≥8 months | 100 (10.3) | 30 (10.5) | 70 (10.2) | 61 (14.6) | 22 (15.6) | 39 (14.0) | 39 (7.1) | 8 (5.6) | 31 (7.6) |
| **SYMPTOMS** | | | | | | | | | |
| **SARI symptoms ^d,e^, n (%)** |  |  |  |  |  |  |  |  |  |
| Cough | 672 (66.8) | 158 (59.8) | 514 (69.3) | 298 (68.2) | 76 (63.9) | 222 (69.8) | 374 (65.7) | 82 (56.6) | 292 (68.9) |
| Fever | 691 (68.6) | 190 (72.0) | 501 (67.3) | 308 (70.3) | 93 (78.2) | 215 (67.4) | 383 (67.2) | 97 (66.9) | 286 (67.3) |
| Shortness of breath | 722 (71.4) | 181 (68.6) | 541 (72.4) | 333 (75.2) | 82 (68.9) | 251 (77.5) | 389 (68.5) | 99 (68.3) | 290 (68.6) |
| Anosmia, ageusia or dysgeusia | 49 (5.1) | 10 (4.0) | 39 (5.5) | 40 (9.7) | 8 (7.2) | 32 (10.6) | 9 (1.6) | 2 (1.4) | 7 (1.7) |
| **Number of SARI symptoms, n (%)** |  |  |  |  |  |  |  |  |  |
| 1 | 247 (25.8) | 80 (31.9) | 167 (23.7) | 91 (22.1) | 27 (24.3) | 64 (21.3) | 156 (28.6) | 53 (37.9) | 103 (25.4) |
| 2 | 373 (39.0) | 87 (34.7) | 286 (40.5) | 151 (36.7) | 43 (38.7) | 108 (35.9) | 222 (40.7) | 44 (31.4) | 178 (44.0) |
| 3+ | 337 (35.2) | 84 (33.5) | 253 (35.8) | 170 (41.3) | 41 (36.9) | 129 (42.9) | 167 (30.6) | 43 (30.7) | 124 (30.6) |
| **HOSPITAL OUTCOMES** | | | | | | | | | |
| **Length of stay (days)** |  |  |  |  |  |  |  |  |  |
| Mean (SD) | 9.4 (10.7) | 10.9 (14.2) | 8.9 (9.0) | 11.4 (13.7) | 14.1 (18.4) | 10.4 (11.2) | 7.6 (6.4) | 7.6 (6.9) | 7.6 (6.2) |
| Median  (IQR) | 6.0  (4.0, 12.0) | 7.0  (4.0, 13.0) | 6.0  (4.0, 11.0) | 7.0  (4.0, 14.0) | 8.0  (5.0, 15.0) | 6.0  (4.0, 14.0) | 6.0  (3.0, 10.0) | 6.0  (3.0, 9.2) | 6.0  (4.0, 10.0) |
| Range | 1.0 - 141.0 | 1.0 - 141.0 | 1.0 - 105.0 | 1.0 - 141.0 | 1.0 - 141.0 | 1.0 - 105.0 | 1.0 - 46.0 | 1.0 - 46.0 | 1.0 - 41.0 |
| **Severe outcomes, n (%)** |  |  |  |  |  |  |  |  |  |
| Hospitalization without ICU admission or in-hospital death | 1006 (90.5) | 269 (89.4) | 737 (90.9) | 438 (83.7) | 125 (83.9) | 313 (83.7) | 568 (96.4) | 144 (94.7) | 424 (97.0) |
| ICU admission without in-hospital death | 46 (4.1) | 12 (4.0) | 34 (4.2) | 37 (7.1) | 9 (6.0) | 28 (7.5) | 9 (1.5) | 3 (2.0) | 6 (1.4) |
| ICU admission (with or without in-hospital death) | 64 (5.8) | 18 (6.0) | 46 (5.7) | 54 (10.3) | 14 (9.4) | 40 (10.7) | 10 (1.7) | 4 (2.6) | 6 (1.4) |
| In-hospital death | 60 (5.4) | 20 (6.6) | 40 (4.9) | 48 (9.2) | 15 (10.1) | 33 (8.8) | 12 (2.0) | 5 (3.3) | 7 (1.6) |
| **COMORBIDITIES** | | | | | | | | | |
| **Number of comorbidities other than IC, n (%)** |  |  |  |  |  |  |  |  |  |
| No comorbid conditions | 214 (19.2) | 10 (3.3) | 204 (25.2) | 95 (18.2) | 5 (3.4) | 90 (24.1) | 119 (20.2) | 5 (3.3) | 114 (26.1) |
| 1 comorbidity | 272 (24.5) | 88 (29.2) | 184 (22.7) | 126 (24.1) | 44 (29.5) | 82 (21.9) | 146 (24.8) | 44 (28.9) | 102 (23.3) |
| 2 comorbidities | 247 (22.2) | 68 (22.6) | 179 (22.1) | 121 (23.1) | 34 (22.8) | 87 (23.3) | 126 (21.4) | 34 (22.4) | 92 (21.1) |
| 3+ comorbidities | 379 (34.1) | 135 (44.9) | 244 (30.1) | 181 (34.6) | 66 (44.3) | 115 (30.7) | 198 (33.6) | 69 (45.4) | 129 (29.5) |
| **Number with specific comorbidities ^d^, n (%)** |  |  |  |  |  |  |  |  |  |
| Asthma | 66 (5.9) | 15 (5.0) | 51 (6.3) | 34 (6.5) | 9 (6.0) | 25 (6.7) | 32 (5.4) | 6 (3.9) | 26 (5.9) |
| Lung disease | 285 (25.6) | 84 (27.9) | 201 (24.8) | 114 (21.8) | 36 (24.2) | 78 (20.9) | 171 (29.0) | 48 (31.6) | 123 (28.1) |
| Cardiovascular disease | 432 (38.8) | 113 (37.5) | 319 (39.3) | 218 (41.7) | 63 (42.3) | 155 (41.4) | 214 (36.3) | 50 (32.9) | 164 (37.5) |
| Hypertension | 549 (49.4) | 137 (45.5) | 412 (50.8) | 267 (51.1) | 70 (47.0) | 197 (52.7) | 282 (47.9) | 67 (44.1) | 215 (49.2) |
| Chronic liver disease | 71 (6.4) | 30 (10.0) | 41 (5.1) | 34 (6.5) | 14 (9.4) | 20 (5.3) | 37 (6.3) | 16 (10.5) | 21 (4.8) |
| Chronic kidney disease | 174 (15.6) | 46 (15.3) | 128 (15.8) | 88 (16.8) | 22 (14.8) | 66 (17.6) | 86 (14.6) | 24 (15.8) | 62 (14.2) |
| Type 2 diabetes | 318 (28.6) | 82 (27.2) | 236 (29.1) | 154 (29.4) | 38 (25.5) | 116 (31.0) | 164 (27.8) | 44 (28.9) | 120 (27.5) |
| Cancer | 243 (21.9) | 243 (80.7) | 0 (0.0) | 112 (21.4) | 112 (75.2) | 0 (0.0) | 131 (22.2) | 131 (86.2) | 0 (0.0) |
| Main IC ^h^ | 111 (10.0) | 111 (36.9) | 0 (0.0) | 55 (10.5) | 55 (36.9) | 0 (0.0) | 56 (9.5) | 56 (36.8) | 0 (0.0) |
| **Q2 2022** |  |  |  |  |  |  |  |  |  |
| **Total** | **538** | **177** | **361** | **293** | **112** | **181** | **245** | **65** | **180** |
| **DEMOGRAPHICS** | | | | | | | | | |
| **Age (years)** |  |  |  |  |  |  |  |  |  |
| Mean (SD) | 70.1 (16.0) | 69.4 (14.3) | 70.4 (16.8) | 70.2 (15.8) | 69.8 (14.2) | 70.6 (16.8) | 69.9 (16.3) | 68.8 (14.6) | 70.3 (16.9) |
| Median  (IQR) | 74.0  (62.0, 82.0) | 72.0  (62.0, 79.0) | 75.0  (62.0, 83.0) | 74.0  (62.0, 82.0) | 72.0  (62.0, 79.2) | 75.0  (62.0, 83.0) | 74.0  (61.0, 82.0) | 70.0  (61.0, 78.0) | 74.5  (61.8, 83.0) |
| Range | 19.0 - 102.0 | 21.0 - 95.0 | 19.0 - 102.0 | 22.0 - 102.0 | 28.0 - 95.0 | 22.0 - 102.0 | 19.0 - 95.0 | 21.0 - 95.0 | 19.0 - 95.0 |
| **Sex, n (%)** |  |  |  |  |  |  |  |  |  |
| Male | 315 (58.6) | 110 (62.1) | 205 (56.8) | 174 (59.4) | 72 (64.3) | 102 (56.4) | 141 (57.6) | 38 (58.5) | 103 (57.2) |
| Female | 223 (41.4) | 67 (37.9) | 156 (43.2) | 119 (40.6) | 40 (35.7) | 79 (43.6) | 104 (42.4) | 27 (41.5) | 77 (42.8) |
| **Country, n (%)** |  |  |  |  |  |  |  |  |  |
| Belgium | 20 (3.7) | 6 (3.4) | 14 (3.9) | 13 (4.4) | 5 (4.5) | 8 (4.4) | 7 (2.9) | 1 (1.5) | 6 (3.3) |
| Italy | 212 (39.4) | 45 (25.4) | 167 (46.3) | 80 (27.3) | 21 (18.8) | 59 (32.6) | 132 (53.9) | 24 (36.9) | 108 (60.0) |
| Spain | 306 (56.9) | 126 (71.2) | 180 (49.9) | 200 (68.3) | 86 (76.8) | 114 (63.0) | 106 (43.3) | 40 (61.5) | 66 (36.7) |
| **Study site, n (%)** |  |  |  |  |  |  |  |  |  |
| CIRI-IT | 212 (39.4) | 45 (25.4) | 167 (46.3) | 80 (27.3) | 21 (18.8) | 59 (32.6) | 132 (53.9) | 24 (36.9) | 108 (60.0) |
| GTPUH | 103 (19.1) | 38 (21.5) | 65 (18.0) | 31 (10.6) | 10 (8.9) | 21 (11.6) | 72 (29.4) | 28 (43.1) | 44 (24.4) |
| CHU Saint-Pierre | 2 (0.4) | 0 (0.0) | 2 (0.6) | 1 (0.3) | 0 (0.0) | 1 (0.6) | 1 (0.4) | 0 (0.0) | 1 (0.6) |
| UZA | 18 (3.3) | 6 (3.4) | 12 (3.3) | 12 (4.1) | 5 (4.5) | 7 (3.9) | 6 (2.4) | 1 (1.5) | 5 (2.8) |
| HUVH | 203 (37.7) | 88 (49.7) | 115 (31.9) | 169 (57.7) | 76 (67.9) | 93 (51.4) | 34 (13.9) | 12 (18.5) | 22 (12.2) |
| **Smoking, n (%)** |  |  |  |  |  |  |  |  |  |
| Never smoker | 181 (33.6) | 65 (36.7) | 116 (32.1) | 120 (41.0) | 45 (40.2) | 75 (41.4) | 61 (24.9) | 20 (30.8) | 41 (22.8) |
| Former smoker | 164 (30.5) | 62 (35.0) | 102 (28.3) | 103 (35.2) | 44 (39.3) | 59 (32.6) | 61 (24.9) | 18 (27.7) | 43 (23.9) |
| Current smoker | 72 (13.4) | 20 (11.3) | 52 (14.4) | 39 (13.3) | 11 (9.8) | 28 (15.5) | 33 (13.5) | 9 (13.8) | 24 (13.3) |
| Missing | 121 (22.5) | 30 (16.9) | 91 (25.2) | 31 (10.6) | 12 (10.7) | 19 (10.5) | 90 (36.7) | 18 (27.7) | 72 (40.0) |
| **Long-term care facility residence, n (%)** |  |  |  |  |  |  |  |  |  |
| Yes | 24 (4.6) | 7 (4.1) | 17 (4.8) | 14 (5.0) | 4 (3.8) | 10 (5.7) | 10 (4.1) | 3 (4.6) | 7 (3.9) |
| **VACCINATION STATUS AT TIME OF HOSPITAL ADMISSION** | | | | | | | | | |
| Unvaccinated, n (%) | 33 (6.1) | 8 (4.5) | 25 (6.9) | 22 (7.5) | 5 (4.5) | 17 (9.4) | 11 (4.5) | 3 (4.6) | 8 (4.4) |
| Incomplete primary series, n (%) | 7 (1.3) | 3 (1.7) | 4 (1.1) | 4 (1.4) | 2 (1.8) | 2 (1.1) | 3 (1.2) | 1 (1.5) | 2 (1.1) |
| Primary series completed but no boosters, n (%) | 57 (10.6) | 21 (11.9) | 36 (10.0) | 24 (8.2) | 11 (9.8) | 13 (7.2) | 33 (13.5) | 10 (15.4) | 23 (12.8) |
| At least one booster dose, n (%) | 441 (82.0) | 145 (81.9) | 296 (82.0) | 243 (82.9) | 94 (83.9) | 149 (82.3) | 198 (80.8) | 51 (78.5) | 147 (81.7) |
| **Number of booster doses ^c^, n (%)** |  |  |  |  |  |  |  |  |  |
| 0 | 57 (11.4) | 21 (12.7) | 36 (10.8) | 24 (9.0) | 11 (10.5) | 13 (8.0) | 33 (14.3) | 10 (16.4) | 23 (13.5) |
| 1 | 414 (83.1) | 127 (76.5) | 287 (86.4) | 223 (83.5) | 80 (76.2) | 143 (88.3) | 191 (82.7) | 47 (77.0) | 144 (84.7) |
| 2 | 27 (5.4) | 18 (10.8) | 9 (2.7) | 20 (7.5) | 14 (13.3) | 6 (3.7) | 7 (3.0) | 4 (6.6) | 3 (1.8) |
| 3+ | 0 (0.0) | 0 (0.0) | 0 (0.0) | 0 (0.0) | 0 (0.0) | 0 (0.0) | 0 (0.0) | 0 (0.0) | 0 (0.0) |
| **Time since last vaccine dose (days)** |  |  |  |  |  |  |  |  |  |
| Mean (SD) | 175.0 (71.3) | 179.7 (83.2) | 172.7 (64.4) | 184.9 (71.9) | 186.4 (85.9) | 183.9 (61.3) | 163.6 (69.0) | 168.0 (77.7) | 162.0 (65.7) |
| Median  (IQR) | 173.0  (132.0, 210.0) | 179.0  (122.0, 221.0) | 170.0  (136.8, 200.2) | 187.0  (146.5, 219.0) | 196.0  (126.0, 231.0) | 184.0  (151.5, 215.5) | 159.0  (122.2, 191.8) | 172.5  (113.5, 216.5) | 157.5  (123.0, 188.2) |
| Range | 1.0 - 440.0 | 1.0 - 411.0 | 1.0 - 440.0 | 1.0 - 440.0 | 1.0 - 411.0 | 13.0 - 440.0 | 1.0 - 428.0 | 23.0 - 383.0 | 1.0 - 428.0 |
| **Time since last vaccine dose, n (%)** |  |  |  |  |  |  |  |  |  |
| <2 months | 22 (4.4) | 14 (8.3) | 8 (2.4) | 13 (4.8) | 10 (9.3) | 3 (1.8) | 9 (3.8) | 4 (6.5) | 5 (2.9) |
| [2 - 4) months | 78 (15.4) | 27 (16.0) | 51 (15.2) | 31 (11.4) | 14 (13.1) | 17 (10.4) | 47 (20.1) | 13 (21.0) | 34 (19.8) |
| [4 - 6) months | 181 (35.8) | 44 (26.0) | 137 (40.8) | 79 (29.2) | 23 (21.5) | 56 (34.1) | 102 (43.6) | 21 (33.9) | 81 (47.1) |
| [6 - 8) months | 167 (33.1) | 55 (32.5) | 112 (33.3) | 111 (41.0) | 39 (36.4) | 72 (43.9) | 56 (23.9) | 16 (25.8) | 40 (23.3) |
| ≥8 months | 57 (11.3) | 29 (17.2) | 28 (8.3) | 37 (13.7) | 21 (19.6) | 16 (9.8) | 20 (8.5) | 8 (12.9) | 12 (7.0) |
| **SYMPTOMS** | | | | | | | | | |
| **SARI symptoms ^d,e^, n (%)** |  |  |  |  |  |  |  |  |  |
| Cough | 391 (72.8) | 122 (68.9) | 269 (74.7) | 201 (68.8) | 76 (67.9) | 125 (69.4) | 190 (77.6) | 46 (70.8) | 144 (80.0) |
| Fever | 353 (65.9) | 107 (60.5) | 246 (68.5) | 178 (61.2) | 64 (57.1) | 114 (63.7) | 175 (71.4) | 43 (66.2) | 132 (73.3) |
| Shortness of breath | 366 (68.3) | 112 (63.6) | 254 (70.6) | 199 (68.2) | 70 (62.5) | 129 (71.7) | 167 (68.4) | 42 (65.6) | 125 (69.4) |
| Anosmia, ageusia or dysgeusia | 17 (3.3) | 5 (3.0) | 12 (3.5) | 12 (4.3) | 4 (3.8) | 8 (4.7) | 5 (2.1) | 1 (1.6) | 4 (2.3) |
| **Number of SARI symptoms, n (%)** |  |  |  |  |  |  |  |  |  |
| 1 | 132 (25.6) | 54 (32.0) | 78 (22.5) | 87 (31.5) | 40 (38.1) | 47 (27.5) | 45 (18.8) | 14 (21.9) | 31 (17.7) |
| 2 | 206 (40.0) | 68 (40.2) | 138 (39.9) | 98 (35.5) | 34 (32.4) | 64 (37.4) | 108 (45.2) | 34 (53.1) | 74 (42.3) |
| 3+ | 177 (34.4) | 47 (27.8) | 130 (37.6) | 91 (33.0) | 31 (29.5) | 60 (35.1) | 86 (36.0) | 16 (25.0) | 70 (40.0) |
| **HOSPITAL OUTCOMES** | | | | | | | | | |
| **Length of stay (days)** |  |  |  |  |  |  |  |  |  |
| Mean (SD) | 10.3 (14.1) | 13.1 (19.6) | 8.9 (10.2) | 11.2 (15.7) | 14.6 (21.5) | 9.2 (10.2) | 9.2 (11.9) | 10.4 (15.5) | 8.7 (10.3) |
| Median  (IQR) | 6.0  (3.0, 11.0) | 6.0  (3.0, 11.0) | 6.0  (3.0, 11.0) | 6.0  (3.0, 12.0) | 6.5  (3.0, 17.2) | 6.0  (3.0, 11.0) | 6.0  (3.0, 11.0) | 6.0  (3.0, 10.0) | 6.0  (2.0, 11.0) |
| Range | 1.0 - 146.0 | 1.0 - 146.0 | 1.0 - 94.0 | 1.0 - 146.0 | 1.0 - 146.0 | 1.0 - 82.0 | 1.0 - 94.0 | 1.0 - 93.0 | 1.0 - 94.0 |
| **Severe outcomes, n (%)** |  |  |  |  |  |  |  |  |  |
| Hospitalization without ICU admission or in-hospital death | 492 (91.6) | 157 (88.7) | 335 (93.1) | 256 (87.7) | 95 (84.8) | 161 (89.4) | 236 (96.3) | 62 (95.4) | 174 (96.7) |
| ICU admission without in-hospital death | 16 (3.0) | 7 (4.0) | 9 (2.5) | 12 (4.1) | 4 (3.6) | 8 (4.4) | 4 (1.6) | 3 (4.6) | 1 (0.6) |
| ICU admission (with or without in-hospital death) | 22 (4.1) | 11 (6.2) | 11 (3.0) | 17 (5.8) | 8 (7.1) | 9 (5.0) | 5 (2.0) | 3 (4.6) | 2 (1.1) |
| In-hospital death | 29 (5.4) | 13 (7.3) | 16 (4.4) | 24 (8.2) | 13 (11.6) | 11 (6.1) | 5 (2.0) | 0 (0.0) | 5 (2.8) |
| **COMORBIDITIES** | | | | | | | | | |
| **Number of comorbidities other than IC, n (%)** |  |  |  |  |  |  |  |  |  |
| No comorbid conditions | 87 (16.2) | 9 (5.1) | 78 (21.6) | 35 (11.9) | 6 (5.4) | 29 (16.0) | 52 (21.2) | 3 (4.6) | 49 (27.2) |
| 1 comorbidity | 110 (20.4) | 34 (19.2) | 76 (21.1) | 63 (21.5) | 22 (19.6) | 41 (22.7) | 47 (19.2) | 12 (18.5) | 35 (19.4) |
| 2 comorbidities | 131 (24.3) | 41 (23.2) | 90 (24.9) | 78 (26.6) | 25 (22.3) | 53 (29.3) | 53 (21.6) | 16 (24.6) | 37 (20.6) |
| 3+ comorbidities | 210 (39.0) | 93 (52.5) | 117 (32.4) | 117 (39.9) | 59 (52.7) | 58 (32.0) | 93 (38.0) | 34 (52.3) | 59 (32.8) |
| **Number with specific comorbidities ^d^, n (%)** |  |  |  |  |  |  |  |  |  |
| Asthma | 28 (5.2) | 7 (4.0) | 21 (5.8) | 17 (5.8) | 3 (2.7) | 14 (7.7) | 11 (4.5) | 4 (6.2) | 7 (3.9) |
| Lung disease | 195 (36.2) | 72 (40.7) | 123 (34.1) | 97 (33.1) | 43 (38.4) | 54 (29.8) | 98 (40.0) | 29 (44.6) | 69 (38.3) |
| Cardiovascular disease | 235 (43.7) | 78 (44.1) | 157 (43.5) | 133 (45.4) | 52 (46.4) | 81 (44.8) | 102 (41.6) | 26 (40.0) | 76 (42.2) |
| Hypertension | 286 (53.2) | 87 (49.2) | 199 (55.1) | 170 (58.0) | 60 (53.6) | 110 (60.8) | 116 (47.3) | 27 (41.5) | 89 (49.4) |
| Chronic liver disease | 30 (5.6) | 11 (6.2) | 19 (5.3) | 17 (5.8) | 7 (6.2) | 10 (5.5) | 13 (5.3) | 4 (6.2) | 9 (5.0) |
| Chronic kidney disease | 89 (16.5) | 38 (21.5) | 51 (14.1) | 59 (20.1) | 25 (22.3) | 34 (18.8) | 30 (12.2) | 13 (20.0) | 17 (9.4) |
| Type 2 diabetes | 125 (23.2) | 42 (23.7) | 83 (23.0) | 62 (21.2) | 26 (23.2) | 36 (19.9) | 63 (25.7) | 16 (24.6) | 47 (26.1) |
| Cancer | 132 (24.5) | 132 (74.6) | 0 (0.0) | 80 (27.3) | 80 (71.4) | 0 (0.0) | 52 (21.2) | 52 (80.0) | 0 (0.0) |
| Main IC ^h^ | 83 (15.4) | 83 (46.9) | 0 (0.0) | 53 (18.1) | 53 (47.3) | 0 (0.0) | 30 (12.2) | 30 (46.2) | 0 (0.0) |
| **Q3 2022** |  |  |  |  |  |  |  |  |  |
| **Total** | **387** | **143** | **244** | **160** | **71** | **89** | **227** | **72** | **155** |
| **DEMOGRAPHICS** | | | | | | | | | |
| **Age (years)** |  |  |  |  |  |  |  |  |  |
| Mean (SD) | 67.3 (16.3) | 66.2 (14.6) | 68.0 (17.2) | 69.4 (14.9) | 68.8 (12.9) | 69.9 (16.4) | 65.8 (17.0) | 63.6 (15.8) | 66.8 (17.5) |
| Median  (IQR) | 70.0  (58.0, 79.5) | 67.0  (59.5, 77.0) | 73.0  (58.0, 81.0) | 72.5  (60.8, 81.0) | 70.0  (60.5, 79.5) | 73.0  (62.0, 82.0) | 68.0  (56.0, 78.5) | 65.0  (56.8, 75.0) | 72.0  (56.0, 81.0) |
| Range | 18.0 - 96.0 | 18.0 - 90.0 | 18.0 - 96.0 | 19.0 - 94.0 | 35.0 - 90.0 | 19.0 - 94.0 | 18.0 - 96.0 | 18.0 - 88.0 | 18.0 - 96.0 |
| **Sex, n (%)** |  |  |  |  |  |  |  |  |  |
| Male | 219 (56.6) | 89 (62.2) | 130 (53.3) | 99 (61.9) | 44 (62.0) | 55 (61.8) | 120 (52.9) | 45 (62.5) | 75 (48.4) |
| Female | 168 (43.4) | 54 (37.8) | 114 (46.7) | 61 (38.1) | 27 (38.0) | 34 (38.2) | 107 (47.1) | 27 (37.5) | 80 (51.6) |
| **Country, n (%)** |  |  |  |  |  |  |  |  |  |
| Belgium | 25 (6.5) | 7 (4.9) | 18 (7.4) | 13 (8.1) | 4 (5.6) | 9 (10.1) | 12 (5.3) | 3 (4.2) | 9 (5.8) |
| Italy | 87 (22.5) | 23 (16.1) | 64 (26.2) | 46 (28.8) | 12 (16.9) | 34 (38.2) | 41 (18.1) | 11 (15.3) | 30 (19.4) |
| Spain | 275 (71.1) | 113 (79.0) | 162 (66.4) | 101 (63.1) | 55 (77.5) | 46 (51.7) | 174 (76.7) | 58 (80.6) | 116 (74.8) |
| **Study site, n (%)** |  |  |  |  |  |  |  |  |  |
| CIRI-IT | 87 (22.5) | 23 (16.1) | 64 (26.2) | 46 (28.8) | 12 (16.9) | 34 (38.2) | 41 (18.1) | 11 (15.3) | 30 (19.4) |
| GTPUH | 117 (30.2) | 35 (24.5) | 82 (33.6) | 23 (14.4) | 13 (18.3) | 10 (11.2) | 94 (41.4) | 22 (30.6) | 72 (46.5) |
| CHU Saint-Pierre | 9 (2.3) | 3 (2.1) | 6 (2.5) | 4 (2.5) | 2 (2.8) | 2 (2.2) | 5 (2.2) | 1 (1.4) | 4 (2.6) |
| UZA | 16 (4.1) | 4 (2.8) | 12 (4.9) | 9 (5.6) | 2 (2.8) | 7 (7.9) | 7 (3.1) | 2 (2.8) | 5 (3.2) |
| HUVH | 158 (40.8) | 78 (54.5) | 80 (32.8) | 78 (48.8) | 42 (59.2) | 36 (40.4) | 80 (35.2) | 36 (50.0) | 44 (28.4) |
| **Smoking, n (%)** |  |  |  |  |  |  |  |  |  |
| Never smoker | 171 (44.2) | 60 (42.0) | 111 (45.5) | 66 (41.2) | 30 (42.3) | 36 (40.4) | 105 (46.3) | 30 (41.7) | 75 (48.4) |
| Former smoker | 100 (25.8) | 39 (27.3) | 61 (25.0) | 44 (27.5) | 19 (26.8) | 25 (28.1) | 56 (24.7) | 20 (27.8) | 36 (23.2) |
| Current smoker | 78 (20.2) | 27 (18.9) | 51 (20.9) | 37 (23.1) | 15 (21.1) | 22 (24.7) | 41 (18.1) | 12 (16.7) | 29 (18.7) |
| Missing | 38 (9.8) | 17 (11.9) | 21 (8.6) | 13 (8.1) | 7 (9.9) | 6 (6.7) | 25 (11.0) | 10 (13.9) | 15 (9.7) |
| **Long-term care facility residence, n (%)** |  |  |  |  |  |  |  |  |  |
| Yes | 20 (5.3) | 2 (1.4) | 18 (7.6) | 8 (5.2) | 1 (1.5) | 7 (8.0) | 12 (5.4) | 1 (1.4) | 11 (7.3) |
| **VACCINATION STATUS AT TIME OF HOSPITAL ADMISSION** | | | | | | | | | |
| Unvaccinated, n (%) | 27 (7.0) | 9 (6.3) | 18 (7.4) | 14 (8.8) | 5 (7.0) | 9 (10.1) | 13 (5.7) | 4 (5.6) | 9 (5.8) |
| Incomplete primary series, n (%) | 7 (1.8) | 3 (2.1) | 4 (1.6) | 1 (0.6) | 1 (1.4) | 0 (0.0) | 6 (2.6) | 2 (2.8) | 4 (2.6) |
| Primary series completed but no boosters, n (%) | 32 (8.3) | 11 (7.7) | 21 (8.6) | 13 (8.1) | 7 (9.9) | 6 (6.7) | 19 (8.4) | 4 (5.6) | 15 (9.7) |
| At least one booster dose, n (%) | 321 (82.9) | 120 (83.9) | 201 (82.4) | 132 (82.5) | 58 (81.7) | 74 (83.1) | 189 (83.3) | 62 (86.1) | 127 (81.9) |
| **Number of booster doses ^c^, n (%)** |  |  |  |  |  |  |  |  |  |
| 0 | 32 (9.1) | 11 (8.4) | 21 (9.5) | 13 (9.0) | 7 (10.8) | 6 (7.5) | 19 (9.1) | 4 (6.1) | 15 (10.6) |
| 1 | 279 (79.0) | 93 (71.0) | 186 (83.8) | 109 (75.2) | 43 (66.2) | 66 (82.5) | 170 (81.7) | 50 (75.8) | 120 (84.5) |
| 2 | 42 (11.9) | 27 (20.6) | 15 (6.8) | 23 (15.9) | 15 (23.1) | 8 (10.0) | 19 (9.1) | 12 (18.2) | 7 (4.9) |
| 3+ | 0 (0.0) | 0 (0.0) | 0 (0.0) | 0 (0.0) | 0 (0.0) | 0 (0.0) | 0 (0.0) | 0 (0.0) | 0 (0.0) |
| **Time since last vaccine dose (days)** |  |  |  |  |  |  |  |  |  |
| Mean (SD) | 245.5 (85.7) | 238.5 (95.0) | 249.6 (79.7) | 223.2 (85.2) | 220.5 (100.5) | 225.4 (70.8) | 260.7 (82.9) | 256.0 (86.5) | 262.9 (81.3) |
| Median  (IQR) | 251.5  (202.8, 288.0) | 251.5  (179.2, 282.8) | 251.5  (211.2, 288.0) | 229.5  (178.2, 268.8) | 238.5  (156.8, 273.0) | 227.5  (191.5, 265.2) | 259.5  (220.5, 297.0) | 257.0  (206.0, 298.0) | 264.0  (228.2, 296.5) |
| Range | 1.0 - 529.0 | 1.0 - 522.0 | 1.0 - 529.0 | 1.0 - 494.0 | 1.0 - 494.0 | 39.0 - 406.0 | 1.0 - 529.0 | 47.0 - 522.0 | 1.0 - 529.0 |
| **Time since last vaccine dose, n (%)** |  |  |  |  |  |  |  |  |  |
| <2 months | 12 (3.3) | 6 (4.5) | 6 (2.7) | 8 (5.5) | 5 (7.6) | 3 (3.8) | 4 (1.9) | 1 (1.5) | 3 (2.1) |
| [2 - 4) months | 14 (3.9) | 8 (6.0) | 6 (2.7) | 8 (5.5) | 5 (7.6) | 3 (3.8) | 6 (2.8) | 3 (4.4) | 3 (2.1) |
| [4 - 6) months | 39 (10.8) | 20 (14.9) | 19 (8.4) | 22 (15.1) | 12 (18.2) | 10 (12.5) | 17 (7.9) | 8 (11.8) | 9 (6.2) |
| [6 - 8) months | 86 (23.9) | 23 (17.2) | 63 (27.9) | 41 (28.1) | 11 (16.7) | 30 (37.5) | 45 (21.0) | 12 (17.6) | 33 (22.6) |
| ≥8 months | 209 (58.1) | 77 (57.5) | 132 (58.4) | 67 (45.9) | 33 (50.0) | 34 (42.5) | 142 (66.4) | 44 (64.7) | 98 (67.1) |
| **SYMPTOMS** | | | | | | | | | |
| **SARI symptoms ^d,e^, n (%)** |  |  |  |  |  |  |  |  |  |
| Cough | 252 (65.6) | 83 (58.5) | 169 (69.8) | 105 (66.5) | 39 (55.7) | 66 (75.0) | 147 (65.0) | 44 (61.1) | 103 (66.9) |
| Fever | 240 (62.3) | 91 (64.1) | 149 (61.3) | 106 (66.7) | 48 (68.6) | 58 (65.2) | 134 (59.3) | 43 (59.7) | 91 (59.1) |
| Shortness of breath | 273 (70.9) | 95 (66.9) | 178 (73.3) | 110 (69.2) | 45 (64.3) | 65 (73.0) | 163 (72.1) | 50 (69.4) | 113 (73.4) |
| Anosmia, ageusia or dysgeusia | 15 (4.1) | 3 (2.2) | 12 (5.3) | 13 (8.6) | 2 (2.9) | 11 (13.4) | 2 (0.9) | 1 (1.4) | 1 (0.7) |
| **Number of SARI symptoms, n (%)** |  |  |  |  |  |  |  |  |  |
| 1 | 99 (27.2) | 44 (32.1) | 55 (24.2) | 45 (30.2) | 23 (34.3) | 22 (26.8) | 54 (25.1) | 21 (30.0) | 33 (22.8) |
| 2 | 173 (47.5) | 65 (47.4) | 108 (47.6) | 57 (38.3) | 31 (46.3) | 26 (31.7) | 116 (54.0) | 34 (48.6) | 82 (56.6) |
| 3+ | 92 (25.3) | 28 (20.4) | 64 (28.2) | 47 (31.5) | 13 (19.4) | 34 (41.5) | 45 (20.9) | 15 (21.4) | 30 (20.7) |
| **HOSPITAL OUTCOMES** | | | | | | | | | |
| **Length of stay (days)** |  |  |  |  |  |  |  |  |  |
| Mean (SD) | 10.4 (12.3) | 12.0 (13.7) | 9.5 (11.2) | 12.4 (16.0) | 13.3 (17.2) | 11.6 (15.1) | 9.0 (8.5) | 10.6 (9.1) | 8.2 (8.1) |
| Median  (IQR) | 7.0  (4.0, 12.0) | 7.0  (4.0, 12.5) | 6.0  (3.0, 11.2) | 7.0  (4.0, 14.2) | 7.0  (4.0, 18.5) | 7.0  (4.0, 13.0) | 7.0  (3.0, 11.0) | 9.0  (5.0, 12.0) | 6.0  (3.0, 11.0) |
| Range | 1.0 - 98.0 | 1.0 - 98.0 | 1.0 - 83.0 | 1.0 - 98.0 | 1.0 - 98.0 | 1.0 - 83.0 | 1.0 - 57.0 | 1.0 - 46.0 | 1.0 - 57.0 |
| **Severe outcomes, n (%)** |  |  |  |  |  |  |  |  |  |
| Hospitalization without ICU admission or in-hospital death | 344 (88.9) | 125 (87.4) | 219 (89.8) | 136 (85.0) | 59 (83.1) | 77 (86.5) | 208 (91.6) | 66 (91.7) | 142 (91.6) |
| ICU admission without in-hospital death | 16 (4.1) | 3 (2.1) | 13 (5.3) | 7 (4.4) | 1 (1.4) | 6 (6.7) | 9 (4.0) | 2 (2.8) | 7 (4.5) |
| ICU admission (with or without in-hospital death) | 25 (6.5) | 8 (5.6) | 17 (7.0) | 14 (8.8) | 6 (8.5) | 8 (9.0) | 11 (4.8) | 2 (2.8) | 9 (5.8) |
| In-hospital death | 27 (7.0) | 15 (10.5) | 12 (4.9) | 17 (10.6) | 11 (15.5) | 6 (6.7) | 10 (4.4) | 4 (5.6) | 6 (3.9) |
| **COMORBIDITIES** | | | | | | | | | |
| **Number of comorbidities other than IC, n (%)** |  |  |  |  |  |  |  |  |  |
| No comorbid conditions | 56 (14.5) | 9 (6.3) | 47 (19.3) | 14 (8.8) | 2 (2.8) | 12 (13.5) | 42 (18.5) | 7 (9.7) | 35 (22.6) |
| 1 comorbidity | 91 (23.5) | 33 (23.1) | 58 (23.8) | 34 (21.2) | 15 (21.1) | 19 (21.3) | 57 (25.1) | 18 (25.0) | 39 (25.2) |
| 2 comorbidities | 97 (25.1) | 36 (25.2) | 61 (25.0) | 49 (30.6) | 22 (31.0) | 27 (30.3) | 48 (21.1) | 14 (19.4) | 34 (21.9) |
| 3+ comorbidities | 143 (37.0) | 65 (45.5) | 78 (32.0) | 63 (39.4) | 32 (45.1) | 31 (34.8) | 80 (35.2) | 33 (45.8) | 47 (30.3) |
| **Number with specific comorbidities ^d^, n (%)** |  |  |  |  |  |  |  |  |  |
| Asthma | 28 (7.2) | 7 (4.9) | 21 (8.6) | 6 (3.8) | 1 (1.4) | 5 (5.6) | 22 (9.7) | 6 (8.3) | 16 (10.3) |
| Lung disease | 152 (39.3) | 63 (44.1) | 89 (36.5) | 65 (40.6) | 31 (43.7) | 34 (38.2) | 87 (38.3) | 32 (44.4) | 55 (35.5) |
| Cardiovascular disease | 150 (38.8) | 51 (35.7) | 99 (40.6) | 70 (43.8) | 28 (39.4) | 42 (47.2) | 80 (35.2) | 23 (31.9) | 57 (36.8) |
| Hypertension | 182 (47.0) | 54 (37.8) | 128 (52.5) | 81 (50.6) | 28 (39.4) | 53 (59.6) | 101 (44.5) | 26 (36.1) | 75 (48.4) |
| Chronic liver disease | 22 (5.7) | 13 (9.1) | 9 (3.7) | 11 (6.9) | 6 (8.5) | 5 (5.6) | 11 (4.8) | 7 (9.7) | 4 (2.6) |
| Chronic kidney disease | 73 (18.9) | 27 (18.9) | 46 (18.9) | 32 (20.0) | 11 (15.5) | 21 (23.6) | 41 (18.1) | 16 (22.2) | 25 (16.1) |
| Type 2 diabetes | 103 (26.6) | 37 (25.9) | 66 (27.0) | 50 (31.2) | 22 (31.0) | 28 (31.5) | 53 (23.3) | 15 (20.8) | 38 (24.5) |
| Cancer | 106 (27.4) | 106 (74.1) | 0 (0.0) | 56 (35.0) | 56 (78.9) | 0 (0.0) | 50 (22.0) | 50 (69.4) | 0 (0.0) |
| Main IC ^h^ | 85 (22.0) | 85 (59.4) | 0 (0.0) | 38 (23.8) | 38 (53.5) | 0 (0.0) | 47 (20.7) | 47 (65.3) | 0 (0.0) |
| **Q4 2022** |  |  |  |  |  |  |  |  |  |
| **Total** | **764** | **225** | **539** | **179** | **63** | **116** | **585** | **162** | **423** |
| **DEMOGRAPHICS** | | | | | | | | | |
| **Age (years)** |  |  |  |  |  |  |  |  |  |
| Mean (SD) | 66.6 (17.4) | 64.6 (16.4) | 67.4 (17.8) | 69.0 (15.9) | 66.1 (14.5) | 70.5 (16.4) | 65.9 (17.8) | 64.0 (17.1) | 66.6 (18.0) |
| Median  (IQR) | 69.5  (56.0, 79.0) | 66.0  (55.0, 78.0) | 71.0  (57.0, 81.0) | 72.0  (60.0, 80.0) | 67.0  (59.0, 77.0) | 74.0  (62.8, 82.2) | 68.0  (55.0, 79.0) | 66.0  (55.0, 78.0) | 69.0  (56.0, 80.0) |
| Range | 18.0 - 100.0 | 18.0 - 94.0 | 18.0 - 100.0 | 23.0 - 94.0 | 23.0 - 90.0 | 24.0 - 94.0 | 18.0 - 100.0 | 18.0 - 94.0 | 18.0 - 100.0 |
| **Sex, n (%)** |  |  |  |  |  |  |  |  |  |
| Male | 409 (53.5) | 134 (59.6) | 275 (51.0) | 104 (58.1) | 39 (61.9) | 65 (56.0) | 305 (52.1) | 95 (58.6) | 210 (49.6) |
| Female | 355 (46.5) | 91 (40.4) | 264 (49.0) | 75 (41.9) | 24 (38.1) | 51 (44.0) | 280 (47.9) | 67 (41.4) | 213 (50.4) |
| **Country, n (%)** |  |  |  |  |  |  |  |  |  |
| Belgium | 74 (9.7) | 12 (5.3) | 62 (11.5) | 13 (7.3) | 3 (4.8) | 10 (8.6) | 61 (10.4) | 9 (5.6) | 52 (12.3) |
| Italy | 170 (22.3) | 44 (19.6) | 126 (23.4) | 49 (27.4) | 13 (20.6) | 36 (31.0) | 121 (20.7) | 31 (19.1) | 90 (21.3) |
| Spain | 520 (68.1) | 169 (75.1) | 351 (65.1) | 117 (65.4) | 47 (74.6) | 70 (60.3) | 403 (68.9) | 122 (75.3) | 281 (66.4) |
| **Study site, n (%)** |  |  |  |  |  |  |  |  |  |
| CIRI-IT | 170 (22.3) | 44 (19.6) | 126 (23.4) | 49 (27.4) | 13 (20.6) | 36 (31.0) | 121 (20.7) | 31 (19.1) | 90 (21.3) |
| GTPUH | 125 (16.4) | 30 (13.3) | 95 (17.6) | 21 (11.7) | 3 (4.8) | 18 (15.5) | 104 (17.8) | 27 (16.7) | 77 (18.2) |
| CHU Saint-Pierre | 44 (5.8) | 6 (2.7) | 38 (7.1) | 8 (4.5) | 1 (1.6) | 7 (6.0) | 36 (6.2) | 5 (3.1) | 31 (7.3) |
| UZA | 30 (3.9) | 6 (2.7) | 24 (4.5) | 5 (2.8) | 2 (3.2) | 3 (2.6) | 25 (4.3) | 4 (2.5) | 21 (5.0) |
| HUVH | 395 (51.7) | 139 (61.8) | 256 (47.5) | 96 (53.6) | 44 (69.8) | 52 (44.8) | 299 (51.1) | 95 (58.6) | 204 (48.2) |
| **Smoking, n (%)** |  |  |  |  |  |  |  |  |  |
| Never smoker | 310 (40.6) | 87 (38.7) | 223 (41.4) | 72 (40.2) | 28 (44.4) | 44 (37.9) | 238 (40.7) | 59 (36.4) | 179 (42.3) |
| Former smoker | 197 (25.8) | 64 (28.4) | 133 (24.7) | 48 (26.8) | 12 (19.0) | 36 (31.0) | 149 (25.5) | 52 (32.1) | 97 (22.9) |
| Current smoker | 206 (27.0) | 58 (25.8) | 148 (27.5) | 43 (24.0) | 15 (23.8) | 28 (24.1) | 163 (27.9) | 43 (26.5) | 120 (28.4) |
| Missing | 51 (6.7) | 16 (7.1) | 35 (6.5) | 16 (8.9) | 8 (12.7) | 8 (6.9) | 35 (6.0) | 8 (4.9) | 27 (6.4) |
| **Long-term care facility residence, n (%)** |  |  |  |  |  |  |  |  |  |
| Yes | 22 (2.9) | 1 (0.5) | 21 (4.0) | 5 (2.8) | 0 (0.0) | 5 (4.4) | 17 (3.0) | 1 (0.6) | 16 (3.9) |
| **VACCINATION STATUS AT TIME OF HOSPITAL ADMISSION** | | | | | | | | | |
| Unvaccinated, n (%) | 51 (6.7) | 11 (4.9) | 40 (7.4) | 11 (6.1) | 4 (6.3) | 7 (6.0) | 40 (6.8) | 7 (4.3) | 33 (7.8) |
| Incomplete primary series, n (%) | 7 (0.9) | 1 (0.4) | 6 (1.1) | 0 (0.0) | 0 (0.0) | 0 (0.0) | 7 (1.2) | 1 (0.6) | 6 (1.4) |
| Primary series completed but no boosters, n (%) | 92 (12.0) | 29 (12.9) | 63 (11.7) | 21 (11.7) | 6 (9.5) | 15 (12.9) | 71 (12.1) | 23 (14.2) | 48 (11.3) |
| At least one booster dose, n (%) | 614 (80.4) | 184 (81.8) | 430 (79.8) | 147 (82.1) | 53 (84.1) | 94 (81.0) | 467 (79.8) | 131 (80.9) | 336 (79.4) |
| **Number of booster doses ^c^, n (%)** |  |  |  |  |  |  |  |  |  |
| 0 | 92 (13.0) | 29 (13.6) | 63 (12.8) | 21 (12.5) | 6 (10.2) | 15 (13.8) | 71 (13.2) | 23 (14.9) | 48 (12.5) |
| 1 | 346 (49.0) | 100 (46.9) | 246 (49.9) | 76 (45.2) | 24 (40.7) | 52 (47.7) | 270 (50.2) | 76 (49.4) | 194 (50.5) |
| 2 | 251 (35.6) | 72 (33.8) | 179 (36.3) | 62 (36.9) | 21 (35.6) | 41 (37.6) | 189 (35.1) | 51 (33.1) | 138 (35.9) |
| 3+ | 17 (2.4) | 12 (5.6) | 5 (1.0) | 9 (5.4) | 8 (13.6) | 1 (0.9) | 8 (1.5) | 4 (2.6) | 4 (1.0) |
| **Time since last vaccine dose (days)** |  |  |  |  |  |  |  |  |  |
| Mean (SD) | 253.5 (154.4) | 265.6 (149.4) | 248.3 (156.3) | 264.9 (150.7) | 270.2 (129.1) | 262.0 (161.7) | 250.0 (155.4) | 263.8 (156.8) | 244.5 (154.7) |
| Median  (IQR) | 296.0  (77.0, 364.0) | 286.0  (152.0, 366.8) | 304.0  (67.5, 362.5) | 301.0  (135.8, 366.0) | 259.0  (205.5, 358.5) | 319.0  (72.0, 367.0) | 295.0  (73.0, 361.0) | 291.0  (122.5, 368.5) | 297.5  (66.2, 359.5) |
| Range | 2.0 - 606.0 | 2.0 - 606.0 | 2.0 - 576.0 | 2.0 - 575.0 | 23.0 - 557.0 | 2.0 - 575.0 | 2.0 - 606.0 | 2.0 - 606.0 | 2.0 - 576.0 |
| **Time since last vaccine dose, n (%)** |  |  |  |  |  |  |  |  |  |
| <2 months | 145 (20.3) | 32 (15.0) | 113 (22.6) | 27 (16.1) | 5 (8.5) | 22 (20.2) | 118 (21.7) | 27 (17.4) | 91 (23.3) |
| [2 - 4) months | 59 (8.3) | 14 (6.5) | 45 (9.0) | 14 (8.3) | 3 (5.1) | 11 (10.1) | 45 (8.3) | 11 (7.1) | 34 (8.7) |
| [4 - 6) months | 34 (4.8) | 16 (7.5) | 18 (3.6) | 8 (4.8) | 5 (8.5) | 3 (2.8) | 26 (4.8) | 11 (7.1) | 15 (3.8) |
| [6 - 8) months | 40 (5.6) | 23 (10.7) | 17 (3.4) | 16 (9.5) | 12 (20.3) | 4 (3.7) | 24 (4.4) | 11 (7.1) | 13 (3.3) |
| ≥8 months | 435 (61.0) | 129 (60.3) | 306 (61.3) | 103 (61.3) | 34 (57.6) | 69 (63.3) | 332 (60.9) | 95 (61.3) | 237 (60.8) |
| **SYMPTOMS** | | | | | | | | | |
| **SARI symptoms ^d,e^, n (%)** |  |  |  |  |  |  |  |  |  |
| Cough | 629 (82.3) | 177 (78.7) | 452 (83.9) | 140 (78.2) | 44 (69.8) | 96 (82.8) | 489 (83.6) | 133 (82.1) | 356 (84.2) |
| Fever | 485 (64.2) | 145 (64.7) | 340 (63.9) | 116 (64.8) | 40 (63.5) | 76 (65.5) | 369 (64.0) | 105 (65.2) | 264 (63.5) |
| Shortness of breath | 519 (68.3) | 143 (63.6) | 376 (70.3) | 125 (69.8) | 40 (63.5) | 85 (73.3) | 394 (67.8) | 103 (63.6) | 291 (69.5) |
| Anosmia, ageusia or dysgeusia | 9 (1.2) | 5 (2.3) | 4 (0.8) | 6 (3.4) | 3 (4.9) | 3 (2.7) | 3 (0.5) | 2 (1.3) | 1 (0.2) |
| **Number of SARI symptoms, n (%)** |  |  |  |  |  |  |  |  |  |
| 1 | 105 (14.4) | 42 (19.2) | 63 (12.3) | 31 (17.8) | 16 (26.2) | 15 (13.3) | 74 (13.3) | 26 (16.5) | 48 (12.0) |
| 2 | 414 (56.6) | 117 (53.4) | 297 (58.0) | 88 (50.6) | 29 (47.5) | 59 (52.2) | 326 (58.5) | 88 (55.7) | 238 (59.6) |
| 3+ | 212 (29.0) | 60 (27.4) | 152 (29.7) | 55 (31.6) | 16 (26.2) | 39 (34.5) | 157 (28.2) | 44 (27.8) | 113 (28.3) |
| **HOSPITAL OUTCOMES** | | | | | | | | | |
| **Length of stay (days)** |  |  |  |  |  |  |  |  |  |
| Mean (SD) | 9.7 (14.8) | 10.4 (14.3) | 9.5 (15.1) | 10.8 (16.9) | 10.1 (8.7) | 11.2 (20.0) | 9.4 (14.1) | 10.5 (16.0) | 9.0 (13.4) |
| Median  (IQR) | 6.0  (4.0, 11.0) | 7.0  (4.0, 11.0) | 6.0  (4.0, 11.0) | 8.0  (4.0, 13.0) | 7.0  (4.5, 14.0) | 8.0  (4.0, 12.2) | 6.0  (4.0, 10.0) | 6.5  (4.0, 10.0) | 6.0  (4.0, 10.0) |
| Range | 1.0 - 201.0 | 1.0 - 173.0 | 1.0 - 201.0 | 1.0 - 201.0 | 1.0 - 52.0 | 1.0 - 201.0 | 1.0 - 173.0 | 1.0 - 173.0 | 1.0 - 166.0 |
| **Severe outcomes, n (%)** |  |  |  |  |  |  |  |  |  |
| Hospitalization without ICU admission or in-hospital death | 691 (90.4) | 195 (86.7) | 496 (92.0) | 151 (84.4) | 50 (79.4) | 101 (87.1) | 540 (92.3) | 145 (89.5) | 395 (93.4) |
| ICU admission without in-hospital death | 36 (4.7) | 9 (4.0) | 27 (5.0) | 10 (5.6) | 4 (6.3) | 6 (5.2) | 26 (4.4) | 5 (3.1) | 21 (5.0) |
| ICU admission (with or without in-hospital death) | 45 (5.9) | 17 (7.6) | 28 (5.2) | 14 (7.8) | 7 (11.1) | 7 (6.0) | 31 (5.3) | 10 (6.2) | 21 (5.0) |
| In-hospital death | 37 (4.8) | 21 (9.3) | 16 (3.0) | 18 (10.1) | 9 (14.3) | 9 (7.8) | 19 (3.2) | 12 (7.4) | 7 (1.7) |
| **COMORBIDITIES** | | | | | | | | | |
| **Number of comorbidities other than IC, n (%)** |  |  |  |  |  |  |  |  |  |
| No comorbid conditions | 125 (16.4) | 11 (4.9) | 114 (21.2) | 15 (8.4) | 2 (3.2) | 13 (11.2) | 110 (18.8) | 9 (5.6) | 101 (23.9) |
| 1 comorbidity | 166 (21.7) | 48 (21.3) | 118 (21.9) | 36 (20.1) | 13 (20.6) | 23 (19.8) | 130 (22.2) | 35 (21.6) | 95 (22.5) |
| 2 comorbidities | 177 (23.2) | 45 (20.0) | 132 (24.5) | 58 (32.4) | 16 (25.4) | 42 (36.2) | 119 (20.3) | 29 (17.9) | 90 (21.3) |
| 3+ comorbidities | 296 (38.7) | 121 (53.8) | 175 (32.5) | 70 (39.1) | 32 (50.8) | 38 (32.8) | 226 (38.6) | 89 (54.9) | 137 (32.4) |
| **Number with specific comorbidities ^d^, n (%)** |  |  |  |  |  |  |  |  |  |
| Asthma | 71 (9.3) | 15 (6.7) | 56 (10.4) | 13 (7.3) | 2 (3.2) | 11 (9.5) | 58 (9.9) | 13 (8.0) | 45 (10.6) |
| Lung disease | 289 (37.8) | 94 (41.8) | 195 (36.2) | 68 (38.0) | 22 (34.9) | 46 (39.7) | 221 (37.8) | 72 (44.4) | 149 (35.2) |
| Cardiovascular disease | 313 (41.0) | 96 (42.7) | 217 (40.3) | 75 (41.9) | 24 (38.1) | 51 (44.0) | 238 (40.7) | 72 (44.4) | 166 (39.2) |
| Hypertension | 372 (48.7) | 107 (47.6) | 265 (49.2) | 104 (58.1) | 33 (52.4) | 71 (61.2) | 268 (45.8) | 74 (45.7) | 194 (45.9) |
| Chronic liver disease | 71 (9.3) | 30 (13.3) | 41 (7.6) | 15 (8.4) | 7 (11.1) | 8 (6.9) | 56 (9.6) | 23 (14.2) | 33 (7.8) |
| Chronic kidney disease | 132 (17.3) | 59 (26.2) | 73 (13.5) | 43 (24.0) | 24 (38.1) | 19 (16.4) | 89 (15.2) | 35 (21.6) | 54 (12.8) |
| Type 2 diabetes | 204 (26.7) | 66 (29.3) | 138 (25.6) | 48 (26.8) | 18 (28.6) | 30 (25.9) | 156 (26.7) | 48 (29.6) | 108 (25.5) |
| Cancer | 159 (20.8) | 159 (70.7) | 0 (0.0) | 45 (25.1) | 45 (71.4) | 0 (0.0) | 114 (19.5) | 114 (70.4) | 0 (0.0) |
| Main IC ^h^ | 120 (15.7) | 120 (53.3) | 0 (0.0) | 36 (20.1) | 36 (57.1) | 0 (0.0) | 84 (14.4) | 84 (51.9) | 0 (0.0) |
| **Q1 2023** |  |  |  |  |  |  |  |  |  |
| **Total** | **710** | **204** | **506** | **116** | **39** | **77** | **594** | **165** | **210** |
| **DEMOGRAPHICS** | | | | | | | | | |
| **Age (years)** |  |  |  |  |  |  |  |  |  |
| Mean (SD) | 68.7 (16.5) | 68.5 (14.8) | 68.8 (17.1) | 74.2 (13.4) | 74.6 (11.2) | 74.0 (14.5) | 67.7 (16.8) | 67.1 (15.2) | 67.9 (17.4) |
| Median  (IQR) | 71.5 (  58.0, 82.0) | 69.5  (59.0, 80.0) | 72.0  (58.0, 82.0) | 76.5  (67.0, 84.0) | 76.0  (66.5, 83.0) | 78.0  (68.0, 85.0) | 70.0  (57.0, 81.0) | 68.0  (58.0, 78.0) | 71.0  (57.0, 82.0) |
| Range | 18.0 - 99.0 | 20.0 - 99.0 | 18.0 - 97.0 | 23.0 - 98.0 | 51.0 - 98.0 | 23.0 - 92.0 | 18.0 - 99.0 | 20.0 - 99.0 | 18.0 - 97.0 |
| **Sex, n (%)** |  |  |  |  |  |  |  |  |  |
| Male | 380 (53.5) | 122 (59.8) | 258 (51.0) | 65 (56.0) | 26 (66.7) | 39 (50.6) | 315 (53.0) | 96 (58.2) | 219 (51.0) |
| Female | 330 (46.5) | 82 (40.2) | 248 (49.0) | 51 (44.0) | 13 (33.3) | 38 (49.4) | 279 (47.0) | 69 (41.8) | 210 (49.0) |
| **Country, n (%)** |  |  |  |  |  |  |  |  |  |
| Belgium | 57 (8.0) | 17 (8.3) | 40 (7.9) | 10 (8.6) | 3 (7.7) | 7 (9.1) | 47 (7.9) | 14 (8.5) | 33 (7.7) |
| Italy | 193 (27.2) | 41 (20.1) | 152 (30.0) | 32 (27.6) | 6 (15.4) | 26 (33.8) | 161 (27.1) | 35 (21.2) | 126 (29.4) |
| Spain | 460 (64.8) | 146 (71.6) | 314 (62.1) | 74 (63.8) | 30 (76.9) | 44 (57.1) | 386 (65.0) | 116 (70.3) | 270 (62.9) |
| **Study site, n (%)** |  |  |  |  |  |  |  |  |  |
| CIRI-IT | 193 (27.2) | 41 (20.1) | 152 (30.0) | 32 (27.6) | 6 (15.4) | 26 (33.8) | 161 (27.1) | 35 (21.2) | 126 (29.4) |
| GTPUH | 127 (17.9) | 34 (16.7) | 93 (18.4) | 15 (12.9) | 5 (12.8) | 10 (13.0) | 112 (18.9) | 29 (17.6) | 83 (19.3) |
| CHU Saint-Pierre | 25 (3.5) | 7 (3.4) | 18 (3.6) | 3 (2.6) | 0 (0.0) | 3 (3.9) | 22 (3.7) | 7 (4.2) | 15 (3.5) |
| UZA | 32 (4.5) | 10 (4.9) | 22 (4.3) | 7 (6.0) | 3 (7.7) | 4 (5.2) | 25 (4.2) | 7 (4.2) | 18 (4.2) |
| HUVH | 333 (46.9) | 112 (54.9) | 221 (43.7) | 59 (50.9) | 25 (64.1) | 34 (44.2) | 274 (46.1) | 87 (52.7) | 187 (43.6) |
| **Smoking, n (%)** |  |  |  |  |  |  |  |  |  |
| Never smoker | 307 (43.2) | 89 (43.6) | 218 (43.1) | 57 (49.1) | 20 (51.3) | 37 (48.1) | 250 (42.1) | 69 (41.8) | 181 (42.2) |
| Former smoker | 210 (29.6) | 75 (36.8) | 135 (26.7) | 37 (31.9) | 12 (30.8) | 25 (32.5) | 173 (29.1) | 63 (38.2) | 110 (25.6) |
| Current smoker | 159 (22.4) | 26 (12.7) | 133 (26.3) | 17 (14.7) | 5 (12.8) | 12 (15.6) | 142 (23.9) | 21 (12.7) | 121 (28.2) |
| Missing | 34 (4.8) | 14 (6.9) | 20 (4.0) | 5 (4.3) | 2 (5.1) | 3 (3.9) | 29 (4.9) | 12 (7.3) | 17 (4.0) |
| **Long-term care facility residence, n (%)** |  |  |  |  |  |  |  |  |  |
| Yes | 23 (3.3) | 7 (3.5) | 16 (3.2) | 3 (2.7) | 0 (0.0) | 3 (3.9) | 20 (3.4) | 7 (4.3) | 13 (3.1) |
| **VACCINATION STATUS AT TIME OF HOSPITAL ADMISSION** | | | | | | | | | |
| Unvaccinated, n (%) | 45 (6.3) | 8 (3.9) | 37 (7.3) | 9 (7.8) | 0 (0.0) | 9 (11.7) | 36 (6.1) | 8 (4.8) | 28 (6.5) |
| Incomplete primary series, n (%) | 11 (1.5) | 3 (1.5) | 8 (1.6) | 1 (0.9) | 0 (0.0) | 1 (1.3) | 10 (1.7) | 3 (1.8) | 7 (1.6) |
| Primary series completed but no boosters, n (%) | 67 (9.4) | 19 (9.3) | 48 (9.5) | 9 (7.8) | 3 (7.7) | 6 (7.8) | 58 (9.8) | 16 (9.7) | 42 (9.8) |
| At least one booster dose, n (%) | 587 (82.7) | 174 (85.3) | 413 (81.6) | 97 (83.6) | 36 (92.3) | 61 (79.2) | 490 (82.5) | 138 (83.6) | 352 (82.1) |
| **Number of booster doses ^c^, n (%)** |  |  |  |  |  |  |  |  |  |
| 0 | 67 (10.2) | 19 (9.8) | 48 (10.4) | 9 (8.5) | 3 (7.7) | 6 (9.0) | 58 (10.6) | 16 (10.4) | 42 (10.7) |
| 1 | 253 (38.7) | 63 (32.6) | 190 (41.2) | 31 (29.2) | 9 (23.1) | 22 (32.8) | 222 (40.5) | 54 (35.1) | 168 (42.6) |
| 2 | 311 (47.6) | 94 (48.7) | 217 (47.1) | 59 (55.7) | 20 (51.3) | 39 (58.2) | 252 (46.0) | 74 (48.1) | 178 (45.2) |
| 3+ | 23 (3.5) | 17 (8.8) | 6 (1.3) | 7 (6.6) | 7 (17.9) | 0 (0.0) | 16 (2.9) | 10 (6.5) | 6 (1.5) |
| **Time since last vaccine dose (days)** |  |  |  |  |  |  |  |  |  |
| Mean (SD) | 288.8 (171.7) | 289.1 (164.9) | 288.7 (174.7) | 274.8 (164.3) | 292.2 (163.5) | 264.8 (165.1) | 291.5 (173.1) | 288.4 (165.7) | 292.7 (176.1) |
| Median  (IQR) | 296.0  (125.0, 431.0) | 296.0  (134.0, 431.0) | 314.0  (123.0, 427.0) | 230.0  (129.0, 425.5) | 285.0  (153.5, 426.0) | 195.0  (126.5, 423.5) | 318.5  (125.0, 431.8) | 297.0  (131.0, 431.0) | 334.0  (123.0, 434.0) |
| Range | 10.0 - 731.0 | 35.0 - 662.0 | 10.0 - 731.0 | 42.0 - 662.0 | 46.0 - 662.0 | 42.0 - 653.0 | 10.0 - 731.0 | 35.0 - 652.0 | 10.0 - 731.0 |
| **Time since last vaccine dose, n (%)** |  |  |  |  |  |  |  |  |  |
| <2 months | 19 (2.9) | 5 (2.6) | 14 (3.0) | 2 (1.9) | 1 (2.6) | 1 (1.5) | 17 (3.0) | 4 (2.5) | 13 (3.2) |
| [2 - 4) months | 127 (19.1) | 34 (17.3) | 93 (19.8) | 19 (17.8) | 5 (12.8) | 14 (20.6) | 108 (19.4) | 29 (18.5) | 79 (19.7) |
| [4 - 6) months | 139 (20.9) | 39 (19.9) | 100 (21.3) | 28 (26.2) | 9 (23.1) | 19 (27.9) | 111 (19.9) | 30 (19.1) | 81 (20.2) |
| [6 - 8) months | 23 (3.5) | 9 (4.6) | 14 (3.0) | 5 (4.7) | 2 (5.1) | 3 (4.4) | 18 (3.2) | 7 (4.5) | 11 (2.7) |
| ≥8 months | 357 (53.7) | 109 (55.6) | 248 (52.9) | 53 (49.5) | 22 (56.4) | 31 (45.6) | 304 (54.5) | 87 (55.4) | 217 (54.1) |
| **SYMPTOMS** | | | | | | | | | |
| **SARI symptoms ^d,e^, n (%)** |  |  |  |  |  |  |  |  |  |
| Cough | 600 (84.5) | 168 (82.4) | 432 (85.4) | 93 (80.2) | 29 (74.4) | 64 (83.1) | 507 (85.4) | 139 (84.2) | 368 (85.8) |
| Fever | 464 (66.3) | 146 (72.3) | 318 (63.9) | 77 (67.5) | 27 (69.2) | 50 (66.7) | 387 (66.0) | 119 (73.0) | 268 (63.4) |
| Shortness of breath | 454 (64.0) | 122 (60.1) | 332 (65.6) | 68 (58.6) | 21 (53.8) | 47 (61.0) | 386 (65.1) | 101 (61.6) | 285 (66.4) |
| Anosmia, ageusia or dysgeusia | 11 (1.6) | 5 (2.6) | 6 (1.2) | 8 (7.3) | 2 (5.6) | 6 (8.2) | 3 (0.5) | 3 (1.9) | 0 (0.0) |
| **Number of SARI symptoms, n (%)** |  |  |  |  |  |  |  |  |  |
| 1 | 95 (14.1) | 29 (15.0) | 66 (13.8) | 23 (21.1) | 9 (25.0) | 14 (19.2) | 72 (12.8) | 20 (12.7) | 52 (12.8) |
| 2 | 381 (56.7) | 106 (54.9) | 275 (57.4) | 53 (48.6) | 19 (52.8) | 34 (46.6) | 328 (58.3) | 87 (55.4) | 241 (59.4) |
| 3+ | 196 (29.2) | 58 (30.1) | 138 (28.8) | 33 (30.3) | 8 (22.2) | 25 (34.2) | 163 (29.0) | 50 (31.8) | 113 (27.8) |
| **HOSPITAL OUTCOMES** | | | | | | | | | |
| **Length of stay (days)** |  |  |  |  |  |  |  |  |  |
| Mean (SD) | 9.0 (11.1) | 8.5 (9.8) | 9.1 (11.5) | 10.3 (12.9) | 8.6 (10.9) | 11.2 (13.8) | 8.7 (10.6) | 8.5 (9.5) | 8.8 (11.1) |
| Median  (IQR) | 6.0  (3.0, 10.0) | 5.0  (3.0, 9.0) | 6.0 (3.0, 11.0) | 6.0  (3.0, 12.0) | 5.0  (3.0, 8.0) | 7.0  (3.0, 13.0) | 6.0  (3.0, 10.0) | 5.0  (3.0, 10.0) | 6.0  (3.0, 10.0) |
| Range | 1.0 - 155.0 | 1.0 - 70.0 | 1.0 - 155.0 | 1.0 - 92.0 | 1.0 - 51.0 | 1.0 - 92.0 | 1.0 - 155.0 | 1.0 - 70.0 | 1.0 - 155.0 |
| **Severe outcomes, n (%)** |  |  |  |  |  |  |  |  |  |
| Hospitalization without ICU admission or in-hospital death | 647 (91.1) | 186 (91.2) | 461 (91.1) | 109 (94.0) | 37 (94.9) | 72 (93.5) | 538 (90.6) | 149 (90.3) | 389 (90.7) |
| ICU admission without in-hospital death | 32 (4.5) | 10 (4.9) | 22 (4.3) | 5 (4.3) | 1 (2.6) | 4 (5.2) | 27 (4.5) | 9 (5.5) | 18 (4.2) |
| ICU admission (with or without in-hospital death) | 37 (5.2) | 11 (5.4) | 26 (5.1) | 5 (4.3) | 1 (2.6) | 4 (5.2) | 32 (5.4) | 10 (6.1) | 22 (5.1) |
| In-hospital death | 31 (4.4) | 8 (3.9) | 23 (4.5) | 2 (1.7) | 1 (2.6) | 1 (1.3) | 29 (4.9) | 7 (4.2) | 22 (5.1) |
| **COMORBIDITIES** | | | | | | | | | |
| **Number of comorbidities other than IC, n (%)** |  |  |  |  |  |  |  |  |  |
| No comorbid conditions | 132 (18.6) | 8 (3.9) | 124 (24.5) | 15 (12.9) | 0 (0.0) | 15 (19.5) | 117 (19.7) | 8 (4.8) | 109 (25.4) |
| 1 comorbidity | 133 (18.7) | 30 (14.7) | 103 (20.4) | 22 (19.0) | 7 (17.9) | 15 (19.5) | 111 (18.7) | 23 (13.9) | 88 (20.5) |
| 2 comorbidities | 161 (22.7) | 55 (27.0) | 106 (20.9) | 26 (22.4) | 7 (17.9) | 19 (24.7) | 135 (22.7) | 48 (29.1) | 87 (20.3) |
| 3+ comorbidities | 284 (40.0) | 111 (54.4) | 173 (34.2) | 53 (45.7) | 25 (64.1) | 28 (36.4) | 231 (38.9) | 86 (52.1) | 145 (33.8) |
| **Number with specific comorbidities ^d^, n (%)** |  |  |  |  |  |  |  |  |  |
| Asthma | 61 (8.6) | 13 (6.4) | 48 (9.5) | 3 (2.6) | 0 (0.0) | 3 (3.9) | 58 (9.8) | 13 (7.9) | 45 (10.5) |
| Lung disease | 259 (36.5) | 86 (42.2) | 173 (34.2) | 42 (36.2) | 19 (48.7) | 23 (29.9) | 217 (36.5) | 67 (40.6) | 150 (35.0) |
| Cardiovascular disease | 300 (42.3) | 85 (41.7) | 215 (42.5) | 59 (50.9) | 19 (48.7) | 40 (51.9) | 241 (40.6) | 66 (40.0) | 175 (40.8) |
| Hypertension | 361 (50.8) | 105 (51.5) | 256 (50.6) | 75 (64.7) | 26 (66.7) | 49 (63.6) | 286 (48.1) | 79 (47.9) | 207 (48.3) |
| Chronic liver disease | 63 (8.9) | 23 (11.3) | 40 (7.9) | 6 (5.2) | 2 (5.1) | 4 (5.2) | 57 (9.6) | 21 (12.7) | 36 (8.4) |
| Chronic kidney disease | 105 (14.8) | 41 (20.1) | 64 (12.6) | 25 (21.6) | 11 (28.2) | 14 (18.2) | 80 (13.5) | 30 (18.2) | 50 (11.7) |
| Type 2 diabetes | 189 (26.6) | 49 (24.0) | 140 (27.7) | 30 (25.9) | 11 (28.2) | 19 (24.7) | 159 (26.8) | 38 (23.0) | 121 (28.2) |
| Cancer | 154 (21.7) | 154 (75.5) | 0 (0.0) | 28 (24.1) | 28 (71.8) | 0 (0.0) | 126 (21.2) | 126 (76.4) | 0 (0.0) |
| Main IC ^h^ | 83 (11.7) | 83 (40.7) | 0 (0.0) | 18 (15.5) | 18 (46.2) | 0 (0.0) | 65 (10.9) | 65 (39.4) | 0 (0.0) |
| **Q2 2023** |  |  |  |  |  |  |  |  |  |
| **Total** | **278** | **66** | **171** | **47** | **22** | **25** | **231** | **85** | **146** |
| **DEMOGRAPHICS** | | | | | | | | | |
| **Age (years)** |  |  |  |  |  |  |  |  |  |
| Mean (SD) | 66.0 (16.9) | 68.0 (15.6) | 64.7 (17.6) | 70.4 (15.8) | 69.1 (16.7) | 71.6 (15.2) | 65.1 (17.0) | 67.7 (15.4) | 63.5 (17.8) |
| Median  (IQR) | 68.0  (58.0, 79.0) | 69.0  (60.0, 80.5) | 68.0  (56.0, 78.0) | 73.0  (60.5, 81.5) | 71.0  (54.5, 81.8) | 74.0  (62.0, 81.0) | 68.0  (58.0, 77.0) | 69.0  (60.0, 77.0) | 67.0  (55.0, 75.8) |
| Range | 18.0 - 97.0 | 19.0 - 92.0 | 18.0 - 97.0 | 27.0 - 97.0 | 40.0 - 90.0 | 27.0 - 97.0 | 18.0 - 94.0 | 19.0 - 92.0 | 18.0 - 94.0 |
| **Sex, n (%)** |  |  |  |  |  |  |  |  |  |
| Male | 166 (59.7) | 66 (61.7) | 100 (58.5) | 28 (59.6) | 15 (68.2) | 13 (52.0) | 138 (59.7) | 51 (60.0) | 87 (59.6) |
| Female | 112 (40.3) | 41 (38.3) | 71 (41.5) | 19 (40.4) | 7 (31.8) | 12 (48.0) | 93 (40.3) | 34 (40.0) | 59 (40.4) |
| **Country, n (%)** |  |  |  |  |  |  |  |  |  |
| Belgium | 17 (6.1) | 4 (3.7) | 13 (7.6) | 2 (4.3) | 1 (4.5) | 1 (4.0) | 15 (6.5) | 3 (3.5) | 12 (8.2) |
| Italy | 37 (13.3) | 11 (10.3) | 26 (15.2) | 5 (10.6) | 2 (9.1) | 3 (12.0) | 32 (13.9) | 9 (10.6) | 23 (15.8) |
| Spain | 224 (80.6) | 92 (86.0) | 132 (77.2) | 40 (85.1) | 19 (86.4) | 21 (84.0) | 184 (79.7) | 73 (85.9) | 111 (76.0) |
| **Study site, n (%)** |  |  |  |  |  |  |  |  |  |
| CIRI-IT | 37 (13.3) | 11 (10.3) | 26 (15.2) | 5 (10.6) | 2 (9.1) | 3 (12.0) | 32 (13.9) | 9 (10.6) | 23 (15.8) |
| GTPUH | 49 (17.6) | 18 (16.8) | 31 (18.1) | 6 (12.8) | 3 (13.6) | 3 (12.0) | 43 (18.6) | 15 (17.6) | 28 (19.2) |
| CHU Saint-Pierre | 11 (4.0) | 2 (1.9) | 9 (5.3) | 1 (2.1) | 0 (0.0) | 1 (4.0) | 10 (4.3) | 2 (2.4) | 8 (5.5) |
| UZA | 6 (2.2) | 2 (1.9) | 4 (2.3) | 1 (2.1) | 1 (4.5) | 0 (0.0) | 5 (2.2) | 1 (1.2) | 4 (2.7) |
| HUVH | 175 (62.9) | 74 (69.2) | 101 (59.1) | 34 (72.3) | 16 (72.7) | 18 (72.0) | 141 (61.0) | 58 (68.2) | 83 (56.8) |
| **Smoking, n (%)** |  |  |  |  |  |  |  |  |  |
| Never smoker | 103 (37.1) | 40 (37.4) | 63 (36.8) | 19 (40.4) | 9 (40.9) | 10 (40.0) | 84 (36.4) | 31 (36.5) | 53 (36.3) |
| Former smoker | 92 (33.1) | 41 (38.3) | 51 (29.8) | 18 (38.3) | 8 (36.4) | 10 (40.0) | 74 (32.0) | 33 (38.8) | 41 (28.1) |
| Current smoker | 68 (24.5) | 18 (16.8) | 50 (29.2) | 8 (17.0) | 4 (18.2) | 4 (16.0) | 60 (26.0) | 14 (16.5) | 46 (31.5) |
| Missing | 15 (5.4) | 8 (7.5) | 7 (4.1) | 2 (4.3) | 1 (4.5) | 1 (4.0) | 13 (5.6) | 7 (8.2) | 6 (4.1) |
| **Long-term care facility residence, n (%)** |  |  |  |  |  |  |  |  |  |
| Yes | 11 (4.0) | 4 (3.7) | 7 (4.1) | 3 (6.4) | 1 (4.5) | 2 (8.0) | 8 (3.5) | 3 (3.5) | 5 (3.4) |
| **VACCINATION STATUS AT TIME OF HOSPITAL ADMISSION** | | | | | | | | | |
| Unvaccinated, n (%) | 26 (9.4) | 4 (3.7) | 22 (12.9) | 6 (12.8) | 0 (0.0) | 6 (24.0) | 20 (8.7) | 4 (4.7) | 16 (11.0) |
| Incomplete primary series, n (%) | 3 (1.1) | 0 (0.0) | 3 (1.8) | 1 (2.1) | 0 (0.0) | 1 (4.0) | 2 (0.9) | 0 (0.0) | 2 (1.4) |
| Primary series completed but no boosters, n (%) | 23 (8.3) | 11 (10.3) | 12 (7.0) | 1 (2.1) | 0 (0.0) | 1 (4.0) | 22 (9.5) | 11 (12.9) | 11 (7.5) |
| At least one booster dose, n (%) | 226 (81.3) | 92 (86.0) | 134 (78.4) | 39 (83.0) | 22 (100.0) | 17 (68.0) | 187 (81.0) | 70 (82.4) | 117 (80.1) |
| **Number of booster doses ^c^, n (%)** |  |  |  |  |  |  |  |  |  |
| 0 | 23 (9.2) | 11 (10.7) | 12 (8.2) | 1 (2.5) | 0 (0.0) | 1 (5.6) | 22 (10.5) | 11 (13.6) | 11 (8.6) |
| 1 | 80 (32.1) | 25 (24.3) | 55 (37.7) | 10 (25.0) | 5 (22.7) | 5 (27.8) | 70 (33.5) | 20 (24.7) | 50 (39.1) |
| 2 | 140 (56.2) | 64 (62.1) | 76 (52.1) | 29 (72.5) | 17 (77.3) | 12 (66.7) | 111 (53.1) | 47 (58.0) | 64 (50.0) |
| 3+ | 6 (2.4) | 3 (2.9) | 3 (2.1) | 0 (0.0) | 0 (0.0) | 0 (0.0) | 6 (2.9) | 3 (3.7) | 3 (2.3) |
| **Time since last vaccine dose (days)** |  |  |  |  |  |  |  |  |  |
| Mean (SD) | 314.4 (179.5) | 298.5 (186.1) | 325.3 (174.6) | 273.0 (156.8) | 254.0 (137.7) | 294.9 (177.8) | 322.4 (182.8) | 310.6 (196.2) | 329.7 (174.4) |
| Median  (IQR) | 213.5  (166.0, 487.0) | 200.0  (164.0, 486.5) | 220.0  (173.0, 487.0) | 195.0  (170.0, 438.0) | 187.0  (171.0, 301.5) | 205.0  (169.5, 500.5) | 216.0  (166.0, 489.0) | 205.0  (154.0, 491.0) | 229.5  (173.0, 483.5) |
| Range | 44.0 - 782.0 | 44.0 - 755.0 | 46.0 - 782.0 | 62.0 - 563.0 | 76.0 - 523.0 | 62.0 - 563.0 | 44.0 - 782.0 | 44.0 - 755.0 | 46.0 - 782.0 |
| **Time since last vaccine dose, n (%)** |  |  |  |  |  |  |  |  |  |
| <2 months | 2 (0.8) | 1 (1.0) | 1 (0.7) | 0 (0.0) | 0 (0.0) | 0 (0.0) | 2 (0.9) | 1 (1.2) | 1 (0.8) |
| [2 - 4) months | 13 (5.2) | 6 (5.8) | 7 (4.7) | 3 (7.3) | 1 (4.5) | 2 (10.5) | 10 (4.7) | 5 (6.2) | 5 (3.8) |
| [4 - 6) months | 73 (29.0) | 35 (34.0) | 38 (25.5) | 14 (34.1) | 9 (40.9) | 5 (26.3) | 59 (28.0) | 26 (32.1) | 33 (25.4) |
| [6 - 8) months | 52 (20.6) | 20 (19.4) | 32 (21.5) | 11 (26.8) | 6 (27.3) | 5 (26.3) | 41 (19.4) | 14 (17.3) | 27 (20.8) |
| ≥8 months | 112 (44.4) | 41 (39.8) | 71 (47.7) | 13 (31.7) | 6 (27.3) | 7 (36.8) | 99 (46.9) | 35 (43.2) | 64 (49.2) |
| **SYMPTOMS** | | | | | | | | | |
| **SARI symptoms ^d,e^, n (%)** |  |  |  |  |  |  |  |  |  |
| Cough | 217 (78.6) | 76 (71.7) | 141 (82.9) | 32 (69.6) | 10 (47.6) | 22 (88.0) | 185 (80.4) | 66 (77.6) | 119 (82.1) |
| Fever | 146 (52.9) | 63 (58.9) | 83 (49.1) | 22 (46.8) | 13 (59.1) | 9 (36.0) | 124 (54.1) | 50 (58.8) | 74 (51.4) |
| Shortness of breath | 217 (78.1) | 78 (72.9) | 139 (81.3) | 32 (68.1) | 12 (54.5) | 20 (80.0) | 185 (80.1) | 66 (77.6) | 119 (81.5) |
| Anosmia, ageusia or dysgeusia | 5 (1.8) | 3 (2.9) | 2 (1.2) | 1 (2.2) | 1 (4.8) | 0 (0.0) | 4 (1.8) | 2 (2.4) | 2 (1.4) |
| **Number of SARI symptoms, n (%)** |  |  |  |  |  |  |  |  |  |
| 1 | 43 (15.8) | 19 (18.1) | 24 (14.4) | 15 (32.6) | 9 (42.9) | 6 (24.0) | 28 (12.4) | 10 (11.9) | 18 (12.7) |
| 2 | 158 (58.1) | 62 (59.0) | 96 (57.5) | 23 (50.0) | 11 (52.4) | 12 (48.0) | 135 (59.7) | 51 (60.7) | 84 (59.2) |
| 3+ | 71 (26.1) | 24 (22.9) | 47 (28.1) | 8 (17.4) | 1 (4.8) | 7 (28.0) | 63 (27.9) | 23 (27.4) | 40 (28.2) |
| **HOSPITAL OUTCOMES** | | | | | | | | | |
| **Length of stay (days)** |  |  |  |  |  |  |  |  |  |
| Mean (SD) | 9.1 (10.9) | 9.7 (12.8) | 8.8 (9.6) | 9.5 (11.3) | 7.8 (8.1) | 11.0 (13.5) | 9.0 (10.9) | 10.1 (13.8) | 8.4 (8.7) |
| Median  (IQR) | 6.0  (3.0, 11.0) | 6.0  (4.0, 10.0) | 6.0  (3.0, 11.0) | 7.0  (4.0, 10.0) | 5.5  (4.0, 8.8) | 7.0  (5.0, 13.0) | 6.0  (3.0, 11.0) | 6.0  (4.0, 12.0) | 5.5  (3.0, 11.0) |
| Range | 1.0 - 101.0 | 1.0 - 101.0 | 1.0 - 70.0 | 1.0 - 70.0 | 1.0 - 38.0 | 1.0 - 70.0 | 1.0 - 101.0 | 1.0 - 101.0 | 1.0 - 61.0 |
| **Severe outcomes, n (%)** |  |  |  |  |  |  |  |  |  |
| Hospitalization without ICU admission or in-hospital death | 239 (86.0) | 89 (83.2) | 150 (87.7) | 41 (87.2) | 19 (86.4) | 22 (88.0) | 198 (85.7) | 70 (82.4) | 128 (87.7) |
| ICU admission without in-hospital death | 21 (7.6) | 10 (9.3) | 11 (6.4) | 3 (6.4) | 1 (4.5) | 2 (8.0) | 18 (7.8) | 9 (10.6) | 9 (6.2) |
| ICU admission (with or without in-hospital death) | 24 (8.6) | 10 (9.3) | 14 (8.2) | 4 (8.5) | 1 (4.5) | 3 (12.0) | 20 (8.7) | 9 (10.6) | 11 (7.5) |
| In-hospital death | 18 (6.5) | 8 (7.5) | 10 (5.8) | 3 (6.4) | 2 (9.1) | 1 (4.0) | 15 (6.5) | 6 (7.1) | 9 (6.2) |
| **COMORBIDITIES** | | | | | | | | | |
| **Number of comorbidities other than IC, n (%)** |  |  |  |  |  |  |  |  |  |
| No comorbid conditions | 33 (11.9) | 4 (3.7) | 29 (17.0) | 2 (4.3) | 0 (0.0) | 2 (8.0) | 31 (13.4) | 4 (4.7) | 27 (18.5) |
| 1 comorbidity | 58 (20.9) | 21 (19.6) | 37 (21.6) | 6 (12.8) | 5 (22.7) | 1 (4.0) | 52 (22.5) | 16 (18.8) | 36 (24.7) |
| 2 comorbidities | 62 (22.3) | 14 (13.1) | 48 (28.1) | 19 (40.4) | 4 (18.2) | 15 (60.0) | 43 (18.6) | 10 (11.8) | 33 (22.6) |
| 3+ comorbidities | 125 (45.0) | 68 (63.6) | 57 (33.3) | 20 (42.6) | 13 (59.1) | 7 (28.0) | 105 (45.5) | 55 (64.7) | 50 (34.2) |
| **Number with specific comorbidities ^d^, n (%)** |  |  |  |  |  |  |  |  |  |
| Asthma | 36 (12.9) | 12 (11.2) | 24 (14.0) | 6 (12.8) | 1 (4.5) | 5 (20.0) | 30 (13.0) | 11 (12.9) | 19 (13.0) |
| Lung disease | 121 (43.5) | 45 (42.1) | 76 (44.4) | 17 (36.2) | 6 (27.3) | 11 (44.0) | 104 (45.0) | 39 (45.9) | 65 (44.5) |
| Cardiovascular disease | 131 (47.1) | 52 (48.6) | 79 (46.2) | 25 (53.2) | 10 (45.5) | 15 (60.0) | 106 (45.9) | 42 (49.4) | 64 (43.8) |
| Hypertension | 146 (52.5) | 59 (55.1) | 87 (50.9) | 29 (61.7) | 14 (63.6) | 15 (60.0) | 117 (50.6) | 45 (52.9) | 72 (49.3) |
| Chronic liver disease | 42 (15.1) | 19 (17.8) | 23 (13.5) | 8 (17.0) | 5 (22.7) | 3 (12.0) | 34 (14.7) | 14 (16.5) | 20 (13.7) |
| Chronic kidney disease | 50 (18.0) | 26 (24.3) | 24 (14.0) | 9 (19.1) | 5 (22.7) | 4 (16.0) | 41 (17.7) | 21 (24.7) | 20 (13.7) |
| Type 2 diabetes | 66 (23.7) | 30 (28.0) | 36 (21.1) | 14 (29.8) | 7 (31.8) | 7 (28.0) | 52 (22.5) | 23 (27.1) | 29 (19.9) |
| Cancer | 81 (29.1) | 81 (75.7) | 0 (0.0) | 20 (42.6) | 20 (90.9) | 0 (0.0) | 61 (26.4) | 61 (71.8) | 0 (0.0) |
| Main IC ^h^ | 41 (14.7) | 41 (38.3) | 0 (0.0) | 5 (10.6) | 5 (22.7) | 0 (0.0) | 36 (15.6) | 36 (42.4) | 0 (0.0) |

^a^ Alternative definition of IC (Table 1); ^b^ Overall, the entire study time from 01 June 2021 to 31 May 2023, quarters based on the symptom onset date; Time periods are divided according to fiscal quarters, in brackets number of study sites in each quarter; ^c^ Among the patients eligible to receive booster (completed primary series); ^d^ Not mutually exclusive; ^e^ The recording of individual SARI symptoms became mandatory after COVIDRIVE master protocol v4.0 (October 10, 2022), therefore records of SARI patients admitted to hospital before this date may lack that information; ^f^ Main SARI definition (Table 1); ^g^ Main SARS-CoV-2 test status definition (Table 1); ^h^ Main IC definition (Table 1). Abbreviations: CI, confidence interval; CIRI-IT, Centro Interuniversitario di Ricerca sull'Influenza e le altre Infezioni Trasmissibili; GTPUH, Hospital Universitario Germans Trias i Pujol; HUVH, Hospital Universitari Vall d’Hebron; IC, immunocompromising condition; ICU, intensive care unit; IQR, interquartile range; n, number; Q, calendar time in fiscal quarter; SARI, severe acute respiratory infection; SARS-CoV-2, severe acute respiratory syndrome coronavirus 2; SD, standard deviation; UZA, Universitair Ziekenhuis Antwerpen. A square bracket “[“ and “]” in a range denotes that the number is included in the range, while regular brackets “(“ and “)” in a range means that the number is not included.

Table S10. Demographic and clinical characteristics of hospitalized SARI patients in three European countries, by study site, and by IC status (main definition)

| **Covariates** | **All SARI ^e^** | | | | | **IC ^a^** | | | | | **Non-IC** | | | | |
| --- | --- | --- | --- | --- | --- | --- | --- | --- | --- | --- | --- | --- | --- | --- | --- |
|  | **CIRI-IT** | **GTPUH** | **CHU Saint-Pierre** | **UZA** | **HUVH** | **CIRI-IT** | **GTPUH** | **CHU Saint-Pierre** | **UZA** | **HUVH** | **CIRI-IT** | **GTPUH** | **CHU Saint-Pierre** | **UZA** | **HUVH** |
| **DEMOGRAPHICS** | | | | | | | | | | | | | | | |
| **Age (years)** |  |  |  |  |  |  |  |  |  |  |  |  |  |  |  |
| Mean (SD) | 68.0 (16.6) | 70.2 (16.2) | 60.0 (17.2) | 59.2 (18.5) | 67.1 (16.7) | 61.6 (13.3) | 64.9 (13.8) | 41.7 (13.5) | 58.1 (16.4) | 62.0 (16.1) | 68.4 (16.8) | 71.2 (16.4) | 60.7 (17.0) | 59.5 (19.0) | 68.3 (16.6) |
| Median  (IQR) | 71.0  (58.0, 81.0) | 73.0  (60.8, 83.0) | 61.0  (51.0, 73.0) | 61.0  (45.0, 76.0) | 70.0  (59.0, 79.0) | 63.0  (56.0, 72.0) | 67.0  (57.0, 74.0) | 44.5  (31.0, 52.0) | 58.0  (45.0, 76.0) | 65.0  (53.0, 74.0) | 72.0  (59.0, 81.0) | 74.5  (62.0, 84.0) | 62.0  (51.0, 74.0) | 62.0  (45.0, 76.0) | 71.0  (60.0, 81.0) |
| Range | 18.0 - 101.0 | 18.0 - 103.0 | 22.0 - 98.0 | 18.0 - 95.0 | 18.0 - 102.0 | 18.0 - 89.0 | 23.0 - 93.0 | 24.0 - 56.0 | 21.0 - 84.0 | 18.0 - 90.0 | 18.0 - 101.0 | 18.0 - 103.0 | 22.0 - 98.0 | 18.0 -  95.0 | 18.0 - 102.0 |
| **Sex, n (%)** |  |  |  |  |  |  |  |  |  |  |  |  |  |  |  |
| Male | 720 (55.9) | 989 (56.7) | 118 (68.2) | 235 (59.5) | 920 (54.8) | 46 (63.0) | 171 (62.4) | 3  (50.0) | 44 (57.1) | 159 (52.6) | 674 (55.4) | 818 (55.6) | 115 (68.9) | 191  (60.1) | 761 (55.3) |
| Female | 569 (44.1) | 755 (43.3) | 55 (31.8) | 160 (40.5) | 759 (45.2) | 27 (37.0) | 103 (37.6) | 3  (50.0) | 33 (42.9) | 143 (47.4) | 542 (44.6) | 652 (44.4) | 52 (31.1) | 127  (39.9) | 616 (44.7) |
| **Country, n (%)** |  |  |  |  |  |  |  |  |  |  |  |  |  |  |  |
| Belgium | 0  (0.0) | 0  (0.0) | 173  (100.0) | 395  (100.0) | 0  (0.0) | 0  (0.0) | 0  (0.0) | 6  (100.0) | 77  (100.0) | 0  (0.0) | 0  (0.0) | 0  (0.0) | 167  (100.0) | 318  (100.0) | 0  (0.0) |
| Italy | 1,289  (100.0) | 0  (0.0) | 0  (0.0) | 0  (0.0) | 0  (0.0) | 73  (100.0) | 0  (0.0) | 0  (0.0) | 0  (0.0) | 0  (0.0) | 1,216 (100.0) | 0  (0.0) | 0  (0.0) | 0  (0.0) | 0  (0.0) |
| Spain | 0  (0.0) | 1,744  (100.0) | 0  (0.0) | 0  (0.0) | 1,679  (100.0) | 0  (0.0) | 274  (100.0) | 0  (0.0) | 0  (0.0) | 302  (100.0) | 0  (0.0) | 1,470  (100.0) | 0  (0.0) | 0  (0.0) | 1,377  (100.0) |
| **Smoking, n (%)** |  |  |  |  |  |  |  |  |  |  |  |  |  |  |  |
| Never smoker | 287 (22.3) | 810 (46.4) | 57 (32.9) | 57 (14.4) | 798 (47.5) | 23 (31.5) | 105 (38.3) | 4  (66.7) | 12 (15.6) | 155 (51.3) | 264 (21.7) | 705 (48.0) | 53 (31.7) | 45  (14.2) | 643 (46.7) |
| Former smoker | 309 (24.0) | 437 (25.1) | 52 (30.1) | 57 (14.4) | 492 (29.3) | 26 (35.6) | 91  (33.2) | 1  (16.7) | 6  (7.8) | 76 (25.2) | 283 (23.3) | 346 (23.5) | 51 (30.5) | 51  (16.0) | 416 (30.2) |
| Current smoker | 244 (18.9) | 257 (14.7) | 59 (34.1) | 36  (9.1) | 274 (16.3) | 14 (19.2) | 50  (18.2) | 0  (0.0) | 5  (6.5) | 38 (12.6) | 230 (18.9) | 207 (14.1) | 59 (35.3) | 31  (9.7) | 236 (17.1) |
| Missing | 449 (34.8) | 240 (13.8) | 5  (2.9) | 245 (62.0) | 115 (6.8) | 10 (13.7) | 28  (10.2) | 1  (16.7) | 54 (70.1) | 33 (10.9) | 439 (36.1) | 212 (14.4) | 4  (2.4) | 191  (60.1) | 82  (6.0) |
| **Long-term care facility residence, n (%)** |  |  |  |  |  |  |  |  |  |  |  |  |  |  |  |
| Yes | 8  (0.6) | 121  (7.1) | 10  (5.8) | 2  (0.6) | 60  (3.7) | 0  (0.0) | 8  (3.0) | 0  (0.0) | 0  (0.0) | 5  (1.7) | 8  (0.7) | 113  (7.8) | 10  (6.0) | 2  (0.7) | 55  (4.1) |
| **VACCINATION STATUS AT TIME OF HOSPITAL ADMISSION** | | | | | | | | | | | | | | | |
| Unvaccinated, n (%) | 152 (11.8) | 187 (10.7) | 46 (26.6) | 116 (29.4) | 140 (8.3) | 5  (6.8) | 19  (6.9) | 1  (16.7) | 13 (16.9) | 12  (4.0) | 147 (12.1) | 168 (11.4) | 45 (26.9) | 103  (32.4) | 128  (9.3) |
| Incomplete primary series, n (%) | 30  (2.3) | 62  (3.6) | 5  (2.9) | 8  (2.0) | 29  (1.7) | 1  (1.4) | 7  (2.6) | 0  (0.0) | 0  (0.0) | 6  (2.0) | 29  (2.4) | 55  (3.7) | 5  (3.0) | 8  (2.5) | 23  (1.7) |
| Primary series completed but no boosters, n (%) | 200 (15.5) | 661 (37.9) | 69 (39.9) | 107 (27.1) | 272 (16.2) | 10 (13.7) | 100 (36.5) | 2  (33.3) | 20 (26.0) | 51 (16.9) | 190 (15.6) | 561 (38.2) | 67 (40.1) | 87  (27.4) | 221 (16.0) |
| At least one booster dose, n (%) | 907 (70.4) | 834 (47.8) | 53 (30.6) | 164 (41.5) | 1238 (73.7) | 57 (78.1) | 148 (54.0) | 3  (50.0) | 44 (57.1) | 233 (77.2) | 850 (69.9) | 686 (46.7) | 50 (29.9) | 120  (37.7) | 1,005 (73.0) |
| **Number of booster doses ^b^, n (%)** |  |  |  |  |  |  |  |  |  |  |  |  |  |  |  |
| 0 | 200 (18.1) | 661 (44.2) | 69 (56.6) | 107 (39.5) | 272 (18.0) | 10 (14.9) | 100 (40.3) | 2  (40.0) | 20 (31.2) | 51 (18.0) | 190 (18.3) | 561 (45.0) | 67 (57.3) | 87  (42.0) | 221 (18.0) |
| 1 | 780 (70.5) | 692 (46.3) | 52 (42.6) | 107 (39.5) | 744 (49.3) | 43 (64.2) | 127 (51.2) | 3  (60.0) | 35 (54.7) | 127 (44.7) | 737 (70.9) | 565 (45.3) | 49 (41.9) | 72  (34.8) | 617 (50.3) |
| 2 | 126 (11.4) | 139  (9.3) | 1  (0.8) | 42 (15.5) | 467 (30.9) | 14 (20.9) | 20  (8.1) | 0  (0.0) | 4  (6.2) | 87 (30.6) | 112 (10.8) | 119  (9.5) | 1  (0.9) | 38  (18.4) | 380 (31.0) |
| 3+ | 1  (0.1) | 3  (0.2) | 0  (0.0) | 15  (5.5) | 27  (1.8) | 0  (0.0) | 1  (0.4) | 0  (0.0) | 5  (7.8) | 19  (6.7) | 1  (0.1) | 2  (0.2) | 0  (0.0) | 10  (4.8) | 8  (0.7) |
| **Time since last vaccine dose (days)** |  |  |  |  |  |  |  |  |  |  |  |  |  |  |  |
| Mean (SD) | 192.7 (132.0) | 154.5 (124.6) | 240.4 (166.2) | 148.6 (101.7) | 220.7 (150.5) | 195.6 (114.0) | 158.0 (124.0) | 242.6 (170.3) | 143.4 (116.9) | 243.8 (154.1) | 192.5 (133.1) | 153.8 (124.8) | 240.3 (166.8) | 150.1 (96.9) | 215.4 (149.2) |
| Median  (IQR) | 160.0  (84.0, 285.0) | 127.0  (63.0, 210.0) | 220.0  (98.0, 368.5) | 132.0  (76.0, 188.0) | 188.0  (106.0, 318.5) | 173.0  (103.5, 257.2) | 132.0  (66.5, 218.5) | 286.0  (119.0, 343.0) | 115.0  (57.5, 163.8) | 222.5  (127.8, 332.0) | 159.0  (83.0, 285.0) | 126.0  (63.0, 209.0) | 219.0  (96.0, 368.8) | 138.0  (82.0, 191.0) | 179.0  (104.0, 316.0) |
| Range | 1.0 - 782.0 | 1.0 - 740.0 | 1.0 - 645.0 | 6.0 - 591.0 | 1.0 - 711.0 | 21.0 - 484.0 | 1.0 - 671.0 | 22.0 - 443.0 | 14.0 - 521.0 | 1.0 - 710.0 | 1.0 - 782.0 | 1.0 - 740.0 | 1.0 - 645.0 | 6.0 -  591.0 | 1.0 - 711.0 |
| **Time since last vaccine dose, n (%)** |  |  |  |  |  |  |  |  |  |  |  |  |  |  |  |
| <2 months | 163 (14.3) | 362 (23.2) | 19 (15.0) | 47 (16.8) | 207 (13.5) | 5  (7.4) | 57  (22.4) | 1  (20.0) | 17 (26.6) | 29 (10.0) | 158 (14.8) | 305 (23.4) | 18 (14.8) | 30  (14.0) | 178 (14.3) |
| [2 - 4) months | 267 (23.5) | 370 (23.8) | 17 (13.4) | 77 (27.6) | 239 (15.5) | 15 (22.1) | 58  (22.7) | 1  (20.0) | 17 (26.6) | 41 (14.1) | 252 (23.6) | 312 (24.0) | 16 (13.1) | 60  (27.9) | 198 (15.9) |
| [4 - 6) months | 200 (17.6) | 316 (20.3) | 21 (16.5) | 77 (27.6) | 302 (19.6) | 16 (23.5) | 50  (19.6) | 0  (0.0) | 16 (25.0) | 48 (16.6) | 184 (17.2) | 266 (20.4) | 21 (17.2) | 61  (28.4) | 254 (20.3) |
| [6 - 8) months | 152 (13.4) | 221 (14.2) | 8  (6.3) | 43 (15.4) | 213 (13.8) | 12 (17.6) | 38  (14.9) | 0  (0.0) | 3  (4.7) | 40 (13.8) | 140 (13.1) | 183 (14.1) | 8  (6.6) | 40  (18.6) | 173 (13.9) |
| ≥8 months | 355 (31.2) | 288 (18.5) | 62 (48.8) | 35 (12.5) | 578 (37.6) | 20 (29.4) | 52  (20.4) | 3  (60.0) | 11 (17.2) | 132 (45.5) | 335 (31.3) | 236 (18.1) | 59 (48.4) | 24  (11.2) | 446 (35.7) |
| **SYMPTOMS** | | | | | | | | | | | | | | | |
| **SARI symptoms ^c,d^, n (%)** |  |  |  |  |  |  |  |  |  |  |  |  |  |  |  |
| Cough | 1,134 (88.0) | 867 (53.3) | 130 (75.6) | 276 (81.9) | 1,170 (73.9) | 66 (90.4) | 132 (49.8) | 5  (83.3) | 60 (85.7) | 193 (67.0) | 1,068 (87.8) | 735 (54.0) | 125 (75.3) | 216  (80.9) | 977 (75.4) |
| Fever | 1,195 (92.7) | 707 (43.7) | 73 (42.2) | 203 (61.5) | 922 (58.5) | 68 (93.2) | 144 (54.5) | 4  (66.7) | 47 (67.1) | 187 (64.9) | 1,127 (92.7) | 563 (41.5) | 69 (41.3) | 156  (60.0) | 735 (57.1) |
| Shortness of breath | 739 (57.5) | 1,221 (74.8) | 140 (80.9) | 276 (78.4) | 1,148 (72.5) | 39 (53.4) | 177 (66.3) | 5  (83.3) | 46 (65.7) | 179 (62.2) | 700 (57.7) | 1,044 (76.4) | 135 (80.8) | 230  (81.6) | 969 (74.8) |
| Anosmia, ageusia or dysgeusia | 108 (8.4) | 19  (1.2) | 3  (1.8) | 21 (20.2) | 21  (1.4) | 8  (11.0) | 2  (0.8) | 0  (0.0) | 6  (26.1) | 5  (1.8) | 100 (8.2) | 17  (1.3) | 3  (1.9) | 15  (18.5) | 16  (1.3) |
| **Number of SARI symptoms, n (%)** |  |  |  |  |  |  |  |  |  |  |  |  |  |  |  |
| 1 | 76  (5.9) | 657 (41.0) | 38 (23.2) | 18 (18.0) | 363 (23.4) | 2  (2.7) | 112 (43.1) | 1  (25.0) | 6  (27.3) | 87 (30.7) | 74  (6.1) | 545 (40.6) | 37 (23.1) | 12  (15.4) | 276 (21.7) |
| 2 | 604 (47.0) | 732 (45.7) | 89 (54.3) | 38 (38.0) | 752 (48.5) | 38 (52.1) | 111 (42.7) | 1  (25.0) | 5  (22.7) | 126 (44.5) | 566 (46.7) | 621 (46.3) | 88 (55.0) | 33  (42.3) | 626 (49.3) |
| 3+ | 605 (47.1) | 213 (13.3) | 37 (22.6) | 44 (44.0) | 437 (28.2) | 33 (45.2) | 37  (14.2) | 2  (50.0) | 11 (50.0) | 70 (24.7) | 572 (47.2) | 176 (13.1) | 35 (21.9) | 33  (42.3) | 367 (28.9) |
| **HOSPITAL OUTCOMES** | | | | | | | | | | | | | | | |
| **Length of stay (days)** |  |  |  |  |  |  |  |  |  |  |  |  |  |  |  |
| Mean (SD) | 9.9 (12.3) | 10.0 (13.6) | 16.1 (27.1) | 10.1 (15.2) | 9.8 (12.5) | 13.6 (17.5) | 9.3  (10.1) | 26.0 (16.3) | 11.5 (20.7) | 11.6 (18.4) | 9.7 (11.9) | 10.2 (14.1) | 15.7 (27.4) | 9.7  (13.5) | 9.4  (10.8) |
| Median  (IQR) | 6.0 (3.0, 11.0) | 7.0  (4.0, 12.0) | 7.0 (5.0, 14.0) | 5.0 (3.0, 11.0) | 6.0  (3.0, 11.0) | 8.0 (5.0, 15.0) | 6.0  (4.0, 11.0) | 25.0 (14.5, 34.8) | 5.0 (3.0, 10.0) | 6.0  (4.0, 12.0) | 6.0 (3.0, 11.0) | 7.0  (4.0, 12.0) | 7.0 (5.0, 13.0) | 5.0  (3.0,  11.0) | 6.0  (3.0, 11.0) |
| Range | 1.0 - 146.0 | 1.0 - 403.0 | 1.0 - 201.0 | 1.0 - 125.0 | 1.0 - 173.0 | 1.0 - 89.0 | 1.0 - 104.0 | 7.0 - 50.0 | 1.0 - 122.0 | 1.0 - 173.0 | 1.0 - 146.0 | 1.0 - 403.0 | 1.0 - 201.0 | 1.0 -  125.0 | 1.0 - 116.0 |
| **Severe outcomes, n (%)** |  |  |  |  |  |  |  |  |  |  |  |  |  |  |  |
| Hospitalization without ICU admission or in-hospital death | 1,239 (96.2) | 1,427 (81.8) | 145 (83.8) | 334 (84.6) | 1,419 (84.5) | 71 (97.3) | 229 (83.6) | 5  (83.3) | 68 (88.3) | 255 (84.4) | 1,168 (96.1) | 1,198 (81.5) | 140 (83.8) | 266  (83.6) | 1,164 (84.5) |
| ICU admission without in-hospital death | 15  (1.2) | 121  (6.9) | 19 (11.0) | 46 (11.6) | 133 (7.9) | 1  (1.4) | 11  (4.0) | 1  (16.7) | 4  (5.2) | 19 (6.3) | 14  (1.2) | 110 (7.5) | 18 (10.8) | 42  (13.2) | 114  (8.3) |
| ICU admission (with or without in-hospital death) | 20  (1.6) | 158  (9.1) | 23 (13.3) | 57 (14.4) | 177 (10.5) | 1  (1.4) | 17  (6.2) | 1  (16.7) | 8  (10.4) | 36 (11.9) | 19  (1.6) | 141  (9.6) | 22 (13.2) | 49  (15.4) | 141 (10.2) |
| In-hospital death | 34  (2.6) | 196 (11.2) | 9  (5.2) | 15  (3.8) | 127 (7.6) | 1  (1.4) | 34  (12.4) | 0  (0.0) | 5  (6.5) | 28  (9.3) | 33  (2.7) | 162 (11.0) | 9  (5.4) | 10  (3.1) | 99  (7.2) |
| **COMORBIDITIES** | | | | | | | | | | | | | | | |
| **Number of comorbidities other than IC, n (%)** |  |  |  |  |  |  |  |  |  |  |  |  |  |  |  |
| No comorbid conditions | 389  (30.2) | 217  (12.4) | 28  (16.2) | 132  (33.4) | 202  (12.0) | 13  (17.8) | 17  (6.2) | 3  (50.0) | 9  (11.7) | 22  (7.3) | 376 (30.9) | 200 (13.6) | 25 (15.0) | 123  (38.7) | 180  (13.1) |
| 1 comorbidity | 335 (26.0) | 355 (20.4) | 39 (22.5) | 80 (20.3) | 348 (20.7) | 16 (21.9) | 65  (23.7) | 2  (33.3) | 30 (39.0) | 67 (22.2) | 319 (26.2) | 290 (19.7) | 37 (22.2) | 50  (15.7) | 281 (20.4) |
| 2 comorbidities | 275 (21.3) | 417 (23.9) | 40 (23.1) | 86 (21.8) | 373 (22.2) | 18 (24.7) | 80  (29.2) | 0  (0.0) | 14 (18.2) | 67 (22.2) | 257 (21.1) | 337 (22.9) | 40 (24.0) | 72  (22.6) | 306 (22.2) |
| 3+ comorbidities | 290 (22.5) | 755 (43.3) | 66 (38.2) | 97 (24.6) | 756 (45.0) | 26 (35.6) | 112 (40.9) | 1  (16.7) | 24 (31.2) | 146 (48.3) | 264 (21.7) | 643 (43.7) | 65 (38.9) | 73  (23.0) | 610 (44.3) |
| **Number with specific comorbidities ^c^, n (%)** |  |  |  |  |  |  |  |  |  |  |  |  |  |  |  |
| Asthma | 51  (4.0) | 146  (8.4) | 29 (16.8) | 34  (8.6) | 141 (8.4) | 6  (8.2) | 18  (6.6) | 1  (16.7) | 6  (7.8) | 15  (5.0) | 45  (3.7) | 128  (8.7) | 28 (16.8) | 28  (8.8) | 126  (9.2) |
| Lung disease | 242 (18.8) | 634 (36.4) | 66 (38.2) | 87 (22.0) | 708 (42.2) | 17 (23.3) | 99  (36.1) | 1  (16.7) | 16 (20.8) | 150 (49.7) | 225 (18.5) | 535 (36.4) | 65 (38.9) | 71  (22.3) | 558 (40.5) |
| Cardiovascular disease | 442 (34.3) | 683 (39.2) | 73 (42.2) | 123 (31.1) | 785 (46.8) | 26 (35.6) | 76  (27.7) | 2  (33.3) | 17 (22.1) | 129 (42.7) | 416 (34.2) | 607 (41.3) | 71 (42.5) | 106  (33.3) | 656 (47.6) |
| Hypertension | 491 (38.1) | 1,010 (57.9) | 74 (42.8) | 122 (30.9) | 926 (55.2) | 31 (42.5) | 126 (46.0) | 1  (16.7) | 21 (27.3) | 139 (46.0) | 460 (37.8) | 884 (60.1) | 73 (43.7) | 101  (31.8) | 787 (57.2) |
| Chronic liver disease | 48  (3.7) | 118  (6.8) | 35 (20.2) | 23  (5.8) | 175 (10.4) | 11 (15.1) | 22  (8.0) | 0  (0.0) | 12 (15.6) | 43 (14.2) | 37  (3.0) | 96  (6.5) | 35 (21.0) | 11  (3.5) | 132  (9.6) |
| Chronic kidney disease | 100 (7.8) | 410 (23.5) | 23 (13.3) | 67 (17.0) | 304 (18.1) | 19 (26.0) | 54  (19.7) | 1  (16.7) | 17 (22.1) | 104 (34.4) | 81  (6.7) | 356 (24.2) | 22 (13.2) | 50  (15.7) | 200 (14.5) |
| Type 2 diabetes | 269 (20.9) | 533 (30.6) | 41 (23.7) | 81 (20.5) | 466 (27.8) | 16 (21.9) | 64  (23.4) | 0  (0.0) | 14 (18.2) | 82 (27.2) | 253 (20.8) | 469 (31.9) | 41 (24.6) | 67  (21.1) | 384 (27.9) |
| Cancer | 248 (19.2) | 389 (22.3) | 27 (15.6) | 59 (14.9) | 449 (26.7) | 23 (31.5) | 182 (66.4) | 1  (16.7) | 41 (53.2) | 136 (45.0) | 225 (18.5) | 207 (14.1) | 26 (15.6) | 18  (5.7) | 313 (22.7) |
| Alternative IC ^f^ | 298 (23.1) | 481 (27.6) | 32 (18.5) | 95 (24.1) | 615 (36.6) | 73 (100.0) | 274 (100.0) | 6 (100.0) | 77 (100.0) | 302 (100.0) | 225 (18.5) | 207 (14.1) | 26 (15.6) | 18  (5.7) | 313 (22.7) |

^a^ Main definition of IC (Table 1); ^b^ Among the patients eligible to receive booster completed primary series); ^c^ Not mutually exclusive; ^d^ The recording of individual SARI symptoms became mandatory after COVIDRIVE master protocol v4.0 (October 10, 2022), therefore records of SARI patients admitted to hospital before this date may lack that information; ^e^ Main SARI definition (Table 1); ^f^ Alternative IC definition – main IC and/or cancer (Table 1). Abbreviations: CI, confidence interval; CIRI-IT, Centro Interuniversitario di Ricerca sull'Influenza e le altre Infezioni Trasmissibili; GTPUH, Hospital Universitario Germans Trias i Pujol; HUVH, Hospital Universitari Vall d’Hebron; IC, immunocompromising condition; ICU, intensive care unit; IQR, interquartile range; n, number; SARI, severe acute respiratory infection; SD, standard deviation; UZA, Universitair Ziekenhuis Antwerpen

Table S11. Demographic and clinical characteristics of hospitalized SARI patients in three European countries, by study site, and by IC status (alternative definition)

| **Covariates** | **All SARI ^e^** | | | | | **IC ^a^** | | | | | **Non-IC** | | | | |
| --- | --- | --- | --- | --- | --- | --- | --- | --- | --- | --- | --- | --- | --- | --- | --- |
|  | **CIRI-IT** | **GTPUH** | **CHU Saint-Pierre** | **UZA** | **HUVH** | **CIRI-IT** | **GTPUH** | **CHU Saint-Pierre** | **UZA** | **HUVH** | **CIRI-IT** | **GTPUH** | **CHU Saint-Pierre** | **UZA** | **HUVH** |
| **DEMOGRAPHICS** | | | | | | | | | | | | | | | |
| **Age (years)** |  |  |  |  |  |  |  |  |  |  |  |  |  |  |  |
| Mean (SD) | 68.0 (16.6) | 70.2 (16.2) | 60.0 (17.2) | 59.2 (18.5) | 67.1 (16.7) | 67.7 (14.4) | 69.3 (14.0) | 60.6 (16.6) | 59.9 (16.6) | 68.0 (15.4) | 68.0 (17.3) | 70.6 (17.0) | 59.9 (17.4) | 59.0 (19.1) | 66.6 (17.4) |
| Median  (IQR) | 71.0 (58.0, 81.0) | 73.0 (60.8, 83.0) | 61.0 (51.0, 73.0) | 61.0 (45.0, 76.0) | 70.0 (59.0, 79.0) | 69.5 (59.0, 77.0) | 71.0 (61.0, 79.0) | 62.5 (53.2, 72.2) | 61.0 (48.5, 76.0) | 70.0 (60.0, 79.0) | 72.0 (58.0, 82.0) | 74.0 (60.0, 84.0) | 60.0 (50.0, 74.0) | 60.5  (43.8, 76.0) | 69.0 (57.0, 79.0) |
| Range | 18.0 - 101.0 | 18.0 - 103.0 | 22.0 - 98.0 | 18.0 - 95.0 | 18.0 - 102.0 | 18.0 - 99.0 | 23.0 - 99.0 | 24.0 - 87.0 | 21.0 - 88.0 | 18.0 - 98.0 | 18.0 - 101.0 | 18.0 - 103.0 | 22.0 - 98.0 | 18.0 -  95.0 | 18.0 - 102.0 |
| **Sex, n (%)** |  |  |  |  |  |  |  |  |  |  |  |  |  |  |  |
| Male | 720 (55.9) | 989 (56.7) | 118 (68.2) | 235 (59.5) | 920 (54.8) | 170 (57.0) | 303 (63.0) | 18 (56.2) | 53 (55.8) | 368 (59.8) | 550 (55.5) | 686 (54.3) | 100 (70.9) | 182  (60.7) | 552 (51.9) |
| Female | 569 (44.1) | 755 (43.3) | 55 (31.8) | 160 (40.5) | 759 (45.2) | 128 (43.0) | 178 (37.0) | 14 (43.8) | 42 (44.2) | 247 (40.2) | 441 (44.5) | 577 (45.7) | 41 (29.1) | 118  (39.3) | 512 (48.1) |
| **Country, n (%)** |  |  |  |  |  |  |  |  |  |  |  |  |  |  |  |
| Belgium | 0  (0.0) | 0  (0.0) | 173 (100.0) | 395 (100.0) | 0  (0.0) | 0  (0.0) | 0  (0.0) | 32 (100.0) | 95 (100.0) | 0  (0.0) | 0  (0.0) | 0  (0.0) | 141 (100.0) | 300 (100.0) | 0  (0.0) |
| Italy | 1,289 (100.0) | 0  (0.0) | 0  (0.0) | 0  (0.0) | 0  (0.0) | 298 (100.0) | 0  (0.0) | 0  (0.0) | 0  (0.0) | 0  (0.0) | 991 (100.0) | 0  (0.0) | 0  (0.0) | 0  (0.0) | 0  (0.0) |
| Spain | 0  (0.0) | 1,744 (100.0) | 0  (0.0) | 0  (0.0) | 1,679 (100.0) | 0  (0.0) | 481 (100.0) | 0  (0.0) | 0  (0.0) | 615 (100.0) | 0  (0.0) | 1,263 (100.0) | 0  (0.0) | 0  (0.0) | 1,064 (100.0) |
| **Smoking, n (%)** |  |  |  |  |  |  |  |  |  |  |  |  |  |  |  |
| Never smoker | 287 (22.3) | 810 (46.4) | 57 (32.9) | 57 (14.4) | 798 (47.5) | 64 (21.5) | 177 (36.8) | 12 (37.5) | 13 (13.7) | 280 (45.5) | 223 (22.5) | 633 (50.1) | 45 (31.9) | 44  (14.7) | 518 (48.7) |
| Former smoker | 309 (24.0) | 437 (25.1) | 52 (30.1) | 57 (14.4) | 492 (29.3) | 96 (32.2) | 171 (35.6) | 10 (31.2) | 13 (13.7) | 198 (32.2) | 213 (21.5) | 266 (21.1) | 42 (29.8) | 44  (14.7) | 294 (27.6) |
| Current smoker | 244 (18.9) | 257 (14.7) | 59 (34.1) | 36  (9.1) | 274 (16.3) | 46 (15.4) | 75  (15.6) | 9  (28.1) | 7  (7.4) | 79 (12.8) | 198 (20.0) | 182 (14.4) | 50 (35.5) | 29  (9.7) | 195 (18.3) |
| Missing | 449 (34.8) | 240 (13.8) | 5  (2.9) | 245 (62.0) | 115 (6.8) | 92 (30.9) | 58  (12.1) | 1  (3.1) | 62 (65.3) | 58  (9.4) | 357 (36.0) | 182 (14.4) | 4  (2.8) | 183  (61.0) | 57  (5.4) |
| **Long-term care facility residence, n (%)** |  |  |  |  |  |  |  |  |  |  |  |  |  |  |  |
| Yes | 8  (0.6) | 121  (7.1) | 10  (5.8) | 2  (0.6) | 60  (3.7) | 1  (0.3) | 19  (4.1) | 2  (6.2) | 0  (0.0) | 20  (3.4) | 7  (0.7) | 102  (8.2) | 8  (5.7) | 2  (0.7) | 40  (3.9) |
| **VACCINATION STATUS AT TIME OF HOSPITAL ADMISSION** | | | | | | | | | | | | | | | |
| Unvaccinated, n (%) | 152 (11.8) | 187 (10.7) | 46 (26.6) | 116 (29.4) | 140 (8.3) | 22  (7.4) | 28  (5.8) | 7  (21.9) | 14 (14.7) | 23  (3.7) | 130 (13.1) | 159 (12.6) | 39 (27.7) | 102  (34.0) | 117 (11.0) |
| Incomplete primary series, n (%) | 30  (2.3) | 62  (3.6) | 5  (2.9) | 8  (2.0) | 29  (1.7) | 8  (2.7) | 14  (2.9) | 1  (3.1) | 1  (1.1) | 7  (1.1) | 22  (2.2) | 48  (3.8) | 4  (2.8) | 7  (2.3) | 22  (2.1) |
| Primary series completed but no boosters, n (%) | 200 (15.5) | 661 (37.9) | 69 (39.9) | 107 (27.1) | 272 (16.2) | 48 (16.1) | 185 (38.5) | 11 (34.4) | 24 (25.3) | 87 (14.1) | 152 (15.3) | 476 (37.7) | 58 (41.1) | 83  (27.7) | 185 (17.4) |
| At least one booster dose, n (%) | 907 (70.4) | 834 (47.8) | 53 (30.6) | 164 (41.5) | 1,238 (73.7) | 220 (73.8) | 254 (52.8) | 13 (40.6) | 56 (58.9) | 498 (81.0) | 687 (69.3) | 580 (45.9) | 40 (28.4) | 108  (36.0) | 740 (69.5) |
| **Number of booster doses ^b^, n (%)** |  |  |  |  |  |  |  |  |  |  |  |  |  |  |  |
| 0 | 200 (18.1) | 661 (44.2) | 69 (56.6) | 107 (39.5) | 272 (18.0) | 48 (17.9) | 185 (42.1) | 11 (45.8) | 24 (30.0) | 87 (14.9) | 152 (18.1) | 476 (45.1) | 58 (59.2) | 83  (43.5) | 185 (20.0) |
| 1 | 780 (70.5) | 692 (46.3) | 52 (42.6) | 107 (39.5) | 744 (49.3) | 177 (66.0) | 218 (49.7) | 13 (54.2) | 41 (51.2) | 283 (48.4) | 603 (71.9) | 474 (44.9) | 39 (39.8) | 66  (34.6) | 461 (49.8) |
| 2 | 126 (11.4) | 139  (9.3) | 1  (0.8) | 42 (15.5) | 467 (30.9) | 42 (15.7) | 35  (8.0) | 0  (0.0) | 8  (10.0) | 192 (32.8) | 84 (10.0) | 104  (9.8) | 1  (1.0) | 34  (17.8) | 275 (29.7) |
| 3+ | 1  (0.1) | 3  (0.2) | 0  (0.0) | 15  (5.5) | 27  (1.8) | 1  (0.4) | 1  (0.2) | 0  (0.0) | 7  (8.8) | 23  (3.9) | 0  (0.0) | 2  (0.2) | 0  (0.0) | 8  (4.2) | 4  (0.4) |
| **Time since last vaccine dose (days)** |  |  |  |  |  |  |  |  |  |  |  |  |  |  |  |
| Mean (SD) | 192.7 (132.0) | 154.5 (124.6) | 240.4 (166.2) | 148.6 (101.7) | 220.7 (150.5) | 188.7 (129.5) | 154.4 (121.0) | 250.6 (173.1) | 152.0 (118.6) | 226.7 (149.2) | 194.0 (132.8) | 154.5 (126.1) | 237.9 (165.3) | 147.2 (94.2) | 217.0 (151.2) |
| Median  (IQR) | 160.0 (84.0, 285.0) | 127.0 (63.0, 210.0) | 220.0 (98.0, 368.5) | 132.0 (76.0, 188.0) | 188.0 (106.0, 318.5) | 158.0 (85.5, 275.8) | 128.0 (67.0, 209.0) | 258.0 (119.0, 343.0) | 121.0 (61.0, 208.0) | 200.0 (114.0, 318.2) | 161.0 (84.0, 289.0) | 126.5 (63.0, 210.2) | 219.0 (96.0, 368.8) | 137.0 (84.0, 186.0) | 179.0 (101.0, 318.5) |
| Range | 1.0 - 782.0 | 1.0 - 740.0 | 1.0 - 645.0 | 6.0 - 591.0 | 1.0 - 711.0 | 4.0 - 755.0 | 1.0 - 740.0 | 19.0 - 580.0 | 14.0 - 521.0 | 1.0 - 710.0 | 1.0 - 782.0 | 1.0 - 738.0 | 1.0 - 645.0 | 6.0 -  591.0 | 1.0 - 711.0 |
| **Time since last vaccine dose, n (%)** |  |  |  |  |  |  |  |  |  |  |  |  |  |  |  |
| <2 months | 163 (14.3) | 362 (23.2) | 19 (15.0) | 47 (16.8) | 207 (13.5) | 35 (12.7) | 100 (22.1) | 3  (12.0) | 20 (24.7) | 69 (11.7) | 128 (14.9) | 262 (23.7) | 16 (15.7) | 27  (13.6) | 138 (14.6) |
| [2 - 4) months | 267 (23.5) | 370 (23.8) | 17 (13.4) | 77 (27.6) | 239 (15.5) | 72 (26.1) | 111 (24.5) | 4  (16.0) | 20 (24.7) | 91 (15.4) | 195 (22.6) | 259 (23.5) | 13 (12.7) | 57  (28.8) | 148 (15.6) |
| [4 - 6) months | 200 (17.6) | 316 (20.3) | 21 (16.5) | 77 (27.6) | 302 (19.6) | 53 (19.2) | 87  (19.2) | 4  (16.0) | 18 (22.2) | 109 (18.4) | 147 (17.1) | 229 (20.7) | 17 (16.7) | 59  (29.8) | 193 (20.4) |
| [6 - 8) months | 152 (13.4) | 221 (14.2) | 8  (6.3) | 43 (15.4) | 213 (13.8) | 34 (12.3) | 75  (16.6) | 1  (4.0) | 7  (8.6) | 89 (15.0) | 118 (13.7) | 146 (13.2) | 7  (6.9) | 36  (18.2) | 124 (13.1) |
| ≥8 months | 355 (31.2) | 288 (18.5) | 62 (48.8) | 35 (12.5) | 578 (37.6) | 82 (29.7) | 80  (17.7) | 13 (52.0) | 16 (19.8) | 234 (39.5) | 273 (31.7) | 208 (18.8) | 49 (48.0) | 19  (9.6) | 344 (36.3) |
| **SYMPTOMS** | | | | | | | | | | | | | | | |
| **SARI symptoms ^c,d^, n (%)** |  |  |  |  |  |  |  |  |  |  |  |  |  |  |  |
| Cough | 1,134 (88.0) | 867 (53.3) | 130 (75.6) | 276 (81.9) | 1,170 (73.9) | 257 (86.2) | 219 (48.2) | 24 (75.0) | 67 (79.8) | 394 (67.8) | 877 (88.5) | 648 (55.2) | 106 (75.7) | 209  (82.6) | 776 (77.4) |
| Fever | 1,195 (92.7) | 707 (43.7) | 73 (42.2) | 203 (61.5) | 922 (58.5) | 277 (93.0) | 226 (49.9) | 15 (46.9) | 58 (69.0) | 366 (63.1) | 918 (92.6) | 481 (41.3) | 58 (41.1) | 145  (58.9) | 556 (55.9) |
| Shortness of breath | 739 (57.5) | 1,221 (74.8) | 140 (80.9) | 276 (78.4) | 1,148 (72.5) | 171 (57.6) | 316 (69.5) | 27 (84.4) | 61 (70.9) | 376 (64.7) | 568 (57.4) | 905 (76.8) | 113 (80.1) | 215  (80.8) | 772 (77.0) |
| Anosmia, ageusia or dysgeusia | 108 (8.4) | 19  (1.2) | 3  (1.8) | 21 (20.2) | 21  (1.4) | 27  (9.1) | 2  (0.4) | 2  (6.7) | 7  (29.2) | 9  (1.6) | 81  (8.2) | 17  (1.5) | 1  (0.7) | 14  (17.5) | 12  (1.2) |
| **Number of SARI symptoms, n (%)** |  |  |  |  |  |  |  |  |  |  |  |  |  |  |  |
| 1 | 76  (5.9) | 657 (41.0) | 38 (23.2) | 18 (18.0) | 363 (23.4) | 18  (6.1) | 200 (44.7) | 6  (20.0) | 6  (26.1) | 166 (29.0) | 58  (5.9) | 457 (39.6) | 32 (23.9) | 12  (15.6) | 197 (20.1) |
| 2 | 604 (47.0) | 732 (45.7) | 89 (54.3) | 38 (38.0) | 752 (48.5) | 143 (48.1) | 191 (42.7) | 15 (50.0) | 6  (26.1) | 264 (46.2) | 461 (46.7) | 541 (46.8) | 74 (55.2) | 32  (41.6) | 488 (49.8) |
| 3+ | 605 (47.1) | 213 (13.3) | 37 (22.6) | 44 (44.0) | 437 (28.2) | 136 (45.8) | 56  (12.5) | 9  (30.0) | 11 (47.8) | 142 (24.8) | 469 (47.5) | 157 (13.6) | 28 (20.9) | 33  (42.9) | 295 (30.1) |
| **HOSPITAL OUTCOMES** | | | | | | | | | | | | | | | |
| **Length of stay (days)** |  |  |  |  |  |  |  |  |  |  |  |  |  |  |  |
| Mean (SD) | 9.9 (12.3) | 10.0 (13.6) | 16.1 (27.1) | 10.1 (15.2) | 9.8 (12.5) | 12.5 (16.7) | 9.5  (9.3) | 15.5 (20.3) | 10.9 (18.8) | 10.7 (15.1) | 9.2 (10.5) | 10.2 (14.9) | 16.2 (28.5) | 9.8  (13.8) | 9.3  (10.7) |
| Median  (IQR) | 6.0 (3.0, 11.0) | 7.0  (4.0, 12.0) | 7.0 (5.0, 14.0) | 5.0 (3.0, 11.0) | 6.0  (3.0, 11.0) | 7.0 (4.0, 14.0) | 7.0  (4.0, 12.0) | 7.0 (5.8, 14.5) | 5.0 (3.0, 10.5) | 6.0  (4.0, 11.0) | 6.0 (3.0, 11.0) | 7.0  (4.0, 12.0) | 7.0 (5.0, 14.0) | 5.0  (3.0,  11.2) | 6.0  (3.0, 12.0) |
| Range | 1.0 - 146.0 | 1.0 - 403.0 | 1.0 - 201.0 | 1.0 - 125.0 | 1.0 - 173.0 | 1.0 - 146.0 | 1.0 - 104.0 | 3.0 - 101.0 | 1.0 - 122.0 | 1.0 - 173.0 | 1.0 - 105.0 | 1.0 - 403.0 | 1.0 - 201.0 | 1.0 -  125.0 | 1.0 - 116.0 |
| **Severe outcomes, n (%)** |  |  |  |  |  |  |  |  |  |  |  |  |  |  |  |
| Hospitalization without ICU admission or in-hospital death | 1,239 (96.2) | 1,427 (81.8) | 145 (83.8) | 334 (84.6) | 1,419 (84.5) | 280 (94.0) | 406 (84.4) | 23 (71.9) | 84 (88.4) | 522 (84.9) | 959 (96.9) | 1,021 (80.8) | 122 (86.5) | 250  (83.3) | 897 (84.3) |
| ICU admission without in-hospital death | 15  (1.2) | 121  (6.9) | 19 (11.0) | 46 (11.6) | 133 (7.9) | 3  (1.0) | 20  (4.2) | 5  (15.6) | 6  (6.3) | 32  (5.2) | 12  (1.2) | 101  (8.0) | 14  (9.9) | 40  (13.3) | 101  (9.5) |
| ICU admission (with or without in-hospital death) | 20  (1.6) | 158  (9.1) | 23 (13.3) | 57 (14.4) | 177 (10.5) | 5  (1.7) | 27  (5.6) | 6  (18.8) | 10 (10.5) | 56  (9.1) | 15  (1.5) | 131 (10.4) | 17 (12.1) | 47  (15.7) | 121 (11.4) |
| In-hospital death | 34  (2.6) | 196 (11.2) | 9  (5.2) | 15  (3.8) | 127 (7.6) | 15  (5.0) | 55  (11.4) | 4  (12.5) | 5  (5.3) | 61  (9.9) | 19  (1.9) | 141 (11.2) | 5  (3.5) | 10  (3.3) | 66  (6.2) |
| **COMORBIDITIES** | | | | | | | | | | | | | | | |
| **Number of comorbidities other than IC, n (%)** |  |  |  |  |  |  |  |  |  |  |  |  |  |  |  |
| No comorbid conditions | 389 (30.2) | 217 (12.4) | 28 (16.2) | 132 (33.4) | 202 (12.0) | 13  (4.4) | 17  (3.5) | 3  (9.4) | 9  (9.5) | 22  (3.6) | 376 (37.9) | 200 (15.8) | 25 (17.7) | 123  (41.0) | 180 (16.9) |
| 1 comorbidity | 335 (26.0) | 355 (20.4) | 39 (22.5) | 80 (20.3) | 348 (20.7) | 107 (35.9) | 91  (18.9) | 3  (9.4) | 33 (34.7) | 108 (17.6) | 228 (23.0) | 264 (20.9) | 36 (25.5) | 47  (15.7) | 240 (22.6) |
| 2 comorbidities | 275 (21.3) | 417 (23.9) | 40 (23.1) | 86 (21.8) | 373 (22.2) | 76 (25.5) | 118 (24.5) | 7  (21.9) | 20 (21.1) | 118 (19.2) | 199 (20.1) | 299 (23.7) | 33 (23.4) | 66  (22.0) | 255 (24.0) |
| 3+ comorbidities | 290 (22.5) | 755 (43.3) | 66 (38.2) | 97 (24.6) | 756 (45.0) | 102 (34.2) | 255 (53.0) | 19 (59.4) | 33 (34.7) | 367 (59.7) | 188 (19.0) | 500 (39.6) | 47 (33.3) | 64  (21.3) | 389 (36.6) |
| **Number with specific comorbidities ^c^, n (%)** |  |  |  |  |  |  |  |  |  |  |  |  |  |  |  |
| Asthma | 51  (4.0) | 146  (8.4) | 29 (16.8) | 34  (8.6) | 141 (8.4) | 9  (3.0) | 29  (6.0) | 6  (18.8) | 9  (9.5) | 35  (5.7) | 42  (4.2) | 117  (9.3) | 23 (16.3) | 25  (8.3) | 106 (10.0) |
| Lung disease | 242 (18.8) | 634 (36.4) | 66 (38.2) | 87 (22.0) | 708 (42.2) | 59 (19.8) | 188 (39.1) | 17 (53.1) | 23 (24.2) | 288 (46.8) | 183 (18.5) | 446 (35.3) | 49 (34.8) | 64  (21.3) | 420 (39.5) |
| Cardiovascular disease | 442 (34.3) | 683 (39.2) | 73 (42.2) | 123 (31.1) | 785 (46.8) | 94 (31.5) | 173 (36.0) | 14 (43.8) | 21 (22.1) | 303 (49.3) | 348 (35.1) | 510 (40.4) | 59 (41.8) | 102  (34.0) | 482 (45.3) |
| Hypertension | 491 (38.1) | 1010 (57.9) | 74 (42.8) | 122 (30.9) | 926 (55.2) | 104 (34.9) | 258 (53.6) | 13 (40.6) | 26 (27.4) | 327 (53.2) | 387 (39.1) | 752 (59.5) | 61 (43.3) | 96  (32.0) | 599 (56.3) |
| Chronic liver disease | 48  (3.7) | 118  (6.8) | 35 (20.2) | 23  (5.8) | 175 (10.4) | 18  (6.0) | 37  (7.7) | 10 (31.2) | 13 (13.7) | 75 (12.2) | 30  (3.0) | 81  (6.4) | 25 (17.7) | 10  (3.3) | 100  (9.4) |
| Chronic kidney disease | 100 (7.8) | 410 (23.5) | 23 (13.3) | 67 (17.0) | 304 (18.1) | 31 (10.4) | 98  (20.4) | 5  (15.6) | 21 (22.1) | 165 (26.8) | 69  (7.0) | 312 (24.7) | 18 (12.8) | 46  (15.3) | 139 (13.1) |
| Type 2 diabetes | 269 (20.9) | 533 (30.6) | 41 (23.7) | 81 (20.5) | 466 (27.8) | 58 (19.5) | 139 (28.9) | 6  (18.8) | 18 (18.9) | 174 (28.3) | 211 (21.3) | 394 (31.2) | 35 (24.8) | 63  (21.0) | 292 (27.4) |
| Cancer | 248 (19.2) | 389 (22.3) | 27 (15.6) | 59 (14.9) | 449 (26.7) | 248 (83.2) | 389 (80.9) | 27 (84.4) | 59 (62.1) | 449 (73.0) | 0  (0.0) | 0  (0.0) | 0  (0.0) | 0  (0.0) | 0  (0.0) |
| Main IC ^f^ | 73 (5.7) | 274 (15.7) | 6 (3.5) | 77 (19.5) | 302 (18.0) | 73 (24.5) | 274 (57.0) | 6 (18.8) | 77 (81.1) | 302 (49.1) | 0  (0.0) | 0  (0.0) | 0  (0.0) | 0  (0.0) | 0  (0.0) |

^a^ Alternative definition of IC (Table 1); ^b^ Among the patients eligible to receive booster completed primary series); c Not mutually exclusive; ^d^ The recording of individual SARI symptoms became mandatory after COVIDRIVE master protocol v4.0 (October 10, 2022), therefore records of SARI patients admitted to hospital before this date may lack that information; ^e^ Main SARI definition (Table 1); ^f^ Main IC definition (Table 1). Abbreviations: CI, confidence interval; CIRI-IT, Centro Interuniversitario di Ricerca sull'Influenza e le altre Infezioni Trasmissibili; GTPUH, Hospital Universitario Germans Trias i Pujol; HUVH, Hospital Universitari Vall d’Hebron; IC, immunocompromising condition; ICU, intensive care unit; IQR, interquartile range; n, number; SARI, severe acute respiratory infection; SD, standard deviation; UZA, Universitair Ziekenhuis Antwerpen. A square bracket “[“ and “]” in a range denotes that the number is included in the range, while regular brackets “(“ and “)” in a range means that the number is not included.

Figure S1. IC prevalence over time among hospitalized SARI, by SARS-CoV-2 test status, comparing different SARI and SARS-CoV-2 test status definitions

1. **
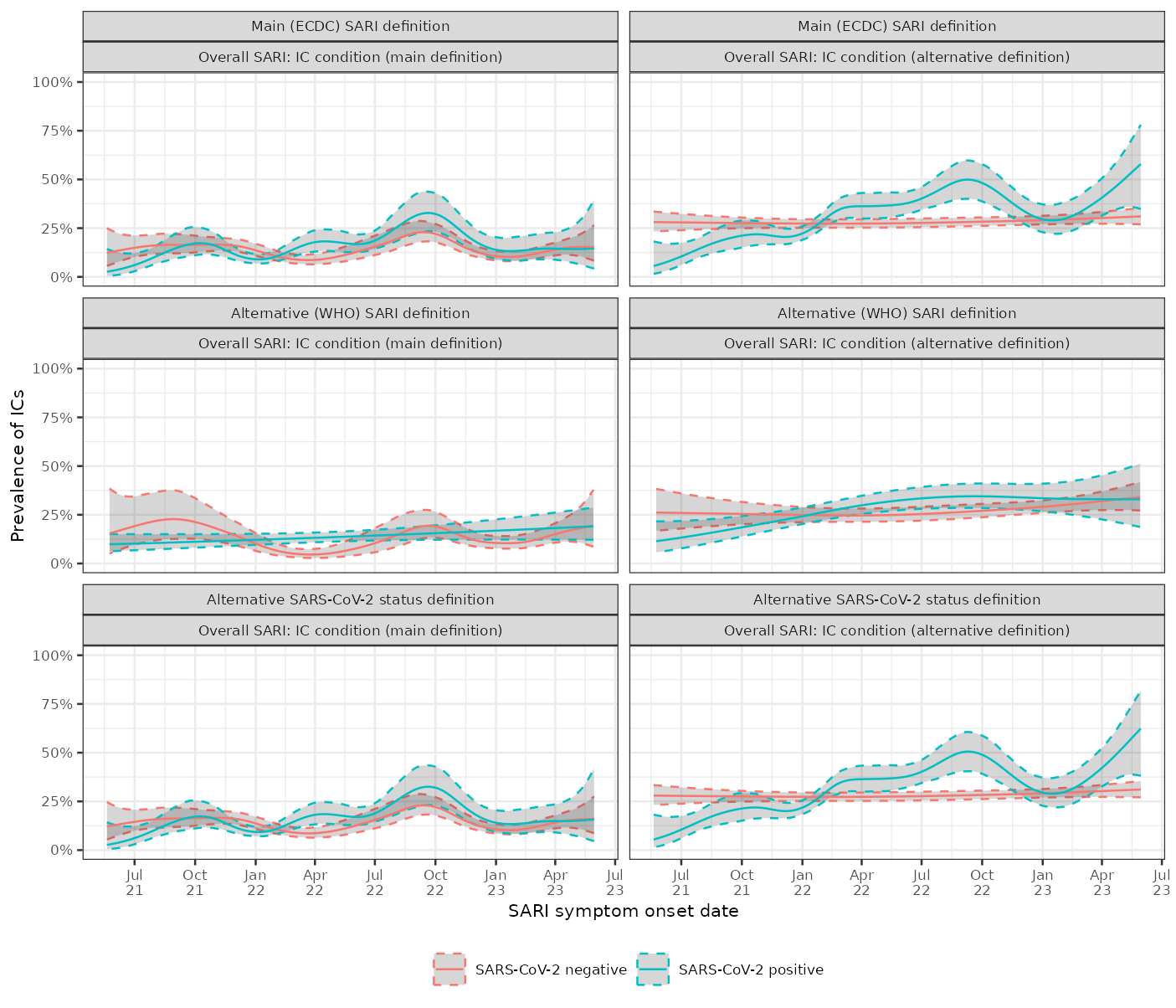
**

**b)**

**c)**

a) Main (ECDC) SARI definition with main SARS-CoV-2 test status definition (specimen collected up to 72h post admission); b) Alternative (WHO) SARI definition with main SARS-CoV-2 test status definition (specimen collected up to 72h post admission); c) Main (ECDC) SARI definition with alternative SARS-CoV-2 test status definition (swabbing up to 24h post admission). Abbreviations: IC, immunocompromising condition; SARI, severe acute respiratory infection; SARS-CoV-2, severe acute respiratory syndrome coronavirus 2; WHO, World Health Organization; 21, 2021; 22, 2022; 23, 2023.

Figure S2. Most frequent (25) combinations of chronic conditions in hospitalized SARI patients, by SARS-CoV-2 test status


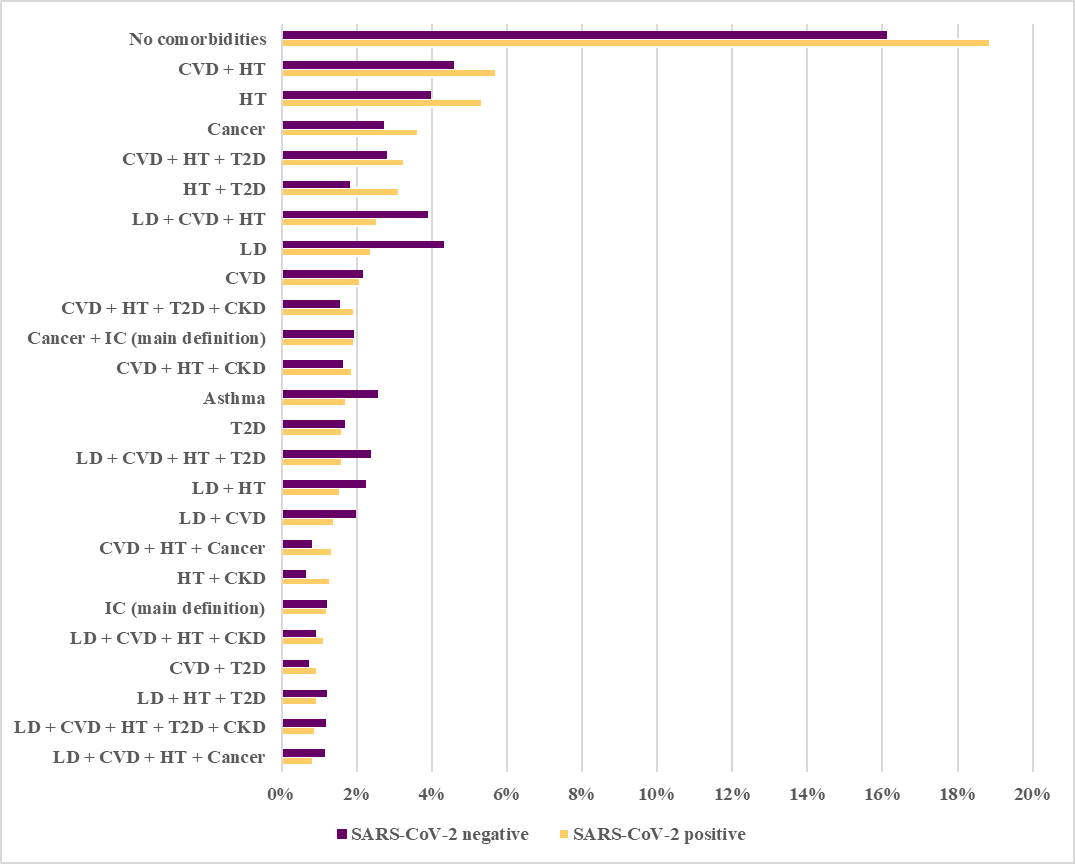


Abbreviations: CKD, chronic kidney disease; CVD, cardiovascular disease; HT, hypertension; IC, immunocompromising condition; LD, chronic liver disease; SARI, severe acute respiratory infection; SARS-CoV-2, severe acute respiratory syndrome coronavirus 2; T2D, Type 2 diabetes
